# Thermally symbiotic integration of osmotic membrane distillation and electrolysis for direct seawater hydrogen production

**DOI:** 10.1038/s41467-026-74854-8

**Published:** 2026-07-07

**Authors:** Gabriela Scheibel Cassol, Chii Shang, Paul Westerhoff, Yinghao Song

**Affiliations:** 1 Department of Civil and Environmental Engineering, The Hong Kong University of Science and Technology, Clear Water Bay, Kowloon, Hong Kong SAR, China; 2 Hong Kong Branch of Chinese National Engineering Research Center for Control & Treatment of Heavy Metal Pollution, The Hong Kong University of Science and Technology, Clear Water Bay, Kowloon, Hong Kong SAR, China; 3 School of Sustainable Engineering and The Built Environment, Arizona State University, Tempe, AZ USA; 4 Department of Building and Real Estate, The Hong Kong Polytechnic University, Hung Hom, Kowloon, Hong Kong SAR, China; 5 Department of Mechanical Engineering, The Hong Kong Polytechnic University, Hung Hom, Kowloon, Hong Kong SAR, China

**Keywords:** Chemical engineering, Energy storage, Hydrogen energy, Energy harvesting

## Abstract

Integrating water purification membranes with electrolysis for in situ hydrogen (H_2_) production from seawater offers a rapid pathway to net-zero, but is limited by salt crossover and insufficient water production in existing approaches. Here we overcome these limitations by integrating osmotic membrane distillation (OMD) with alkaline water electrolysis (AWE). Driven by dual thermal and osmotic gradients to enhance salt-free water vapour transport, the OMD-AWE delivers a H_2_ production rate of 60 kg m^–2^ day^–1^ with excellent stability over 500 h of continuous operation. To eliminate the external heating energy penalty of OMD, we propose a thermally symbiotic architecture that converts the AWE’s waste heat to OMD’s thermal driving force while OMD simultaneously providing cooling to maintain AWE optimal temperatures, as validated by our thermal-water-hydrogen model. This thermal symbiosis not only makes OMD-AWE energy self-sufficient with energy efficiency of 51 kWh kg(H_2_)^–1^ but also establishes a self-regulating mechanism that phase-locks thermal driving force to fluctuating electrical inputs, synchronising water supply with demand to overcome renewable intermittency. Our approach enables flexible component matching and thermal self-sufficiency at any scale, providing a framework for membrane-integrated electrolysis, demonstrating both technical excellence and economic viability towards a sustainable hydrogen economy.

## Abstract (web summary)

A seawater-to-hydrogen system combines membrane distillation with water electrolysis, using waste heat recycling for energy efficiency and salt-free operation with high hydrogen yields and excellent stability.

## Introduction

Power-to-hydrogen (P2H) systems convert excess electricity to green H_2_ via water electrolysis, offering a promising solution to manage intermittent renewable energy supply^1–3^. However, city-scale P2H intensifies freshwater stress, requiring 20–50 L of water for producing per kg of H_2_ from water splitting and electrolysis-equipment cooling^4–6^. Seawater presents an abundant alternative but requires desalination through reverse osmosis (RO) to remove impurities before electrolysis. This introduces an often-overlooked energy penalty. Although RO’s energy consumption is only 1% compared to water electrolysis^7^, this energy is permanently lost, whereas the majority of energy supplied to electrolysis is not lost but rather converted and stored as chemical potential energy within the H_2_ product itself. Minimising this energy loss is crucial, as every 0.1 kWh m^–3^ reduction in water treatment translates to megawatt-scale annual savings. The desalination challenge becomes even more pressing as P2H tends to utilise abundant wind resources offshore. RO requires large footprints, frequent maintenance, precise controls, and complex chemical handling infrastructure, making its installation on harsh marine environments impractical. Building coastal RO facilities adds substantial engineering costs and complexity. As an alternative, direct electrolysis of untreated seawater has attracted widespread interest, driving development of chlorine-resistant and selective catalysts and membrane separator^8,9^. Despite high scientific merit, these are far from commercial viability, unlikely contributing to 2050 net zero^10,11^.

Water purification membrane-integrated electrolysis is an emerging in situ approach that enables simultaneous seawater purification and H_2_ production, without requiring separation or collection of the purified water. Forward osmosis coupled with water splitting (FOWS_AWE_) uses a concentrated potassium hydroxide (KOH) solution that simultaneously acts as an osmotic agent to extract water and as an electrolyte to drive AWE, enabling continuously H_2_ production at a rate of 40.3 kg per m^2^ of membrane per day (kg m^−2^ day^−1^) from wastewater effluent^12^. However, its liquid-phase water transport mechanism threatens FOWS_AWE_ stability because small amounts of salts cross FO membranes alongside water (Supplementary Note 1), which reduces osmotic driving force, degrades membranes, and compromises both water purification and electrolysis. Integrating PTFE membranes to AWE (PTFE-AWE), also known as self-dampening electrolyte-assisted direct seawater electrolysis (SDE-DSE), eliminates salt passages by allowing only water vapour transport through their hydrophobic membrane pores, maintaining stability over 3200 h^13^. However, this approach produced H_2_ at a rate of 0.09% of FOWS_AWE_ due to insufficient water supply, limited by its isothermal osmotic distillation (OD) creating a small 0.24 kPa vapour pressure gradient (Δ*P*_v_). More critically, the direct coupling of OD with AWE creates inherent constraints for system optimisation and scaling, as both optimal water production and AWE conditions are not allowed. Heating the feed to activate thermally driven membrane distillation (MD) improves Δ*P*_v_ by >20 times (Supplementary Fig. 2), but its energy consumption is 17–70 times higher than RO, making it economically unfeasible. Crucially, the absence of external heat sources in remote or offshore environments is the fundamental barrier preventing SDE-DSE from overcoming its water supply limitations, severely restricting its geographical deployment and scalability. Furthermore, operating AWE at high temperatures raises KOH’s vapour pressure (*P*_v_) above that of the feed, causing reverse vapour transport. Pressurised H_2_ production is also impossible, as operating above PTFE’s liquid entry pressure (LEP, 200–500 kPa^14^) leads to liquid penetration and membrane wetting. Membrane-integrated electrolysis that simultaneously achieves high yield, long-term stability, and practical efficiency remains an unresolved challenge.

We hypothesise that thermal integration can create a symbiotic relationship between the PTFE membrane module and AWE, where each directly solves critical challenges of the other, allowing both to operate under optimal conditions without over-pressure and reverse flux issues. The waste heat from AWE, typically an operational burden requiring costly cooling, perfectly matches MD’s massive thermal demand. This synergy transforms two perceived weaknesses into strategic advantages by converting AWE’s waste heat into free energy to drive MD water purification, while MD’s thermal absorption capacity acts as a heat sink to cool AWE, enabling its stable and efficient operation under controlled temperatures. This approach is expected to create energy-efficient PTFE-AWE that maximises H_2_ output, prevents degradation from overheating and salt contamination. Therefore, identifying the optimal MD configuration and thermal condition presents a critical design choice, requiring comparison of temperature-dependent behaviours in water flux, fouling, and wetting among commonly used air gap MD (AGMD), direct contact MD (DCMD), and osmotic MD (OMD)^15–17^. These are distinguished by their permeate side, which consists of a gas layer, a water stream, and an osmotic agent, respectively. Simultaneously, defining AWE’s thermal compatibility is essential for maximising overall system efficiency.

**Fig. 1:**
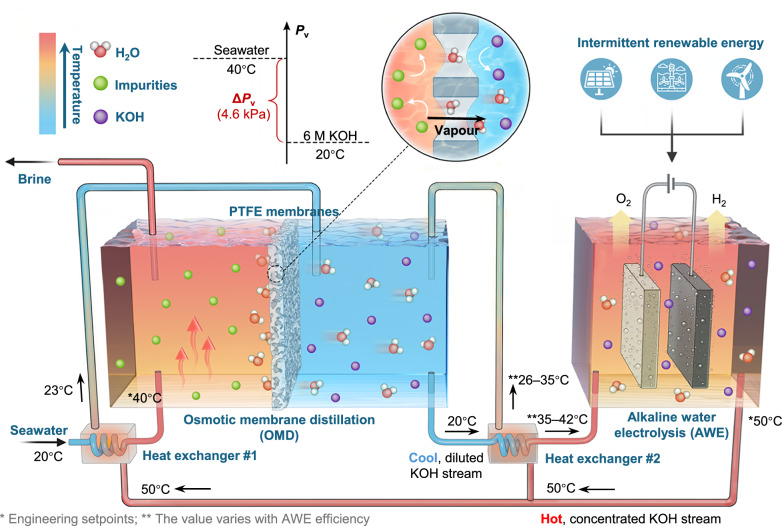
Direct green H_2_ production from seawater using a thermally symbiotic osmotic distillation-alkaline water electrolysis (OMD-AWE) system. The design utilises precisely designed self-sustaining heat exchange loops to convert the waste heat from AWE into the primary driver of the OMD process. This thermal integration enables OMD operation at an optimal 40 °C feed with 6 M KOH as osmotic agent to deliver high water flux with minimal fouling and wetting risks. In return, the OMD provides passive cooling and a continuous supply of pure water, enabling AWE to operate at 50 °C for efficient H_2_ production.

Here we present a thermally symbiotic architecture integrating OMD and AWE (Fig. 1). To establish the physical prerequisites essential for this symbiosis, we first experimentally determined the operational boundaries of the system. We identified 40  °C seawater feed paired with ambient 6 M KOH osmotic agent provides optimal OMD thermal conditions, delivering sufficient water flux over 20 L m^–2^ h^–1^ (LMH) for H_2_ production while minimising membrane scaling and wetting risks to maintain optimal long-term salt rejection. Its dual-driving-force ensures stable water production that resists latent heat losses during water vapour transport. By using external heating to maintain this optimal gradient, our OMD-AWE achieved a H_2_ output of 60.2 kg m^–2^ day^–1^, 4 times higher than FOWS_AWE_, while maintaining stability over 500 h of continuous operation. To eliminate the external energy penalties at scale, we designed self-sustaining heat exchange loops through thermodynamic modelling. This thermal symbiosis captures waste heat from AWE to heat the OMD’s feed to 40 °C, while simultaneously cooling the AWE to maintain its temperature at ~50  °C, ensuring high-efficiency, salt-free electrolysis. This integrated approach achieves energy consumption of 51.05 kWh kg(H_2_)^–1^ and a cost of $5.73 kg(H_2_)^–1^, competitive with pure-water electrolysis. Supported by a scalable and modular design, these findings establish the thermally symbiotic OMD-AWE as a high-performance, cost-competitive, and sustainable solution to water-energy nexus challenges.

## Results

### Thermal trade-offs in PTFE membrane water flux and stability

**Fig. 2:**
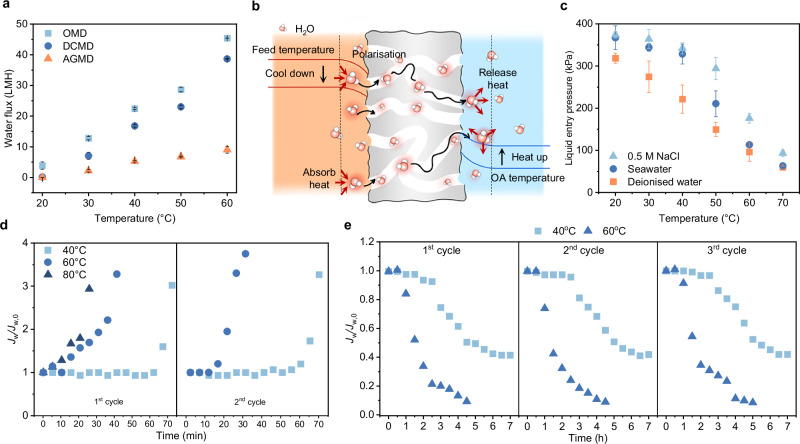
Temperature dependent trade-offs between water flux and stability in membrane distillation. **a** The comparison in water flux between OMD, direct contact membrane distillation (DCMD), and air gap membrane distillation (AGMD) at different feed temperatures; **b** a schematic showing thermal gradient loss from temperature polarisation at the membrane surface or axial temperature drop along its flow path due to latent heat transfer during water vapour transport; **c** Liquid entry pressure (LEP) as a function of feed temperature tested with pure water, 0.5 M NaCl, and real seawater; **d** real-time water flux to initial water flux (*J*_w_/*J*_w,0_) at different feed temperatures during accelerated wetting tests using 0.5 M NaCl feed containing 0.1 mM SDBS and 6 M KOH as osmotic agent; and **e** *J*_w_/*J*_w,0_ at different feed temperatures during accelerated scaling tests using a feed containing 30 mM Na_2_SO_4_ and 30 mM CaCl_2_ and 6 M KOH as osmotic agent. All error bars represent standard deviation from two independent replicates.

To identify the optimal thermal integration design for PTFE-AWE, we first investigated the effects of different MD configurations and temperatures on the key performance metrics of PTFE membranes including water flux, wetting resistance, and fouling behaviour. The water flux tests were conducted using a 0.5 M sodium chloride (NaCl) feed solution at various temperatures up to 60  °C (controlled by a water bath) against a permeate side maintained at room temperature (RT) of ~20  °C, which consisted of either a 6 M KOH osmotic agent for OMD, deionised water for DCMD, or ambient air for AGMD, with the results compared in Fig. 2a. Water flux for all configurations increased with temperature due to the exponential rise in Δ*P*_v_, confirming that thermally-driven MD enhances water production. OMD shows the highest water flux across all temperatures; for instance, at a representative temperature of 40  °C, OMD achieved a water flux of 22.44 LMH, which was 34% higher than DCMD and over 4 times greater than AGMD. In standalone MD systems where external heating is a major cost, AGMD is preferred for its high thermal efficiency because its air gap serves as a thermal insulator that minimises conductive heat loss, despite the slow diffusion of water vapour across it^16,17^. However, AWE integration changes these priorities. The availability of free waste heat and high water demand for electrolysis make AGMD the least suitable option, while DCMD and OMD are the primary candidates which use a cold liquid permeate in direct contact with membranes to immediately condense water vapour for higher water flux.

Unlike DCMD, which relies entirely on thermal gradients, OMD offers distinct advantages through its dual-driving-force mechanism. By utilising a 6 M KOH solution as osmotic agent, OMD creates an additional non-thermal 0.26 kPa vapour pressure depression on the permeate side due to the reduced water activity. This supplementary force results in a water flux that is consistently over 5 LMH higher than DCMD under identical thermal conditions. This directly translates into two operational benefits. First, OMD can operate at a lower temperature to match DCMD’s productivity (i.e., OMD produces 22.44 LMH water requires a feed temperature of 40  °C whereas DCMD needs over 50  °C), which reduces the risks of mineral scaling and membrane wetting. Second, the osmotic driving force makes OMD more resilient to thermal gradient loss caused by latent heat of evaporation. Each water molecule crossing the membrane must first absorb latent heat to vaporise at the hot feed surface, then release this heat upon condensing at the cooler permeate^18^. This intrinsic heat transfer is not an inefficiency but rather the core engine of the process, as vapour transport inherently cools the feed while heating the permeate. This phenomena introduces both temperature polarisation at the membrane surface and an axial temperature drop along its flow path (Fig. 2b), challenges that intensify with larger membrane areas and longer modules when scaling up the process. Although continuous reheating can compensate for this heat loss, it is not a viable option for our AWE integration, which relies on finite waste heat. This is precisely where the osmotic gradient becomes indispensable, providing a consistent driving force even as thermal gradients weaken along the module. This osmotic backup ensures stable water flux, making OMD uniquely suited for large-scale applications with limited or low-grade heat sources. Although OMD is overlooked for standalone water purification due to intensive energy consumption to regenerate its osmotic agent and separate the purified water^19^, this issue is eliminated when integrated with AWE as the permeated water is directly consumed by electrolysis.

To ensure stability of the selected OMD, a key challenge is preventing membrane wetting, where liquid feed penetrates its hydrophobic pores and compromises salt rejection^20,21^. The PTFE membrane’s resistance to wetting was evaluated by measuring its LEP (i.e., the minimum pressure required to force liquid through) at various temperatures for pure water, a seawater-equivalent 0.5 M NaCl solution, and real seawater (composition shown in Supplementary Table 12) (Fig. 2c). In general, the LEP for all these test liquids is highly sensitive to temperature, dropping over 75% with increasing liquid temperature from 20  °C to 70  °C, due to the decreased water surface tension and thermal softening of the PTFE polymer allowing the pores to stretch and enlarge under pressure^22^. A reduced LEP indicates increased susceptibility to wetting and reduced pressure tolerance, risking operational failure^23^. This forces the system to be operated at lower temperatures to maintain a safe wetting margin, which limits the water flux. However, the presence of dissolved salts in the NaCl solution and the seawater increases LEP by 17% over pure water at 20  °C, while providing better temperature stability up to 40  °C, with values maintaining above 300 kPa. These salt ions, surrounded by hydration shells, are repelled from the hydrophobic PTFE surface, creating a more ordered water layer at the interface that increases the contact angle and balances out the softening^24^. A tipping point was observed at 40  °C, beyond where the LEP began to drop sharply for both saline liquids,as the thermal energy becomes sufficient to overcome the salt-induced interfacial stability, shifting the primary failure mechanism to polymer softening. However, seawater showed a more rapid LEP decline than the NaCl solution at high temperatures, likely due to natural surfactants reaching their Krafft points (typically 15–50  °C for most ionic surfactants with alkyl chains ranging from C10 to C16^25^). Above these temperatures, such surfactants become highly soluble and form micelles instead of crystals or gels, allowing them to adsorb to the liquid-vapour interface at the pore entrances where they lower the local surface tension and enable liquid permeation at a lower pressure.

To validate these LEP findings, surfactant-accelerated wetting tests were conducted using a 0.5 M NaCl solution spiked with 0.1 mM sodium dodecylbenzenesulphonate (SDBS), a common C12 alkyl-chain ionic surfactant, at 40  °C, 60  °C, and 80  °C (Fig. 2d). The membrane performance was monitored through the ratio of real-time water flux to initial water flux (*J*_w_/*J*_w,0_), where a ratio of ~1.0 indicates stable operation and a sharp increase signals wetting. At 80  °C, immediate membrane wetting occurred, while at 60  °C the system maintained stability for only 10 min before wetting. Operation at 40  °C extended the stable period by 5 times compared to 60  °C. Membrane regeneration through pure water backwashing and oven drying restored performance for both 40  °C and 60  °C^20^. However, the 60  °C operation requires more frequent regeneration cycles, which not only consumes energy and purified water but also increases the risk of irreversible membrane damage, as each cycle may can accumulate residual surfactants that accelerate subsequent re-wetting. These findings support 40  °C as the optimal feed temperature, maintaining wetting resistance with reduced surfactant-induced issue.

Accelerated scaling tests using gypsum-supersaturated solutions (30 mM calcium chloride (CaCl_2_) and 30 mM sodium sulphate (Na_2_SO_4_)) were conducted to examine scaling behaviour at different temperatures. Gypsum was selected as a model scalant because its crystallisation is predominantly temperature-dependent, exhibits minimal sensitivity to pH adjustments, and forms more compact and adherent scale layers that are more resistant to simple acid cleaning than the softer and more manageable CaCO_3_ scales^26^. As shown in Fig. 2e, the membrane at 40 °C maintained a stable water flux for around 200 min before scaling began to impede water transport. Importantly, the scale was fully removed via a 30 min chemical wash with a 0.05 M solution of the chelating agent ethylenediaminetetraacetic acid (EDTA)^27^, recovering the initial flux over three consecutive cycles. An extended 20-cycle test further confirmed this regenerability, maintaining near-complete flux recovery and demonstrating that membrane scaling at 40  °C is highly reversible (Supplementary Figs. 6–8 and Table 13). The chemical inertness of PTFE ensures that such EDTA treatments induce no cumulative damage over long-term operation. While these accelerated tests utilised inorganic salts, real seawater contains natural organic matter (NOM) that mitigates inorganic scaling by transforming mineral deposits from invasive needle-like structures to spherical aggregates through adsorption at crystal growth sites (Supplementary Figs. 9–11 and Table 14)^28^. This natural inhibition prevents hard scale formation, resulting in a foulant layer that is easily reversed during routine maintenance. In contrast, at 60  °C, scaling behaviour is markedly more aggressive due to the inverse solubility of gypsum and kinetic acceleration of crystal nucleation and growth at the membrane interface^29^, leading to rapid scaling and a sharp flux decline within 100 min. Furthermore, the higher temperature promotes scale formation deeper within the membrane pores rather than just surface crystallisation^30^, reducing cleaning efficiency and potentially requiring longer cleaning times or higher EDTA concentrations for full recovery. Collectively, these scaling characteristics, combined with LEP analysis and wetting tests, further reinforce 40 °C as the optimal OMD temperature for AWE integration, offering the best balance between water flux and membrane stability to ensure zero salt contamination. This contrasts with drinking water MD systems that prioritises maximum output over 60  °C while occasional salt passage is acceptable for human health.

### Ultrafast and stable H_2_ production via OMD-AWE integration

With the optimal operating conditions for OMD established, the next step was to integrate it with AWE and validate its H_2_ output and stability. The key to continuous operation is to achieve a stable water balance, where the rate of water consumed by AWE (*Q*_AWE_) must match the rate of water permeating through the PTFE membrane (*Q*_PTFE_) as governed by Eq. 1.

1 $${{Q}_{{PTFE}}=S \cdot {c}_{m} \cdot \theta \cdot {\Delta P}_{v}=Q}_{{AWE}}={i}_{{cell}}\frac{{FE} \cdot {{N}_{{cell}} \cdot V}_{m}}{2F}$$

*Q*_PTFE_ is a function of the membrane area (*S*, m^2^), its permeation coefficient (c_m_, kg m^–2^ s^–1^ Pa^–1^), a polarisation coefficient (*θ*), and Δ*P*_v_ (Pa). Meanwhile, *Q*_AWE_ is proportional to the applied current of the AWE stack (*i*_cell_, A) with the proportionality constant incorporating Faradaic efficiency (*FE*), the number of cells in the AWE stack (*N*_cell_ = 5 in our setup), the molar volume of H_2_O (*V*_m_ = 0.018 L mol^–1^ at 296 K of T and 1 atm), and Faraday’s constant (*F* = 96,485 C mol^–1^). Balancing *Q*_PTFE_ and *Q*_AWE_ yielded a linear correlation (Eq. 2). To describe this system-level relationship, we define a metric, i.e., membrane area-specific current (*J*_m_), which is the AWE current required per unit of PTFE membrane area to maintain the water balance. This metric is crucial as it governs the system-level integration between OMD and AWE, and is fundamentally different from the electrolyser’s current density (i.e., current per electrode area), which is a component-level metric that describes electrochemical reaction rates at the catalyst surface. As Eq. 2 demonstrates, this relationship simplifies to a linear correlation where Jm is directly proportional to Δ*P*_v_ via a constant slope (*Φ*) which is determined by all constant parameters of *c*_m_, *θ*, *FE*, *N*_cell_, *F*, and *V*_m_.

2 $${J}_{m}=\frac{{i}_{{cell}}}{S}=\frac{2F}{{{FE} \, N}_{{cell}}{V}_{m}}{c}_{m}\theta \cdot {\Delta P}_{v}=\varPhi \cdot {\Delta P}_{v}$$

**Fig. 3:**
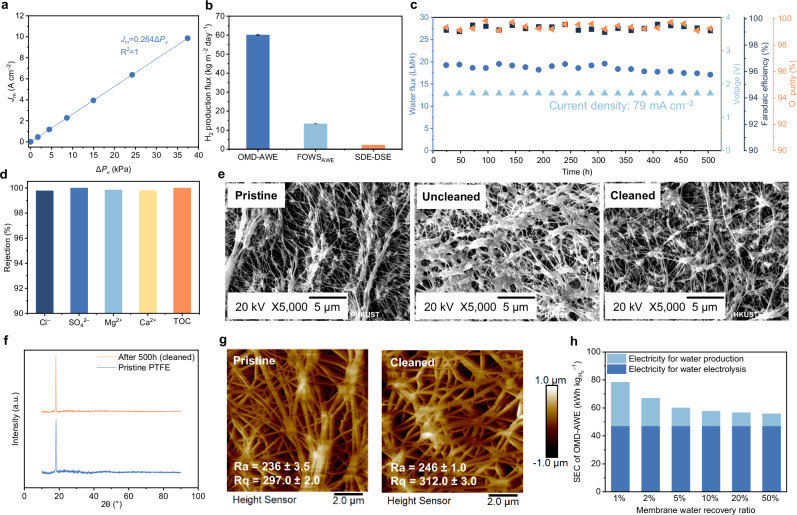
Key performance metrics of OMD-AWE. **a** membrane area-specific current (*J*_m_) as a function of vapour pressure gradient (Δ*P*_v_) to maintain water-hydrogen balance for continuous OMD-AWE operation (R^2^ = 1); **b** comparative H_2_ production flux of OMD-AWE, FOWS_AWE_, and SDE-DSE; **c** a 500-h continuous OMD-AWE operation tracking water flux, cell voltage, Faradaic efficiency, and O_2_ purity (Conditions: [feed] = seawater, [KOH] = 6 M, *T*_feed_ = 40 °C, *T*_KOH_ = 20 °C); **d** the rejection of chloride (Cl^–^), sulphate (SO_4_^2–^), calcium (Ca^2+^), magnesium (Mg^2+^), and total organic carbon (TOC) at the end of the 500 h operation; **e** SEM images showing the surface morphology of the pristine PTFE membrane, the uncleaned PTFE membrane after 500-h operation, and the cleaned PTFE membrane after 500 h operation; **f**, **g** XRD and AFM comparing the pristine PTFE membrane with the cleaned membrane after 500 h operation, respectively; and **h** specific energy consumption (*SEC*) of OMD-AWE with different membrane water recovery ratios. All error bars represent standard deviation from two independent replicates.

For these constant parameters, only *c*_m_, *θ*, and *FE* are unknown, but the product of *c*_m_ and *θ*, which represents the effective membrane permeability, can be experimentally determined by measuring *Q*_PTFE_ at specific Δ*P*_*v*_ and *S*, while *FE* can be obtained by comparing the experimentally measured H_2_ production rate (*r*_H2_) with the theoretical *r*_H2_ under a same *i*_cell_ using a separate AWE electrolyser (“Methods” Eq. 16). With *Φ* determined to be 0.264 (Fig. 3a), this model serves as a predictive tool for module design. We can predict the required *i*_cell_ to match any OMD operating condition or determine *S* to match any AWE setup by simply calculating Δ*P*_v_, which is a function of temperature and concentration of the feed and osmotic agent as described by Clausius–Clapeyron equation and Raoult’s law, respectively^31,32^ (details see Supplementary Note 2). It is important to note that this model uses current rather than current density as it offers versatility across electrolyser systems. Commercial or custom-built electrolysers vary in materials and architecture, creating different current density distributions. By reporting absolute current, researchers can easily adapt our model to their specific configurations, enhancing experimental reproducibility.

Guided by this model, the suggested OMD configuration was then integrated with AWE (polarisation curve of our 5-cell AWE stack is shown in Supplementary Fig. 12) to determine H_2_ output and operational stability. Our OMD-AWE with 0.5 M NaCl feed at 40  °C (maintained by an external water bath) and a 6 M KOH osmotic agent, which shows a water flux of 22.44 LMH (Fig. 2a), driven by 4.6 kPa Δ*P*_v_, requires *J*_m_ of 1.34 A cm^–2^ (Fig. 3a). Due to the fixed geometry of our custom-made membrane cell (*S* = 1 cm^2^) water was generated at a rate of 0.037 mL min^−1^. To strictly maintain the system’s water balance, the AWE current was used as the adjustable parameter and set to 1.34 A to exactly consume this produced volume. Consequently, the 5-cell AWE stack operated at a partial current density of 0.089 A cm^−2^. If operated at an industrial current density of 0.5 A cm^−2^ (which requires a total current of 7.6 A), the membrane would scale to 5.67 cm^2^. This validates the water balance principle, demonstrating that in real-world engineering, the membrane area can be flexibly sized to match any target electrolyser capacity. For subsequent continuous operation, the H_2_ production flux (i.e., H_2_ production rate per m^2^ of membrane) was measured by water displacement after separation from the KOH electrolyte via a custom-built degassing tower (Supplementary Fig. 13) and compared with other competing integrated systems (Fig. 3b). The OMD-AWE exhibited a H_2_ production flux of 60.2 kg m^–2^ day^–1^, which was over 25 times higher than that of isothermal SDE-DSE (or OD-AWE), attributed to a 20-fold increase in Δ*P*_v_ from heating the feed to 40 °C^12,13,33^. The H_2_ flux was also 4 times higher than that of previous FOWS_AWE_ when fed with 0.5 M NaCl, demonstrating that the combined thermal and osmotic gradient provides a far more potent driving force for water transport than isolated osmotic potential in liquid-phase systems^12,13,33^.

We experimentally tested this externally heated OMD-AWE system for 500 h with real seawater to assess long-term material and operational stability (Fig. 3c). The electrochemical performance remained stable with cell voltage stabilised at 1.72 V under constant current of 1.13 A, Faradaic efficiency >99%, and O_2_ purity >99% throughout the entire experiment. These results confirm the complete absence of parasitic chlorine evolution^34^, which is suppressed because OMD rejected >99% salts (Fig. 3d), maintaining a highly alkaline 6 M KOH environment that thermodynamically favours oxygen evolution. The sustained >99% O_2_ purity, even under low-current conditions, confirms no gas crossover^35^, as the ZIRFON diaphragm prevents internal gas mixing and our degassing tower eliminates entrained microbubbles within the KOH loop. Concurrently, the OMD demonstrated a stable water flux of 19.05 ± 0.41 LMH for the first 300 h before gradually declining to 17.79 ± 0.42 LMH by the end of the test (Fig. 3c). This minor decline is attributed to initial fouling formation, as suggested by scanning electron microscopy (SEM) characterisation of the of the uncleaned membrane surface after 500-h operation (Fig. 3e). Energy-dispersive X-ray spectroscopy (EDX) identified this foulant layer as a mixture of inorganic scale (Ca, Mg, S, and O at 2.32 atom%) and carbonaceous deposits, evidenced by a decrease in the surface fluorine-to-carbon (F/C) atomic ratio from 1.75 to 1.53 (Supplementary Fig. 14 and Table 15). Since the 500-h test utilised raw biologically active seawater, these carbonaceous deposits might include marine microorganisms and their hydrophilic extracellular polymeric substances (EPS). Operating at a moderate 40 °C avoids the microbial thermal stress typical of high-temperature MD (60–70 °C), which triggers EPS hyper-secretion and catastrophic biofouling^36,37^. This ensures a thin, stable biological interface. Importantly, this mixed fouling layer proved highly reversible after EDTA-based chemical wash. Post-cleaning EDX analysis showed that the inorganic scaling elements dropped to 0 atom% and the F/C ratio recovered to 1.69, indicating the complete removal of all mineral scale and the majority of the carbonaceous deposits. X-ray photoelectron spectroscopy (XPS) confirmed this reversibility, showing significantly diminished Ca 2*p*, Mg 1 *s*, C 1 *s*, and O 1 *s* peaks after cleaning (Supplementary Fig. 15). To remove residual organic foulants and prevent long-term biofilm accumulation, widely used high-pH alkaline cleaners could be used in addition to EDTA cleaning^38^. Furthermore, incorporating antiscalant chemicals into the feed or implementing basic prefiltration (e.g., sand filters) can also reduce scaling risk and extend cleaning intervals^39^.

Beyond reversible fouling, the PTFE membrane exhibited exceptional thermomechanical and chemical stability. SEM and ImageJ analysis of the cleaned membrane revealed an intact node-and-fibril microstructure with no evidence of pore collapse or widening (Fig. 3e, Supplementary Fig. 16). X-ray diffraction (XRD) and atomic force microscopy (AFM) confirmed that bulk crystallinity and nanoscale roughness remained virtually unchanged (Fig. 3f, g), ruling out any polymer etching or degradation caused by microbial metabolism. XPS further verified chemical integrity, showing perfectly matched carbon–fluorine and elemental fluorine spectra with no evidence of defluorination or chlorine incorporation. Utilising its intrinsic elasticity under near-atmospheric pressure, the membrane safely absorbed the continuous 20  °C thermal gradient, prevented any micro-cracking, delamination, or seal failure, as evidenced by the sustained high salt rejection (Fig. 3d) and stable KOH concentration (Supplementary Fig. 17). Analysis of the electrolyser electrodes confirmed their pristine condition and complete protection from seawater contaminants. Both SEM and EDX analyses revealed that the Ni nanowire cathode and NiFe anode retained their structural integrity and original elemental composition, with no surface fouling or traces of contaminants such as calcium, magnesium, or silica (Supplementary Figs. 18–19 and Table 16). In addition, our physically decoupled modular design provides crucial operational flexibility. This allows either the OMD or AWE to be taken offline for routine maintenance (e.g., membrane cleaning) without system failure. Since membrane fouling depends on cumulative permeate volume rather than strictly operational time, our 500-h test at >25-fold higher water flux than SDE-DSE extracted permeate equivalent to >12,500 h of isothermal operation, nearly 4 times that of previous 3200-h demonstrations. Combined with the proven reversibility of such accelerated fouling, this represents a significantly more rigorous validation of membrane durability, confirming that the system can sustain robust operation well beyond 500 h through routine maintenance.

Despite the high H_2_ flux and stable operation, OMD-AWE requires an additional sensible heat input of 0.023 kWh to heat each kg of feed water from RT to 40  °C, alongside a latent heat input of 0.833 kWh to drive each kg of water across the membrane. Given the common industry benchmark of 10 kg of water per kg of H_2_ produced, the additional energy required by external heating (*E*_addl_, kWh kg(H_2_)^–1^) is calculated by Eq. 3:

3 $${E}_{{addl}}=10\frac{k{g}_{{H}_{2}O}}{{{kg}}_{{H}_{2}}}\times 0.833\frac{{kWh}}{k{g}_{{H}_{2}O}}+\frac{10\frac{k{g}_{{H}_{2}O}}{{{kg}}_{{H}_{2}}}}{{RR}}\times 0.023\frac{{kWh}}{k{g}_{{H}_{2}O}}$$

Thus, the overall energy penalty increases exponentially with a decreasing water recovery ratio (RR, the ratio of water production rate to feed flowrate) as shown in Fig. 3h, representing a significant cost issue as current OMD systems usually operate at a RR of less than 10%^40,41^. At a RR of 10%, the energy penalty amounts to 10.6 kWh kg(H_2_)^–1^ corresponding to a 22.6% increase relative to the baseline 47.1 kWh kg(H_2_)^–1^ for water splitting using our electrolyser. This penalty escalates dramatically to 31.3 kWh kg(H_2_)^–1^ under a lab-scale RR (e.g., <1%). Consequently, OMD-AWE’s specific energy consumption (*SEC*), i.e., energy consumption per kg of H_2_ produced, exceeds those of SDE-DSE and FOWS_AWE_ by up to 69%, making energy efficiency improvements essential for economic viability.

### Thermal self-circulation improves OMD-AWE energy efficiency

**Fig. 4:**
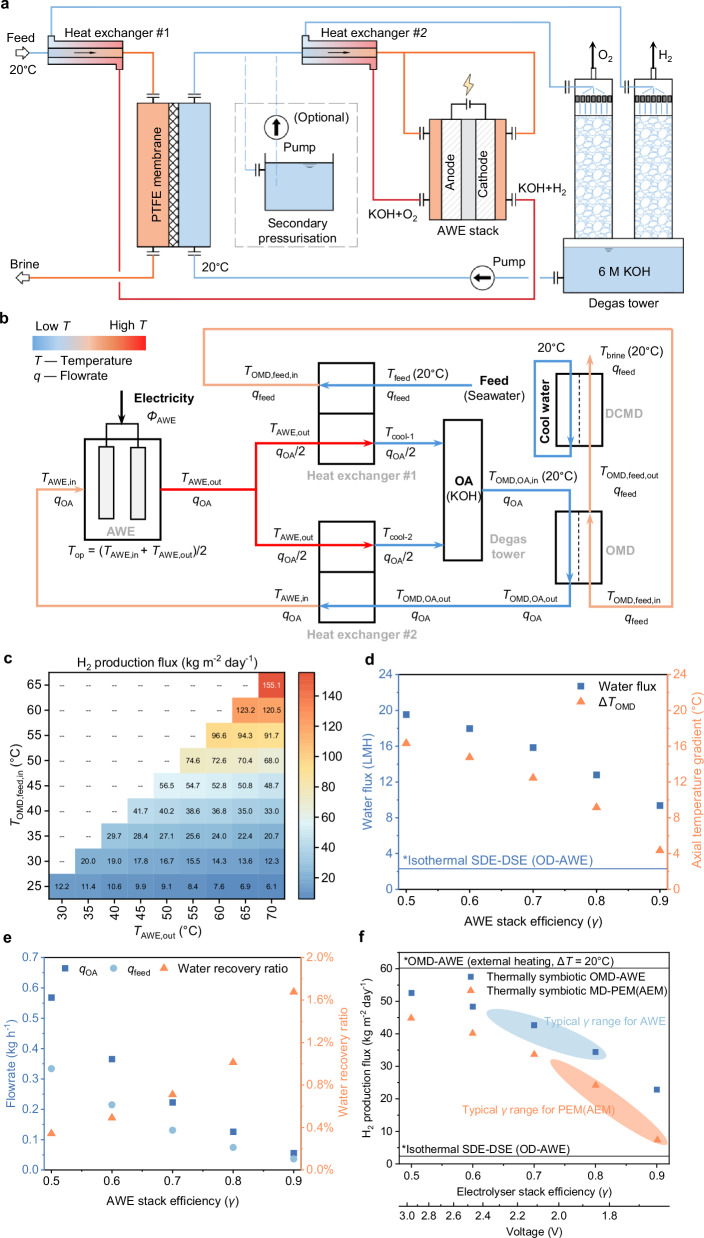
A thermal self-circulation design for OMD-AWE. **a** Precisely designed heat exchange loops that capture waste heat from AWE to drive OMD while provide cooling for AWE; **b** schematic diagram showing the thermal balance and stream flow paths throughout the loops; **c** H_2_ production flux as functions of AWE outlet temperature (*T*_AWE,out_) and OMD’s feed inlet temperature (*T*_OMD,feed,in_) as two controlling points for achieving thermal self-circulation; **d** the actual water flux (left axis) and axial temperature gradient (Δ*T*_OMD_) (right axis) and as a function of AWE stack efficiency (*γ*) under thermally symbiotic architecture, with horizontal reference line showing the water flux of isothermal SDE-DSE; **e** feed flowrate (*q*_feed_) and OA flowrate (*q*_OA_) (left axis), with corresponding water recovery ratio (right axis), as a function of *γ* under thermally symbiotic architecture; **f** the comparison in H_2_ production flux between the thermally symbiotic OMD-AWE and MD-PEM/AEM systems as a function of electrolyser *γ* or cell voltage, while the shaded regions represent typical efficiency ranges for AWE (blue) and PEM/AEM (orange) electrolysers and horizontal reference lines show the H_2_ flux of OMD-AWE with external heating (upper line) and isothermal SDE-DSE (lower line), respectively.

A potential approach to reduce the energy footprint of OMD-AWE involves harnessing the waste heat from AWE to power OMD, because AWE is an exothermic process that generates heat due to irreversible chemical reactions and ohmic resistance^42^. While cooling systems are generally required to prevent electrolyser overheating and degradation^43,44^, we propose an integrated dual heat exchanger system that simultaneously recovers waste heat and provides necessary cooling (Fig. 4a). The system captures thermal energy from the two gas-containing KOH streams existing the AWE stack. One heat exchanger heats the feed for OMD operation, while the second preheats the KOH osmotic agent (OA) returning from OMD to the AWE stack. Subsequently, the two cooled KOH streams flow to a degas tower for H_2_ and O_2_ collection, while the reconcentrated and degassed KOH stream is recirculated back to the OMD unit, forming a closed-loop circulation.

To assess if the waste heat can sustain thermal self-circulation, we developed a thermal-water-hydrogen model to quantify the system’s thermal balance as demonstrated in Fig. 4b. The waste heat flow generated from AWE (*Φ*_AWE_) due to electrical inefficiency is captured by the KOH stream passing through the electrolyser (flowrate, *q*_*OA*_), increasing its temperature from inlet (*T*_AWE,in_) to outlet (*T*_AWE,out_) as expressed in Eq. 4. This captured heat elevates the AWE’s operating temperature (*T*_op_, the average of *T*_AWE,in_ and *T*_AWE,out_), with *T*_AWE,out_ controlled as an engineering setpoint to prevent overheating depended on materials’ thermal tolerance.

4 $${\varPhi }_{{AWE}}=\left(U-{U}_{{th}}\right){i}_{{cell}}{N}_{{cell}}={{SEC}}_{t}\left(\frac{1-\gamma }{\gamma }\right){r}_{{H}_{2}}={c}_{{KOH}}{q}_{{OA}}\left({T}_{{AWE},{out}}-{T}_{{AWE},{in}}\right)$$

5 $${r}_{{H}_{2}}={c}_{m}{\theta \Delta P}_{v} \cdot \rho \frac{{M}_{{H}_{2}}}{{M}_{{H}_{2}O}}S$$

*U*_th_ (1.48 V) represents the thermoneutral voltage for water electrolysis, which translates to theoretical *SEC* (*SEC*_t_) of 39.4 kWh kg^−1^ based on high heat value (HHV) of H_2_. *U* could be determined from AWE polarisation curves as functions of *i*_cell_ and *T*_op_. *γ* represents electrolyser stack efficiency, which equals to *U*_th_/*U*. *r*_H2_ is the H_2_ production rate as a function of water flux (determined by *c*_m_, *θ*, and ∆*P*_v_), water density (*ρ*), molar mass (*M*) of H_2_ and water, and *c*_KOH_ is the specific heat capacity of 6 M KOH (3.50 kJ kg^−1^ K^−1^) (Eq. 5). The two heated KOH streams, flowing at equal flowrates, then heat the feed (with flowrate *q*_feed_ and temperature *T*_feed_) from RT to the target OMD inlet temperature (*T*_OMD,feed,in_), and heat the recirculated KOH leaving the OMD module at *T*_OMD,OA,out_ to AWE inlet at *T*_AWE,in_ through two heat exchangers (Hx1 and Hx2), respectively (Eqs. 6–7).

6 $${\varPhi }_{{Hx},1} 	=\varepsilon \cdot {c}_{{KOH}}\frac{{q}_{{OA}}}{2}\left({T}_{{AWE},{out}}-{T}_{{feed}}\right)={c}_{{seawater}}{q}_{f{eed}}\left({T}_{{OMD},{feed},{in}}-{T}_{{feed}}\right) \\ 	={c}_{{KOH}}\frac{{q}_{{OA}}}{2}\left({T}_{{AWE},{out}}-{T}_{{cool},1}\right)$$

7 $${\varPhi }_{{Hx},2} 	=\varepsilon \cdot {c}_{{KOH}}\frac{{q}_{{OA}}}{2}\left({T}_{{AWE},{out}}-{T}_{{OMD},{OA},{out}}\right)={c}_{{KOH}}{q}_{{OA}}\left({T}_{{AWE},{in}}-{T}_{{OMD},{OA},{out}}\right) \\ 	={c}_{{KOH}}\frac{{q}_{{OA}}}{2}\left({T}_{{AWE},{out}}-{T}_{{cool},2}\right)$$

where *c*_seawater_ is the specific heat capacity of seawater (4.02 kJ kg^−1^ K^−1^)^45,46^, *Φ*_hx_ is the heat flow through heat exchangers, *T*_cool_ is the KOH temperature exiting the heat exchangers, and *ε* is the heat exchanger effectiveness (i.e., the ratio of actual heat transfer to maximum possible heat transfer), >90% using a counter-flow plate-type configuration^47,48^. The impact of *ε* on system performance is detailed in Supplementary Note 3. Both pipes and heat exchangers could be wrapped with aluminum-clad polyurethane foam insulation, resulting in negligible heat losses (Supplementary Note 4). On the other hand, the heated seawater enters OMD and drive water vapour transport, where the heat flow through the membrane (*Φ*_OMD_) consists of the portion carried by vapour (*Φ*_vapour_) and that by membrane conduction (*Φ*_mc_), as expressed in Eqs. 8–10.

8 $${\varPhi }_{{OMD}}={\varPhi }_{{vapour}}+{\varPhi }_{{mc}}=\left({{c}_{m}\theta {\Delta P}_{v}\Delta H}_{v}+U{\Delta T}_{{OMD}}\right) \cdot S$$

9 $${\varDelta T}_{{OMD}}=\frac{\left({T}_{{OMD},{feed},{in}}-{T}_{{OMD},{OA},{out}}\right)-\left({T}_{{OMD},{feed},{out}}-{T}_{{OMD},{OA},{in}}\right)}{{{\mathrm{ln}}}\left[\left({T}_{{OMD},{feed},{in}}-{T}_{{OMD},{OA},{out}}\right)/\left({T}_{{OMD},{feed},{out}}-{T}_{{OMD},{OA},{in}}\right)\right]} \cdot \theta$$

10 $$\frac{1}{U}=\frac{1}{{h}_{{feed}}}+\frac{t}{{k}_{{membrane}}}+\frac{1}{{h}_{{OA}}}=\frac{1}{{h}_{{feed}}}+\frac{t}{{k}_{{PTFE}} \cdot \frac{2\left(1-\varphi \right)+3\varphi \left({k}_{{air}}/{k}_{{PTFE}}\right)}{3-\varphi+2\varphi \left({k}_{{air}}/{k}_{{PTFE}}\right)}}+\frac{1}{{h}_{{OA}}}$$

*Φ*_vapour_ is proportional to water flux (which is a function of *c*_m_, *θ*, and Δ*P*_*v*_) via latent heat of vaporisation (Δ*H*_v_, 2400 kJ kg^−1^) and OMD’s membrane area (*S*_OMD_ = 1 cm^2^ with our setup). *Φ*_mc_ depends on the overall heat transfer coefficient (*U*) and axial temperature gradient (Δ*T*_OMD_) along the module length considering counter-current flow and adjusted by a *θ* of 0.6 (Eq. 9). *U* incorporates the convective heat transfer (*h*) of both feed and OA (set at 1,000 W m^−2^ K^−1^ for laminar flow), and the membrane conductive resistance which equals its thickness (*t*, 65 μm) divided by effective thermal conductivity (*k*_membrane_) (Eq. 10). The *k*_membrane_ accounts for both the conduction through PTFE polymer (*k*_PTFE_, 0.25 W m^−1^ K^−1^) and pore-filling air (*k*_air_, 0.026 W m^−1^ K^−1^) weighted by the membrane porosity (*φ*, 0.8). Obtaining *Φ*_OMD_ allows to determine the temperature changes of feed and OA after OMD through Eq. 11.

11 $${\varPhi }_{{OMD}}={c}_{{seawater}}{q}_{{feed}}\left({T}_{{OMD},{feed},{in}}-{T}_{{OMD},{feed},{out}}\right)={c}_{{KOH}}{q}_{{OA}}\left({T}_{{OMD},{OA},{out}}-{T}_{{OMD},{OA},{in}}\right)$$

By solving Eqs. 4–11 with treating *T*_OMD,feed,in_ and *T*_AWE,out_ as input varibles, we simulated how thermal integration improves H_2_ yields of a commercial single-cell AWE electrolyser (0.5 A cm^−2^, nanowire/NiFe electrodes and ZIRFON diaphragm) integrated with a 1 cm^2^ membrane (Fig. 4c). Increasing *T*_OMD,feed,in_ boosts H_2_ flux due to increased water supply, while increasing *T*_AWE,out_ decreases H_2_ flux at any fixed *T*_OMD,feed,in_ because higher AWE temperatures improve electrochemical kinetics but reduces waste heat generation. To maintain *T*_OMD,feed,in_, in despite reduced heat input, the system must decrease OA flowrate, which results in a larger temperature drop across the membrane due to latent heat transfer and reduces water flux. Our earlier trade-off analyses established that operating OMD above 40  °C risks membrane scaling and wetting; therefore, *T*_OMD,feed,in_ must be capped at 40  °C to ensure long-term durability. Along the 40 °C feed boundary, the system still delivers exceptional H_2_ fluxes over 40 kg m^−2^ day^−1^ with *T*_AWE,out_ controlled at ~50  °C. This demonstrates that AWE does not need high temperatures but a 5–10 °C higher temperature represents the thermodynamic optimal window that balances high H_2_ yields with membrane stability. While PTFE membranes provide a robust baseline for OMD-AWE integration, emerging membrane materials offer opportunities for system upgrading, such as low-friction carbon nanotubes for faster vapour transport, photothermal coatings for improved thermal efficiency, and omniphobic surfaces for wetting resistance^49–54^. Future membrane development could prioritise engineering these functionalities with alkali-stable materials that withstand the hot KOH environment, guided by advanced in situ/operando characterisations (e.g., Raman and X-ray scattering, optical visualisation techniques)^55–59^. These techniques will be crucial to verify the membrane’s shielding effect on the catalyst’s dynamic surface structure, while monitoring real-time membrane scaling and pore wetting under dynamic thermal and chemical stress.

As OMD relies on AWE waste heat, a critical concern is whether this thermally symbiotic architecture can be sustained across highly efficient electrolysers and different configurations. Electrolysers vary widely in electrode materials, cell architectures, and current densities, which dictate their overall *γ* and available waste heat. Therefore, we modelled the thermal balance for self-circulation as a function of *γ* as shown in Fig. 4d–f (key operating parameters are listed in Supplementary Table 17, and thermal balance is validated in Supplementary Fig. 20). For a less efficient AWE stack (*γ* = 0.5) that provides abundant waste heat, the system maintains a robust Δ*T*_OMD_ of 15.8 °C, resulting in an acceptable water flux penalty of just 13% relative to the ideal 22.44 LMH achieved by external heating (Fig. 4d). Conversely, a highly efficient AWE (*γ* = 0.9) produces minimal waste heat, making the OMD highly susceptible to latent heat loss, reducing Δ*T*_OMD_ to 4.3 °C and limiting the water flux to only 9.3 LMH. This variation in water flux is fundamentally governed by the thermodynamic constraints placed on the system’s flow rates (*q*_feed_ and *q*_OA_) to achieve thermal self-circulation. To maintain optimal temperature setpoints (*T*_OMD,feed,in_ = 40 °C and *T*_AWE,out_ = 50 °C), the flow rates must be precisely matched to the available waste heat (Fig. 4e). If the flowrates are too high, the limited thermal energy is insufficient to heat the streams to their target temperatures. Conversely, their flowrates cannot be too low. A lower *q*_OA_ increases its residence time in the AWE cell, causing OA to absorb excessive heat beyond the materials’ thermal tolerance. Similarly, a lower *q*_feed_ increases residence time in heat exchangers, causing feed temperature to exceed the optimal 40  °C and creating fouling and wetting risks. While reducing the heat exchange area could control the feed at 40  °C, the resulting insufficient thermal load would be quickly consumed by vaporisation, making Δ*T*_OMD_ to collapse and thus compromising water flux for H_2_ production or, more likely, lead to a complete system failure. Note that these flowrates are reported for a 1 cm^2^ membrane area and scale linearly with membrane size. Our experimental validation integrating two heat exchangers in OMD-AWE provides empirical evidence supporting the feasibility of thermal self-circulation (Supplementary Fig. 22).

Our strategic use of low RRs is the key that unlocks the thermal symbiosis, a design choice that is the direct opposite of the standalone desalination paradigm. In standalone systems, high water recovery is paramount as their primary costs and limiting resources are the pretreated feed water, extensive pumping and heating energy, and the high-salinity brine disposal. This forces them into a low-flowrate regime that is incapable of capturing large-scale, low-grade heat sources. Our design is optimised for maximum system-level energy efficiency and stability. This paradigm shift is precisely why a low RR becomes a powerful asset. By using abundant, untreated seawater in a high-flowrate, low-recovery loop, we create a powerful heat transfer engine. This allows the seawater to capture and productively use the AWE’s waste heat as primary driver for efficient and stable water production. The high water flux minimises the required membrane area to just 10 m^2^ per MW of AWE capacity, resulting in a compact physical footprint (<1 m^2^) by using high packing-density membrane modules that are negligible compared to the electrolyser installation. Another advantage of the low RR design is the simplification of brine management. It allows to produce brine with minimal salinity increase of <5% (Supplementary Fig. 23), falling within natural coastal salinity variations and enabling environmentally safe, direct ocean discharge without costly treatment processes. Ultimately, this design trades a negligible increase in low-pressure pumping energy for substantial gains in energy efficiency, operational stability, and simplified brine management.

These findings suggest a significant opportunity for commercial AWE stacks (*γ* = 0.62–0.82). Within this real-world range, our system is thermally self-sustaining with H_2_ fluxes of 30–45 kg m^−2^ day^−1^ (Fig. 4f) while co-producing 40% more clean water beyond electrolysis needs (Supplementary Figs. 24–25). Even under extreme high-efficiency scenario (*γ* = 0.9), OMD-AWE still achieves 5.6 times higher H_2_ yields than the baseline SDE-DSE. Beyond AWE, this thermal integration is adaptable to Proton Exchange Membrane (PEM) and Anion Exchange Membrane (AEM) electrolysis, as the chemically inert PTFE membrane is compatible with both acidic and dilute alkaline environments. However, MD-PEM and MD-AEM consistently underperform OMD-AWE due to the absent or less concentrated electrolyte for osmotic driving force. Typical PEM and AEM systems (*γ* = 0.74–0.93) yield less waste heat, resulting in lower H_2_ fluxes (<35 kg m^−2^ day^−1^). Although AWE’s lower efficiency is viewed as a drawback compared to PEM/AEM technologies, our thermally symbiotic architecture inverts this inefficiency into a powerful thermodynamic asset. Combined with AWE’s low cost, high durability, and technological maturity, this makes it the optimal choice. In contrast, highly pursued PEM/AEM systems underperform in this integrated context, requiring higher current densities to yield equivalent H_2_ as AWE systems. It should be noted that while the thermal self-circulation feasibility is validated through our modelling, laboratory-scale execution faces heat loss constraints due to unfavourable surface-to-volume ratios (Supplementary Note 4). Larger-scale pilot validation, where thermal losses are naturally minimised, will be essential to fully realise this symbiotic architecture.

### Dynamic self-regulation under intermittent renewable input

In membrane-electrolysis, the most critical risk is the cumulative mismatch between water production and consumption from intermittent renewable energy input. If production exceeds consumption (e.g., during power lulls), the continuous water influx causes electrolyte dilution, system overflow, and collapse of osmotic driving force, requiring additional energy-intensive separation processes. Conversely, excess consumption during peak power causes progressive dry-out. The continuous removal of water causes KOH concentration to spike, leading to localised corrosion, overheating and KOH crystallisation that blocks electrolyser flow channels. Managing this mismatch through frequent shutdown and restart cycles is highly destructive to hydrophobic membranes. Shutdown creates stagnation and high-salinity boundary layers at the membrane where salts quickly become supersaturated and crystallise, causing irreversible scaling. Frequent hydraulic shocks from pump restarts cause mechanical fatigue in the polymer matrix, lowering LEP and inducing pore wetting. Our OMD-AWE overcomes this mismatch through dynamic self-regulation, because the thermal driving force (waste heat) is physically phase-locked to the input power, the system naturally adjusts water supply in exact proportion to electrolyser demand, ensuring continuous synchronisation without destructive on/off control. Although sudden power changes create brief delays before the system temperature fully adjusts, the circulating KOH acts as a liquid reservoir that accommodates temporary fluctuations in both volume and concentration. Since renewable energy fluctuations are cyclical, these transient deviations naturally cancel out over time, preventing cumulative mismatches.

**Fig. 5:**
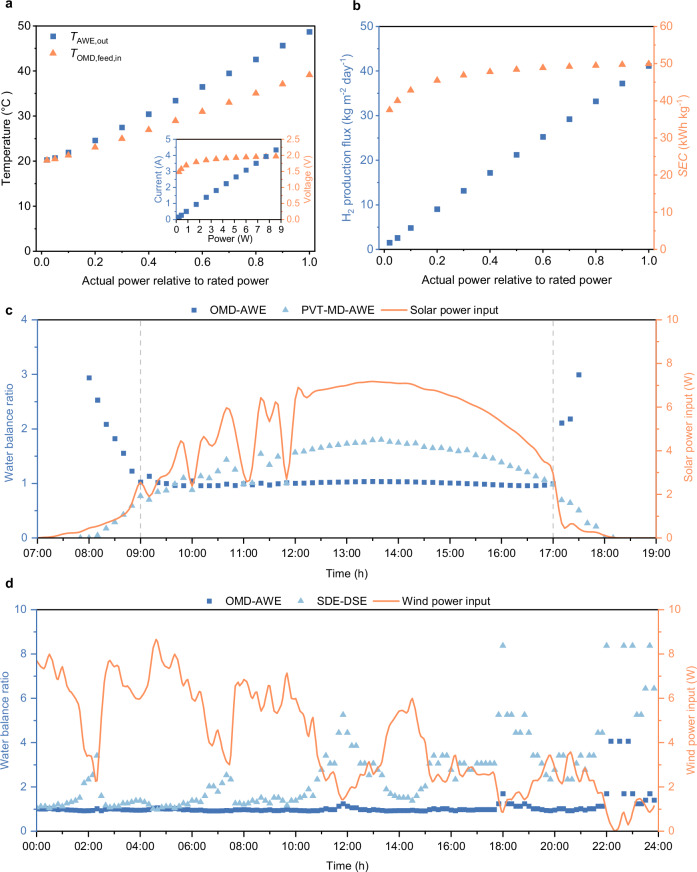
The dynamic response of the thermally symbiotic OMD-AWE under intermittent power input. **a** the changes in *T*_AWE,out_ and *T*_OMD,feed,in_ as a function of the AWE power, with inset showing current and voltage variations with input power; **b** the changes in H_2_ production flux and *SEC* as a function of the AWE power; **c** the comparison in water balance between OMD-AWE and PVT-MD-AWE under intermittent solar power input, with vertical dashed lines marking the start and end of the operational window; **d** the comparison in water balance between OMD-AWE and SDE-DSE under intermittent wind power input. Water balance ratios calculated at discrete time points from continuous power data. (The simulation considers a single-cell electrolyser operated at a current density of 0.5 A cm^−2^ with Ni nanowire-based cathode and NiFe-based anode separated by a ZIRFON diaphragm at an area of 7.5 cm^2^ integrated with a 1 cm^2^ PTFE membrane, with detailed system configurations and operating parameters shown in Supplementary Fig. 4).

To validate these, we applied the thermal-water-hydrogen model to simulate the system’s dynamic response using instantaneous power as the input variable (methods in Supplementary Note 5). As power input drops from the rated condition, the electrolyser current reduces proportionally, which lowers waste heat generation and naturally cools *T*_AWE,out_ and *T*_OMD,feed,in_ (Fig. 5a). Such temperature drop reduces Δ*T*_OMD_, scaling down water supply to match the reduced electrolysis demand. In addition, the system naturally transitions to a higher-efficiency mode during partial loads, as the reduction in ohmic voltage losses at lower current densities dominates over slight increases in kinetic overpotentials at lower AWE temperatures, resulting in a 25% decrease in *SEC* (Fig. 5b). We then simulated the system stability under realistic solar and wind profiles characterised by significant intermittency (Supplementary Fig. 5). The stability was indicated by a water balance ratio of water production rate to water consumption rate, both derived from fitting the power profile to the system with methods detailed in Supplementary Note 5. Under intermittent solar power (Fig. 5c), our OMD-AWE maintains a stable ratio of around 1.0 during 09:00–17:00, even during highly intermittent morning hours with cloud cover. This contrasts with traditional photovoltaic-thermal (PVT)-MD systems, which exhibit asynchronous responses throughout the day^60^. During mornings, low ambient temperatures enhance PV electrical efficiency, but insufficient thermal input limits water production. At midday, high temperatures maximise thermal input but reduce PV efficiency due to overheating. This mismatch creates a parabolic water balance curve with ratios varying from 0 to 1.8. Similarly, under erratic 24-h offshore wind profiles, the OMD-AWE also maintains near-perfect balance, contrasting with isothermal SDE-DSE whose water balance fluctuates wildly from 1 to over 10. (Fig. 5d). Over 24 h, SDE-DSE generates near 100% water surplus, causing catastrophic electrolyte dilution requiring energy-intensive separation to fix. The only exception to this dynamic self-regulation occurs during extreme power lulls (below 35% rated power). Under such power conditions, the electrolyser’s water consumption is minimised, but the osmotic driving force of 6 M KOH continues to extract water, causing accumulation if left unchecked. Suspending feed supply during these off-periods prevents excess accumulation and enables maintenance activities like membrane cleaning to prepare for resumed high-power. These findings confirm that the OMD-AWE provides a universally self-regulating architecture, maintaining operational stability under intermittent power inputs without requiring external thermal infrastructure or complex control systems. Beyond thermodynamic water balance, the decoupling of the membrane and electrolyser ensures that power fluctuations do not compromise the PTFE membrane’s intrinsic permeability or salt rejection. While intermittent operation poses known durability challenges to standard AWE catalysts^61^, these are general electrochemical issues rather than limitations of our water-energy coupling mechanism. The chemical inertness of the PTFE barrier ensures our architecture is fully compatible with standard battery buffering^62^ or emerging intermittency-resistant electrodes^63,64^, providing a comprehensive pathway for long-term electrochemical stability.

### Comparative techno-economic and environmental assessment

**Fig. 6:**
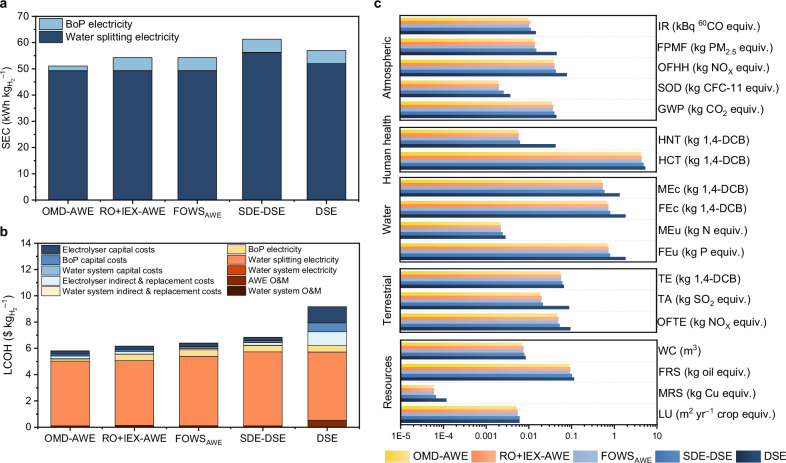
Techno-economic and environmental assessment of OMD-AWE. **a** The comparison in *SEC* between OMD-AWE and other seawater-to-hydrogen systems: RO + IEX-AWE, FOWS_AWE_, SDE-DSE, DSE; **b** Breakdown of LCOH from OMD-AWE compared against the other four competing systems; and **c** the life cycle environmental impacts (IR, ionising radiation; FPMF, fine particulate matter formation; OFHH, ozone formation–human health; SOD, stratospheric ozone depletion; SOD, stratospheric ozone depletion; GWP, global warming potential; HNT, human non-carcinogenic toxicity; HCT, human carcinogenic toxicity; MEc, marine ecotoxicity; FEc, freshwater ecotoxicity; MEu, marine eutrophication; FEu, freshwater eutrophication; TE, terrestrial ecotoxicity; TA, terrestrial acidification; OFTE, ozone formation–terrestrial ecosystems; WC, water consumption; FRS, fossil resource scarcity; MRS, mineral resource scarcity LU, land use).

The thermally symbiotic OMD-AWE offers a cost-competitive and sustainable pathway for seawater H_2_ production, supported by a comprehensive analysis of its energy consumption, techno-economic analysis (TEA), and life-cycle assessment (LCA) as shown in Fig. 6 (details see Supplementary Notes 6–7). The OMD-AWE was compared against four competing systems: i) conventional RO and ion exchange (IEX)-pretreated AWE (RO + IEX-AWE), ii) FOWS_AWE_^33^, iii) SDE-DSE^13^, and iv) direct electrolysis of untreated seawater (DSE)^65^. As shown in Fig. 6a, OMD-AWE achieves the lowest *SEC* of 51.05 kWh kg(H_2_)^–1^ due to the near-zero energy burden for water purification and the elimination of external active cooling systems from our thermally symbiotic design. The only electricity addition beyond water splitting is 1.75 kWh kg(H_2_)^–1^ from electricity balance-of-plant (BoP) for running pumps, making it even competitive with commercial pure-water electrolysis (51.1–66.7 kWh kg(H_2_)^–1^). Other four systems consume around 3 times more BoP electricity on cooling, while FOWS_AWE_ and SDE-DSE requires 7% and 14% more electricity for water splitting due to the FO membrane’s intolerance to KOH concentrations above 1 M and temperature constraints, respectively. Since energy represents the primary operational cost^66^, OMD-AWE is identified as the most cost-effective choice, revealed by TEA which uses the levelised cost of H_2_ (LCOH) as the objective function. At current electricity prices ($0.1 kWh^−1^), it shows the lowest LCOH of $5.73 kg(H_2_)^−1^, remaining 6.3%, 12.2%, 17.8%, and 52.8% lower than RO + IEX-AWE, FOWS_AWE_, SDE-DSE, and DSE, respectively (Fig. 6b). At more favourable electricity costs used in other studies ($0.02–0.04 kWh^−1^ ^67,68^, our LCOH drops to $1.65–2.67 kg(H_2_)^−1^. The cost difference compared to DSE highlights the critical importance of durability and system complexity. Traditional DSE suffers from prohibitively high LCOH due to expensive noble metal catalysts and shortened lifetimes. State-of-the-art kW-scale direct seawater electrolysers have shifted toward a catalyst-centric paradigm, engineering specialised materials to resist chloride attack, demonstrating stability for hundreds to thousands of hours^67,68^. Their industrial viability depends on surviving multi-year lifetimes (>10,000 h) under the fluctuating, harsh matrix of real seawater, while still requiring chemical pretreatment and energy-intensive cooling. Our OMD-AWE offers a complementary isolationist paradigm that isolates electrolyser from seawater, decoupling stability from catalyst design. This enables use of mature, low-cost AWE components with established industrial longevities (>80,000 h) while eliminating active cooling and pretreatment. More importantly, these catalyst-centric and isolationist paradigms are not mutually exclusive; future systems could profoundly benefit from their synergy, by pairing advanced catalysts with membrane pre-purification to further reduce contaminant burdens and maximise industrial lifespans.

The energy and cost efficiency of OMD-AWE yield comprehensive environmental benefits as demonstrated by LCA (Fig. 6c). When compared to the conventional RO + IEX-AWE and FOWS_AWE_, it generates 5–11% and 4–6% lower environmental impacts, respectively. The reductions are even greater when compared to SDE-DSE (10–37%), including the reductions in global warming potential (15%), marine eutrophication (18%), and stratospheric ozone depletion (37%), benefited from energy-efficient operating conditions and smaller membrane areas due to high water flux. The most substantial improvement is seen against DSE with impacts reduced by 17–660%, as our system avoids inherent inefficiency, operational complexity, and the environmental burden of noble metal catalysts.

### Implications and future perspectives

The development of the thermally symbiotic system carries broad implications for the future of green H_2_ production from seawater. By coupling OMD and AWE as perfect thermodynamic complements, this architecture shifts the paradigm of seawater splitting from complex catalyst engineering to thermodynamic system design, simultaneously solving the heat-dependency bottleneck of membrane distillation and overcoming the low H_2_ yield and catalyst durability limitations of direct seawater electrolysis. This convergence enables a high H_2_ production rate with stable operation demonstrated over 500 h, offering a cost-competitive and sustainable pathway. The complete isolation from seawater contaminants through chemically inert PTFE membranes enables broad compatibility with diverse electrode materials, from non-precious metal catalysts in AWE to those for other alkaline, neutral, or acidified electrolysis, allowing research focus on proven pure-water catalysts to accelerate industrial adoption and reduce costs. Our thermal-water-hydrogen model provides a flexible co-design framework that enables balanced system optimisation. The model could not only match OMD with AWE at any condition but also design optimal operational windows for thermal self-sustaining operation. This modular design avoids over-pressure and reverse flux issues and allows for independent optimisation of each unit, ensuring adaptable scaling. By buffering the fluctuations of renewable energy while co-producing surplus water and simplifying brine management, this system unlocks the potential for autonomous, decentralised, and offshore operations.

Beyond H_2_ production, this thermal symbiosis architecture establishes a universal thermodynamic framework for other water-energy nexus challenges. By coupling exothermic electrochemical processes, e.g., CO_2_ reduction, ammonia synthesis, and lithium extraction, with thermally-driven separations, this architecture repurposes parasitic Joule heating to drive MD-based distillation. In doing so, the system simultaneously provides essential passive cooling, recovers liquid chemical products, and extracts pure water to push residual brines toward zero-liquid discharge. This architecture shifts the traditional chemical engineering paradigm, transforming isolated engineering liabilities into self-sustaining resource mining and chemical manufacturing engines.

## Methods

### Chemicals

KOH (85.0–100.5%), Na_2_SO_4_ (≥99%), and EDTA ( ≥ 99%) were purchased from Scharlau. NaCl ( ≥ 99%), CaCl_2_ (≥97%), and SDBS (technical grade) were purchased from Sigma-Aldrich. SRNOM was purchased from the International Humic Substance Society (IHSS). Stock solutions were prepared by dissolving these chemicals in ultrapure water (>18.2 MΩ·cm) produced by a water purification system (Millipore, USA).

### Membrane module for evaluating water vapour and salt transport

The membrane module comprises a custom-made cross-flow membrane cell featuring two symmetric chambers for feed and permeate separated by a single flat membrane sheet with an effective contact area of 1 cm^2^ (1 × 1 cm). A thin-film composite (TFC) membrane (Porifera, 051303) was used for FO tests, while a PTFE membrane (its pore size distribution was analysed by ImageJ as shown in Supplementary Fig. 16) was used for MD/OD tests. For both feed and permeate sides, the respective feed and permeate solutions were recirculated from 1 L tanks through the cell by a peristaltic pump operated at a flowrate of 50 ml min^–1^. The transport of Cl^–^, SO_4_^2–^, Ca^2+^, Mg^2+^, and TOC across membranes was evaluated by the changes in their concentrations in the feed. Cl^–^ and SO_4_^2–^ concentrations were determined using ion chromatography (IC, 940 Professional IC Vario, Metrohm), Ca^2+^ and Mg^2+^ levels were measured by an Inductively Coupled Plasma-Optical Emission Spectrometer (ICP-OES, 725-ES, Varian), and TOC was determined using a total organic carbon analyser (TOC-L analyser, Shimadzu). The obtained concentrations measured from the feed tank were normalised by the loss of water and further used to calculate their rejection by the membrane using Eq. 12:

12 $${Rejection}\left(\%\right)=\left(1-\frac{{C}_{P}}{{C}_{f}}\right)\times 100$$

where *C*_*p*_ and *C*_*f*_ represent the concentration of each compound permeated through the membrane and its initial concentration, respectively.

### Measurement of liquid entry pressure

The membrane’s resistance to wetting was quantified by measuring LEP, which is defined as minimum pressure required to force liquid through the hydrophobic membrane pores. The LEP was measured using a custom-built apparatus, as shown in Supplementary Fig. 26. The system consisted of a high-pressure N_2_ cylinder equipped with a pressure regulator to slowly control gas feed, a sealed tube holding the PTFE membrane, and a pressure gauge to monitor the applied pressure. For each measurement, a dry membrane sample was mounted in the tube with its feed side filled with the test liquid while the permeate side opens to atmosphere. N_2_ gas was gradually released to the tube, increasing the applied pressure. The permeate surface was continuously monitored, and the LEP was recorded as the pressure at the exact moment the first liquid droplet was observed.

### Surfactant-accelerated wetting tests

Surfactant-accelerated wetting tests was conducted using 0.1 mM SDBS feed and 6 M KOH OA under the same operational conditions as previous sections. The wetting behaviour was specifically investigated at different feed temperatures of 40, 60, and 80  °C, while OA was maintained at 20  °C. The experiments at 40 and 60 °C comprised two consecutive cycles of up to 2 h each. Membrane performance was monitored through *J*_w_/*J*_w,0_, where *J*_w,0_ is the initial flux in the first cycle. A ratio at ~1.0 indicates stable operation and a sharp increase signals wetting^21^. Between cycles, the membrane was regenerated by a 30 min deionised water rinse followed by oven drying at 50  °C for 3 h^20^.

### Accelerated scaling tests

Gypsum scaling behaviour was investigated using a supersaturated gypsum solution as the feed and 6 M KOH as OA. The feed solution was prepared by mixing equal volumes (500 mL each) of 60 mM Na_2_SO_4_ and 60 mM CaCl_2_, resulting in a final solution containing 30 mM of each salt. The experiments were conducted using the previously described membrane setup and operational conditions. The scaling propensity was evaluated at different feed temperatures of 40 and 60  °C, while OA temperature was maintained at 20  °C. Scaling behaviour was monitored over three consecutive cycles of 7 h each by tracking *J*_w_/*J*_w,0_ where a decline signifies scale formation. Between each cycle, the membrane was regenerated via a 30-min cleaning with 0.05 M EDTA followed by 10-min deionised water rinsing^27^.

### Integrating OMD with AWE for continuous H_2_ production

The OMD and AWE unit were integrated as shown in Supplementary Fig. 27. During operation, clean water is extracted from the feed stream via OMD using PTFE membranes. The permeated clean water dilutes the circulating osmotic agent (KOH, 6 M), and is then electrolysed in the AWE stack, splitting it into hydrogen and oxygen. A gravity-based degas tower was used to separate H_2_ and O_2_ from the KOH streams^32^. The removal of water as gas reconcentrates the KOH solution back to its original 6 M concentration for recirculation.

The AWE electrolyser is a 5-cell alkaline stack (LBE-5SC, Light Bridge Inc.). Each cell contains series-connected bipolar nickel-based composite electrodes including a Ni nanowire-based cathode (4.4 cm diameter, JUNA TECH) and a NiFe-based anode (5.1 cm diameter, JUNA TECH). The two electrodes are separated by a 500 μm thick diaphragm (ZIRFON PERL UTP 500). A DC power supply (RXN-605D, Zhaoxin) maintained a constant current, while system stability was assessed by monitoring voltage. The feed temperature was maintained using a water bath (XMTD205, Jiangsu Kexi Instrument) with a temperature controller. The water flux across membranes (*J*_w_, LMH) was gravimetrically measured by the loss of water weight in the feed tank over time using an electronic balance (FX-3000GD, A&D Instruments) as represented by the Eq. 13.

13 $${J}_{W}=\frac{\Delta M}{\rho \cdot S \cdot \Delta t}$$

where Δ*M* is the water weight change during the test (g), and Δ*t* is the time interval of recording water weight. The hydrogen production rate (${r}_{{H}_{2}}$, L day^–1^) was determined by measuring the volume of water displaced from an inverted cylinder over specified time intervals. The hydrogen flux (kg m^–2^ day^–1^) was calculated by dividing the obtained H_2_ production rate by the membrane area. The purity of the collected H_2_ gas was determined using a gas chromatography (7890B GC System, Agilent) equipped with a thermal conductivity detector. The Faradaic efficiency (*FE*) of the AWE stack was determined by dividing the experimentally determined ${r}_{{H}_{2}}$ by the theoretical hydrogen production rate (${r}_{{H}_{2},{ideal}}$), as shown in Eq. 14.

14 $${FE}=\frac{{r}_{{H}_{2}}}{{r}_{{H}_{2},{ideal}}}=\frac{{r}_{{H}_{2}}}{\frac{{i}_{{cell}}}{2F}\times \frac{R \, \cdot \, T}{{P}_{0}}}$$

The OA conductivity was continuously monitored to track the KOH concentration. The long-term stability of OMD-AWE was assessed for 500 h using real seawater. Due to the recirculating nature of the laboratory-scale system, the seawater in the feed tank was replaced every 24 h to prevent compositional shift. In alignment with industrial membrane plant practices for scaling mitigation, EDTA was dosed into the feed solution at a concentration of 0.5% (w/v). The electrodes and membrane morphologies before and after the 500-h continuous operation were characterised by SEM (JSM-6390, JEOL), while their elemental composition changes were identified by EDX (JSM-6390, JEOL). Sample preparation involved gold coating via sputter deposition to ensure sufficient surface conductivity (K575xd, Emitech Ltd). Pore size distribution was analysed from SEM images using ImageJ software^69^. Membrane surface topography was further investigated by AFM (Dimension Nexus, Bruker). Bulk crystalline phases were identified by powder XRD (X’Pert Pro, Panalytical), while surface chemical composition was evaluated by XPS (Axis Supra + , Kratos Analytical Limited). The crystallinity of the PTFE membranes before and after the 500-h test was calculated using the Hermans-Weidinger method, as shown in Eq. 15^70^.

15 $${X}_{c}=\frac{{I}_{c}}{{I}_{c}+{1.8I}_{a}}\times 100\%$$

where *X*_c_ is the crystallinity of PTFE, *I*_c_ is the integral intensity of the crystalline area, and *I*_a_ is the integral intensity of the amorphous area. The *SEC* of the membrane-integrated AWE systems (kWh kg(H_2_)^–1^) was calculated by Eq. 16.

16 $${SEC}=\frac{{i}_{{cell}}U}{{r}_{{H}_{2}}}+10\frac{k{g}_{{H}_{2}O}}{{{kg}}_{{H}_{2}}}\times 0.833\frac{{kWh}}{k{g}_{{H}_{2}O}}+\frac{10\frac{k{g}_{{H}_{2}O}}{{{kg}}_{{H}_{2}}}}{{RR}}\times 0.023\frac{{kWh}}{k{g}_{{H}_{2}O}}$$

## Supplementary information

Supplementary Information

Transparent Peer Review file

## Source data

Source Data

## Data Availability

The data that supports the findings of the study are included in the main text and Supplementary Information files. Source data are provided with this paper.
