# Supplementary material for: Thermally symbiotic integration of osmotic membrane distillation and electrolysis for direct seawater hydrogen production
Source: Nat Commun. 2026 Jul 7;17:8388. doi: 10.1038/s41467-026-74854-8 (PMC13473115; doi:10.1038/s41467-026-74854-8)
Supplement: Supplementary file 1 [file 41467_2026_74854_MOESM1_ESM.pdf]

Supplementary Information

Thermally symbiotic integration of osmotic membrane distillation and electrolysis for direct seawater hydrogen production

Gabriela Scheibel Cassol[1], Chii Shang [1,2], Paul Westerhoff [3], and Yinghao Song*[1,4,5]

1. Department of Civil and Environmental Engineering, The Hong Kong University of Science and Technology, Clear Water Bay, Kowloon, Hong Kong SAR, China

2. Hong Kong Branch of Chinese National Engineering Research Center for Control & Treatment of Heavy Metal Pollution, The Hong Kong University of Science and Technology, Clear Water Bay, Kowloon, Hong Kong SAR, China

3. School of Sustainable Engineering and The Built Environment, Arizona State University, Tempe, AZ, 85287, United States of America

4. Department of Building and Real Estate, The Hong Kong Polytechnic University, Hung Hom, Kowloon, Hong Kong SAR, China

5. Department of Mechanical Engineering, The Hong Kong Polytechnic University, Hung Hom, Kowloon, Hong Kong SAR, China

* Correspondence: ysongat@connect.ust.hk (Y. Song)

Table of Contents

Supplementary Note 1 Operation of $FOWS_{AWE}$ with seawater as feed 4
Supplementary Fig. 1 Water and salt transport through TFC membranes 6
Supplementary Table 1 Surface elemental composition analysis of the thin-film composite (TFC) membranes. 7
Supplementary Note 2 Calculating the vapour pressure 8
Supplementary Fig. 2 The comparison of vapour pressure (P_v) 9
Supplementary Note 3 Heat exchanger design and effectiveness analysis................................ 10
Supplementary Fig. 3 The impact of heat exchangers. 13
Supplementary Note 4 Heat loss analysis and thermal insulation strategy for pipes and heat exchangers.. 14
Supplementary Note 5. Dynamic response simulation for intermittent renewable energy 17
Supplementary Fig. 4 The designed OMD-AWE configuration and operating parameters used for intermittent renewable energy input analysis. 17
Supplementary Fig. 5 Solar irradiance, wind speed, and ambient air temperature of Hong Kong on 4th February 2026 obtained from Hong Kong Observatory. 20
Supplementary Note 6. Techno-economic analysis 21
Supplementary Table 4 Capital cost (CAPEX) of different water purification technologies.... 22
Supplementary Table 5 Input parameters for different electrolysis systems. 25
Supplementary Note 7. Life cycle assessment.. 26
Supplementary Table 6. Life cycle inventory for the AWE. 27
Supplementary Table 7. Life cycle inventory for osmotic membrane distillation (OMD). 28
Supplementary Table 8. Life cycle inventory for reverse osmosis coupled with ion exchange resins (RO+IEX). 29
Supplementary Table 9. Life cycle inventory for forward osmosis (FO)................................. 30
Supplementary Table 10. Life cycle inventory for osmotic distillation (OD) system.............. 31
Supplementary Table 11. Life cycle inventory data for direct seawater electrolysis (DSE)..... 32
Supplementary Fig. 6 Accelerated scaling tests. 33
Supplementary Fig. 7 SEM images of the PTFE membrane during accelerated scaling tests. . 34
Supplementary Fig. 8 EDX spectrum of the PTFE membrane during accelerated scaling tests. 34
Supplementary Fig. 9 Normalised water flux during.. 35
Supplementary Fig. 10 SEM images of the PTFE membrane 36

Supplementary Fig. 12 Polarisation curve of our 5-cell AWE electrolyser 37
Supplementary Fig. 13 A packed-bed degassing tower used for H_2 and O_2 separation from KOH. 38
Supplementary Fig. 14 EDX spectrum of the PTFE membrane... 39
Supplementary Fig. 15 XPS spectra of the PTFE membrane. 40
Supplementary Fig. 16 Pore size distribution of the PTFE membrane..................................... 41
Supplementary Fig. 17 The KOH concentration during the 500-h continuous operation of OMD-AWE. 42
Supplementary Fig. 18 SEM images of the electrodes before and after 500-h operation. 43
Supplementary Fig. 19 EDX spectrum of the electrodes after the 500-h operation. 43
Supplementary Fig. 20 Heat flow profile. 44
Supplementary Fig. 21 The heat flow of key units in OMD-AWE with different AWE stack efficiency (γ). 45
Supplementary Fig. 22 Temperature profiles demonstrate heat harvest in the OMD-AWE system by using heat exchangers. 46
Supplementary Fig. 23 Salinity changes from feed seawater (standard 35 g kg^{-1}) to brine at designed water recovery rates. 47
Supplementary Fig. 24 A schematic showing how surplus heat powers additional membrane modules for creating valuable secondary water output.. 48
Supplementary Fig. 25 Surplus Water Index (SWI) as a function AWE stack efficiency (γ)... 49
Supplementary Fig. 26 The setup for measuring liquid entry pressure (LEP) of the PTFE membranes. 50
Supplementary Fig. 27 Schematic diagram of the membrane-integrated electrolysis system. . 51
Supplementary Table 12 Detailed composition of the real seawater sample used in this study. 52
Supplementary Table 13 Surface elemental composition analysis of the PTFE membrane during accelerated scaling tests. 53
Supplementary Table 14 Surface elemental composition analysis of the PTFE membrane during accelerated scaling tests in the presence of organic matter... 54
Supplementary Table 15 Surface elemental composition analysis of the PTFE membrane. 55
Supplementary Table 16 Surface elemental composition analysis of the electrodes. 56
Supplementary Table 17 Operating parameters for OMD-AWE thermal self-circulation........ 57
Supplementary references 58

Supplementary Note 1 Operation of $FOWS_{AWE}$ with seawater as feed

While $FOWS_{AWE}$ using thin film composite (TFC) membranes performs well when treating relatively diluted streams such as secondary wastewater[1], seawater's high salt content presents a challenge for its system stability. To address this, we examined the reverse salt flux of KOH and transport of chloride ions (Cl^-) across TFC membranes using a membrane cell with 1 M KOH as the osmotic agent and 0.5 M NaCl as the feed due to its predominance in seawater, and the results are shown in Supplementary Fig. 1a. The TFC membrane exhibited a high RSF of KOH, at averaging 173 g m^{-2} h^{-1} (GMH), while the feed Cl^- concentration decreased by 3% during the 120 min continuous operation. Although a 97% rejection rate of Cl^- was achieved, it means that this 3% of Cl^- crossed the membrane, contaminating the feed water quality beyond the acceptable limit of commercial AWE electrolyser (i.e., requiring Type II water, with resistivity >1 MΩ·cm)[2]. These results revealed the mechanism of water and salt transport through TFC membranes when treating seawater as demonstrated in Supplementary Fig. 1b, based on a well-established solution-diffusion theory[3]. The hydrophilic polyamide layer serves as the functional core of TFC membranes, allowing water to readily dissolve into their polyamide matrix. The dissolved water then diffuses from the feed side (where water has higher chemical potential due to lower solute concentration) to the osmotic side (where concentrated KOH lowers water's chemical potential). This natural movement along the chemical potential gradient generates the forward water flux. Meanwhile, ions such as Cl^-, K^+, and OH^- are mostly rejected due to their low solubility in polyamide, charge repulsion, and surrounding hydration shells that prevent diffusion[4]. Despite these barriers, large chemical potential gradients still drive measurable ion transport, i.e., 0.5 M Cl^- in feed (vs. no Cl^- in osmotic agent) generates Cl^- flux, and 1 M KOH in osmotic agent induces reverse flux of KOH towards the feed.

These undesirable salt transports across TFC membranes compromise $FOWS_{AWE}$ stability. Reversely transported KOH may protonate the membrane surface, creating a negative charge

that attracts cations such as calcium and magnesium, risking scaling and exacerbated concentration polarisation that impacts impurity rejection[5–8]. In addition, exposure to high pH would swell the polyamide layer, forming microscopic defects that further facilitate salt transport[9]. On the other hand, the crossed Cl^- would reach the anode of AWE where it is oxidised to chlorine ($2Cl^- \rightarrow Cl_2 + 2e^-$, standard potential = 1.36 V vs. SHE), competing with oxygen evolution reaction (OER)[10,11]. The generated chlorine corrodes electrode materials and degrades separators, reducing the electrolyser lifespan, and compromises H_2 production efficiency and purity since part of the applied current is used for Cl^- oxidation rather than water splitting[2,12,13]. Moreover, chlorine and its chemically related species like hypochlorous acids could also reversely transport across the TFC membrane because they are small, neutral, and have appreciable solubility in polyamide, degrading the membrane and progressively reducing impurity rejection. An additional 72 h continuous test integrating the TFC membrane with AWE confirmed the chlorine formation and its reverse transport, with detected chlorine at 4.2 μg L^{-1} in the feed. After the 72-h continuous operation, the TFC membrane's polyamide surface was changed from a pristine "leaf-like" to a post-use "rose-like" morphology (Supplementary Fig. 1c–d), as revealed by scanning electron microscopy (SEM). This surface change is because chlorine breaks the amide C-N bonds while incorporating oxygen to form carboxyl groups, as evidenced by a 115% increase in the surface O/N ratio identified by energy-dispersive X-ray spectroscopy (EDX) (Supplementary Table 1). These findings suggest that the $FOWS_{AWE}$ using TFC membranes which rely on solution-diffusion transport mechanism is unsuitable for processing seawater feedstocks presenting high salt content.

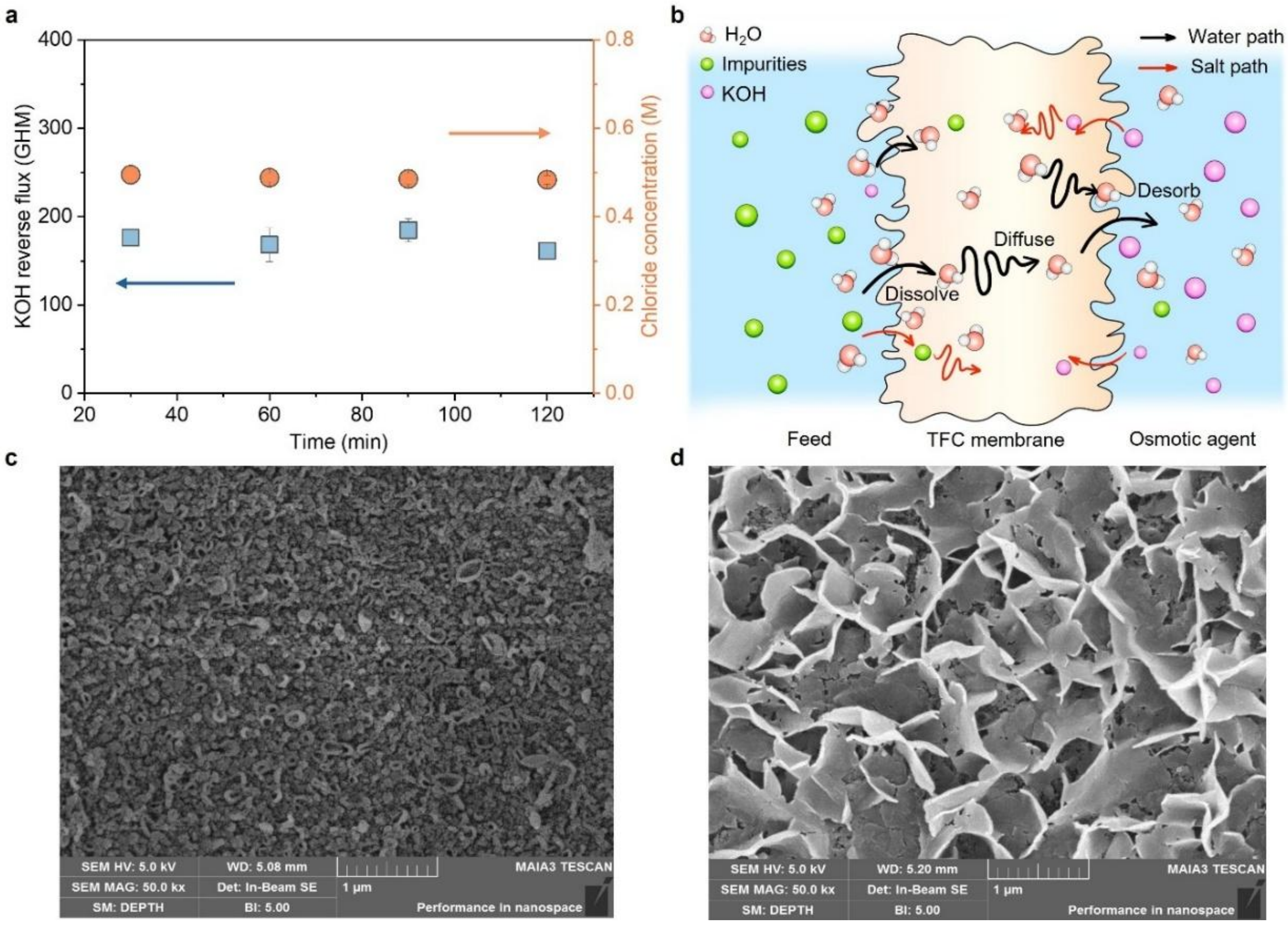

Supplementary Fig. 1 Water and salt transport through TFC membranes. a The changes in KOH reverse salt flux across TFC membranes and chloride ion concentration in feed during the 2-h testing period; **b** the water and salt transport mechanism through TFC membranes based on solution-diffusion theory; and **c–d** SEM images showing the polyamide surface morphology of the selective layer of the TFC membrane before and after 72-h continuous operation of $FOWS_{AWE}$ intergraded systems, respectively. (Conditions: Feed [NaCl] = 0.5 M, osmotic agent [KOH] = 1 M, $T_{feed} = T_{KOH}$ = 20°C, error bars represent standard deviation from two independent replicates.)

Supplementary Table 1 Surface elemental composition analysis of the thin-film composite (TFC) membranes. EDX showing surface elemental composition change of the selective layer between a pristine TFC selective layer and the TFC selective layer after 72 h of operation.

TFC membrane	Element	Atom (%)
Pristine	C	74.24
	N	13.35
	O	12.42
	O/N ratio	0.93
After 72 hours	C	64.76
	N	11.07
	O	22.14
	Impurities	2.03
	O/N ratio	2.00

Supplementary Note 2 Calculating the vapour pressure

The Clausius-Clapeyron relation establishes the relationship between temperature and the equilibrium between a liquid and its vapour phase. As the temperature increases, the vapour pressure (P_v) of the liquid rises exponentially[14,15]. This relation can be expressed by Equation S1:

$$ln\left(\frac{P_2}{P_1}\right) = \frac{\Delta H_{vap}}{R}\left(\frac{1}{T_1} - \frac{1}{T_2}\right) \quad (S1)$$

where P_1 and P_2 are the vapour pressures of pure water at the reference temperature T_1 (293 K) and at another temperature T_2 (e.g., 313 K), ΔH_{vap} is the enthalpy (heat) of vaporisation of water (40.7 kJ mol^{-1} at 373K and 1 atm), and R is the gas constant (8.3145 J mol^{-1} K^{-1}). To account for the effect of dissolved solutes on P_v, Raoult's law was applied. Raoult's law states that the P_v of a solution is equal to the P_v of the pure solvent at the same temperature multiplied by the mole fraction of the solvent present[14,16,17], as expressed in Equation S2:

$$P_{solution} = x_{solvent} P^0_{water} \quad \text{(S2)}$$

where $P_{solution}$ is the vapour pressure of the solution (kPa), $x_{solvent}$ is the mole fraction of the solvent (water), and P^o_{water} is the vapour pressure of pure water at a specified temperature. We employed Raoult's law to determine the change in P_v when transitioning from pure water to a solution of NaCl (0.5 M) for the feed solution, or KOH (6 M) for the draw solution. For instance, to calculate the P_v of the feed at 293 K, $x_{solvent}$ was multiplied by P_1, while to calculate the P_v of the feed at 313 K, $x_{solvent}$ was multiplied by the P_2 (calculated at 313 K using Equation S1). Supplementary Fig. 2 provides a schematic explanation of how the changes in P_v values are affected by variations in temperature and solute concentration, and how these changes impact the vapour pressure gradient (ΔP_v) across the membrane.

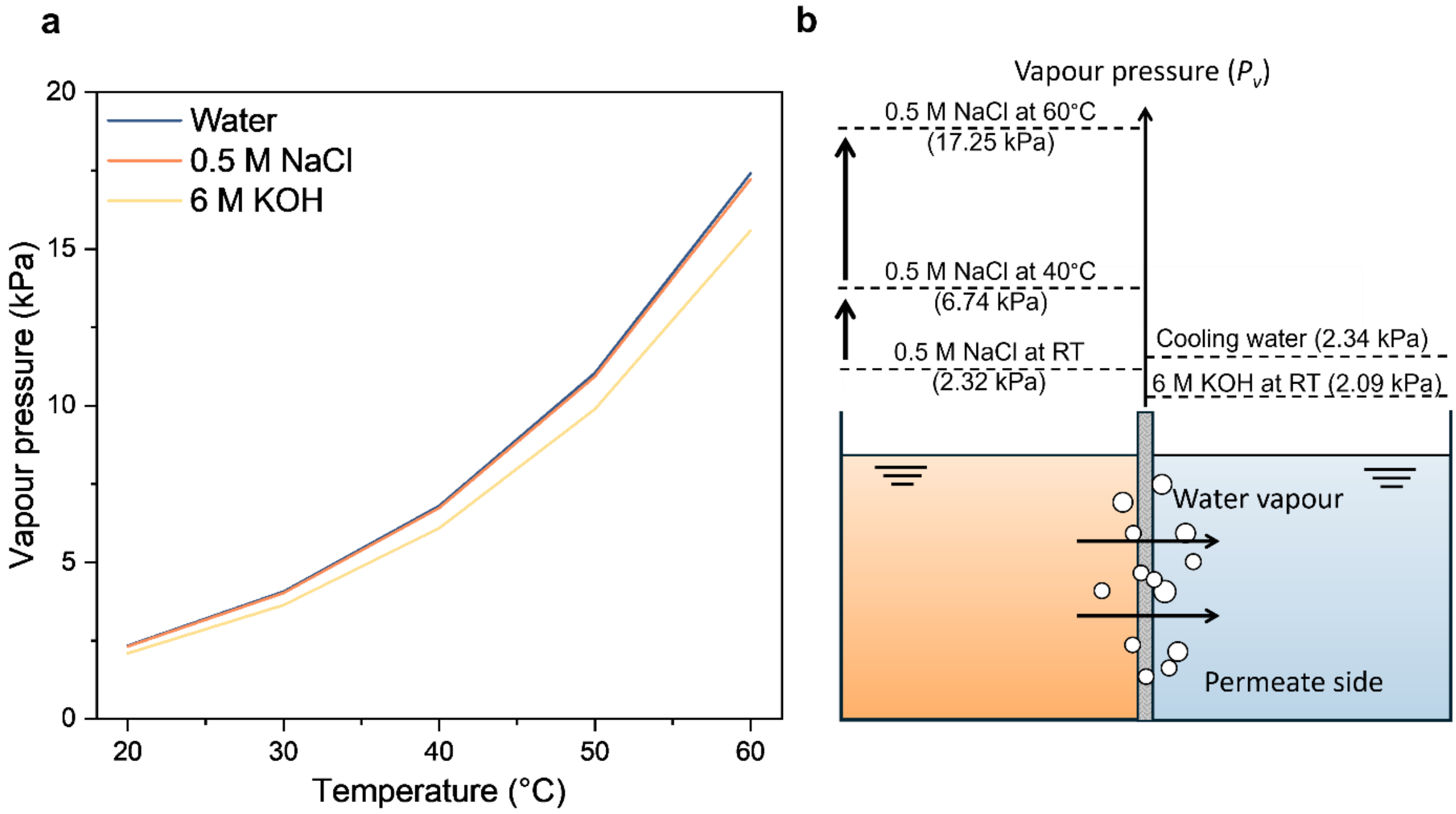

Supplementary Fig. 2 The comparison of vapour pressure (P_v) for **a** pure water, 0.5 M NaCl, and 6 M KOH at different temperatures; and **b** the schematic allowing us to calculate the vapour pressure gradient (ΔP_v) for i) isothermal osmotic distillation (OD) with feed at RT (20°C) and 6 M KOH osmotic agent at RT, ii) osmotic membrane distillation (OMD) with feed at 40°C and 60°C and 6M KOH osmotic agent at RT, and iii) direct contact membrane distillation (DCMD) with feed at 40°C and 60°C and cooling water at 20°C at the permeate side.

Supplementary Note 3 Heat exchanger design and effectiveness analysis

To accurately predict system performance and optimise thermal energy utilisation, we incorporated a heat exchanger design model based on the Number of Transfer Units (*NTU*) method to examine how heat exchanger effectiveness (ε) impacts system performance. The ε is the ratio of the actual heat transfer (Φ_{actual}) to the maximum thermodynamically possible heat transfer (Φ_{max}) which would occur if the heat exchanger had infinite surface area (as shown in Eq. S3)[18].

$$\varepsilon = \frac{\Phi_{actual}}{\Phi_{max}} = \frac{C_h(T_{h,in} - T_{h,out})}{C_{min}(T_{h,in} - T_{c,in})} = \frac{C_c(T_{c,out} - T_{c,in})}{C_{min}(T_{h,in} - T_{c,in})} \quad (S3)$$

where subscripts *h* and *c* represent hot and cold fluids, respectively, *T* is the temperature of fluids, *C* represents the heat capacity rate (W K^{-1}) obtained from the product of flowrate (q) and specific heat capacity (c), C_{min} denotes the smaller of the two heat capacity rates (i.e., $C_{min} = \min[C_h, C_c]$). The value of ε depends on the heat exchanger's flow arrangement, heat transfer area, and turbulence, with values typically ranging from 0.60 (shell-and-tube) to 0.95 (plate). ε is different from thermal efficiency (η) which refers to the ratio of heat output to heat input, which represents how well insulated the unit is from the environment. Based on this definition, the thermal balance equations for heat exchanger 1 (Hx,1) and 2 (Hx,2) are shown in Eqs. S4–5, respectively.

$$\Phi_{Hx,1} = \varepsilon \cdot c_{KOH} \frac{q_{OA}}{2}(T_{AWE,out} - T_{feed}) = c_{seawater} q_{feed}(T_{OMD,feed,in} - T_{feed})$$

$$= c_{KOH} \frac{q_{OA}}{2}(T_{AWE,out} - T_{cool,1}) \quad (S4)$$

$$\Phi_{Hx,2} = \varepsilon \cdot c_{KOH} \frac{q_{OA}}{2}(T_{AWE,out} - T_{OMD,OA,out}) = c_{KOH} q_{OA}(T_{AWE,in} - T_{OMD,OA,out})$$

$$= c_{KOH} \frac{q_{OA}}{2}(T_{AWE,out} - T_{cool,2}) \quad (S5)$$

To characterise the heat exchanger design, we used the *NTU* method, which represents the dimensionless thermal size of the exchanger and is calculated as a function of ε and heat capacity rate ratio (C_r) which is the ratio of the minimum to maximum heat capacity rates (Eq. S6). By applying a standard overall heat transfer coefficient (U) of 2,000 W m^{-2} K^{-1} typical for liquid-to-liquid system, we determined normalised heat transfer area (R_{hx}) which is the ratio of heat exchanger area (S_{hx}) required to achieve the target *NTU* to the membrane area (S) as shown in Eq. S7.

$$NTU = \frac{1}{C_r - 1}\ln\left(\frac{\varepsilon - 1}{\varepsilon C_r - 1}\right) = \frac{1}{\frac{C_{min}}{C_{max}} - 1}\ln\left(\frac{\varepsilon - 1}{\varepsilon\frac{C_{min}}{C_{max}} - 1}\right) \quad (S6)$$

$$R_{hx} = \frac{S_{hx}}{S} = \frac{NTU \cdot C_{min}}{U \cdot S} \quad (S7)$$

Incorporating these equations into our model, we could evaluate the impact of ε on the thermally symbiotic OMD-AWE considering ε ranging value from 0.6 to 0.95 and determine design parameters for heat exchangers (Supplementary Fig. 3). The simulation considers a single-cell electrolyser operated at a current density of 0.5 A cm^{-2} (using a Ni nanowire-based cathode and NiFe-based anode separated by a ZIRFON diaphragm, same as our experimental setup) integrated with 1-cm^2 membrane. We set the AWE outlet temperatures ($T_{AWE,out}$) at 50°C and the OMD feed temperature ($T_{OMD,feed,in}$) at 40°C. The results reveal that reducing ε from 0.95 to 0.60 leads to a 26% reduction in both water flux and H_2 production flux (Supplementary Fig. 3a), due to reduced heat recovery, which compromises the temperature gradient across the OMD unit. At lower effectiveness levels, AWE operates at a slightly lower temperature, resulting in a higher operating voltage that generates additional waste heat to compensate for the reduced thermal recovery (Supplementary Fig. 3b). Despite this voltage increase, the specific energy consumption (*SEC*) shows no significant increase. This is because the system output (water and hydrogen production) scales down with the thermal limits, ensuring that the energy input per

unit of product remains stable. Additionally, to maintain the target OMD feed temperature of 40°C under limited heat transfer (i.e., reduced ε), the system flowrates must be reduced (Supplementary Fig. 3c). While necessary for thermal balance, this reduction limits the latent heat transfer potential, contributing to lower water flux. For a heat exchanger design, the sizing analysis highlights a clear trade-off between performance and compactness (Supplementary Fig. 3d). An effectiveness of $\varepsilon = 0.60$ corresponds to a NTU of 1.1–1.6 and requires a heat exchanger area less than half the size of the membrane ($R_{hx} < 0.5$). This configuration is highly compact and easily achievable with low-cost shell-and-tube exchangers. Achieving $\varepsilon = 0.9$ requires an NTU of 3.4–4.6, corresponding to R_{hx} of 1.53–2.08 (i.e., 53–108% larger than the membrane area). Although this requires high-performance plate-type heat exchangers, the footprint remains comparable to the membrane, ensuring the system remains spatially viable. We also provided the inlet and outline temperature of the two heat exchangers as shown in Supplementary Table. 2. These results suggest that while high-effectiveness heat exchangers maximise H_2 yields, the system functions well even with lower-effectiveness alternatives. This flexibility allows optimisation based on space and cost constraints rather than requiring premium heat exchanger designs.

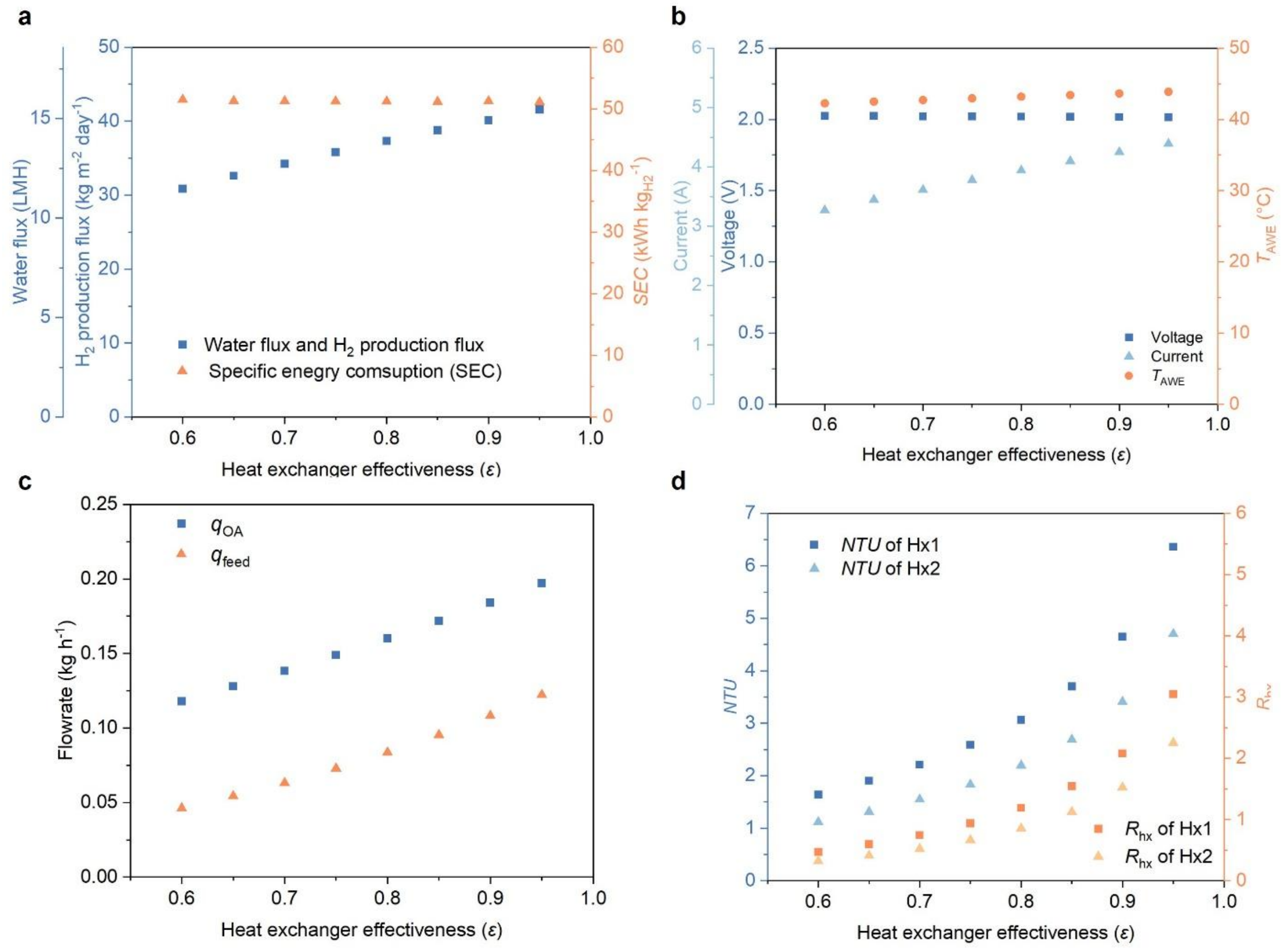

Supplementary Fig. 3 The impact of heat exchangers. a water flux, H_2 production flux, and *SEC* of the thermally symbiotic OMD-AWE as a function of ε; **b** the current, voltage, and operating temperature of AWE as a function of ε; **c** the flowrate of KOH osmotic agent (q_{OA}) and that of feed seawater (q_{feed}) as a function of ε; and **d** *NTU* and R_{hx} as a function of ε. (Conditions: The simulation considers a single-cell electrolyser operated at a current density of 0.5 A cm^{-2} using a Ni nanowire-based cathode and NiFe-based anode separated by a ZIRFON diaphragm)

Supplementary Table 2 The inlet and outline temperature of heat exchangers (Hx1 and Hx2).

ε	**Temperatures in Hx1 (°C)**			**Temperatures in Hx2 (°C)**				
	$T_{AWE,out}$	$T_{cool\text{-}1}$	$T_{OMD,feed,in}$	T_{feed}	$T_{AWE,out}$	$T_{cool\text{-}2}$	$T_{AWE,in}$	$T_{OMD,OA,out}$
0.95	50.0	21.5	40.0	20.0	50.0	27.8	37.7	26.7
0.9	50.0	23.0	40.0	20.0	50.0	29.2	37.3	26.9
0.85	50.0	24.5	40.0	20.0	50.0	30.5	36.8	27.1
0.8	50.0	26.0	40.0	20.0	50.0	31.8	36.4	27.3
0.75	50.0	27.5	40.0	20.0	50.0	33.1	35.9	27.4
0.7	50.0	29.0	40.0	20.0	50.0	34.3	35.4	27.6
0.65	50.0	30.5	40.0	20.0	50.0	35.5	35.0	27.7
0.6	50.0	32.0	40.0	20.0	50.0	36.7	34.5	27.8

Supplementary Note 4 Heat loss analysis and thermal insulation strategy for pipes and heat exchangers

In our thermally integrated systems, thermal management along the fluid transfer lines is a critical consideration. In typical laboratory-scale setups, significant heat loss is unavoidable due to the inherently high surface-area-to-volume ratio of small-diameter tubing and extremely low fluid flow rates. To quantify this effect and validate the feasibility of thermal self-circulation at a commercial scale, we developed a steady-state heat transfer model comparing our laboratory setup to a 1 MW industrial plant.

For our experimental setup, considering the AWE electrolyser works at a current density of 0.5 A cm^{-2} and cell voltage of 2.02 V (based on our polarisation curves) with outlet temperature ($T_{\mathrm{AWE,out}}$) at 50°C. To maintain self-circulation, the required flowrate for the KOH osmotic agent (OA) is 0.184 kg h^{-1}. We modelled the heat loss for a 0.5 m long transfer line for delivering the KOH using PharMed BPT Tubing (25#, inner diameter (d_{pipe}) = 4.8 mm and outer diameter (d_{pipe}')) = 8 mm). We consider thermal insulation by wrapping the pipe with flexible Aluminum-clad Expanded Polyethylene (EPE) foam at a thickness of 5 mm, showing a thermal conductivity (k_{ins}) of 0.038 W m^{-1} K^{-1}. Thus, the temperature drop (ΔT_{drop}) as the KOH transfers from the outlet of electrolyser to the inlet of heat exchangers could be determined by using Eqs. S8–9.

$$\Delta T_{drop} = \frac{T_{AWE,out} - T_{amb}}{R_{th} \cdot c_{KOH} \cdot q_{OA}} \quad (S8)$$

$$R_{th} = \frac{\ln\left(\frac{d_{ins}}{d_{pipe}}\right)}{2\pi \cdot k_{ins} \cdot L} \quad (S9)$$

where c_{KOH} is the specific heat capacity of KOH at 3500 J kg^{-1} K^{-1}, q_{OA} is its flowrate at 0.184 kg h^{-1}, T_{amb} is ambient temperature at 20°C, and R_{th} is the thermal resistance which is a function of the outer diameter of the insulation layer (d_{ins} =2×5 + 8 = 18 mm), the pipe's inner diameter

(d_{pipe}), k_{ins} and transferring distance (L, at ~0.5 m). Therefore, the calculated R_{th} is around 11 K W^{-1}, and thus ΔT_{drop} is 15°C (i.e., dropping from 50°C to 35°C) even with insulation applied. If $T_{AWE,out}$ is 100°C, the drop becomes 40°C. Thus, achieving thermal self-circulation at this lab-scale is thermodynamically impossible, since heat loss was too great to sustain the thermal loop.

However, the thermal insulation would work perfectly for an industry large scale as detailed in Supplementary Table 3. Considering scaling our 8.6 W lab unit (2.02 V and 4.25 A) up to a 1 MW industrial plant. The flowrate of KOH becomes 116,279 times (q_{OA} = 21,3950 kg h^{-1}). If maintaining a standard engineering practice for flow velocity at ~1.5 m s^{-1}, we can select a DN65 pipe (the inner and outer diameter is 62.7 and 73 mm) wrapped with Aluminum-clad polyurethane (PU) foam (k_{ins} = 0.024 W m^{-1} K^{-1}, which is less flexible but offers better thermal resistance than the EPE foam) at a thickness of 20 mm. The pipe is assumed to be 20 m long. By applying the model with these industrial parameters, the temperature of KOH in this scale only drops by 0.007°C, which is negligible. Even without thermal insulation by using only PVC pipes (k_{ins} = 0.2 W m^{-1} K^{-1}), the temperature drop is only 0.2°C. Our comparative analysis reveals that the observed temperature drop is specific to the lab setup. For real-world applications, standard insulation methods like Aluminum-clad PU foam are sufficient to maintain the thermal profile.

Similarly, to examine the thermal efficiency (η) of heat exchangers, we estimated the heat loss from heat exchangers for a 1 MW-scale OMD-AWE system by scaling up our 8.6 W lab unit (2.02 V, 4.25 A). Based on our AWE stack efficiency of 73.4%, the 1 MW system generates ~266 kW of waste heat. The required internal heat exchange areas for Hx1 and Hx2 are 24.3 m^2 and 17.8 m^2, respectively (for $\varepsilon = 0.9$). Assuming a standard plate heat exchanger packing density of 50 m^2 m^{-3}, the corresponding unit volumes are 0.49 m^3 and 0.36 m^3. We modelled these units with Length (L) × Width (W) × Height (H) of 1.0 × 0.5 × 1.0 m for Hx1 and L × W × H = 1.0 × 0.5 × 0.7 for Hx2, resulting in external surface areas of 4.0 m^2 and 3.1 m^2, respectively. We further assumed thermal insulation using Aluminum-clad polyurethane (PU) foam (k_{ins} = 0.024

W m^{-1} K^{-1}) with a thickness (d_{ins}) of 20 mm. The heat loss through heat exchangers ($\Phi_{loss,Hx}$) was calculated using Eq. S10:

$$\Phi_{loss,Hx} = \frac{k_{ins} \cdot S_{hx} \cdot \left(T_{AWE,out} - T_{amb}\right)}{d_{ins}} \quad (S10)$$

Assuming an ambient temperature (T_{amb}) of 20°C, the calculated $\Phi_{loss,Hx}$ for Hx1 and Hx2 are 144 W and 111 W, respectively. These losses represent <0.05% of the 266-kW waste heat, confirming that thermal efficiency approaches 100% at the industrial scale.

Supplementary Table 3 The comparison in thermal loss between lab and industrial setup.

Feature	Experimental setup (8 W AWE)	Industrial application (1 MW AWE)
Flowrate	0.184 kg h^{-1}	21,395 kg h^{-1}
Pipes with thermal insulation	25# PharMed BPT Tubing wrapped with 5 mm thick Aluminum-clad Expanded Polyethylene (EPE) foam, L = 0.5 m, k_{ins} = 0.038 W m^{-1} K^{-1}	DN65 PVC pipe wrapped with 20 mm thick Aluminum-clad polyurethane (PU) foam, L = 20 m, k_{ins} = 0.024 W m^{-1} K^{-1}
Thermal resistance	11 K W^{-1}	0.19 K W^{-1}
Temperature drop	15°C (drop from 50 to 35°C)<br>40°C (drop from 100 to 60°C)	0.007°C<br>(0.2°C if no thermal insulation)

Supplementary Note 5. Dynamic response simulation for intermittent renewable energy

1. Baseline system configuration

To evaluate the system's performance under intermittent renewable energy, we first established an optimal baseline configuration under steady-state conditions. We modelled a commercial single-cell AWE electrolyser (utilising a Ni nanowire-based cathode, a NiFe-based anode, and a ZIRFON diaphragm) operated at a current density of 0.5 A cm^{-2} integrated with 1-cm^2 membrane unit. The target engineering setpoints were fixed at an AWE outlet temperature ($T_{AWE,out}$) of 50°C and an OMD feed temperature ($T_{OMD,feed,in}$) of 40°C. Based on these setpoints, the model generated the optimal baseline parameters, including the AWE's rated power (8.56 W), current (4.25 A), and voltage (2.02 V), as well as the required stream flowrates and heat exchanger sizing (the Number of Transfer Units, NTU). These system configurations and operating parameters are summarised in Supplementary Fig. 4.

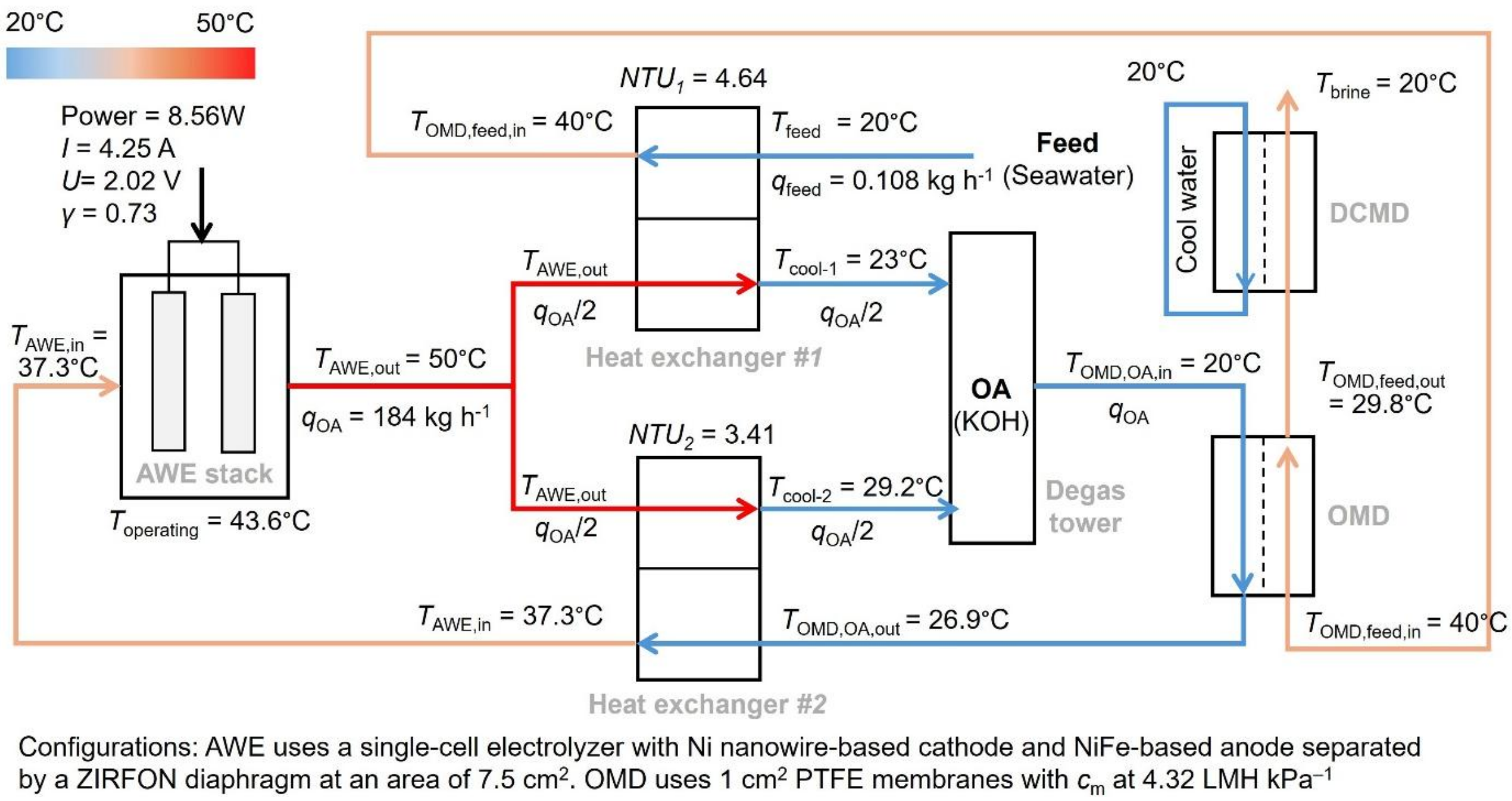

Supplementary Fig. 4 The designed OMD-AWE configuration and operating parameters used for intermittent renewable energy input analysis.

2. Establishing dynamic response as a function of power

We then transitioned the model from a static design framework to a dynamic simulation tool to account for intermittent renewable energy. In practical renewable energy systems, a Maximum Power Point Tracking (MPPT) DC-DC converter is usually used to optimise power transfer between the source (e.g., PV panel, wind turbine) and electrolyser. This MPPT converter dynamically optimises voltage and current levels to match the electrolyser's polarisation curve as input power fluctuates, ensuring maximum energy utilisation. To predict system performance under varying power (P), we established the relationship between the AWE's applied voltage (U, V), cell current (I_{cell}, A), and operating temperature (T_{op}, °C) which could be derived from fitting the varied power with empirical polarisation curves of electrolysis. Using our electrode materials with 7.5 cm^2 area as an example, U as a function of I_{cell} and T_{op} were established as shown in Eq. S11 which was further incorporated into the MPPT power fitting considering a typical conversion efficiency (η_{MPPT}) at 95% from the output power of source power (P) to that of AWE (P_{AWE}) (Eq. S12).

$$U = \left(0.00003{T_{op}}^2 - 0.0026T_{op} + 0.2248\right)\ln I_{cell} + \left(0.00003{T_{op}}^2 - 0.0062T_{op} + 1.9314\right) \quad (S11)$$

$$P \cdot \eta_{MPPT} = P_{AWE} = U \cdot I_{cell} \quad (S12)$$

These value of U and I_{cell} were then connected to the AWE's waste heat generation (Φ_{AWE}) as shown in Eq. S13).

$$\Phi_{AWE} = (U - U_{th})I_{cell}N_{cell} \quad (S13)$$

where U_{th} (1.48 V) represents the thermoneutral voltage for water electrolysis, N_{cell} is the number of electrolyser cell (at 1 for a single-cell electrolyser). By incorporating these equations into our model, we simulated dynamic thermal responses to varied power inputs. Using P as the input variable, the model calculates current/voltage relationships and stream temperatures while other

operating parameters (e.g., flowrate, *NTU*) remain constant. These results further determine system performance like water flux, H_2 production flux, and specific energy consumption (*SEC*).

3. Solar PV and Wind Power Integration

To examine if the system can maintain stable operation under realistic intermittent power input, we evaluated its dynamic water balance under realistic solar and wind power scenarios. We first integrated a standard PV module into the simulation, coupled by an MPPT algorithm. The simulation determines the PV cell temperature (T_{PV}, °C) as a function of ambient air temperature (T_{amb}, °C) and solar irradiance (G, W m^{-2}), using the nominal operating cell temperature of the PV cell (T_{NOCT}, °C) under T_{amb} of 20°C and G of 800 W m^{-2} (typically 48°C), as shown in Eq. S14.

$$T_{PV} = T_{amb} + \frac{G}{800}(T_{NOCT} - 20) \quad (S14)$$

This temperature is used to calculate its instantaneous efficiency (η_{PV}) by comparing T_{PV} to its standard 25°C value using Eq. S15, where η_{stc} is the rated PV efficiency at 25°C (considering 20%) and β is the temperature coefficient which defines how much power is lost per °C (typically at 0.35% °C^{-1}).

$$\eta_{PV} = \eta_{stc}[1 - \beta(T_{PV} - 25)] \quad (S15)$$

Finally, the actual power delivered to the AWE (P_{AWE}) is obtained from the incident solar energy adjusted for both η_{PV} and η_{MPPT} as shown in Eq. S16, where the PV area (S_{PV}) is 0.043 m^2 based on the AWE's 8.56 W rated power under standard conditions (1000 W m^{-2}) and η_{stc} of 20%.

$$P_{AWE} = P_{PV} \cdot \eta_{MPPT} = G \cdot S_{PV} \cdot \eta_{PV} \cdot \eta_{MPPT} \quad (S16)$$

Using actual data from Hong Kong Observatory on 4th Feb. 2026 (Supplementary Fig. 5), we generated a variable P_{AWE} profile over an operational day (7:00–19:00) characterised by significant intermittency between 9:30 and 12:00 due to cloud cover. The OMD-AWE's stability was indicated by a water balance ratio calculated by the water production rate through OMD

divided by the water consumption rates of AWE, both of which were obtained by fitting the P_{AWE} profile to our model. The water consumption rate is proportional to the applied current while water production rate is determined by vapor pressure gradient across the membrane. For comparison, a traditional photovoltaic thermal MD systems (PVT(MD)) was also simulated under the same weather conditions using the model from Ali, A. S. & Bounahmidi (2025)[19]. Similarly, wind power input was calculated using a proportional correlation with the wind speed profile and then applied to our model, with results compared to state-of-the-art SDE-DSE system[20].

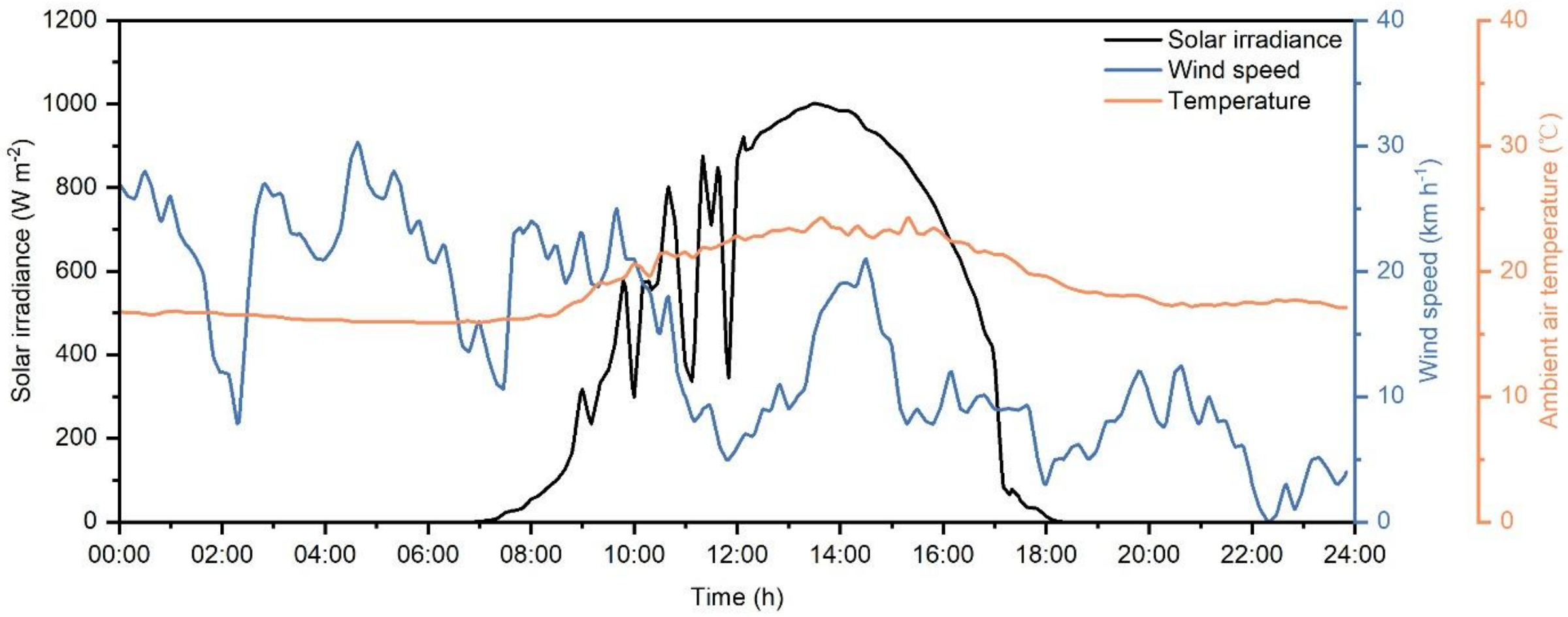

Supplementary Fig. 5 Solar irradiance, wind speed, and ambient air temperature of Hong Kong on 4th February 2026 obtained from Hong Kong Observatory.

Supplementary Note 6. Techno-economic analysis

The levelised cost of hydrogen (LCOH) serves as the objective function for this techno-economic analysis. To calculate the LCOH of the integrated systems, data for individual processes (e.g., seawater pretreatment, electrolysis) were combined and adjusted to account for system-specific interactions. The first scenario combined osmotic membrane desalination (OMD) with AWE, representing our proposed system (OMD-AWE). The second scenario employed a hybrid pretreatment system using reverse osmosis (RO) followed by ion exchange (IEX) prior to AWE, corresponding to conventional desalination practices (RO+IEX-AWE). The third scenario introduced the forward osmosis water splitting ($FOWS_{AWE}$) configuration[1]. For direct seawater electrolysis (DSE) alternatives, the fourth scenario examined a self-dampening electrolyte DSE (SDE-DSE) system[20], which corresponds to an osmotic distillation (OD, membrane distillation at room temperature) system combined with an AWE, while the fifth scenario assessed conventional DSE without pretreatment[21,22]. The LCOH was calculated as the ratio of total annualised system costs to annual hydrogen production, as shown in Eq. S17:

$$LCOH = \frac{TotalCost}{Q_{H_2}} \quad (S17)$$

where Q_{H_2} is the annual hydrogen production (kg year^{-1}), and $TotalCost$ represents the sum of annualised capital expenditures (CAPEX), operating expenses (OPEX), and replacement costs (REP) of both water producing and electrolysis systems. The total cost is derived as Eq. S18[23]:

$$TotalCost = AF \times CAPEX + OPEX + REP \quad (S18)$$

The annualisation factor (AF) converts upfront capital investments into equivalent annual payments, accounting for the discount rate (i = 6%) and project lifetime (n = 20 years), as shown in Eq. S19:

$$AF = \frac{i(1+i)^n}{(1+i)^n - 1} \quad (S19)$$

Capital Expenditures (CAPEX)

The total CAPEX comprises the water purification system (*waterSystem*) and electrolyser costs (*electrolyser*), as shown in Eq. S20:

$$CAPEX = waterSystem + electrolyzer \quad (S20)$$

All the costs are adjusted to 2024 values using the Chemical Engineering Plant Cost Index (CEPCI)[24], as shown in Eq. S21:

$$C_{I,curr} = C_{I.ref} \frac{CEPCI_{2023}}{CEPCI_{ref}} \quad (S21)$$

where $CEPCI_{ref}$ depends on the reference year (e.g., $CEPCI_{2017}$ is 567.5).

Water purification system

The capital expenditure for the water purification system is a function of the plant's capacity and the specific technology employed is calculated using Eq. (S22)[25]:

$$waterSystem = \dot{q}_{water} \times wUnitCost \quad (S22)$$

where $\dot{q}_{water}$ is the purified water production rate (10 kg per 1 kg of H_2), and $wUnitCost$ is the plant (OMD, RO, FO, or OD) size unit cost, which are presented in Supplementary Table 4.

Supplementary Table 4 Capital cost (CAPEX) of different water purification technologies.

**Water system**	**CAPEX (\$ per m^3 h^{-1})**
Osmotic membrane distillation [24]	4,304
Reverse osmosis + ion exchange unit [24,26]	4,515
Forward osmosis [27]	787
Osmotic distillation [24]	2,032

The OD system for the SDE-AWE combination requires a very large membrane area due to its significantly lower water flux. As it is a non-modular design, we assumed a unit cost of $40 per m^2 (40% of the modular OMD cost) to reflect custom installation.

Electrolyser System CAPEX

The electrolyser system CAPEX includes stack costs ($stackCost$), balance-of-plant (BoP, 55% of stack costs), and supplementary costs (SC, 20% of stack + BoP), calculated by Eq. S23[28]:

$$electrolyzer = stackCost + BoP + SC \quad (S23)$$

The stack costs were derived from a unit cost ($unitStackCost$), calculated using Eq. S24[28]:

$$stackCost = unitStackCost \times Power_{rated} \quad (S24)$$

where $Power_{rated}$ is the nameplate capacity of the electrolyser or the power consumption of the stack, which can be translated to the hydrogen production rate per year ($\dot{q}_{H_2}$, kg $year^{-1}$). For the DSE system, this cost was 1758 \$ kW^{-1}, approximately five times higher[13,29] than the 351.55 \$ kW^{-1} for the AWE system[28].

Operating Expenditures (OPEX)

Water system OPEX

In this study the thermal energy utilised of the OMD system is assumed to be readily available as and free of cost since the electrolyser needs to be cooled continuously during operation. Therefore, the electrical energy demand is dependent only on the energy consumption of the pumps, considering the flow rate of the feed channels and resulting pressure loss. Therefore, the specific energy consumption (SEC_{water}) of OMD was assumed to be 0.75 kWh m^{-3}. In the case of FO systems, a SEC_{water} of 0.1 kWh m^{-3} was assumed for pumping system since the secondary processes to separate the clean water and regenerate the draw solution are not required. RO systems consume 4.5 kWh per m^{-3} of water produced [30], while IEX incurs a variable cost (IEX)

of 2.64 \$ per m^{-3} of water produced[23]. The insurance costs (IN) for the water systems are calculated to be 0.5% while service, while the operation and maintenance costs ($O\&M_{water}$) are estimated at 2% of the total capital investment costs for all water treatment systems. Therefore, the OPEX of the OMD, FO, OD, and RO+IEX systems are calculated as Eq. S25 and S26, respectively:

$$OPEX_{OMD/FO/OD} = \dot{q}_{Water}SEC_{water}p_{el} + IN + O\&M_{water} \ (S25)$$

$$OPEX_{RO+IEX} = \dot{q}_{Water}SEC_{water}p_{el} + IEX + IN + O\&M_{water} \ (S26)$$

where p_{el} is the unit price for electricity and $\dot{q}_{Water}$ is the yearly distillate capacity based on the stoichiometric requirement of 10 kg of water per kg of hydrogen.

Electrolyser OPEX

The electrolysers OPEX a sum of the stack specific energy consumption (SEC_{stack}) and the balance of plant electrical usage (SEC_{BoP}) to produce 1 kg of hydrogen, as shown in Eq. S27:

$$OPEX_{electrolyzer} = \dot{q}_{H_2} \times SEC_{stack} + \dot{q}_{H_2} \times SEC_{BoP} + IN + O\&M_{electrolyzer} \ (S27)$$

where $\dot{q}_{H_2}$ is the hydrogen production rate per year and $O\&M_{electrolyzer}$ is the fixed operations and maintenance factor, established at 2.0% of the total electrolyser capital investment, while IN is calculated to be 0.5%. Approximately 65% of the BoP consumption is attributed to cooling processes (primarily chillers)[31]. Since the OMD+AWE system eliminates the need for active cooling, this portion of the energy consumption can be excluded. The SEC_{stack} and the SEC_{BoP} for each process is detailed in Supplementary Table 5.

Supplementary Table 5 Input parameters for different electrolysis systems.

Parameter	OMD+AWE	RO+IEX+ AWE	$FOWS_{AWE}$	SDE-DSE	DSE
SEC_{stack} (kWh kg$(H_2)^{-1}$)	49.30	49.30	52.75	56.30	52.00
SEC_{BoP} (kWh kg$(H_2)^{-1}$)	1.75	5.00	5.00	5.00	5.00

The SEC_{stack} in the OMD+AWE and RO+AWE systems corresponds to operation at 80% efficiency (based on HHV). The SEC_{stack} of the $FOWS_{AWE}$ suffers a penalty for operating with the non-ideal KOH concentration of 1 M, resulting in a 7% increase in electricity consumption. The stack's operating voltage in the SDE-DSE system exceeds the average AWE consumption by around 14%, indicating lower efficiency[20,32]. This is attributed to reaction condition limitations (e.g., temperature constraints) imposed by the integrated membrane design, resulting in a higher SEC_{stack} of 56.3 kWh kg$(H_2)^{-1}$. The SEC of the DSE is an average of recent results[21,33]. Operations and maintenance costs of the electrolyser systems are approximated as 2% of their total capital costs[23].

Replacement Costs

The IEX resin replacement cost is embedded in the per-m^3 OPEX. The OMD and the FO membrane systems require replacement every 5 years, while RO membranes are replaced every 7 years. All membrane replacements are valued at 60% of the original CAPEX. The electrolyser stack undergoes replacement every 10 years at 15% of its initial CAPEX[34]. The present value of these costs is calculated using a 6% discount rate, as Eq. S28:

$$REP = \sum_{n=1}^{num\_replacements} \frac{C_{repl}}{(1+r)^{n \times lifetime}} \quad (S28)$$

Supplementary Note 7. Life cycle assessment

A Life Cycle Assessment (LCA) was conducted to evaluate the environmental impacts of hydrogen production systems, with all modelling and impact quantification performed using SimaPro software 9.5.0.1. The study defined its functional unit as 100 kg of hydrogen produced, which corresponds to 1000 kg (1 m^3) of water input based on the stoichiometric requirement of 10 kg of water per kg of hydrogen. The system boundary followed a cradle-to-gate approach, encompassing processes from raw material extraction to hydrogen generation. Five hydrogen production scenarios were analysed, each integrating alkaline water electrolysis (AWE) with different water supply systems for indirect seawater electrolysis or evaluating a direct seawater electrolysis (DSE) approach. To model these integrated systems, life cycle inventory (LCI) data for individual processes (e.g., seawater pretreatment, electrolysis) were combined and adjusted to account for system-specific interactions. The first scenario combined osmotic membrane desalination (OMD)[35,36] with AWE[28], representing our proposed system (OMD-AWE). The second scenario employed a hybrid pretreatment system using reverse osmosis (RO)[37,38] followed by ion exchange (IEX)[39] prior to AWE, corresponding to conventional desalination practices (RO+IEX-AWE). The third scenario introduced the forward osmosis water splitting ($FOWS_{AWE}$[1]) configuration, adapting RO-based LCI data to simulate FO performance. The fourth scenario examined a self-dampening electrolyte DSE (SDE-DSE) system[20] incorporating modified OMD parameters together with the AWE LCI, while the fifth scenario assessed conventional DSE without pretreatment using an AWE-derived LCI baseline. The LCI data were obtained from primary process measurements and adjusted when necessary to reflect integrated process interactions, with full inventory and justifications for all adjustments provided in Supplementary Tables 6–11. For environmental impact assessment, the ReCiPe 2016 Midpoint (H) methodology was employed to analyse key impact categories.

Supplementary Table 6. Life cycle inventory for the AWE. The data for AWE with a functional unit of 100 kg of hydrogen production[28].

Stage	Equipment/processes	Materials/energy
AWE stack	Copper [GLO]	1.93E-02 kg
	Steel, unalloyed [GLO]	1.93E+00 kg
	Nickel, 99.5% [GLO]	1.83E-01 kg
	Aluminium [GLO]	4.34E-03 kg
	Polyvinylchloride [RoW]	7.52E-03 kg
	Tetrafluoroethylene [GLO]	7.52E-04 kg
	Acrylonitrile-butadiene-styrene copolymer [GLO]	1.54E-03 kg
	Polyphenylene sulfide [GLO]	3.28E-03 kg
	Polysulfone [GLO]	2.51E-03 kg
	N-methyl-2-pyrrolidone [GLO]	1.25E-02 kg
	Aniline [RoW]	4.72E-04 kg
	Acetic anhydride [GLO]	5.20E-04 kg
	Purified terephthalic acid [GLO]	8.48E-04 kg
	Nitric acid, without water, in 50% solution state [RoW]	3.18E-04 kg
	Hydrochloric acid, without water, in 30% solution state [RoW]	1.25E-03 kg
	Graphite [GLO]	4.14E-03 kg
	Lubricating oil [GLO]	4.63E-06 kg
	Zirconium oxide [GLO]	1.06E-02 kg
	Carbon monoxide [RoW]	1.45E-03 kg
	Industrial machine heavy, unspecified [RoW]	1.54E-06 kg
	Plaster mixing [GLO]	7.52E-03 kg
Operation	Electricity, high voltage, solar [GLO] - Electrolysis	*
	Nitrogen, liquid [RoW]	2.90E-02 kg
	Potassium hydroxide [GLO]	1.90 kg
	Electricity, high voltage, solar [GLO] - BoP	**

*The electrolysis electricity consumption was 49.25 kWh per kg H_2 for AWE systems (OMD, RO+IEX, FO) and 56.30 kWh per kg H_2 for SDE-AWE.

** The BoP electricity consumption was 5 kWh per kg H_2 for all systems except OMD-AWE (1.7 kWh per kg H_2 due to reduced cooling requirements).

Supplementary Table 7. Life cycle inventory for osmotic membrane distillation (OMD). The inventory data used for the OMD system with a functional unit of 1000 kg of water production.

Stage	Equipment/processes	Materials/energy
Module	Non-woven polypropylene (MD membrane) [GLO]	0.126 kg/m^2 membrane
	Polyethylene (spacer) [GLO]	0.809 kg/m^2 membrane
	Polypropylene (housing) [GLO]	0.268 kg/ m^2 membrane
	Steel (housing) [GLO]	1.533 kg/m^2 membrane
	Injection mounding [GLO]	1.56 kg/m^2 membrane
Recuperator	Steel, high alloyed [GLO]	7 kg/kWth *
OMD plant construction	Gravel, crushed [GLO]	1.40E-05 kg
	Concrete [GLO]	3.70E-02 kg
	Sand [GLO]	3.30E-03 kg
	Anhydrite rock [GLO]	2.50E-02 kg
	Brick [GLO]	5.50E-05 kg
	Bitumen adhesive compound [GLO]	6.60E-05 kg
	Steel, unalloyed [GLO]	1.45E-03 kg
	Steel, low alloyed [GLO]	1.45E-03 kg
	Steel, high alloyed [GLO]	5.90E-05 kg
	Polyvinylchloride [GLO]	7.00E-06 kg
	Glass fiber reinforced plastic [GLO]	2.30E-04 kg
	Polypropylene [GLO]	7.00E-06 kg
	Polyethylene [GLO]	3.30E-04 kg
	Excavation, hydraulic digger [GLO]	2.50E-04 kg
	Extrusion, plastic pipes [GLO]	8.04E-02 kg
	Extrusion, plastic film [GLO]	3.44E-02 kg
	Hot rolling, steel [GLO]	1.45E-03 kg
	Sodium hydroxide [GLO]	1.02E-02 kg
	Sodium hypochlorite [GLO]	1.45E-04 kg
	Tetrafluoroethylene [GLO]	2.51E-04**
	Electricity [GLO]	0.75 kWh

* Two heat exchangers were considered based on our proposed system.
** Replaced every 5 years.

Supplementary Table 8. Life cycle inventory for reverse osmosis coupled with ion exchange resins (RO+IEX). The data used for the RO+IEX with a functional unit of 1000 kg of water production.

Stage	Equipment/processes	Materials/energy
Reverse osmosis construction	Soil excavation [GLO]	2.50E−04 kg
	Gravel, crushed [GLO]	1.40E-02 kg
	Concrete [GLO]	3.70E-02 kg
	Sand [GLO]	3.30E-03 kg
	Anhydrite rock [RoW]	2.50E-02 kg
	Brick [GLO]	5.50E−05 kg
	Bitumen	6.60E−05 kg
	Steel, low-alloyed [GLO]	2.90E-03 kg
	Stainless steel [GLO]	5.90E−05 kg
	Polyvinylchloride pipes [GLO]	6.60E−06 kg
	Glass fibre reinforced pipes [GLO]	2.30E−04 kg
	Synthetic rubber [GLO]	1.80E−05 kg
	Polypropylene [GLO]	7.10E−06 kg
	Polyethylene pipe and liner [GLO]	3.30E−04 kg
Reverse osmosis operation	Polyamide membranes, Nylon [GLO]	4.60E−05 kg *
	Polysulphone membrane [GLO]	2.75E-05 kg *
	Antiscaling	4.50E-03 kg
	Polypropylene [GLO]	1.69E-04 kg
	Coated steel [GLO]	8.91E-04 kg
	Sodium hypochlorite [RoW]	7.06E-03 kg
	Sodium hydrogen sulfite [GLO]	1.41E-02 kg
	Sulphuric acid [RoW]	5.88E-02 kg
	Electricity	4.50 kWh
IEX device production	Steel, low-alloyed [GLO]	1.36E-03 kg
	Cationic resin [RoW]	5.39E-04 kg
	Anionic resin [RoW]	5.99E-04 kg
	Steel, low-alloyed [GLO]	5.71E-05 kg
	Silica sand [GLO]	1.43E-03 kg
IEX operation	Hydrochloric acid, 30% [RoW]	1.80E-01 kg
	Sodium hydroxide, 50% [GLO]	1.11E-01 kg
	Electricity	0.10 kWh

* Replaced every 7 years.

Supplementary Table 9. Life cycle inventory for forward osmosis (FO). The data used for the FO system (within $FOWS_{AWE}$ system) with a functional unit of 1000 kg of water production (modified from RO).

Stage	Equipment/processes	Materials/energy
Forward osmosis construction	Soil excavation [GLO]	2.50E−04 kg
	Gravel, crushed [GLO]	1.40E-02 kg
	Concrete [GLO]	3.70E-02 kg
	Sand [GLO]	3.30E-03 kg
	Anhydrite rock [RoW]	2.50E-02 kg
	Brick [GLO]	5.50E−05 kg
	Bitumen	6.60E−05 kg
	Steel, low-alloyed [GLO]	2.90E-03 kg
	Stainless steel [GLO]	2.95E-05 kg*
	Polyvinylchloride pipes [GLO]	6.60E−06 kg
	Glass fibre reinforced pipes [GLO]	2.30E−04 kg
	Synthetic rubber [GLO]	1.80E−05 kg
	Polypropylene [GLO]	0**
	Polyethylene pipe and liner [GLO]	0**
Forward osmosis operation	Polyamide membranes, Nylon [GLO]	9.30E−05
	Polysulphone membrane [GLO]	5.50E-05
	Antiscaling	4.50E-03
	Polypropylene [GLO]	1.69E-04
	Coated steel [GLO]	8.91E-04
	Sodium hypochlorite [RoW]	7.06E-03
	Sodium hydrogen sulfite [GLO]	1.41E-02
	Sulphuric acid [RoW]	5.88E-02
	Electricity	0.1***

* Reduced by 50% compared to RO as the FO system does not require high pressure pumping.
** No water storage is needed as the FO system is directly coupled with AWE, sending water straight to electrolysis.
*** Electrical consumption is limited to pumping power.

Supplementary Table 10. Life cycle inventory for osmotic distillation (OD) system. The data used for the OD system (within SDE-AWE system) with a functional unit of 1000 kg of water production (modified from OMD).

Stage	Equipment/processes	Materials/energy
Module	Non-woven polypropylene (MD membrane) [GLO]	0*
	Polyethylene (spacer) [GLO]	0*
	Polypropylene (housing) [GLO]	0*
	Steel (housing) [GLO]	0*
	Injection mounding [GLO]	0*
Recuperator	Steel, high alloyed (for DCMD) [GLO]	0 **
OMD plant construction*	Gravel, crushed [GLO]	1.40E-05 kg
	Concrete [GLO]	3.70E-02 kg
	Sand [GLO]	3.30E-03 kg
	Anhydrite rock [GLO]	2.50E-02 kg
	Brick [GLO]	5.50E-05 kg
	Bitumen adhesive compound [GLO]	6.60E-05 kg
	Steel, unalloyed [GLO]	1.45E-03 kg
	Steel, low alloyed [GLO]	1.45E-03 kg
	Steel, high alloyed [GLO]	5.90E-05 kg
	Polyvinylchloride [GLO]	7.00E-06 kg
	Glass fiber reinforced plastic [GLO]	2.30E-04 kg
	Polypropylene [GLO]	7.00E-06 kg
	Polyethylene [GLO]	3.30E-04 kg
	Excavation, hydraulic digger [GLO]	2.50E-04 kg
	Extrusion, plastic pipes [GLO]	8.04E-02 kg
	Extrusion, plastic film [GLO]	3.44E-02 kg
	Hot rolling, steel [GLO]	1.45E-03 kg
	Sodium hydroxide [GLO]	1.02E-02 kg
	Sodium hypochlorite [GLO]	1.45E-04 kg
	Tetrafluoroethylene [GLO]	6.1E-03**
	Electricity [GLO]	0.75 kWh

* The membrane is directly integrated into the AWE system, eliminating the need for additional housing/spacers.
** The SDE-AWE system does not require heat exchangers as it operates at ambient temperature.
*** Assuming onshore deployment (simplifies infrastructure vs. offshore).

Supplementary Table 11. Life cycle inventory data for direct seawater electrolysis (DSE). The data used for a DSE system with a functional unit of 100 kg of hydrogen production (modified from AWE inventory).

Stage	Equipment/processes	Materials/energy
AWE stack	Copper [GLO]	1.93E-02 kg
	Steel, chromium steel [GLO]	1.93E+00 kg
	Nickel, 99.5% [GLO]	1.83E-01 kg
	Aluminium [GLO]	4.34E-03 kg
	Polyvinylchloride [RoW]	7.52E-03 kg
	Tetrafluoroethylene [GLO]	7.52E-04 kg
	Acrylonitrile-butadiene-styrene copolymer [GLO]	1.54E-03 kg
	Polyphenylene sulfide [GLO]	3.28E-03 kg
	Polysulfone [GLO]	2.51E-03 kg
	N-methyl-2-pyrrolidone [GLO]	1.25E-02 kg
	Aniline [RoW]	4.72E-04 kg
	Acetic anhydride [GLO]	5.20E-04 kg
	Purified terephthalic acid [GLO]	8.48E-04 kg
	Nitric acid, without water, in 50% solution state [RoW]	3.18E-04 kg
	Hydrochloric acid, without water, in 30% solution state [RoW]	1.25E-03 kg
	Graphite [GLO]	4.14E-03 kg
	Lubricating oil [GLO]	4.63E-06 kg
	Zirconium oxide [GLO]	1.06E-02 kg
	Carbon monoxide [RoW]	1.45E-03 kg
	Industrial machine heavy, unspecified [RoW]	1.54E-06 kg
	Plaster mixing [GLO]	7.52E-03 kg
	Nickel-Iron alloy (NiFe) [GLO]	1.83E-01 kg*
	Molybdenum [GLO]	1.83E-01 kg**
	Platinum (Pt) [GLO]	2.56E-4 kg**
Operation	Electricity, high voltage, solar [GLO] - Electrolysis	52.5 kWh/kg
	Nitrogen, liquid [RoW]	2.90E-02 kg
	Potassium hydroxide [GLO]	1.90 kg
	Sodium hydroxide, without water [GLO]	0.51***
	Electricity, high voltage, solar [GLO] - BoP	5.0 kWh/kg

* Corrosion-resistant anode (NiFe) for seawater.
** HER catalyst materials.
*** Theoretical NaOH consumption for Mg^{2+}/Ca^{2+} pre-precipitation adjusted for 90% recovery efficiency.

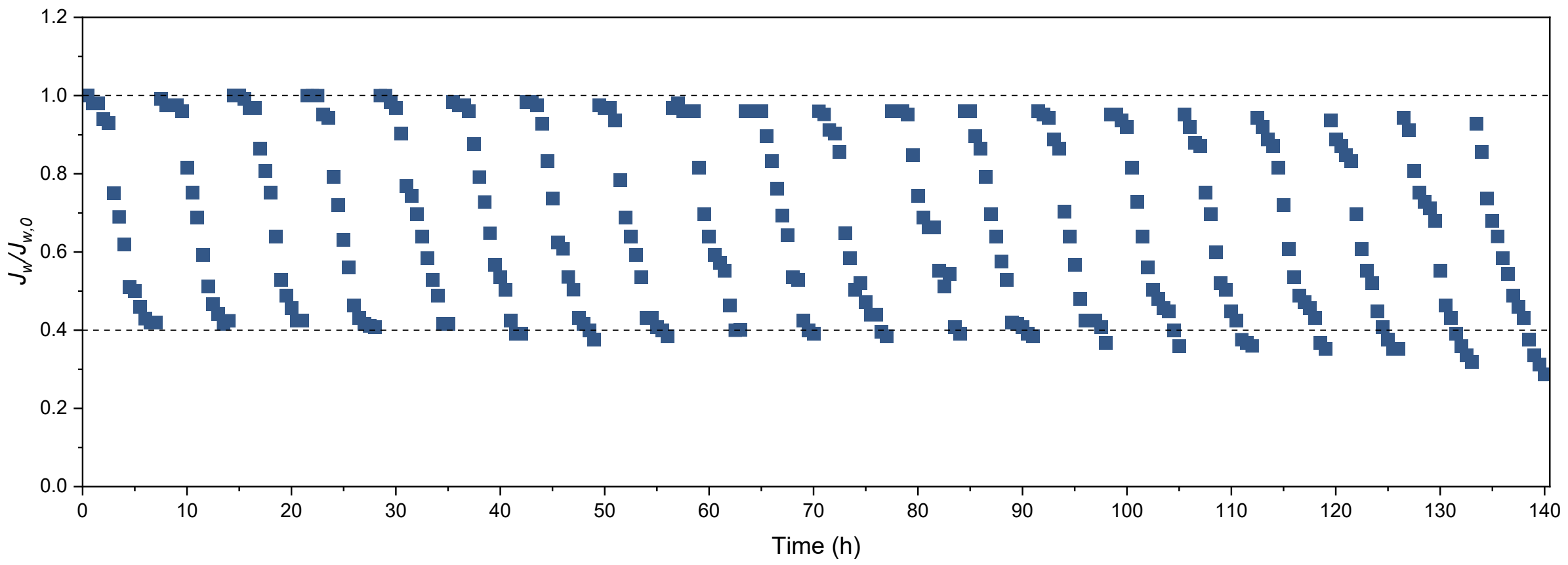

Supplementary Fig. 6 Accelerated scaling tests. $J_w/J_{w,0}$ over 20 cycles a feed containing 30 mM Na_2SO_4 and 30 mM $CaCl_2$ and 6 M KOH as osmotic agent. Each cycle involved 7 h of scaling followed by membrane cleaning through a 30-min flush with 0.05 M EDTA and a 10-min rinse with deionised water. Thermal conditions: T_{feed} = 40°C, T_{KOH} = 20°C.

Over the 20-cycle scaling test, the PTFE membrane demonstrated minimal performance decline with exceptional regenerative capacity. The first 6 cycles showed complete flux recovery (i.e., the normalised water flux ratio ($J_w/J_{w,0}$) consistently recovering to 1.0 after cleaning), while even after 20 cycles under such harsh accelerated conditions, the membrane still maintained >95% of $J_w/J_{w,0}$. SEM and EDX analysis confirmed the effectiveness of the EDTA-based cleaning. Large gypsum crystals were deposited on the membrane surface after 20 cycles but were removed after cleaning (Supplementary Fig. 7). EDX analysis showed an 84% reduction in the surface atomic concentrations of calcium (Ca), sulphur (S), and oxygen (O) which are key elemental markers of gypsum (Supplementary Fig. 8 and Table 13). The gradual reduction in flux recovery over the 20 cycles might be attributed to the residual 14% of these mineral phases. This behaviour is consistent with the PTFE membrane's small pore size, which provides excellent initial scaling resistance but may gradually retain microscopic crystal nuclei within the pore structures that are less accessible to cleaning. For future industrial deployment, this minor pore-entrapped scaling can be effectively mitigated through standard continuous antiscalant dosing or by heated EDTA cleaning. Maintaining >95% flux recovery after 20 accelerated cycles demonstrates robust

resistance to irreversible fouling and confirms the membrane's suitability for long-term operation under normal scaling environments.

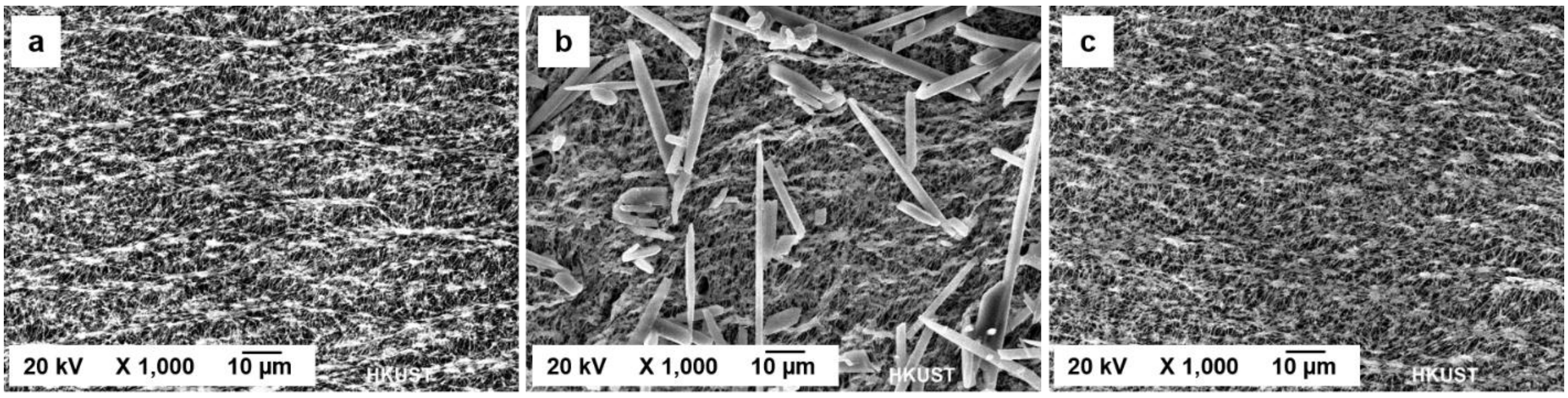

Supplementary Fig. 7 SEM images of the PTFE membrane during accelerated scaling tests. **a** the pristine PTFE membrane; **b** the PTFE membrane after 20 cycles without cleaning; and **c** the cleaned PTFE membrane after 20 cycles. Conditions: [feed] = 30 mM Na_2SO_4 and 30 mM $CaCl_2$, [KOH] = 6.0 M, T_{feed} = 40°C, T_{KOH} = 20°C.

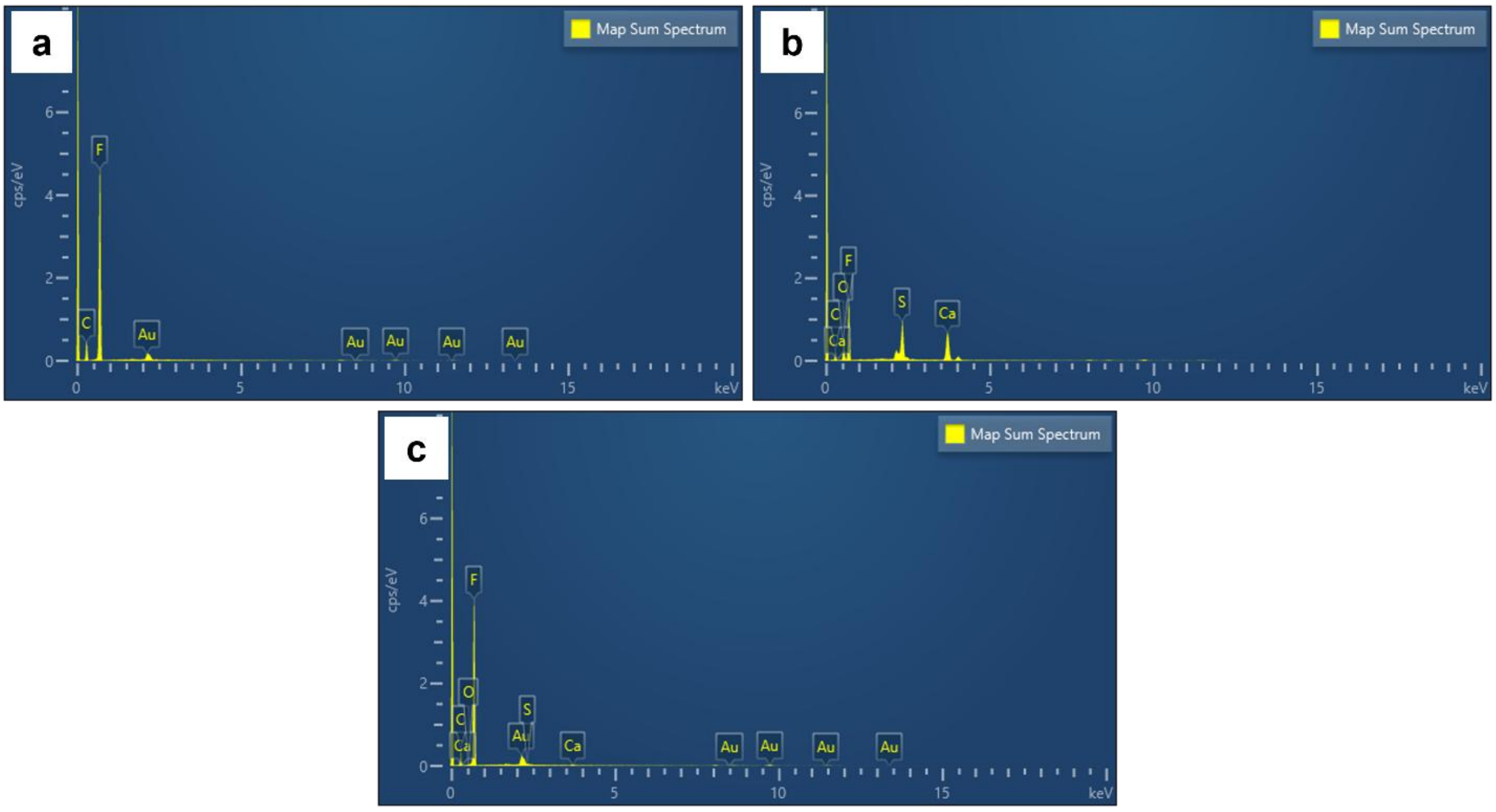

Supplementary Fig. 8 EDX spectrum of the PTFE membrane during accelerated scaling tests. Surface elemental composition of **a** the pristine PTFE membrane; **b** the PTFE membrane after 20 cycles without cleaning; and **c** the cleaned PTFE membrane after 20 cycles. Conditions: [feed] = 30 mM Na_2SO_4 and 30 mM $CaCl_2$, [KOH] = 6.0 M, T_{feed} = 40°C, T_{KOH} = 20°C.

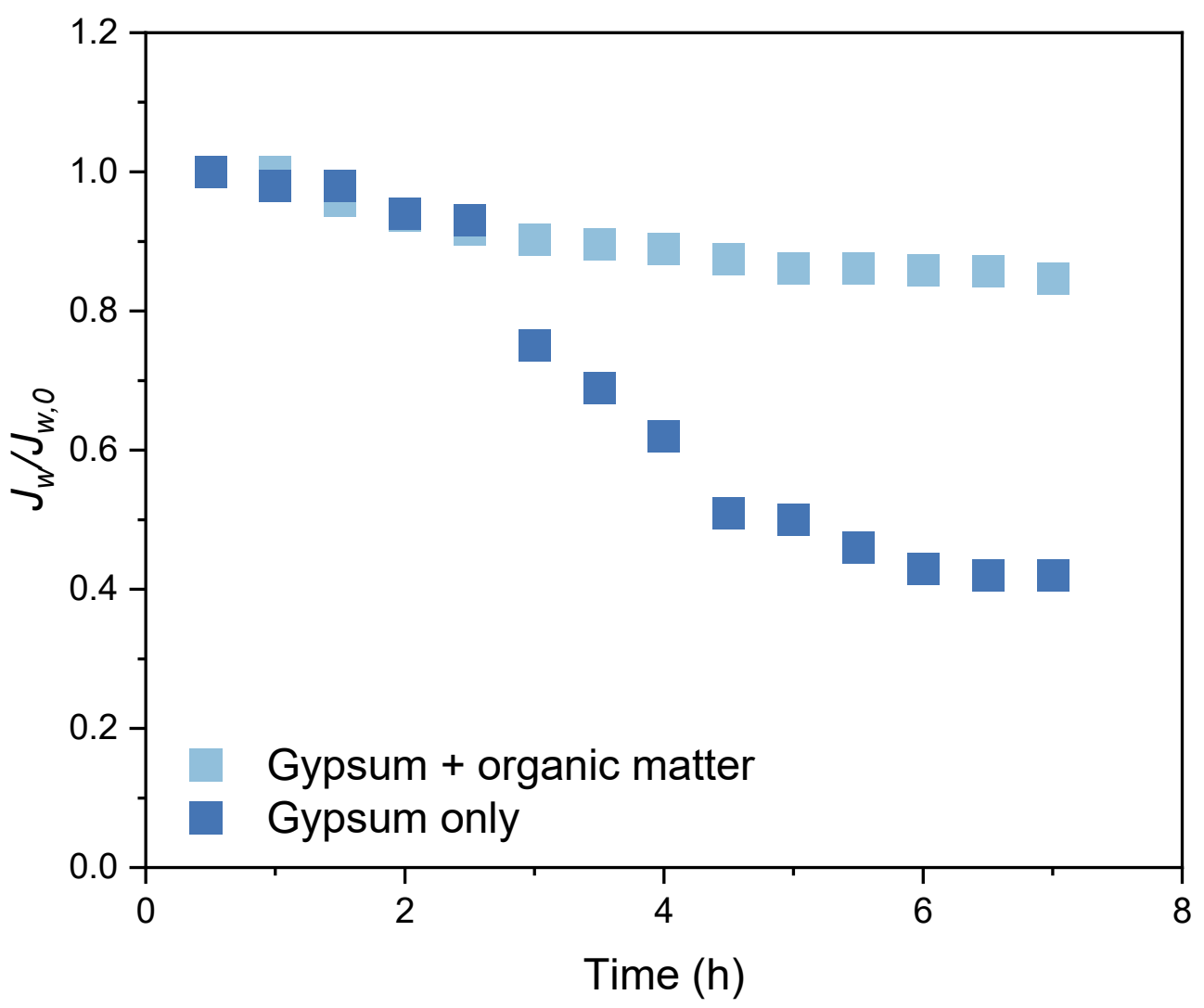

Supplementary Fig. 9 Normalised water flux during accelerated scaling tests in the presence and absence of organic matter. Conditions: [feed] = 30 mM Na_2SO_4 and 30 mM $CaCl_2$, [SRNOM] = 10 mg L^{-1} or 0 mg L^{-1}, [KOH] = 6.0 M, T_{feed} = 40°C, T_{KOH} = 20°C.

After 7 hours of operation, the normalised water flux ($J_w/J_{w,0}$) was reduced to only 0.84 for the gypsum +SRNOM feed, doubled the value for the gypsum only. Such substantial difference suggests that organic matters interfered with gypsum crystallisation. NOM can adsorb to active crystal growth sites and increases the energy barrier for nucleation. thereby reducing the risk of pore wetting[40]. The SEM and EDX analyses provide direct evidence for this organic-inorganic interaction. In the presence of SRNOM, gypsum crystals lost their sharp, invasive needle-like structures and instead formed rounded, composite aggregates that are far less likely to penetrate and wet the membrane pores (Supplementary Fig. 10). EDX analysis revealed a dramatic 30-fold reduction in surface-bound inorganic scale, with the atomic concentrations of gypsum-associated elements (Ca, S, O) decreased from 34.23% to 1.17%, when SRNOM was present (Supplementary Fig. 11 and Table 14). In addition, the F/C atomic ratio, which serves as an indicator of the membrane surface coverage by carbonaceous deposits, decreased from the pristine value of 1.75 to 1.42 in the gypsum-only test and to 1.39 in the test with SRNOM. The slightly lower F/C ratio in the presence of SRNOM reflects the additional organic carbon contributed by the NOM itself, suggesting the formation of a mixed organic-inorganic layer

where SRNOM is incorporated into the scaling matrix. Despite similar F/C ratios, the nature of fouling differed significantly. Gypsum-only testing showed extensive inorganic coverage (34.23%), while the gypsum + SRNOM testing yielded minimal inorganic deposition (1.17%) with organic matter as the dominant foulant.

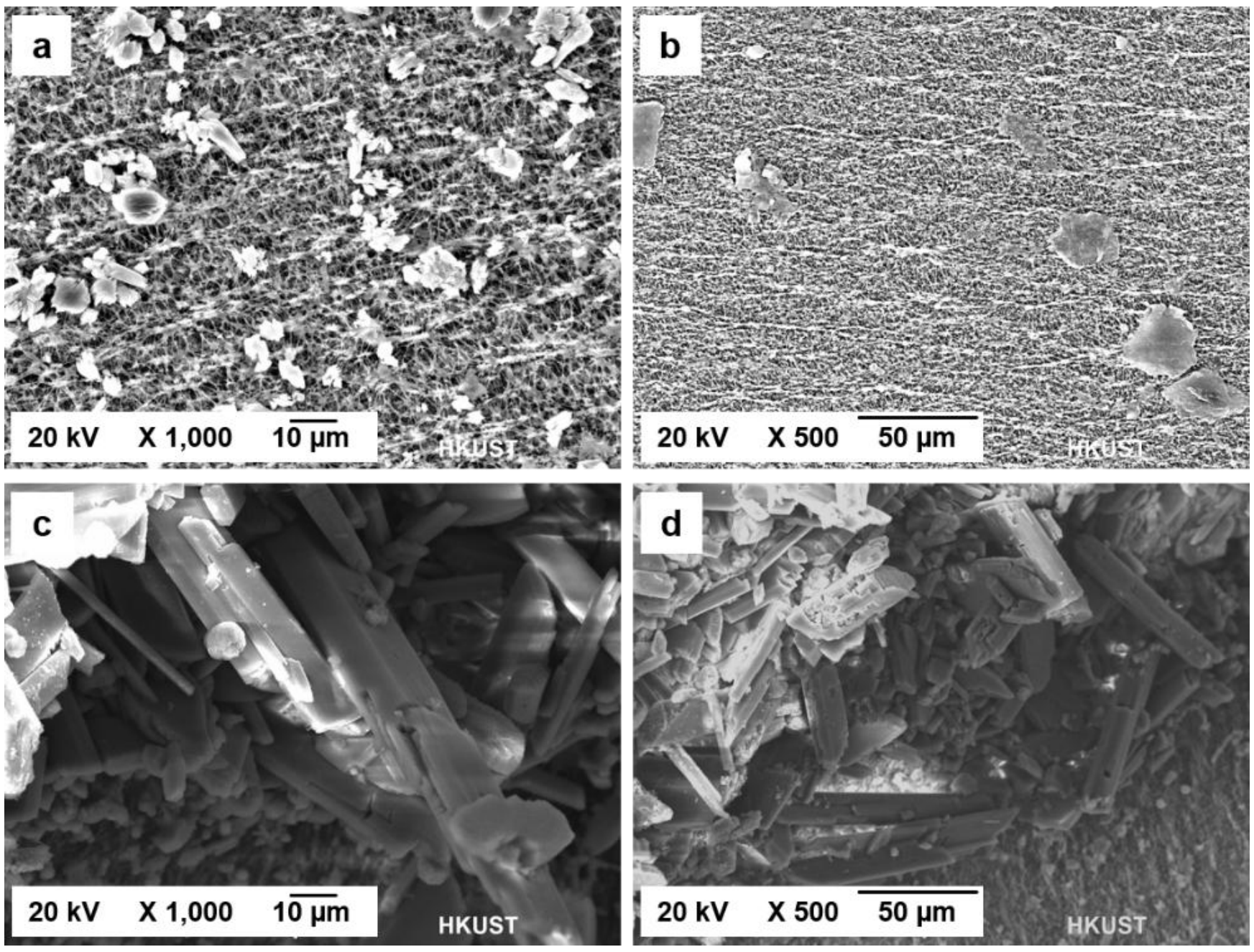

Supplementary Fig. 10 SEM images of the PTFE membrane following accelerated scaling tests in the presence and absence of organic matter. a–b Gypsum + SRNOM; **c–d** gypsum-only. Conditions: The gypsum solution contains 30 mM Na_2SO_4 and 30 mM $CaCl_2$, while the concentration of SRNOM is 10 mg L^{-1}. T_{feed} = 40°C, T_{KOH} = 20°C.

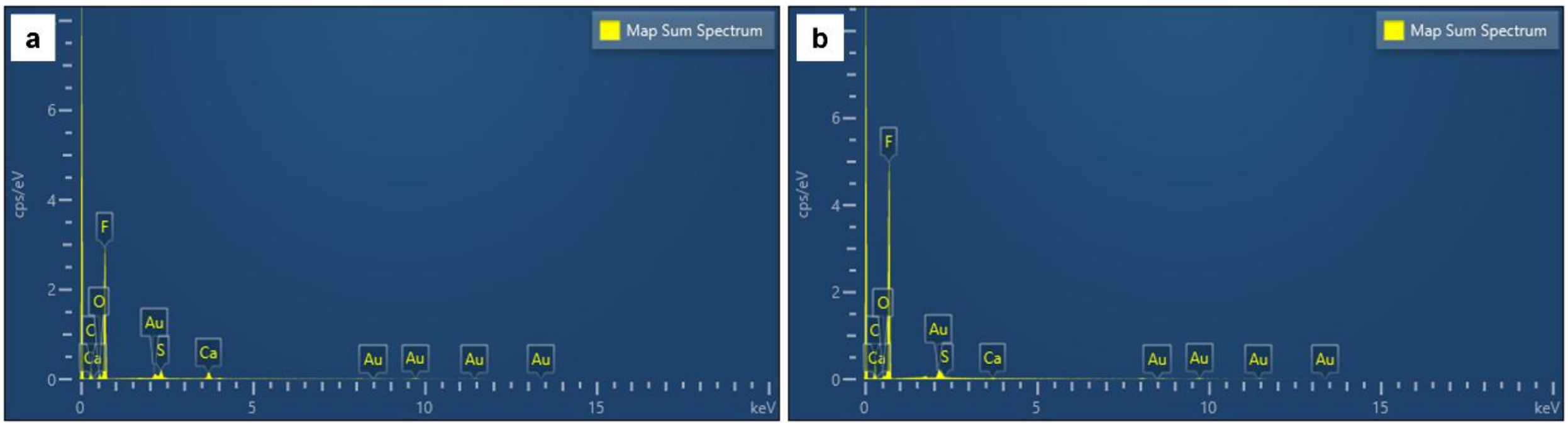

Supplementary Fig. 11 EDX spectrum of the PTFE membrane following accelerated scaling tests in the presence and absence of organic matter. a–b Gypsum + SRNOM; **c–d** gypsum-only. Conditions: The gypsum solution contains 30 mM Na_2SO_4 and 30 mM $CaCl_2$, while the concentration of SRNOM is 10 mg L^{-1}. T_{feed} = 40°C, T_{KOH} = 20°C.

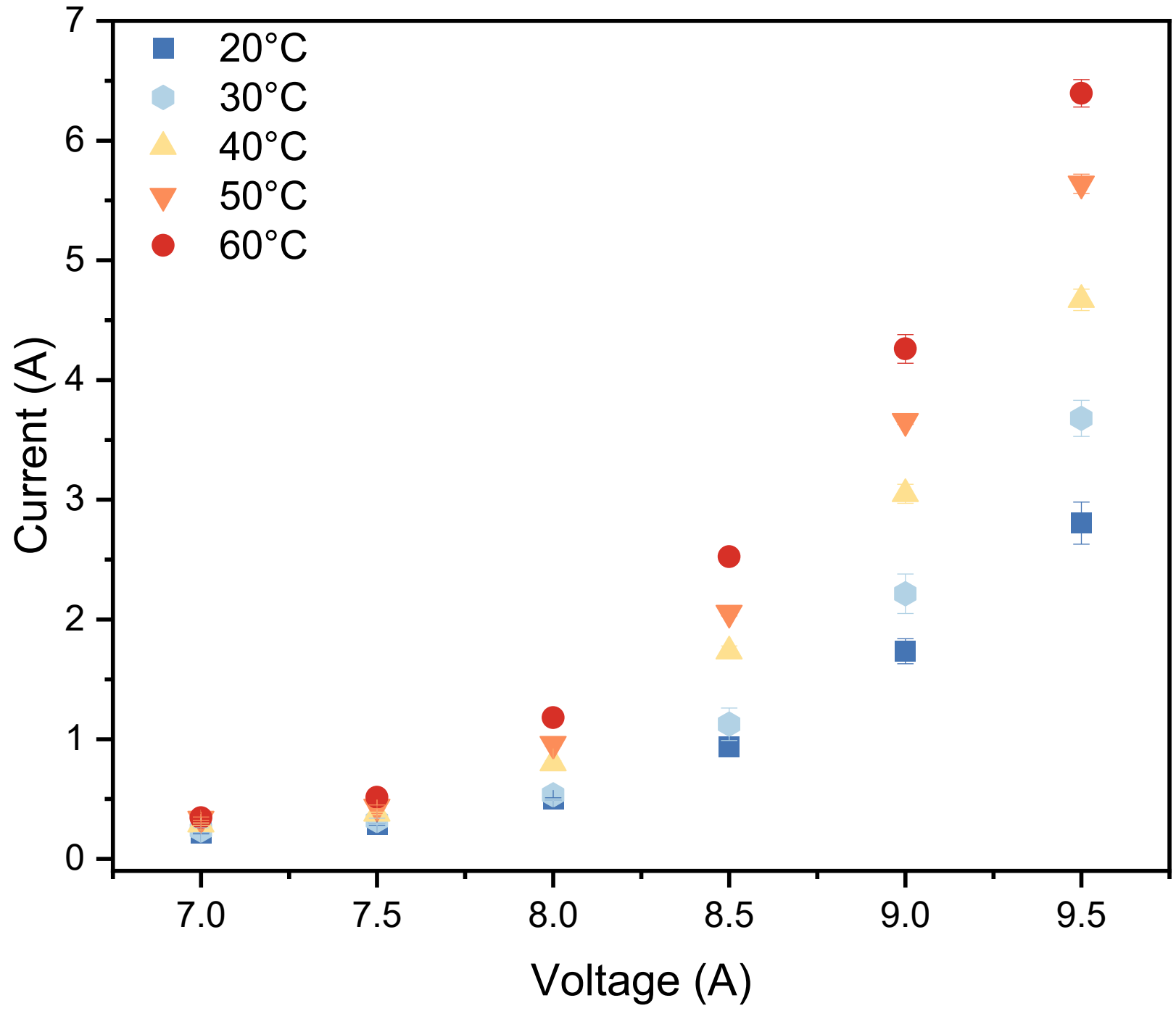

Supplementary Fig. 12 Polarisation curve of our 5-cell AWE electrolyser using 6 M KOH as electrolyte. Error bars represent standard deviation from two independent replicates.

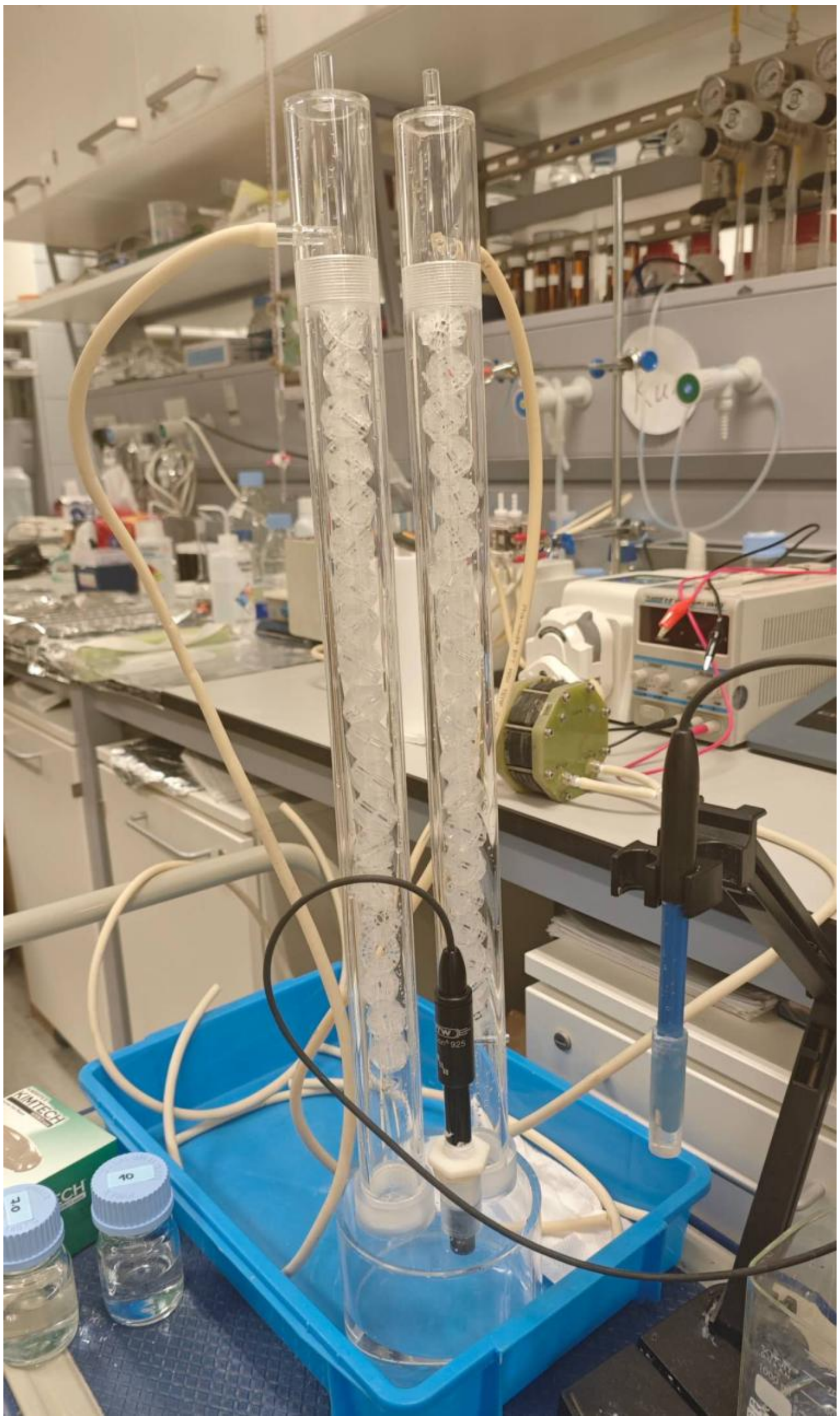

Supplementary Fig. 13 A packed-bed degassing tower used for H_2 and O_2 separation from KOH.

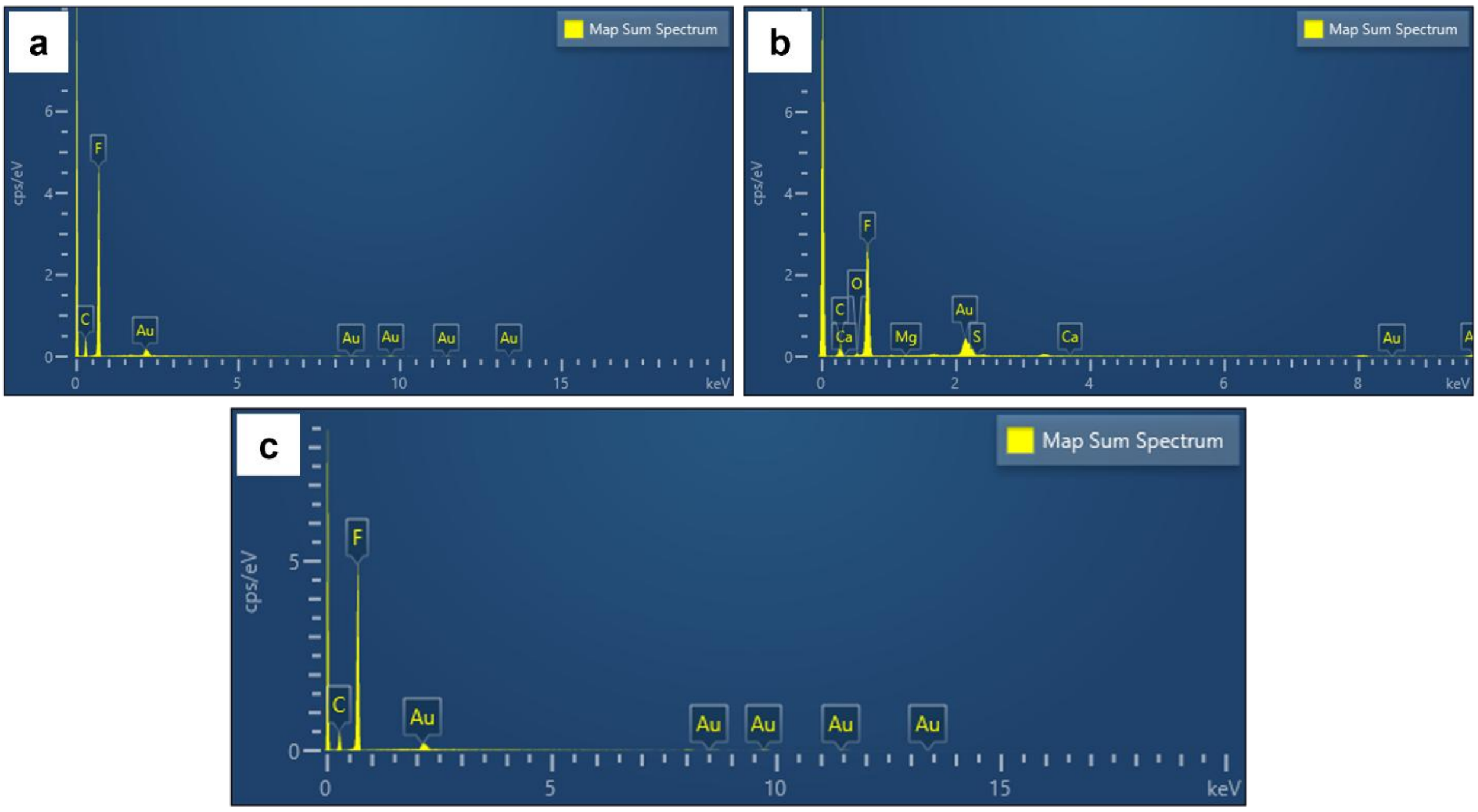

Supplementary Fig. 14 EDX spectrum of the PTFE membrane. Surface elemental composition of **a** the pristine PTFE membrane; **b** the PTFE membrane after 500-h operation without cleaning; and **c** the cleaned PTFE membrane after 500-h operation. Conditions: [feed] = seawater, [KOH] = 6.0 M, T_{feed} = 40°C, T_{KOH} = 20°C.

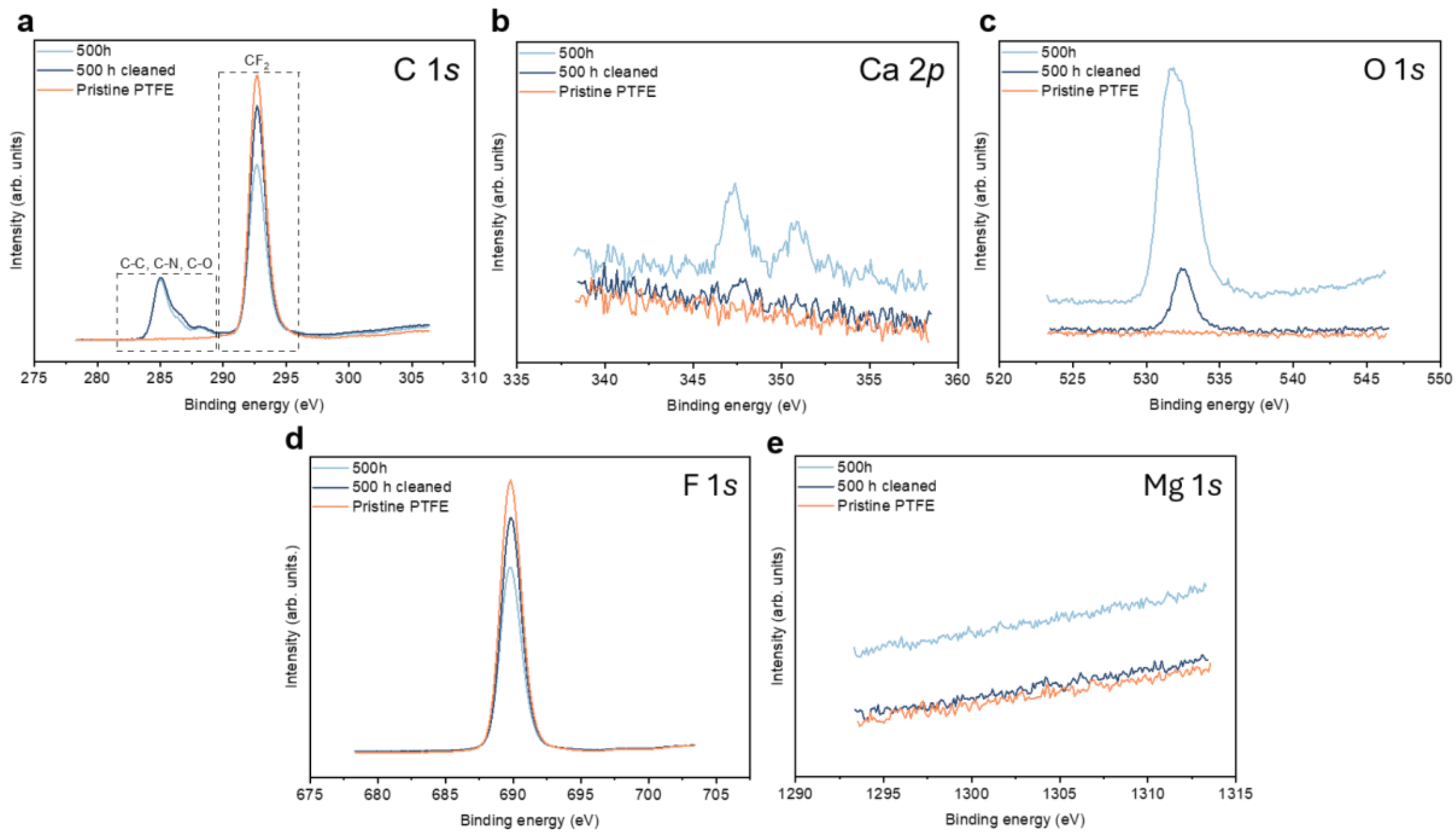

Supplementary Fig. 15 XPS spectra of the PTFE membrane. High-resolution XPS spectrum of the pristine PTFE membrane, the membrane after 500 h of operation, and the membrane after cleaning following the 500 h test, showing the regions for **a** carbon (C 1*s*); **b** calcium (Ca 2*p*); **c** oxygen (O 1*s*), **d** fluorine (F 1*s*); and **e** magnesium (Mg 1*s*). Conditions: [feed] = seawater, [KOH] = 6.0 M, T_{feed} = 40°C, T_{KOH} = 20°C.

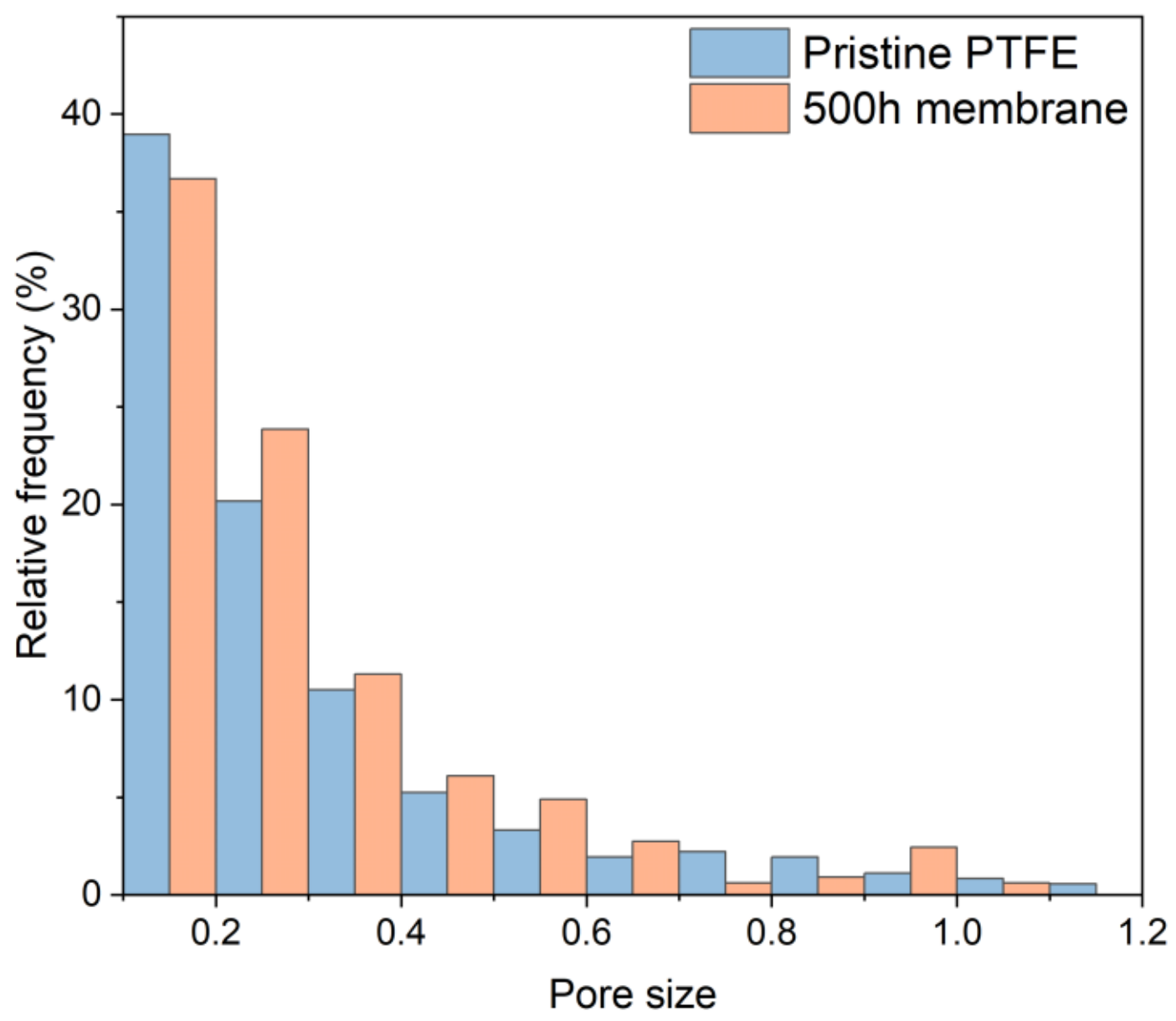

Supplementary Fig. 16 Pore size distribution of the PTFE membrane. The pore diameter histogram of **a** the pristine PTFE membrane; and **b** the PTFE membrane after 500-h operation. Conditions: [feed] = seawater, [KOH] = 6.0 M, T_{feed} = 40°C, T_{KOH} = 20°C.

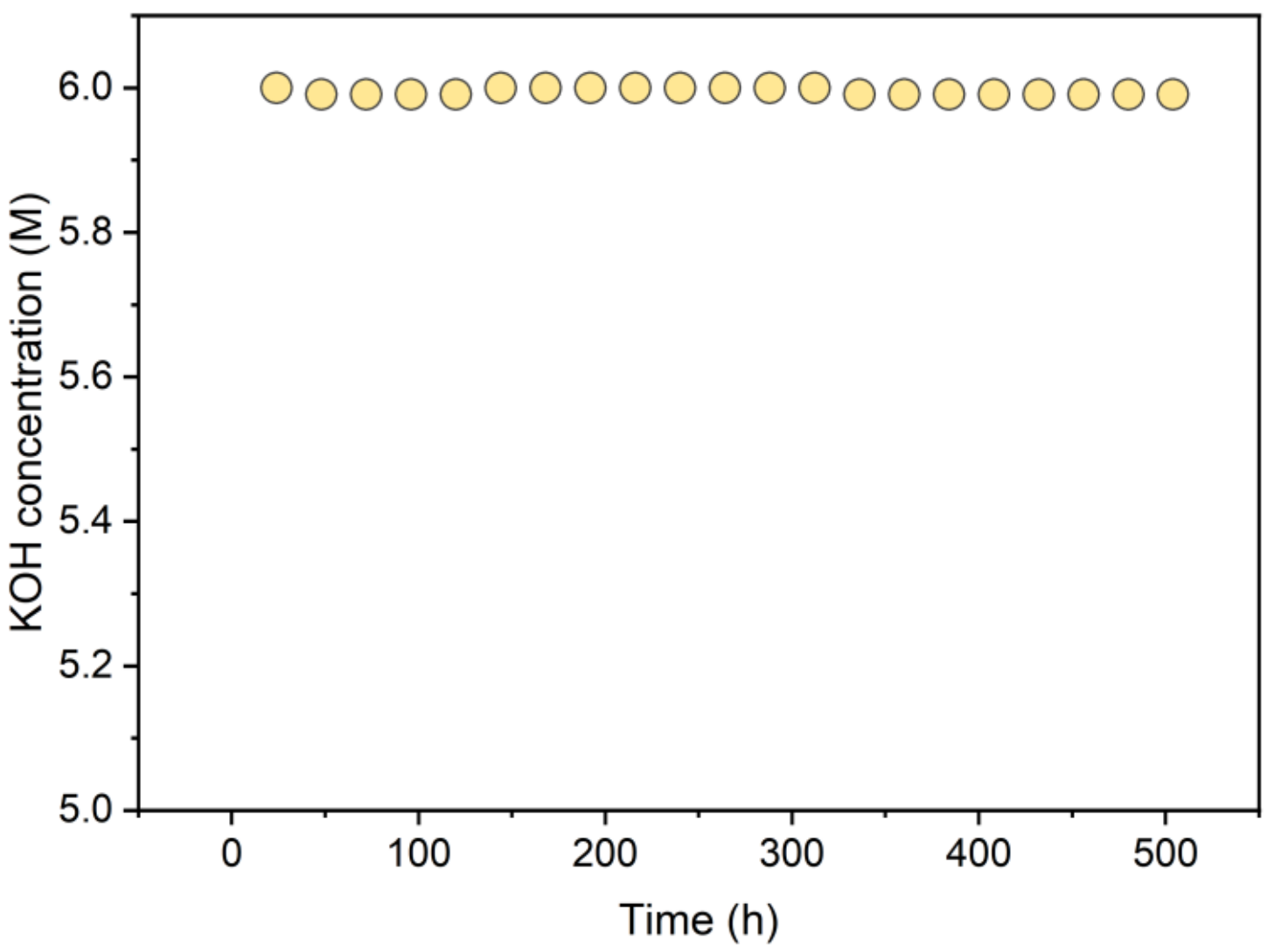

Supplementary Fig. 17 The KOH concentration during the 500-h continuous operation of OMD-AWE. Conditions: [feed] = seawater, [KOH] = 6 M, T_{feed} = 40°C, T_{KOH} = 20°C.

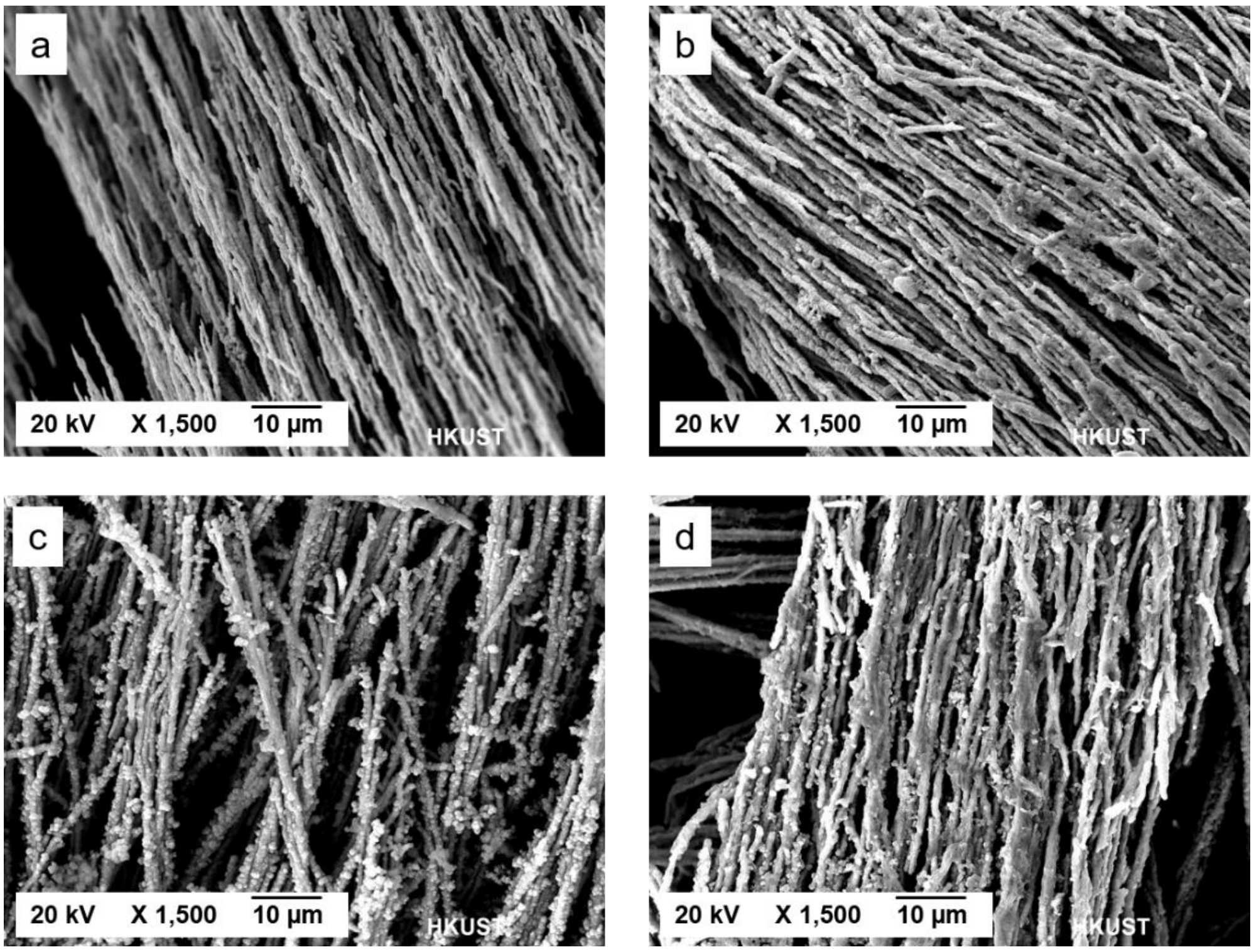

Supplementary Fig. 18 SEM images of the electrodes before and after 500-h operation. Surface morphology of **a** the pristine Ni nanowire cathode; **b** the Ni nanowire cathode after 500-h operation; **c** pristine NiFe anode; and **d** NiFe anode after 500-h operation. Conditions: [feed] = seawater, [KOH] = 6.0 M, T_{feed} = 40°C, T_{KOH} = 20°C.

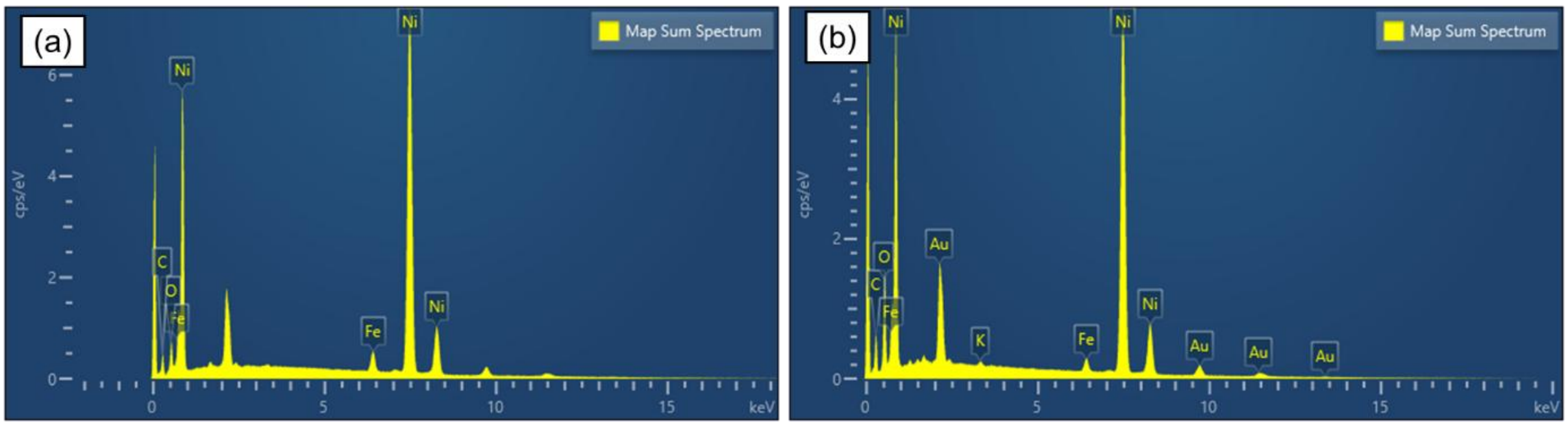

Supplementary Fig. 19 EDX spectrum of the electrodes after the 500-h operation. Surface elemental composition of **a** the Ni nanowire cathode after 500-h operation; and **b** the NiFe anode after 500-h operation. Conditions: [feed] = seawater, [KOH] = 6.0 M, T_{feed} = 40°C, T_{KOH} = 20°C.

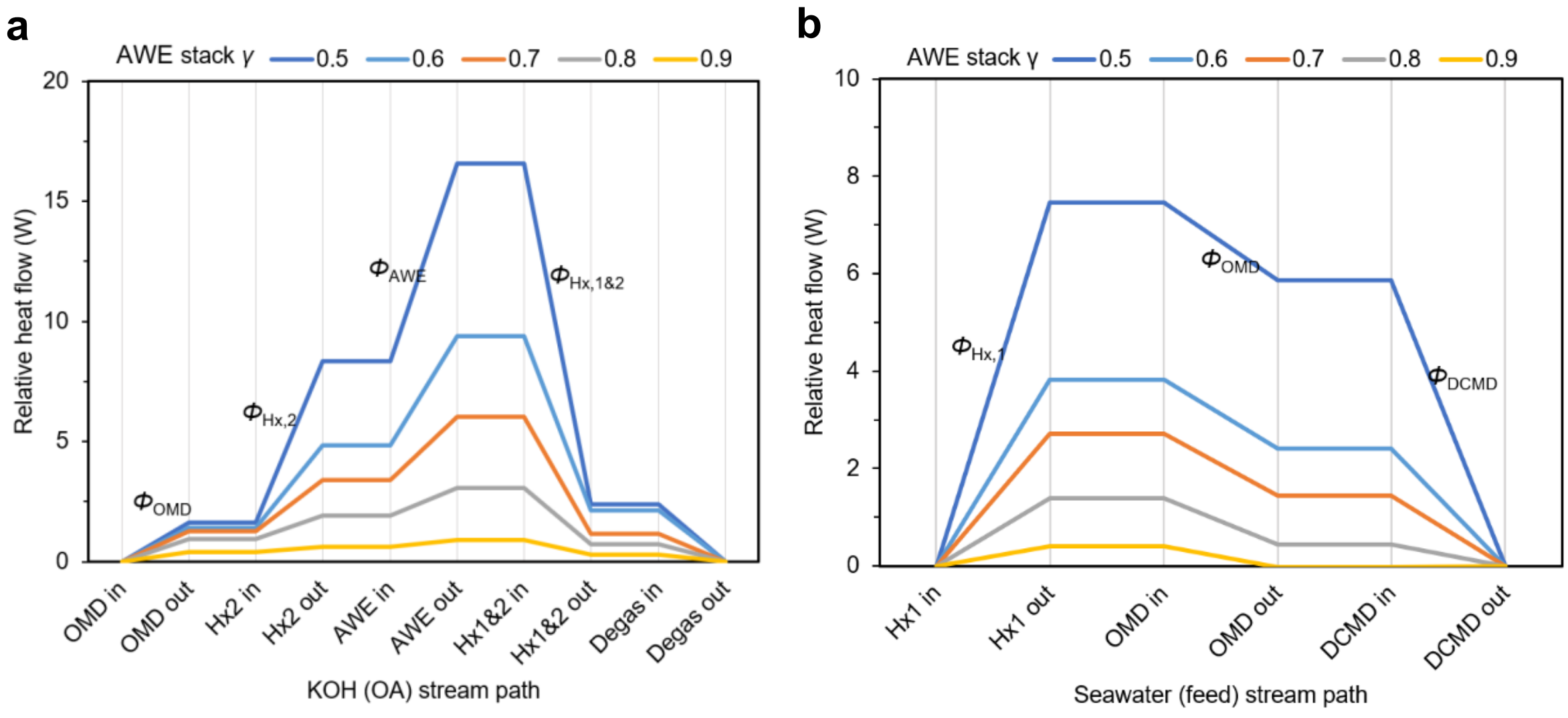

Supplementary Fig. 20 Heat flow profile. Relative heat flux of **a** OA and **b** feed streams compared to their initial RT conditions when circulating within their respective paths,

This figure shows the heat flow profile of the OA and feed stream along their respective paths (relative to their initial RT conditions), calculated from Φ_{AWE}, Φ_{OMD}, and Φ_{Hx} (Supplementary Fig. 21). Starting from the degas tower, the OA stream gains heat from the OMD module and then significantly from heat exchanger #2 and AWE stack, leading to its peak value. Subsequently, it loses heat as it passes through both heat exchangers. The remaining residual heat is then dissipated at the degas tower. The feed first gains heat from heat exchanger #1, where it recovers heat from the hotter OA stream. It then supplies heat to the OMD module for vapor transport, causing a reduction in its heat flow. The residual heat of feed after OMD is used for secondary DCMD water production. These profiles demonstrate effective thermal balance, with heat flow returning to baseline levels at the end of each path.

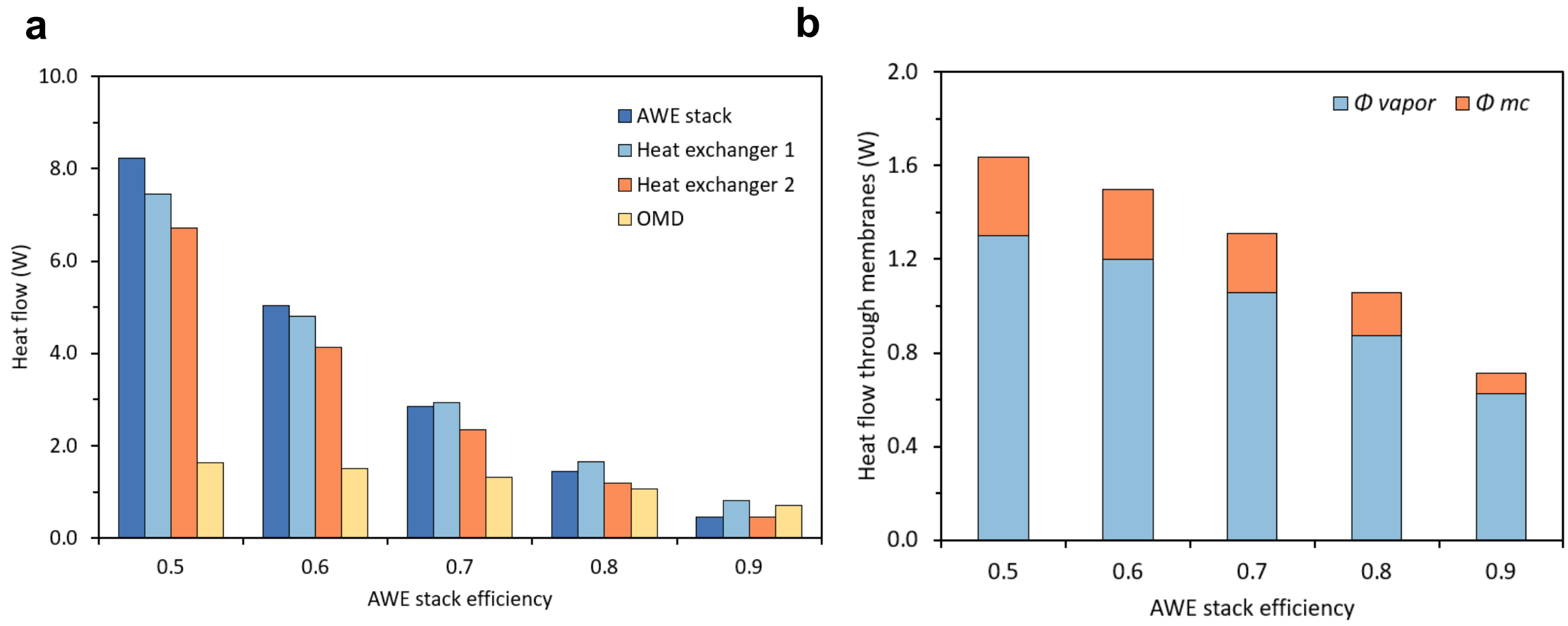

Supplementary Fig. 21 The heat flow of key units in OMD-AWE with different AWE stack efficiency (γ). a The heat flow from the AWE stack, the two exchangers, and OMD membrane module with different γ; and **b** the difference in heat flow between vapor transport (Φ_{vapor}) and membrane conduction (Φ_{mc}) changed with γ.

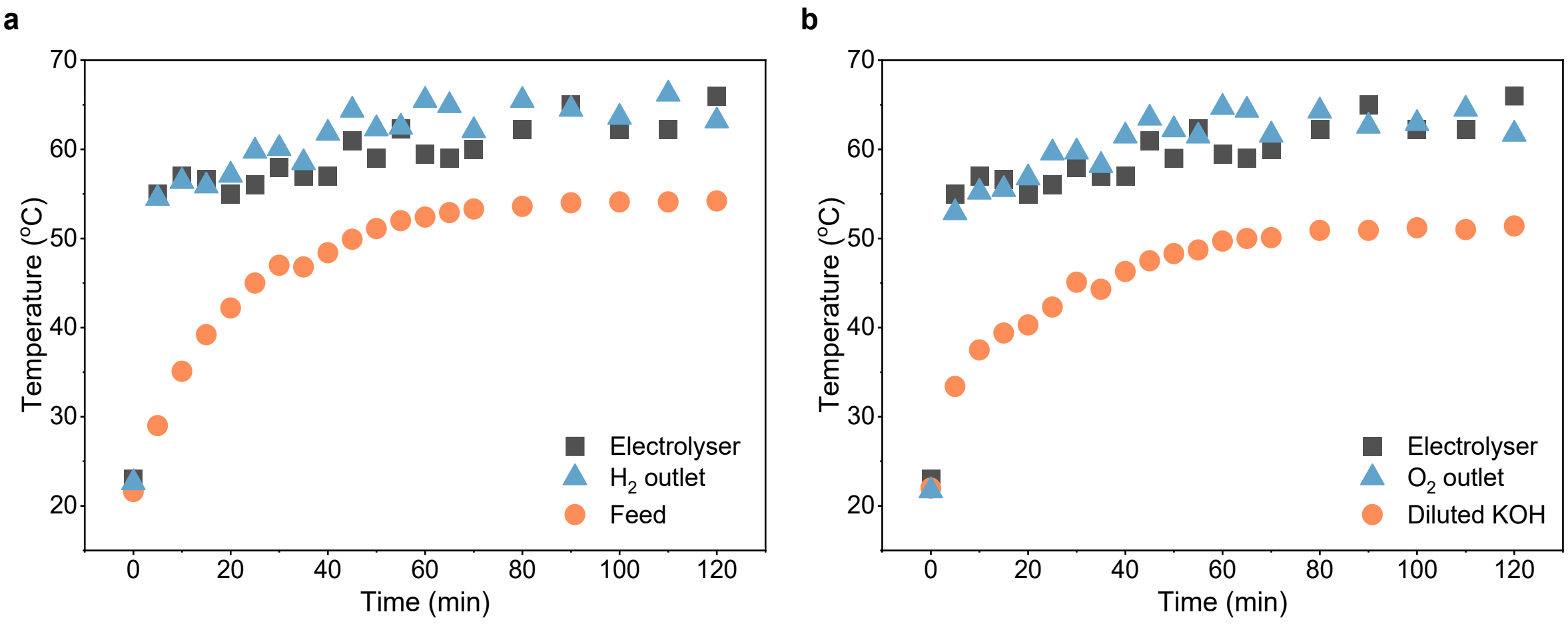

Supplementary Fig. 22 Temperature profiles demonstrate heat harvest in the OMD-AWE system by using heat exchangers. a The temperature change of the OMD feed solution as it harvests heat from the hydrogen (H_2) stream; and **b** the temperature profile of the diluted KOH solution as it recovers heat from the oxygen (O_2) stream just before entering the electrolyser.

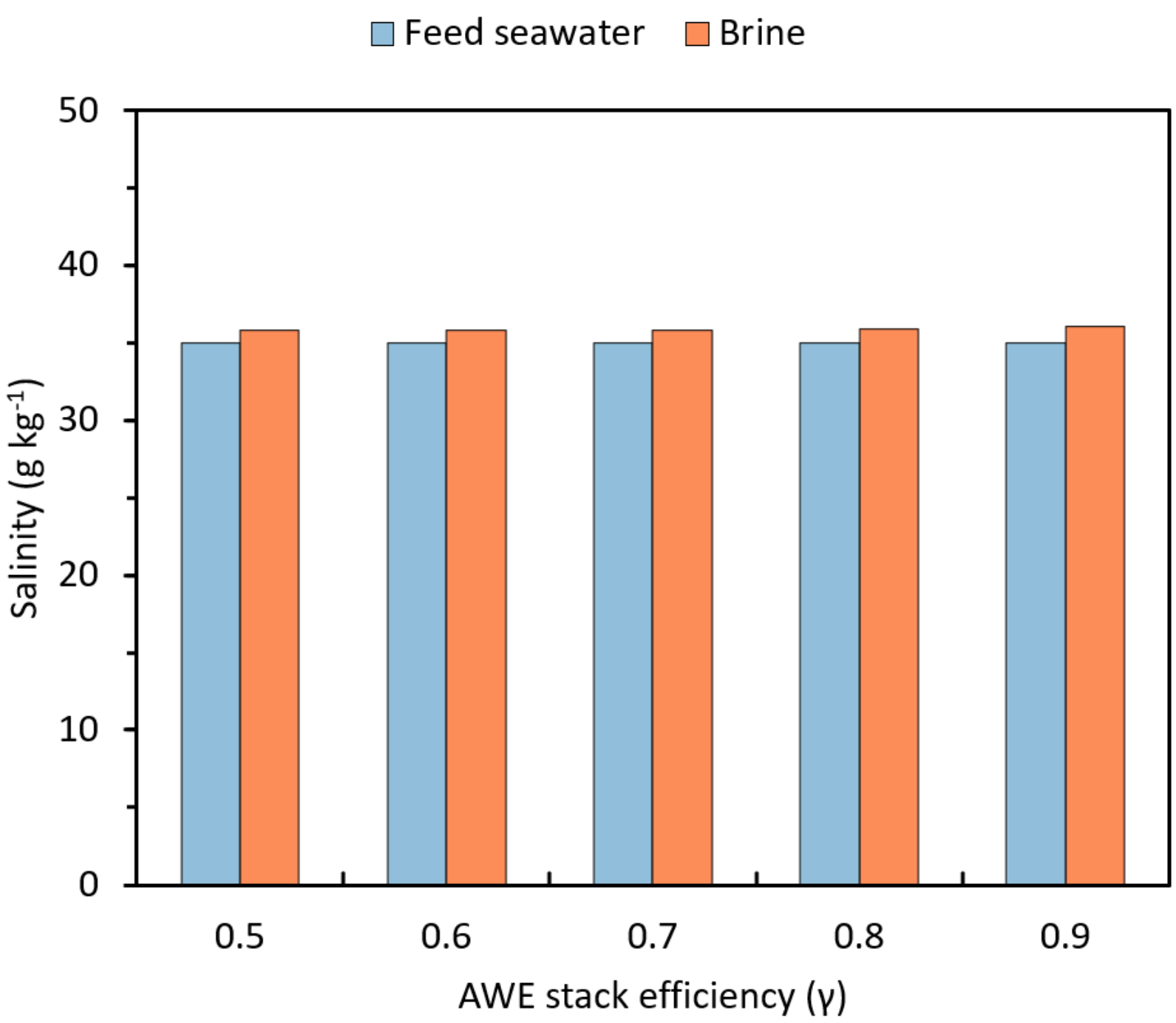

Supplementary Fig. 23 Salinity changes from feed seawater (standard 35 g kg^{-1}) to brine at designed water recovery rates.

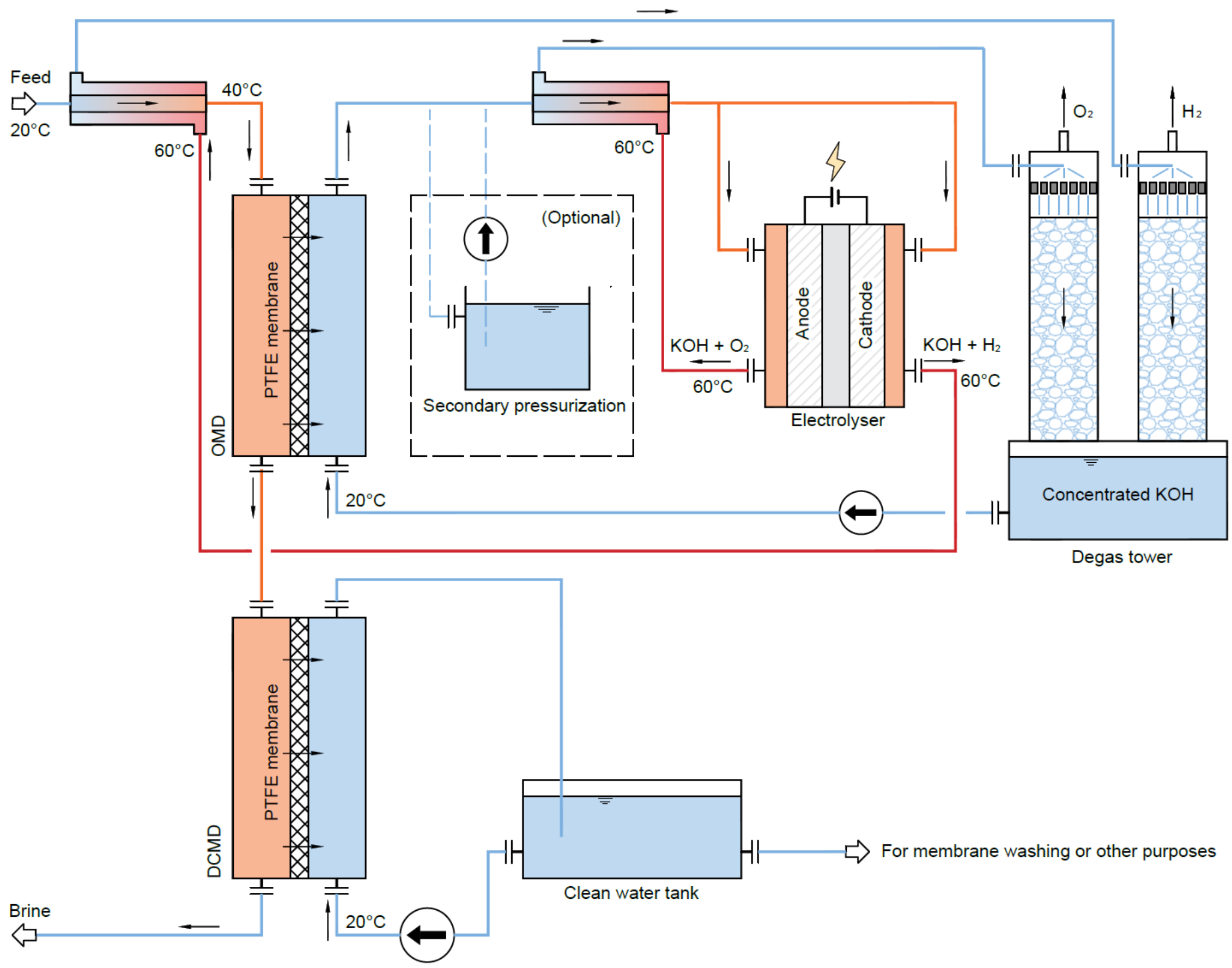

Supplementary Fig. 24 A schematic showing how surplus heat powers additional membrane modules for creating valuable secondary water output.

The OMD feed outlet stream retains heat above RT, which can be utilised for additional clean water production through a secondary DCMD module as shown in Supplementary Fig. 24. As the warm stream cools to RT, the released heat flow via DCMD (Φ_{DCMD}) drives surplus water vapour transport and thermal conduction as expressed by Eq. S29. Based on this available heat, we can calculate the required DCMD membrane area (S_{DCMD}) with the corresponding surplus water flux ($J_{\mathrm{w,DCMD}}$). The system's ability to co-produce clean water is quantified by a Surplus Water Index (SWI), establishing a baseline of 1 for the water used in electrolysis and adding to it the ratio of the surplus DCMD water to the primary OMD water (Eq. S30). An SWI above 1 indicates a net surplus of clean water, demonstrating simultaneous H_2 and clean water production.

$$\Phi_{DCMD} = c_{seawater} q_{feed} \left(T_{OMD,feed,out} - RT\right) = \left[c_m \theta \Delta P_v \Delta H_v + U \frac{\left(T_{OMD,feed,out} - 20°C\right)}{2}\right] \cdot S_{DCMD} \quad (S29)$$

$$Surplus\ Water\ Index\ (SWI) = 1 + \frac{J_{w,DCMD} S_{DCMD}}{J_{w,OMD} S_{OMD}} \quad (S30)$$

When commercial AWE stacks operate at γ of 0.65–0.80, the system is not only thermally self-sustaining but also achieves an SWI of over 1.4 (Supplementary Fig. 25), co-producing 40% more clean water beyond electrolysis needs, all while maintaining a low water flux penalty.

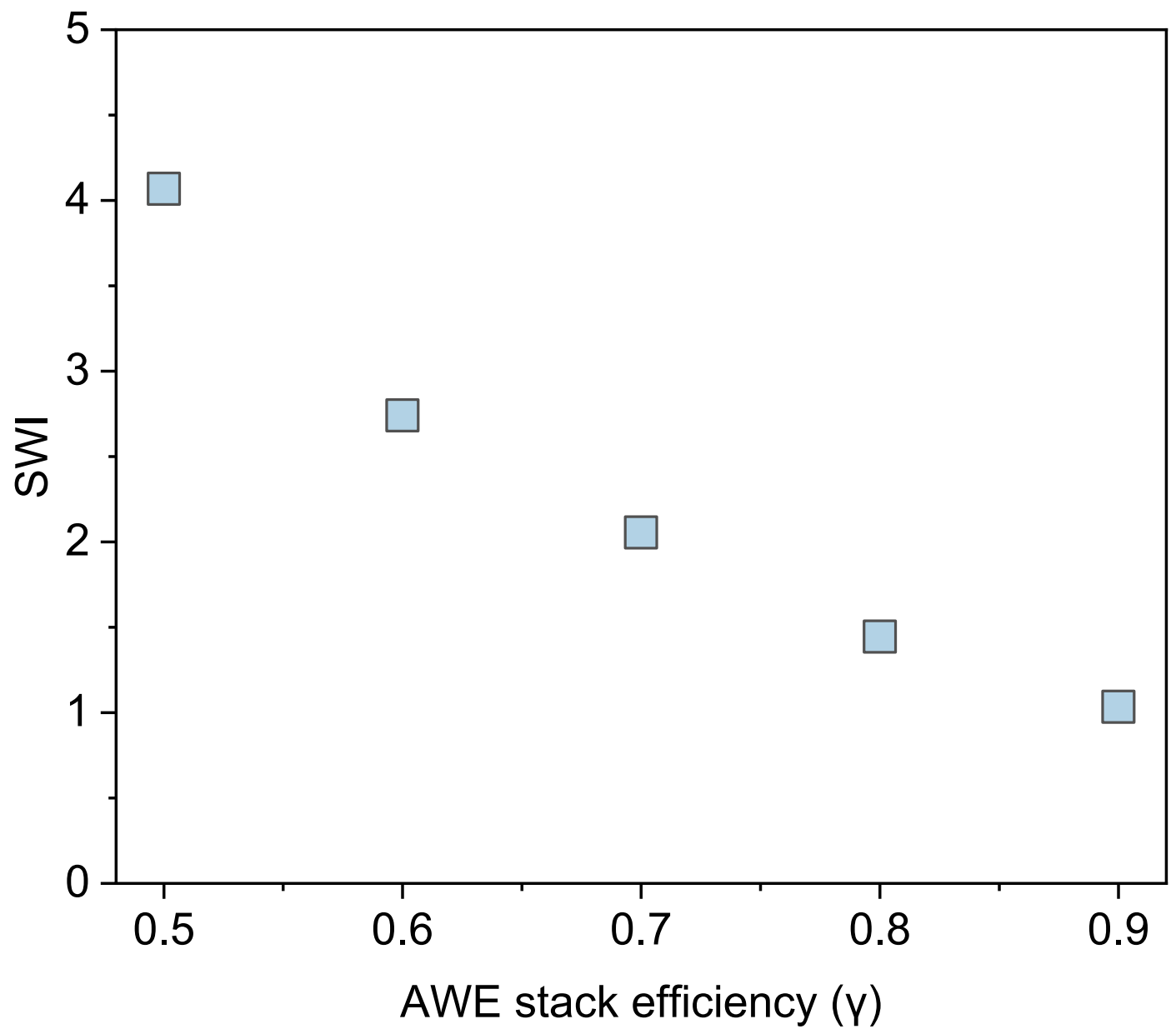

Supplementary Fig. 25 Surplus Water Index (SWI) as a function AWE stack efficiency (γ).

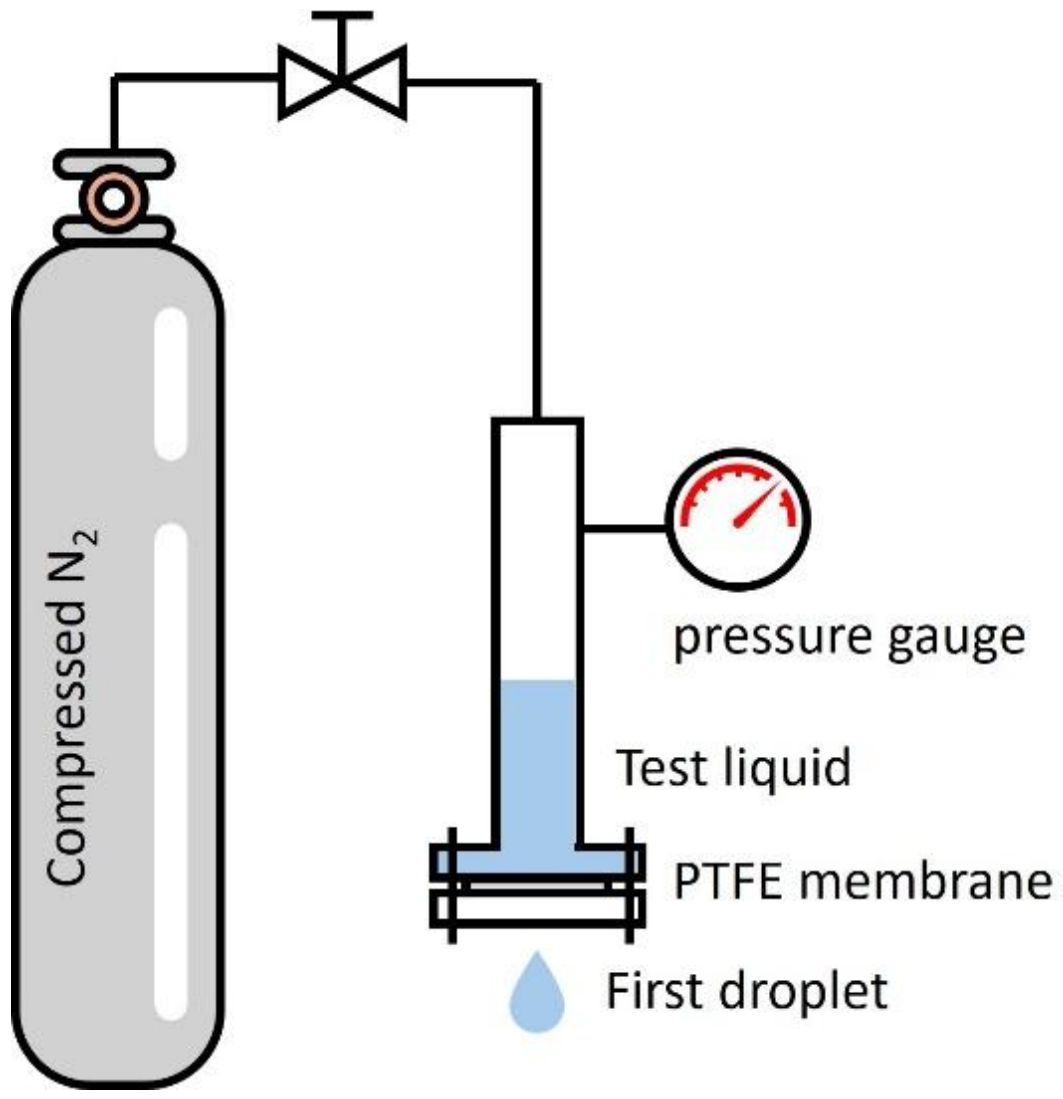

Supplementary Fig. 26 The setup for measuring liquid entry pressure (LEP) of the PTFE membranes.

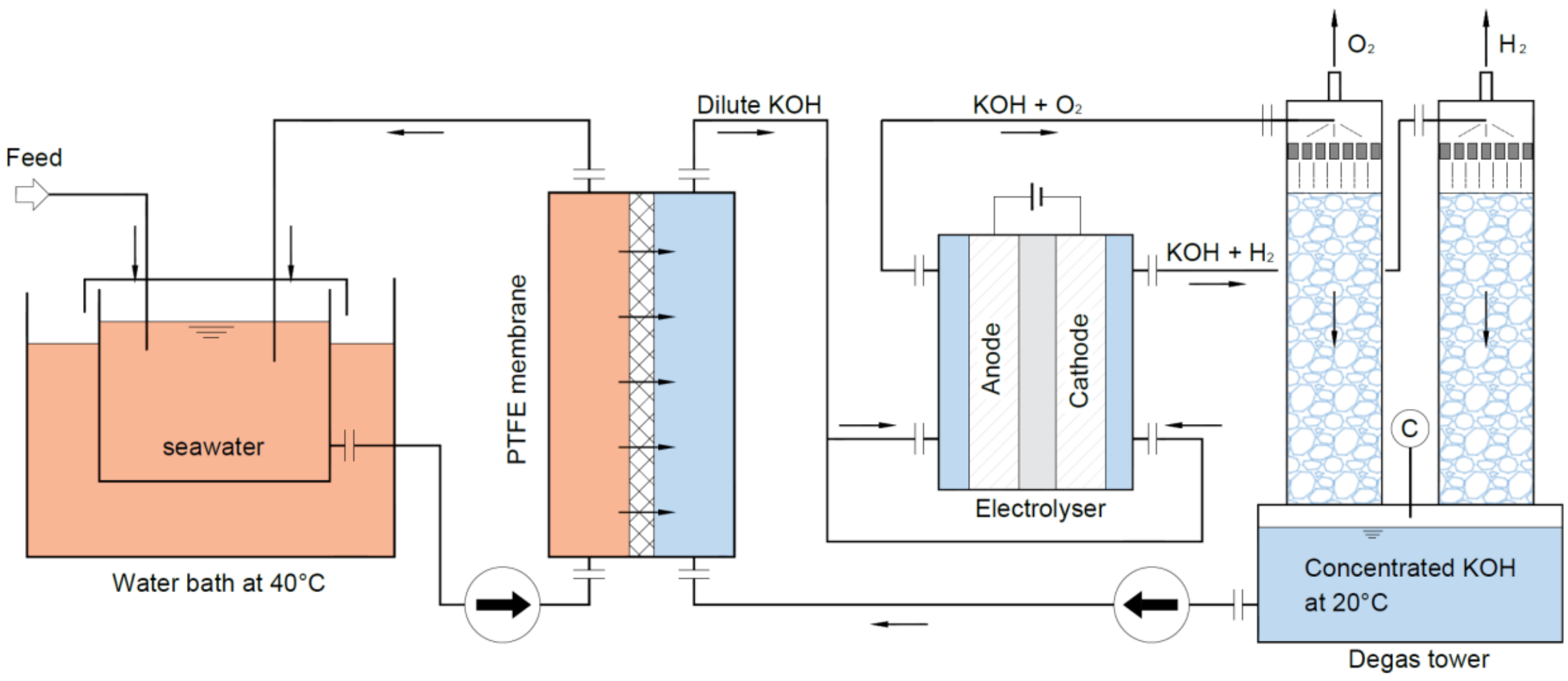

Supplementary Fig. 27 Schematic diagram of the membrane-integrated electrolysis system. The figure illustrates the configuration and main components of the membrane-integrated alkaline water electrolysis (AWE) system, including the feed tank, membrane module, electrolyser, and degas tower.

Supplementary Table 12 Detailed composition of the real seawater sample used in this study.

Parameter	Value
pH	7.86
Conductivity (mS cm^{-1})	52.6
TOC (mg L^{-1} as C)	2.38
Sulphate (mg L^{-1})	3,345.79
Chloride (mg L^{-1})	19,235.88
Magnesium (mg L^{-1})	1,222.44
Calcium (mg L^{-1})	325.68

Supplementary Table 13 Surface elemental composition analysis of the PTFE membrane during accelerated scaling tests. EDX showing surface elemental composition of a pristine PTFE membrane and the PTFE membrane after 20 cycles of accelerated scaling testing.

PTFE membrane	Element	Wt%	Wt% Sigma	Atom (%)
Pristine	C	26.55	0.72	36.37
	F	73.45	0.72	63.63
	Others*	0	0	0
	F/C ratio	2.77	–	1.75
After 20 cycles without cleaning	C	18.88	0.92	28.25
	F	48.1	0.75	45.51
	Others*	33.02	–	26.23
	F/C ratio	2.55	–	1.61
After 20 cycles cleaned	C	25.36	0.84	35.55
	F	67.87	0.82	60.16
	Others*	6.77	–	4.30
	F/C ratio	2.68	–	1.69

*"Others" include Ca, S, and O.

Supplementary Table 14 Surface elemental composition analysis of the PTFE membrane during accelerated scaling tests in the presence of organic matter. EDX showing surface elemental composition of a PTFE membrane without cleaning using gypsum + Suwannee River Natural Organic Matter (SRNOM) as the feed and gypsum only as the feed.

PTFE membrane	Element	Wt%	Wt% Sigma	Atom (%)
Pristine	C	26.55	0.72	36.37
	F	73.45	0.72	63.63
	Others*	0	0	0
	F/C ratio	2.77	–	1.75
Gypsum only	C	18.43	1.15	27.16
	F	41.43	0.84	38.6
	Others*	40.14	–	34.23
	F/C ratio	2.25	–	1.42
Gypsum+SRNOM	C	30.81	0.77	41.29
	F	67.91	0.78	57.54
	Others*	1.28	–	1.17
	F/C ratio	2.20	–	1.39

*"Others" include Ca, S, and O.

Supplementary Table 15 Surface elemental composition analysis of the PTFE membrane. EDX showing surface elemental composition of the pristine PTFE membrane and the PTFE membrane after 500 h of operation before and after cleaning.

PTFE membrane	Element	Wt%	Wt% Sigma	Atom (%)
Pristine	C	26.55	0.72	36.37
	F	73.45	0.72	63.63
	Others*	0	0	0
	F/C ratio	2.77	–	1.75
After 500 hours without cleaning	C	28.54	0.97	38.58
	F	69.13	0.97	59.09
	Others*	2.33	–	2.32
	F/C ratio	2.42	–	1.53
After 500 hours cleaned	C	27.28	0.35	37.24
	F	72.71	0.35	62.75
	Others*	0	0	0
	F/C ratio	2.67	–	1.69

*"Others" include Ca, Mg, S, and O.

Supplementary Table 16 Surface elemental composition analysis of the electrodes. EDX showing surface elemental composition of both pristine and used anode and cathode after 500 h of operation.

Electrode	Elements	Pristine electrodes Atom (%)	Electrodes after 500 h Atom (%)
Anode	Ni	93.23	92.13
	Fe	6.77	7.86
Cathode	Ni	99.66	96.90
	Fe	0.34	3.10

Supplementary Table 17 Operating parameters for OMD-AWE thermal self-circulation.

γ	$T_{AWE,in}$	$T_{AWE,out}$	$T_{AWE,avg}$	q_{OA}	q_{feed}	$T_{OMD,OA,out}$	$T_{OMD,feed,out}$	$T_{OMD,feed,in}$	$T_{OMD,OA,in}$
/	°C	°C	°C	kg h^{-1}	kg h^{-1}	°C	°C	°C	°C
0.5	35.1	50.0	42.6	0.568	0.334	23.0	35.6	40	20
0.6	35.8	50.0	42.9	0.366	0.215	24.2	33.8	40	20
0.7	36.8	50.0	43.4	0.223	0.131	26.0	31.1	40	20
0.8	38.2	50.0	44.1	0.126	0.074	28.6	27.2	40	20
0.9	41.5	50.0	45.8	0.056	0.036	33.1	22.5	40	20

(Condition: S_{OMD} = 1 cm^2)

Supplementary references

1. Scheibel Cassol, G., Shang, C., An, A. K., Khanzada, N. K., Ciucci, F., Manzotti, A., Westerhoff, P., Song, Y. & Ling, L. Ultra-fast green hydrogen production from municipal wastewater by an integrated forward osmosis-alkaline water electrolysis system. *Nat. Commun.* **15**, 2617 (2024).

2. Becker, H., Murawski, J., Shinde, D. V., Stephens, I. E. L., Hinds, G. & Smith, G. Impact of impurities on water electrolysis: a review. *Sustain. Energy Fuels* **7**, 1565–1603 (2023).

3. Wijmans, J. G. & Baker, R. W. The solution-diffusion model: a review. *J. Memb. Sci.* **107**, 1–21 (1995).

4. Lu, S., Song, Y., Guo, K., Chen, X., Xu, J. & Zhao, L. Effect of aqueous electrolytes on the electrochemical behaviors of ordered mesoporous carbon composites after KOH activation as supercapacitors electrodes. *Journal of Electroanalytical Chemistry* **818**, 58–67 (2018).

5. Arena, J. T., Chwatko, M., Robillard, H. A. & McCutcheon, J. R. pH Sensitivity of Ion Exchange through a Thin Film Composite Membrane in Forward Osmosis. *Environ. Sci. Technol. Lett.* **2**, 177–182 (2015).

6. Coronell, O., Mariñas, B. J., Zhang, X. & Cahill, D. G. Quantification of Functional Groups and Modeling of Their Ionization Behavior in the Active Layer of FT30 Reverse Osmosis Membrane. *Environ. Sci. Technol.* **42**, 5260–5266 (2008).

7. Nguyen, H. T., Nguyen, N. C., Chen, S.-S., Ngo, H. H., Guo, W. & Li, C.-W. A new class of draw solutions for minimizing reverse salt flux to improve forward osmosis desalination. *Science of The Total Environment* **538**, 129–136 (2015).

8. Chun, Y., Mulcahy, D., Zou, L. & Kim, I. A Short Review of Membrane Fouling in Forward Osmosis Processes. *Membranes (Basel).* **7**, 30 (2017).

9. Freger, V. Swelling and Morphology of the Skin Layer of Polyamide Composite Membranes: An Atomic Force Microscopy Study. *Environ. Sci. Technol.* **38**, 3168–3175 (2004).

10. Gohil, J. M. & Suresh, A. K. Chlorine attack on reverse osmosis membranes: Mechanisms and mitigation strategies. *J. Memb. Sci.* **541**, 108–126 (2017).

11. Yang, L., Wang, J., Chen, X., Yuan, C., Bai, L., Meng, S. & Cao, G. Effect of aluminum and manganese ions on the chlorination of polyamide membranes. *Desalination* **567**, 116989 (2023).

12. Hausmann, J. N., Schlögl, R., Menezes, P. W. & Driess, M. Is direct seawater splitting economically meaningful? *Energy Environ. Sci.* **14**, 3679–3685 (2021).

13. Lee, B., Wang, L., Wang, Z., Cooper, N. J. & Elimelech, M. Directing the research agenda on water and energy technologies with process and economic analysis. *Energy Environ. Sci.* **16**, 714–722 (2023).

14. Alkhudhiri, A., Darwish, N. & Hilal, N. Membrane distillation: A comprehensive review. *Desalination* **287**, 2–18 (2012).

15. Wang, Z., Zheng, F., Wu, Y. & Wang, S. Membrane osmotic distillation and its mathematical simulation. *Desalination* **139**, 423–428 (2001).

16. Qtaishat, M., Matsuura, T., Kruczek, B. & Khayet, M. Heat and mass transfer analysis in direct contact membrane distillation. *Desalination* **219**, 272–292 (2008).

17. Chen, C. T. A. High-pressure specific heat capacities of pure water and seawater. *J. Chem. Eng. Data* **32**, 469–472 (1987).

18. Teke, İ., Ağra, Ö., Atayılmaz, Ş. Ö. & Demir, H. Determining the best type of heat exchangers for heat recovery. *Appl. Therm. Eng.* **30**, 577–583 (2010).

19. Ali, A. S. & Bounahmidi, T. Photovoltaic-thermal driven membrane distillation water desalination: Dynamic simulation in an actual weather condition. *Energy* **324**, 135943 (2025).

20. Xie, H., Zhao, Z., Liu, T., Wu, Y., Lan, C., Jiang, W., Zhu, L., Wang, Y., Yang, D. & Shao, Z. A membrane-based seawater electrolyser for hydrogen generation. *Nature* **612**, 673–678 (2022).

21. Yu, L., Ning, M., Wang, Y., Yuan, C. & Ren, Z. Direct seawater electrolysis for hydrogen production. *Nat. Rev. Mater.* https://doi.org/10.1038/s41578-025-00826-x (2025) doi:10.1038/s41578-025-00826-x.

22. Fei, H., Liu, R., Liu, T., Ju, M., Lei, J., Wang, Z., Wang, S., Zhang, Y., Chen, W., Wu, Z., Ni, M. & Wang, J. Direct Seawater Electrolysis: From Catalyst Design to Device Applications. *Advanced Materials* https://doi.org/10.1002/adma.202309211 (2023) doi:10.1002/adma.202309211.

23. Winter, L. R., Cooper, N. J., Lee, B., Patel, S. K., Wang, L. & Elimelech, M. Mining Nontraditional Water Sources for a Distributed Hydrogen Economy. *Environ. Sci. Technol.* **56**, 10577–10585 (2022).

24. Schwantes, R., Morales, Y., Pomp, E., Singer, J., Chavan, K. & Saravia, F. Thermally driven ultrapure water production for water electrolysis – A techno-economic analysis of membrane distillation. *Desalination* **608**, 118848 (2025).

25. Moharram, N. A., Bayoumi, S., Hanafy, A. A. & El-Maghlany, W. M. Techno-economic analysis of a combined concentrated solar power and water desalination plant. *Energy Convers. Manag.* **228**, 113629 (2021).

26. Kumar, P., Date, A., Mahmood, N., Kumar Das, R. & Shabani, B. Freshwater supply for hydrogen production: An underestimated challenge. *Int. J. Hydrogen Energy* **78**, 202–217 (2024).

27. Valladares Linares, R., Li, Z., Yangali-Quintanilla, V., Ghaffour, N., Amy, G., Leiknes, T. & Vrouwenvelder, J. S. Life cycle cost of a hybrid forward osmosis – low pressure reverse osmosis system for seawater desalination and wastewater recovery. *Water Res.* **88**, 225–234 (2016).

28. Lee, B., Cho, H.-S., Kim, H., Lim, D., Cho, W., Kim, C.-H. & Lim, H. Integrative techno-economic and environmental assessment for green H2 production by alkaline water electrolysis based on experimental data. *J. Environ. Chem. Eng.* **9**, 106349 (2021).

29. Gao, F.-Y., Yu, P.-C. & Gao, M.-R. Seawater electrolysis technologies for green hydrogen production: challenges and opportunities. *Curr. Opin. Chem. Eng.* **36**, 100827 (2022).

30. González, D., Amigo, J. & Suárez, F. Membrane distillation: Perspectives for sustainable and improved desalination. *Renewable and Sustainable Energy Reviews* **80**, 238–259 (2017).

31. Ding, S., Guo, B., Hu, S., Tian, Z., Gu, J., Zhang, T., Yang, F. & Ouyang, M. Experimental and modeling study on energy flow of 250 kW alkaline water electrolysis system under steady state conditions and cold start process. *Fuel* **350**, 128799 (2023).

32. Liu, T., Zhao, Z., Tang, W., Chen, Y., Lan, C., Zhu, L., Jiang, W., Wu, Y., Wang, Y., Yang, Z., Yang, D., Wang, Q., Luo, L., Liu, T. & Xie, H. In-situ direct seawater electrolysis using floating platform in ocean with uncontrollable wave motion. *Nat. Commun.* **15**, 5305 (2024).

33. Wu, L., Xu, Y., Wang, Q., Zou, X., Pan, Z., Leung, M. K. H. & An, L. Direct seawater electrolysis for green hydrogen production: electrode designs, cell configurations, and system integrations. *Energy Environ. Sci.* **18**, 4596–4624 (2025).

34. Tang, J., Guo, K., Guan, D., Hao, Y. & Shao, Z. A semi-vapor electrolysis technology for hydrogen generation from wide water resources. *Energy Environ. Sci.* **17**, 7394–7402 (2024).

35. Xu, J., Liang, Y., Luo, X., Chen, J., Yang, Z. & Chen, Y. Techno-economic-environmental analysis of direct-contact membrane distillation systems integrated with low-grade heat sources: A multi-objective optimization approach. *Appl. Energy* **349**, 121640 (2023).

36. Liang, Y., Xu, J., Luo, X., Chen, J., Yang, Z. & Chen, Y. Cradle-to-grave life cycle assessment of membrane distillation systems for sustainable seawater desalination. *Energy Convers. Manag.* **266**, 115740 (2022).

37. Muñoz, I. & Fernández-Alba, A. R. Reducing the environmental impacts of reverse osmosis desalination by using brackish groundwater resources. *Water Res.* **42**, 801–811 (2008).

38. Tarnacki, K., Meneses, M., Melin, T., van Medevoort, J. & Jansen, A. Environmental assessment of desalination processes: Reverse osmosis and Memstill®. *Desalination* **296**, 69–80 (2012).

39. Yuan, Y., Lu, J., Gu, D., Lou, Y., Xue, N., Li, G., Liao, W. & Zhang, N. Deduction of carbon footprint for membrane desalination processes towards carbon neutrality: A case study on electrodeionization for ultrapure water preparation. *Desalination* **559**, 116648 (2023).

40. Tong, T., Liu, X., Li, T., Park, S. & Anger, B. A Tale of Two Foulants: The Coupling of Organic Fouling and Mineral Scaling in Membrane Desalination. *Environ. Sci. Technol.* **57**, 7129–7149 (2023).
