# Supplementary material for: Thermally symbiotic integration of osmotic membrane distillation and electrolysis for direct seawater hydrogen production
Source: Nat Commun. 2026 Jul 7;17:8388. doi: 10.1038/s41467-026-74854-8 (PMC13473115; doi:10.1038/s41467-026-74854-8)
Supplement: Supplementary file 2 [file 41467_2026_74854_MOESM2_ESM.pdf]

nature portfolio

Peer Review File

Thermally symbiotic integration of osmotic membrane distillation and electrolysis for direct seawater hydrogen production

Corresponding Author: Dr Yinghao Song

This file contains all reviewer reports in order by version, followed by all author rebuttals in order by version.

Version 0:

Reviewer comments:

Reviewer #1

(Remarks to the Author)
Recommendation: Reject
This manuscript presents an evaluation of two membrane-based water purification technologies—osmotic dilution (OD) and osmotic membrane distillation (OMD)—integrated with alkaline water electrolysis (AWE) to enable hydrogen and clean water production. The study explores the use of PTFE and TFC membranes in these systems and introduces a thermal self-circulation strategy utilizing waste heat from the electrolyzer. The integration of membrane technologies with electrolyzers—particularly the reuse of electrolysis waste heat to enhance system efficiency—is a timely and important direction that remains underexplored. While the concept is promising, the current version of the manuscript lacks sufficient technical clarity and does not clearly differentiate its innovations from existing work. In addition, several methodological concerns need to be addressed. With substantial reorganization, a more focused presentation, and clearer articulation of novelty—particularly in the title and abstract—the manuscript could be considerably strengthened. However, at this stage, I do not recommend the manuscript for publication in Nature Communications.

1. Limited novelty
Although the manuscript highlights PTFE membranes and the OMD process as key innovations, these concepts are well-established. The hydrophobic PTFE membrane has been widely used in membrane distillation for decades and is a mature material in the water purification field. The description in the abstract may inadvertently mislead readers into perceiving it as a novel or advanced material.

Similarly, the OMD technique itself has been studied for over a decade. As presented, the OMD system in this work does not appear to deviate significantly from previous OMD designs. The manuscript repeatedly emphasizes the novelty of the system, particularly in lines 218–229, but it is difficult to identify a unique technical advancement from this description. I recommend the authors provide a clear schematic and detailed discussion of the specific design features that distinguish their OMD implementation from existing methods.

2. Concerns about AWE
The integration of OMD with AWE is an interesting concept, but the operating parameters raise significant technical concerns. For example, the applied current density of 1.17 A/cm2, derived from a water-hydrogen balance model (lines 244–249), is considerably higher than typical operational values for alkaline electrolyzers, which generally operate below 0.5 A/cm2. This high current could potentially accelerate degradation of the system.
Furthermore, the manuscript describes a 4.5% voltage increase within the first 24 hours (line 295) as minor, but such a rise in voltage over a short period could be indicative of rapid performance degradation, possibly due to impurities in the water or high current density. It would be helpful if the authors could comment on whether any diagnostic techniques, such as EIS or polarization curves, were conducted to assess the performance of the AWE under these conditions.
It may also be worth discussing whether the water produced via OMD could be buffered or stored, decoupling the electrolysis step from water production, thereby avoiding the need for continuous operation at such high current densities.

3. Framing and focus of the Introduction and Results part
While the authors aim to highlight the advantages of OMD over other methods, such as forward osmosis (FO) or reverse

osmosis (RO), the Introduction devotes considerable space to reviewing FO and RO technologies. A more focused comparison with existing OMD or MD-based systems would be more appropriate and relevant to the manuscript's core claims.
Additionally, a substantial portion of the Results section focuses on performance of TFC membranes under OD conditions. While it is useful to illustrate the limitations of prior approaches, the level of detail dedicated to TFC membranes shifts attention away from the central contribution and may dilute the manuscript's focus.
A large portion of the discussions of mechanism in the Results section are not derived from the authors' experimental data, but rather looks like a literature review that would be more appropriately placed in the Introduction section.

4. Missing experimental details and context
The manuscript reports impressive hydrogen production rates, but does not specify the actual membrane area or module configuration used in the OMD and OD setups. While the AWE stack itself appears to have an active area of 12 cm2 and 5 cells (lines 478–481). Considering the high current density applied, a substantial amount of water is needed, which in turn demands a high rate of clean water production. Therefore, clarification of the membrane size and system scale of the OMD is essential.

5. Additional comments
The title and abstract could be revised to better reflect the full scope of the work, including the TFC membrane and osmotic distillation.
Please write out the full term "osmotic membrane distillation (OMD)" at first mention in the abstract (line 26).
The manuscript currently lacks a clearly labeled "Introduction" section, which may affect readability.
The sentence on lines 44–45 regarding energy losses from RO is unclear and sounds not logical. Please consider revising for clarity and logic flow.

Reviewer #2

(Remarks to the Author)
Direct seawater electrolysis has garnered significant attention, thereby promoting the development of chlorine-resistant and selective electrocatalysts as well as membrane separators. This manuscript submitted by Song et al. integrated hydrophobic polytetrafluoroethylene (PTFE) membranes with alkaline electrolysis enabling sustainable hydrogen production from seawater by selectively permitting water vapor transport while blocking salt penetration. Enhanced dual osmotic/vapor-pressure gradients achieve a hydrogen output of 52.7 kg day^{-1} m^{-2}, quadrupling conventional hydrophilic membrane performance while maintaining system durability. Thermal self-circulation utilizing electrolyzer waste heat via dual heat exchangers eliminates external energy inputs, reducing energy consumption to 47.8 kWh kg^{-1} while co-generating purified water. This integrated approach synergistically addresses renewable hydrogen scalability and freshwater scarcity with efficiency rivaling pure-water electrolysis systems. Therefore, I recommend publishing this manuscript after minor revisions. Below is a list of specific comments and questions the authors should address before publication.

1.Will the surface structure and micro-pore structure of the PTFE membrane gradually undergo irreversible changes in multiple long-term cycles? How to evaluate its lifespan and regeneration performance?
2.In the current research system, apart from Ni-based alloy electrodes, how does the author consider the diversity of electrode materials and the compatibility with the membrane?
3.Will the microorganisms, organic pollutants or colloids existing in real seawater affect the membrane flux or cause pollution? Is additional pretreatment necessary?
4.How does the author consider the scale-up of system integration, such as the increase in membrane area, and whether it will bring new problems in heat transfer, mass transfer, etc.?
5.It is recommended to provide the full name of OMD upon its first appearance in the abstract.

Reviewer #3

(Remarks to the Author)
This manuscript proposed a membrane-integrated seawater electrolysis process of OMD-AWE to produce hydrogen with water electrolysis in alkaline condition, addressing the challenge in H2 production from non-freshwater sources. They provide that the integrated design uses only 0.4–1.1 kWh m–3 for water purification, and maintains energy efficiency comparable to commercial pure-water electrolyzer. This new process is interesting due to the utilization of waste heat from AWE processes; however, some critical issues have to be classified to improve its novelty.
1) It is difficult to find significant progress with that a direct seawater electrolysis method for hydrogen production (Nature volume 612, pages673–678 (2022)), except for the harnessing waste heat from AWE for thermal self-circulation.
2) Comparing with RO process for desalination, MD and OMD processes can save large energy undoubtedly due to the use of porous hydrophobic membranes. Despite many advantages in membrane technology, challenges remain for MD's large-scale industrial adoption, including high energy consumption, flux decline due to fouling, and economic feasibility. (Water Air Soil Pollut (2025) 236:24)
3) This manuscript has completed a continuous 72 h test of OMD-AWE treating real seawater to demonstrated stable performance, the results is not enough to proof its durability, especial for biofilm fouling with seawater which usually make the original hydrophobic membrane become hydrophilic one.
4) The flow rate and heat balance need to be evaluated for OMD-AWE system, heat flux from conduction between the hydrophobic membrane, and that from heat exchanger before electrolyzer.

Reviewer #4

(Remarks to the Author)
The manuscript demonstrates that a hydrophobic PTFE-based osmotic membrane–distillation (OMD) module, thermally coupled to an alkaline water-electrolysis (AWE) stack, delivers a record hydrogen flux of 52.7 kg $day^{-1} m^{-2}$ while rejecting salts almost completely. Although this flux greatly exceeds that of prior PTFE/OD systems, the advance appears incremental rather than conceptual because (i) PTFE-mediated vapor-phase desalination is well established; (ii) recovery of low-grade electrolyser heat has already been modelled; and (iii) no techno-economic, life-cycle, or >100 h durability data are provided to show competitiveness against RO + AWE or FO-AWE at scale. Consequently, the novelty and impact fall short of the usual Nat Commun Communication standard. The specific issues below, if addressed, could significantly strengthen the work.

1) Clarify the genuine transport advance (pp. 11–13) — Heating the feed to raise ΔP_v in PTFE-OMD is intuitive and widely practised in membrane-distillation desalination. The manuscript should identify any new physical insight beyond this well-known strategy.

2) Provide a full techno-economic comparison — Include levelised cost of hydrogen (LCOH), CAPEX/OPEX breakdowns, and life-cycle (LCA) metrics versus (i) OMD-AWE, (ii) FO-AWE (Nat Commun 15, 2617, 2024), (iii) RO + EDI + AWE, and (iv) direct seawater electrolysis. Recent MD-AWE TEA work already reports 1.3 US\$ kg^{-1}-H_2; the present study must benchmark against this figure.

3) Demonstrate long-term stability — The current 72 h test is insufficient. Please provide ≥500 h continuous operation on real seawater, tracking water flux, cell voltage, Faradaic efficiency and post-mortem SEM/EDX of both membrane and electrodes to substantiate durability claims.

4) Quantify brine management and recovery — Report overall water-recovery ratio, brine salinity (>70 g L^{-1}) disposal strategy, and associated energy/CO_2-equivalent burden.

5) Membrane wetting resistance (Fig. 3b–d) — Supply the liquid-entry pressure (LEP) of the PTFE membrane at 40 °C and discuss the impact of surfactants/organics present in natural seawater.

6) Thermodynamic reference data (Fig. 4b) — Add the water-vapour pressure of 1 M KOH at 20 °C to aid comparison.

7) Ensure consistent units and notation — Use SI units (kg $m^{-2} h^{-1}$) throughout; if “LHM” is retained, define it on first use.

8) Disclose raw electrochemical data — Provide full E–I curves for the five-cell AWE stack in the Supplementary Information.

9) Diagnose initial voltage rise (Fig. 4e) — Identify whether the early-stage degradation originates in the OMD-to-AWE integration (e.g., temperature, pressure, electrolyte dilution) or within the AWE stack itself.

10) Justify the choice of 1 M KOH — Because conventional AWE commonly operates at higher KOH concentrations for improved conductivity, explain the rationale for selecting 1 M despite the likely performance penalty.

11) Foot-print versus productivity — Provide a quantitative assessment of system foot-print as a function of hourly H_2 output and water-recovery ratio. OMD’s inherent low recovery may demand large membrane areas; please evaluate this at practical scales.

12) Interpretation of the Surplus Water Index (SWI) — While a high SWI signals good thermal-integration potential, it does not guarantee that OMD water flux meets electrolyser demand. Clarify how SWI interacts with actual flux requirements and recovery ratios in sizing the membrane module.

13) Define all acronyms at first mention (e.g., “LHM”, “SEC”).

Reviewer #5

(Remarks to the Author)
I co-reviewed this manuscript with one of the reviewers who provided the listed reports. This is part of the Nature Communications initiative to facilitate training in peer review and to provide appropriate recognition for Early Career Researchers who co-review manuscripts.

Version 1:

Reviewer comments:

Reviewer #1

(Remarks to the Author)
Thank you very much for your detailed responses, which have addressed most of my concerns and clarified my questions. I

appreciate the careful effort and time you have invested in providing such thorough explanations. The previously misleading phrasing has been revised, and the current description of the innovation is more accurate and appropriately reflects the novelty of the work. However, I still have a few remaining questions, for which I would sincerely appreciate further clarification:

1. Since I am not a specialist in water treatment, my understanding is based on the literature I have reviewed. In most publications, the flux is typically reported in units of LMH. Could you please confirm whether the notation LHM is appropriate in your context?

2. Thank you very much for providing the detailed experimental procedures. Given that the active area of your OMD module is 1 cm^2 and the reported flux is 20 LMH, the corresponding permeate production rate is approximately 0.033 mL min^{-1}. Considering that your AWE system consists of a five-cell stack, I would like to inquire about the approximate water feed rate for AWE. Specifically, is the permeate production of the OMD module sufficient to sustain the water demand of the stack?

3.In addition, the IV curve is commonly referred to as the polarization curve. Thank you very much for providing the additional electrochemical characterization data. These measurements are highly valuable for assessing and interpreting the performance of the cell.

4.Could you please elaborate further on the thermal management strategy implemented in your system? I am particularly interested in whether the outlet water temperature of the AWE (reported as 60 °C) can be maintained at approximately the same temperature upon reaching the inlet of the heat exchanger. In typical laboratory setups, even when the reservoir is heated to 100 °C, the water temperature at the cell inlet often decreases to around 60 °C even with thermal insulation applied along the flow path, due to unavoidable heat losses.

5.Since the focus of the manuscript has been shifted to a thermally integrated system, I would like to suggest including more discussion on the utilization of thermal energy. Providing additional details or analysis on how heat is recovered, distributed, and applied within the system would strengthen the manuscript and better highlight its relevance and impact. Specifically, I would appreciate more information regarding the design of the heat exchanger. The reported heat exchange efficiency appears to be exceptionally high, and I am curious about the specific design features that enable such effective heat transfer.

6.In Figure 1, regarding Heat Exchanger 2, could you please clarify the outlet temperature of the cold stream? Is it possible for it to be heated up to 60°C, since it flows directly into the AWE after passing through the heat exchanger? This seems unlikely, as theoretically the cold stream temperature should not exceed that of the hot stream.

Reviewer #2

(Remarks to the Author)
In this article, the authors reported a thermally symbiotic osmotic membrane distillation-electrolysis (OMD-AWE) system for direct hydrogen production from seawater. The work featured a clever thermal management design that converted waste heat from the electrolysis process into the driving force for membrane distillation, simultaneously addressing water supply and system cooling challenges. The concept was novel and held significant application potential. However, the mechanistic explanations at the material level and the experimental support for key conclusions remained relatively weak. Therefore, the manuscript could be reconsidered for publication Nature communication after major revision.

Q1: The authors should conduct an in-depth analysis and clarify the impact of fluctuating potentials in practical operating environments—such as those caused by the intermittency of renewable energy power supply—on the system's stability. The current study was conducted under constant current/potential conditions, which does not align with the actual production scenarios for most green hydrogen. The referenced article (Nature. 2025, 639, 360–367. and Nat. Sustain. 2025, https://doi.org/10.1038/s41893-025-01665-y.) elucidated the corrosion issues associated with potential fluctuations in seawater electrolysis. Consequently, evaluating the system's performance degradation, membrane wetting risks, and electrode corrosion behavior under dynamic potential conditions is of critical importance.
(1) If intermittent square or sawtooth wave potentials simulating renewable energy sources (such as photovoltaic power generation) are applied, what observable degradation would occur in the system's hydrogen production Faraday efficiency, water flux across the membrane module, and ion rejection rate?
(2) Potential fluctuations may accelerate passivation/dissolution of electrode surfaces or cause localized overheating of PTFE membranes. Please provide corresponding experimental evidence or theoretical simulations to demonstrate the chemical and physical stability of the system's core materials (Ni/NiFe electrodes and PTFE membranes) under dynamic operating conditions.
(3) Please discuss whether your thermal-water-hydrogen model possesses the capability to incorporate fluctuating potential as an input parameter and predict system performance (such as hydrogen production rate and thermal balance). If not, how should the model be improved to accommodate such practical operating conditions?

Q2: The authors attributed the flux decline to scaling, but the microstructural evolution of the PTFE membrane itself under long-term operation remains unclear. Could the authors comment on whether any irreversible alterations in polymer morphology—such as changes in crystallinity, pore size distribution, or surface roughness—were observed following the 500-hour test? Additional characterization data, for instance from XRD, BET analysis, or AFM, would be invaluable in clarifying the long-term structural stability of the membrane material under these operating conditions. This reference (J. Am. Chem. Soc. 2025, 147, 28, 24604–24614.) can be referred to.

Q3: The excellent ion rejection data confirms that the OMD process successfully isolates the electrolyser from bulk chloride

and bromide. However, have you considered or detected the potential for volatile chlorine species (e.g., Cl_2, HOCl) to form at the anode and subsequently permeate back through the PTFE membrane as gases? Such reverse transport could potentially re-contaminate the feed side and, more critically, induce chemical degradation of the PTFE membrane itself upon long-term exposure. Please comment on this potential risk and any relevant experimental observations. These references (J. Am. Chem. Soc. 2024, 146, 8, 5324–5332. and Nat. Commun., 2023, 14, 4822.) can be referred to.
(1) Given the system's closed-loop nature, did your gas chromatography analysis of the produced oxygen stream detect any trace amounts of chlorine gas (Cl_2) or other volatile chlorinated species?
(2) Following the long-term test, was NMR analysis performed on the PTFE membrane to confirm whether any chemical degradation, such as defluorination or structural rearrangement, occurred? Such evidence would be crucial to verify the occurrence of chemical attack under operational conditions.

Q4: The manuscript highlights the system's high hydrogen production flux. To better position this OMD-AWE technology within the existing landscape, could the authors provide a comparative analysis with state-of-the-art direct kW-scale seawater electrolyzers? A discussion focusing on key metrics such as system complexity, stability under real seawater feedstock, and the projected balance-of-plant requirements would be highly informative for assessing its practical competitiveness. These references (J. Am. Chem. Soc. 2025, 147, 20, 17190–17200. and Nat. Commun. 2024, 15, 10351.) can be referred to.

Q5: Safety is a paramount concern for electrolysis systems. The integrated system produces hydrogen and oxygen streams simultaneously. Could the authors please comment on the potential risk of hydrogen crossover into the oxygen stream? Specifically, have you measured the hydrogen-in-oxygen concentration, and does it remain well below the lower explosion limit (LEL) of 4% throughout the 500-hour operation? This is critical for assessing the system's operational safety and the adequacy of the gas separation design. This reference (Nat. Rev. Clean Technol. 2025, 1, 380–395.) can be referred to.
(1) Could you provide the actual gas chromatography data for both the hydrogen and oxygen streams, specifying the measured concentration of H_2 in O_2 and O_2 in H_2?
(2) Beyond the diaphragm in the AWE stack, does the PTFE membrane in the OMD module pose any risk of gas crossover between the seawater and KOH loops? Please evaluated its permeability to H_2 or O_2 gases under operational conditions
(3) The gravity-based degas tower is mentioned as the key gas-liquid separation unit. Could you elaborate on its design (e.g., dimensions, internals) and provide evidence of its separation efficacy? Specifically, how does it ensure that entrained electrolyte droplets containing dissolved H_2 or O_2 do not lead to gradual gas crossover in the recirculating loops?

Q6: From a technology development perspective, could the authors please provide an outlook on the potential and challenges of integrating emerging hydrophobic materials (such as carbon nanotube membranes, graphene membranes, or organic-inorganic hybrid membranes) into the proposed system? Specifically, how might these advanced materials enhance water flux and antifouling performance, and what would be the key compatibility considerations (e.g., thermal stability under 40–60°C operation, chemical resistance to 6 M KOH, and mechanical integrity under flow conditions) for their practical implementation in this thermally symbiotic OMD-AWE system?

Q7: The unique "self-dampening" operating environment, which provides a continuous supply of ultra-pure water, likely creates distinct catalyst working states compared to conventional electrolyzers. To uncover these differences, what in-situ/operando characterization techniques (e.g., in-situ Raman or X-ray absorption spectroscopy) could be employed to directly observe the dynamic surface structure and valence state of the NiFe anode and Ni cathode under actual operating conditions? Understanding this real-time dynamic process is crucial for custom-designing the most compatible and efficient catalysts specifically for this OMD-AWE system. These references (J. Am. Chem. Soc. 2025, 147, 44, 40807–40815. and Nat. Energy. 2025, 10, 880–889.) can be referred to.

Q8: The author mentioned that “Scaling behaviour was monitored over three consecutive cycles of 7 h each”. For assessing industrial viability, this limited number of cycles is insufficient to evaluate long-term durability. It is critical to provide:
(1) It is imperative to provide data on water flux recovery rates across a substantially higher number of fouling-cleaning cycles (e.g., 10-20 cycles). Such an extended dataset is required to establish a definitive trend regarding the membrane's regenerative performance and long-term resistance to repeated scaling.
(2) A critical discussion is needed on whether the regeneration protocol, particularly with EDTA, induces any cumulative damage to the PTFE membrane's intrinsic properties.

Q9: The AWE utilized in this work, while robust, is typically limited to lower current densities compared to advanced AEM or PEM electrolyzers. Given that your OMD system demonstrates an exceptionally high water flux capable of supporting intense reactions, have you considered its integration with higher-current-density electrolyzers like AEM or PEM? What would be the key system-level adaptations required, particularly regarding thermal management (for PEM's lower operating temperature) and the chemical compatibility of the PTFE membrane with acidic (PEM) or low-alkalinity (AEM) environments? These references (Nat. Energy. 2024, 8, 264–272. and Nat. Catal. 2024, 7, 944–952.) can be referred to.

Q10: The author mentioned that “The manuscript clearly states that EDTA was dosed (0.5% w/v) into the seawater feed during the 500-h longevity test”. The use of EDTA in the real seawater experiments, while practical for demonstrating long-term operation, potentially masks the inherent anti-fouling performance of the PTFE membrane and the true fouling challenges in natural environments. To accurately assess the system's robustness, the following clarifications are essential:
(1) Please clarify the duration for which the OMD-AWE system can operate stably using real seawater without the addition of any antiscalants or cleaning agents. This would establish a baseline for the membrane's intrinsic fouling resistance and inform the required frequency for maintenance cleaning in a practical scenario.
(2) The fouling analysis primarily identified inorganic scaling. However, real seawater contains a complex mixture of natural

organic matter (NOM), such as humic acids, and inorganic salts. Was there any evidence of synergistic fouling, where NOM and inorganic ions (e.g., Ca^{2+}, Mg^{2+}) co-precipitate or form a mixed organic-inorganic foulant layer on the membrane surface that is more resistant to cleaning?

Q11: The PTFE membrane is subjected to a consistent ~20°C temperature gradient across its structure during operation. While PTFE is known for its thermal stability, the long-term effect of this steady-state thermal gradient on the membrane's mechanical integrity requires further discussion.
(1) Could author please discuss the potential for thermomechanical stress buildup within the membrane structure? Specifically, could the thermal expansion coefficient mismatch between the PTFE polymer and any potential reinforcing materials (or between the membrane and its module housing) lead to issues such as creep or delamination over extended operation?
(2) Furthermore, is there any post-mortem evidence from the 500-hour test—such as visual inspection, SEM imaging of the membrane cross-section, or records of seal failure—that indicates physical deformation (e.g., warping) of the membrane or a loss of integrity at the sealing interfaces? Empirical evidence is crucial to rule out these potential failure modes.

Reviewer #3

(Remarks to the Author)
This revised manuscript reconsidered the merits and limitation for a membrane-integrated seawater electrolysis process of OMD-AWE to produce hydrogen with water electrolysis in alkaline condition, addressing the challenge in H2 production from non-freshwater sources. Their research provides a flexible design framework, enabling the OMD and AWE at any configuration while determining optimal operational windows for thermal self-sufficiency at any scale, moreover, the results showed a record-high H2 production rate with running over 500 h with direct seawater electrolysis.
One point needs to clarify that the influence of ocean life upon PTFE membrane fouling due to hydrophilic species, which usually changes surface hydrophobic properties and reduces desalination in OMD. In generally, comparing with the original version, the revised manuscript has obtained obvious improvement.

Reviewer #4

(Remarks to the Author)
The authors have significantly improved the manuscript in response to the reviewers' comments.
The additional experiments, long-term operation, and expanded TEA/LCA analysis strengthen the robustness and practical relevance of the work.
However, I remain unconvinced that the study delivers a sufficiently fundamental conceptual advance for Nature Communications.
In my view, the main contribution lies in a careful system-level integration and benchmarking of already known elements—alkaline water electrolysis, osmotically driven membrane distillation, thermal coupling, and low-temperature/low-recovery operation—rather than in a genuinely new physical or chemical principle.
The three "counter-intuitive" design guidelines emphasized by the authors appear to formalize trade-offs (between temperature, fouling/wetting, and recovery) that are already qualitatively recognized in the MD and electrolysis communities. While this does not diminish the practical value of the proposed configuration, it does limit the degree of novelty, and I would characterize the advance as incremental rather than transformative.

Reviewer #5

(Remarks to the Author)
I co-reviewed this manuscript with one of the reviewers who provided the listed reports. This is part of the Nature Communications initiative to facilitate training in peer review and to provide appropriate recognition for Early Career Researchers who co-review manuscripts.

Version 2:

Reviewer comments:

Reviewer #1

(Remarks to the Author)
I would like to acknowledge that the authors have provided a very comprehensive response and have made detailed revisions to several technical aspects of the manuscript. The effort put into the updated manuscript and the clarity of certain experimental sections is evident.
However, regarding the core concept of the "thermally symbiotic" system, I find the current presentation somewhat misleading. The "Thermal Symbiosis" appears to be a theoretical projection rather than an experimentally validated achievement in this study. In the Abstract and Introduction, the authors describe a "thermally symbiotic system" that operates stably for 500 hours. Yet, in the Response Letter, it is admitted that heat loss was significant and that waste heat utilization was not actually realized in the experimental setup, relying instead on modelling simulations. This creates a discrepancy with the revised manuscript, which states that "Both pipes and heat exchangers are wrapped with aluminium-clad polyurethane foam insulation, resulting in negligible heat losses."

Please revise the title or the main text to clarify that the proposed "thermally symbiotic system" is a result of numerical modelling, rather than an experimental result of the 500-hour operation. Furthermore, if the primary novelty of this work lies in the "thermally symbiotic" system derived from thermodynamic calculations, I believe the current elaboration is not sufficiently profound or comprehensive. The authors might consider expanding this section to provide a more in-depth discussion.

Reviewer #2

(Remarks to the Author)
All the issues have been resolved, so it is recommended to accept.

Reviewer #3

(Remarks to the Author)
This study investigated a thermally symbiotic osmotic membrane distillation-electrolysis (OMD-AWE) system for direct hydrogen production from seawater, and made 500 hours measurement actually. For a real application, it brings novel technology definitely; however, on the view of academic advance, this is only incremental rather than transformative progress. A similar program has been published as seen in Nature 612, 673–678 (2022), titled a membrane-based seawater electrolyser for hydrogen generation, in which a demonstration system was stably operated at a current density of 250 milliamperes per square centimetre for over 3,200 hours under practical application conditions, without any thermal energy input and no biofouling and extracellular polymeric substances (EPS) accumulation. At least, more durable pilot test over 3,200h may support its real availability.

Reviewer #4

(Remarks to the Author)
The authors have properly revised the manuscript in response to the reviewers’comments.

Reviewer #5

(Remarks to the Author)
I co-reviewed this manuscript with one of the reviewers who provided the listed reports. This is part of the Nature Communications initiative to facilitate training in peer review and to provide appropriate recognition for Early Career Researchers who co-review manuscripts.

Version 3:

Reviewer comments:

Reviewer #1

(Remarks to the Author)
The revisions made to the manuscript are clear and well-justified, and the authors have responded in a thorough and professional manner. I have no further comments at this stage and am pleased to recommend the manuscript for acceptance in its current revised form. I would like to thank the authors for their time and effort in preparing the detailed response and for the quality of the revised work.

Reviewer #3

(Remarks to the Author)
I agree the point of view that an osmotic membrane distillation need continuous heat energy input, and it is exactly the most critical limitation for MD without thermal energy supply. A system simulation is also useful for promoting its engineering proceeding of OMD-AWE to hydrogen production with direct seawater electrolysis in alkaline condition. Although that, I have reviewed this manuscript many times, and find almost no obvious change of manuscript's novelty. I believe that it is difficulty to improve its novelty only based on the idea of thermally symbiotic integration of osmotic membrane distillation process. For this reason, this manuscript maybe suitable for professional journals due to the lack of conceptual framework innovation to meet the requirement of Nature Communications publications.

Open Access This Peer Review File is licensed under a Creative Commons Attribution 4.0 International License, which permits use, sharing, adaptation, distribution and reproduction in any medium or format, as long as you give appropriate credit to the original author(s) and the source, provide a link to the Creative Commons license, and indicate if changes were made.
In cases where reviewers are anonymous, credit should be given to 'Anonymous Referee' and the source.
The images or other third party material in this Peer Review File are included in the article's Creative Commons license, unless indicated otherwise in a credit line to the material. If material is not included in the article's Creative Commons license and your intended use is not permitted by statutory regulation or exceeds the permitted use, you will need to obtain permission directly from the copyright holder.
To view a copy of this license, visit https://creativecommons.org/licenses/by/4.0/

Former Title: In situ co-production of hydrogen and clean water from seawater via osmotic membrane distillation-integrated electrolysis

Revised Title: Thermally symbiotic osmotic membrane distillation-electrolysis unlocks efficient and stable hydrogen production directly from seawater

Authors: Gabriela Scheibel Cassol, Chii Shang, Paul Westerhoff, Yinghao Song

Manuscript ID: NCOMMS-25-36631

Before responding to the comments of the reviewers, the authors would like to express our deepest appreciation for their time and effort in reviewing this manuscript.

Responses to comments of Reviewer 1:

This manuscript presents an evaluation of two membrane-based water purification technologies—osmotic dilution (OD) and osmotic membrane distillation (OMD)—integrated with alkaline water electrolysis (AWE) to enable hydrogen and clean water production. The study explores the use of PTFE and TFC membranes in these systems and introduces a thermal self-circulation strategy utilizing waste heat from the electrolyzer. The integration of membrane technologies with electrolyzers—particularly the reuse of electrolysis waste heat to enhance system efficiency—is a timely and important direction that remains underexplored. While the concept is promising, the current version of the manuscript lacks sufficient technical clarity and does not clearly differentiate its innovations from existing work. In addition, several methodological concerns need to be addressed. With substantial reorganization, a more focused presentation, and clearer articulation of novelty—particularly in the title and abstract—the manuscript could be considerably strengthened. However, at this stage, I do not recommend the manuscript for publication in Nature Communications.

Reply:

We sincerely thank the reviewer for the constructive evaluation of our manuscript. We agree that the original version lacked sufficient technical clarity and did not adequately differentiate our innovations from existing work. The reviewer's insightful feedback has been valuable in guiding a substantial reorganization of the manuscript to address these critical issues. In response to the reviewer's comments, we have undertaken extensive revisions, with over 80% of the original text being rewritten to improve the manuscript's structure, focus, and clarity. The key improvements include: i) a clearer demonstration of our novelty about the thermal symbiosis design, ii) a more focused analysis for MD systems, iii) demonstrated long-term stability, and iv) enhanced technical clarity and details.

Given the extensive revisions, we have provided concise summaries of the key changes in our point-by-point response. For detailed explanations and the new supporting data, we respectfully refer the reviewer to the full revised manuscript, where all major changes have been highlighted in blue. We are confident that these revisions have addressed the reviewer's concerns and have transformed the manuscript into a much stronger, more focused, and impactful contribution.

Comment 1: Limited novelty

Although the manuscript highlights PTFE membranes and the OMD process as key innovations, these concepts are well-established. The hydrophobic PTFE membrane has been widely used in membrane distillation for decades and is a mature material in the water purification field. The description in the abstract may inadvertently mislead readers into perceiving it as a novel or advanced material.

Similarly, the OMD technique itself has been studied for over a decade. As presented, the OMD system in this work does not appear to deviate significantly from previous OMD designs. The manuscript repeatedly emphasizes the novelty of the system, particularly in lines 218–229, but it is difficult to identify a unique technical advancement from this description. I recommend the authors provide a clear schematic and detailed discussion of the specific design features that distinguish their OMD implementation from existing methods.

Reply:

We thank the reviewer for these insightful comments for providing us with the opportunity to clarify the novelty of our work. We agree that PTFE membranes and the OMD process are well-established technologies while our previous description may mislead readers into thinking these components are novel in themselves. The core innovation of our work lies in the precisely designed thermal symbiosis between these established components, creating a system that solves a critical, previously unresolved bottleneck in the field of membrane-integrated electrolysis, allowing it to achieve performance far beyond what was previously possible.

The current state-of-the-art in situ seawater electrolysis system reported in *Nature* 612, 673–678 (2022) combines isothermal OD with AWE in a physically coupled design for simultaneous water purification and electrolysis[1]. While its vapor transport mechanism through hydrophobic PTFE membranes ensures complete salt rejection, its limited osmotic driving force results in insufficient water production that constrains H_2 output. More critically, this direct coupling creates inherent constraints for optimization and scaling, as optimal conditions for PTFE's water vapor transport and AWE are not allowed. For instance, AWE can only operate at room temperatures rather than its optimal 60–90°C, since increasing the electrolyte temperature leads to reverse vapor transport due to higher vapor pressure than feed. Pressurized H_2 production is also impossible as operating above PTFE membrane's liquid entry pressure (LEP, 200–500 kPa[2]) causes membrane wetting and compromises salt rejection. Although heating the feed to introduce a thermal gradient can enhance water flux, this approach requires 17–70 times more energy than conventional reverse osmosis (RO) processes. Consequently, scaling up this system for real world application would require substantial increases in membrane areas and energy input, creating significant barriers to economic viability. **Our key innovation is a thermal integration design** that physically separates the PTFE membrane module from the AWE electrolyzer while maintaining their thermal connection for thermally symbiotic operation. This thermal integration converts AWE's waste heat, which is an operational burden requiring costly cooling, into a valuable asset that drives water vapor transport through OMD using the PTFE membrane. In return, the OMD provides the AWE with both passive cooling and a continuous supply of high-purity water. This allows both OMD and AWE to operate under their respective optimal conditions simultaneously, without over-pressure and reverse flux issues. Due to the enhanced water production and the AWE's optimal temperature operation, this system delivered record-high 60.2 kg day^{-1} m^{-2} of H_2 production rate that is 25 times higher than that from the Nature paper, while maintaining stability for 500-h continuous operation with

superior energy and cost metrics. We have revised the manuscript, particularly the **Introduction and Abstract**, to ensure we do not mislead readers and demonstrate our core innovation.

The reviewer's second comment is also constructive in highlighting the need to differentiate our OMD implementation. We have added a dedicated discussion to demonstrate how our thermally symbiotic design relies on a series of "counter-intuitive" operational principles that reverse the conventional wisdom of standalone water purification.

1. A milder 40°C operational temperature is better. Conventional MD-based water systems follow a "hotter is better" principle, typically operating over 60°C to maximize water flux and water recovery[3–5]. However, such high temperatures increase membrane scaling, wetting risks, and potential salt crossover. While these risks are manageable in drinking water production, where certain levels of benign salts are acceptable and temporary shutdowns for membrane maintenance are feasible, the requirements change when purifying water for electrolyzer that has zero tolerance for salt. Even a minor wetting event that would be manageable in water treatment can introduce sufficient salt to cause irreversible damage to the electrolyzer through corrosion and catalyst degradation. Any cost saved by pushing for a higher water flux and recovery is paid for dearly in catastrophic damage to the far more expensive electrolyzer. This fundamental difference redefines optimal operating conditions where stability becomes as crucial as productivity. In this work, we identified 40°C OMD temperature for AWE integration, rather than pursuing maximum possible temperatures, maintaining sufficient water flux while operating safely below membrane failure thresholds. Supporting data from water flux measurements, LEP tests, and wetting/scaling tests confirming this 40°C temperature choice are detailed in **Revised Manuscript Lines 124–220** **"Thermal trade-offs between water flux and stability of PTFE membranes"**.

2. The heat source of OMD is from AWE. Another key feature of our OMD design is utilizing AWE's waste heat, unlike conventional OMD systems that require energy-intensive external heating. It is estimated that additional heating energy of 0.023 kWh is required for heating per kg of seawater from 20°C to 40°C, and another 0.833 kWh per kg of produced water is needed to maintain the thermal gradient against latent heat loss during vapor transport. Given that OMD's water recovery is limited to 10%[6,7], producing per kg of H_2 requires an additional 10.6 kWh of energy for OMD water production, corresponding to a 22.6% increase relative to the baseline 47.1 kWh kg(H_2)$^{-1}$ needed for water splitting. This makes OMD integration economically unfavourable. To overcome this energy penalty, we proposed a thermal self-circulation design that converts AWE's waste heat to drive OMD while simultaneously providing passive cooling for AWE. Our thermal modelling confirms that waste heat from commercial AWE stacks is sufficient to maintain thermal self-sustaining operation. By eliminating the external heating requirements, this design makes our seawater electrolysis system as energy efficient as pure water electrolysis. The detailed modelling and discussion about the use of AWE waste heat are provided in **Revised Manuscript Lines 340–462:** **Thermal self-circulation improves OMD-AWE energy efficiency**, and the related energy and cost analysis compared to other competing systems is presented in **Manuscript Lines 472–504: "Comparative techno-economic and environmental assessment**.

3. Low recovery as a performance enhancer. We highlight that reversing the conventional goal of high-water recovery is a strategic advantage that unlocks the system's full potential. Standalone desalination systems are constrained by the high costs of water pretreatment, extensive heating and pump energy, and brine disposal, forcing them to prioritize high recovery to be economically viable[8]. This locks them into a low-flowrate regime that is incapable of absorbing and productively using a large, low-grade heat source like an electrolyzer's waste heat. Our system design inverts this paradigm. By identifying the AWE's waste heat as the true limiting resource, we optimize for system-level energy efficiency instead of water resource efficiency. We therefore employ a low-recovery (<2.5%), high-flowrate loop that acts as a powerful heat transfer engine. This high flow rate efficiently captures the AWE's waste heat and creates a large thermal reservoir to maintain OMD's thermal driving force. This approach not only enables a higher, more stable flux with a smaller membrane footprint but also yields the critical secondary benefit of simplified, environmentally safe brine management. This new insight is discussed in **Revised Manuscript Lines 445–462.**

In addition, a clearer schematic showing the detailed thermal self-circulation loops is updated to the **Revised Manuscript Fig. 1.**

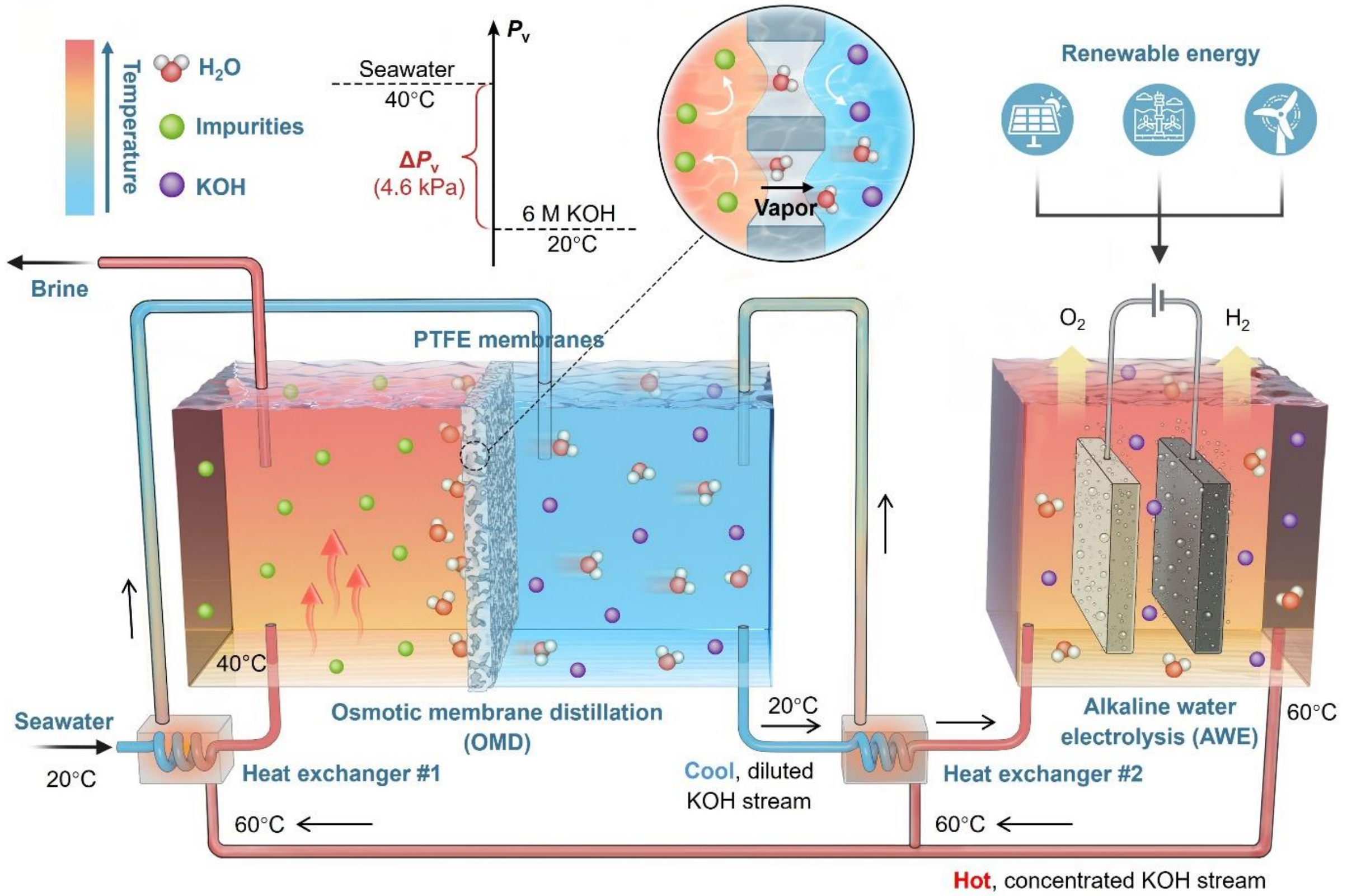

Fig. 1 Direct green H_2 production from seawater via a thermally symbiotic OMD-AWE system. The design utilises precisely designed self-sustaining heat exchange loops to convert the waste heat from AWE into the primary driver of the OMD process. This thermal integration enables OMD operation at an optimal 40°C feed with 6 M KOH as osmotic agent to deliver high

water flux with minimal fouling and wetting risks. In return, the OMD provides passive cooling and a continuous supply of pure water, enabling AWE to operate at its optimal 60°C for efficient H_2 production.

References:

1. Xie, H., Zhao, Z., Liu, T., Wu, Y., Lan, C., Jiang, W., Zhu, L., Wang, Y., Yang, D. & Shao, Z. A membrane-based seawater electrolyser for hydrogen generation. *Nature* **612**, 673–678 (2022).
2. Rácz, G., Kerker, S., Kovács, Z., Vatai, G., Ebrahimi, M. & Czermak, P. Theoretical and Experimental Approaches of Liquid Entry Pressure Determination in Membrane Distillation Processes. *Periodica Polytechnica Chemical Engineering* **58**, 81–91 (2014).
3. Lawson, K. W. & Lloyd, D. R. Membrane distillation. *Journal of Membrane Science* **124,** 1–25 (1997).
4. Kim, HW., Jang, A. & Jeong, S. Operational strategy preventing scaling and wetting in an intermittent membrane distillation process. *npj Clean Water* **6**, 50 (2023).
5. López-Porfiri, P., Ramos-Paredes, S., Núñez, P. *et al.* Towards the technological maturity of membrane distillation: the MD module performance curve. *npj Clean Water* **6**, 18 (2023).
6. Guillén-Burrieza, E., Zaragoza, G., Miralles-Cuevas, S. & Blanco, J. Experimental evaluation of two pilot-scale membrane distillation modules used for solar desalination. *J Memb Sci* **409–410**, 264–275 (2012).
7. Alquraish, M. M., Mejbri, S., Abuhasel, K. A. & Zhani, K. Experimental Investigation of a Pilot Solar-Assisted Permeate Gap Membrane Distillation. *Membranes* (Basel) **11**, 336 (2021).
8. Ullah, R., Khraisheh, M., Esteves, R. J., McLeskey, J. T., AlGhouti, M., Gad-El-Hak, M. & Tafreshi, H. V. Energy efficiency of direct contact membrane distillation. *Desalination* **433,** 56–67 (2018).

Comment 2: Concerns about AWE

The integration of OMD with AWE is an interesting concept, but the operating parameters raise significant technical concerns. For example, the applied current density of 1.17 A/cm2, derived from a water-hydrogen balance model (lines 244–249), is considerably higher than typical operational values for alkaline electrolyzers, which generally operate below 0.5 A/cm2. This high current could potentially accelerate degradation of the system.

Furthermore, the manuscript describes a 4.5% voltage increase within the first 24 hours (line 295) as minor, but such a rise in voltage over a short period could be indicative of rapid performance degradation, possibly due to impurities in the water or high current density. It would be helpful if

the authors could comment on whether any diagnostic techniques, such as EIS or polarization curves, were conducted to assess the performance of the AWE under these conditions.

It may also be worth discussing whether the water produced via OMD could be buffered or stored, decoupling the electrolysis step from water production, thereby avoiding the need for continuous operation at such high current densities.

Reply:

We sincerely thank the reviewer for raising this important concern regarding the AWE operating parameters. We agree that operating a standard AWE at a current density of 1.17 A cm^{-2} would lead to rapid degradation. We recognize that our manuscript caused confusion on this critical point, and we are grateful for the opportunity to clarify. The value of 1.17 A cm^{-2} presented in our work is not the electrochemical current density of AWE (i.e., current per electrode area). Instead, it is a system-level performance metric we have defined as the "membrane area-specific current (J_m)". This metric is calculated as the total AWE operating current divided by the PTFE membrane area, and its purpose is to normalize the H_2 production rate to the required water purification capacity. In this context, a J_m of 1.17 A cm^{-2} means that 1.17 A of total current is required to consume the amount of water produced by OMD with 1 cm^2 of PTFE membranes. The actual electrochemical current density in our AWE setup was 0.077 A cm^{-2} (calculated as the applied current of 1.17 A divided by the 15.2 cm^2 electrode area). This value is well within the safe and stable operating range for typical AWE systems and poses no degradation risk.

In the revised manuscript, we have changed the 1 M KOH electrolyte to 6 M KOH as suggested by our reviewers. This increases J_m to 1.34 A cm^{-2} when we used 0.5 NaCl feed to test its H_2 production rates. This corresponds to an AWE current density of 0.088 A cm^{-2}. For the 500-h long-term test, J_m is 1.2 A cm^{-2} due to seawater's higher salinity, and thus the AWE current density is 0.079 A cm^{-2}. Both remain safely below typical AWE operating limits. To avoid misunderstanding, we have added the current density (0.079 A cm^{-2}) in **the Revised Manuscript Fig. 3c**, while to ensure this distinction between the system-level J_m and actual electrochemical current density is clear to readers, we have added the clarification in **the Revised Manuscript**:

Lines 245–253: "To describe this system-level relationship, we define a new metric, i.e., membrane area-specific current (J_m), which is the AWE current required per unit of PTFE membrane area to maintain the water balance. This metric is crucial as it governs the system-level integration between OMD and AWE and is fundamentally different from the electrolyser's current density (i.e., current per electrode area), which is a component-level metric that describes electrochemical reaction rates at the catalyst surface."

We also thank the reviewer for the concern about 4.5% voltage increase at startup. We attribute this initial voltage increase not to permanent electrochemical degradation, but a temporary system-level instability during the startup and equilibration phase. This increase was likely because OMD temporarily produce water than the AWE consumes it during startup. This leads to a dilution of the KOH electrolyte, which in turn increases ohmic resistance and thus the operating voltage. Once

the system reaches its stable thermal and ionic equilibrium, the water production and consumption rates balance, and the voltage stabilizes. This trend is different from typical degradation behaviour which shows continuous voltage increase over time. This effect was particularly pronounced in our initial experiments in which we used a 1 M KOH. At this low concentration, the system's ohmic resistance is a bit more sensitive to small changes in dilution. In our revised manuscript, we have addressed this instability by operating with an optimal 6 M KOH which is not only more efficient but also less sensitive to the temporary system-level instability. This is proven by our extended 500-h stability test as shown in Fig. 3c. The cell voltage was stabilized at 1.72 V for the entire 500-hour duration. Other key performance metrics, including stable water flux and Faradaic efficiency >99%, also provides evidence supporting the excellent stability of our system. Analysis of the electrolyser electrodes after the 500-h test confirmed their pristine condition and complete protection from seawater contaminants. Both SEM and EDX revealed that the Ni nanowire cathode and NiFe anode retained their structural integrity and original elemental composition, with no surface fouling or traces of contaminants such as calcium, magnesium, or silica (Supplementary Figs. 6–7). While we did not perform EIS or polarization curve measurements, we believe the 500-h stability data provides the most compelling evidence that our system did not undergo degradation.

Details of the 500-h stability are added in the **Revised Manuscript Lines 285–314**. The supported SEM and EDX results are presented in **Supplementary Figs. 6–7**.

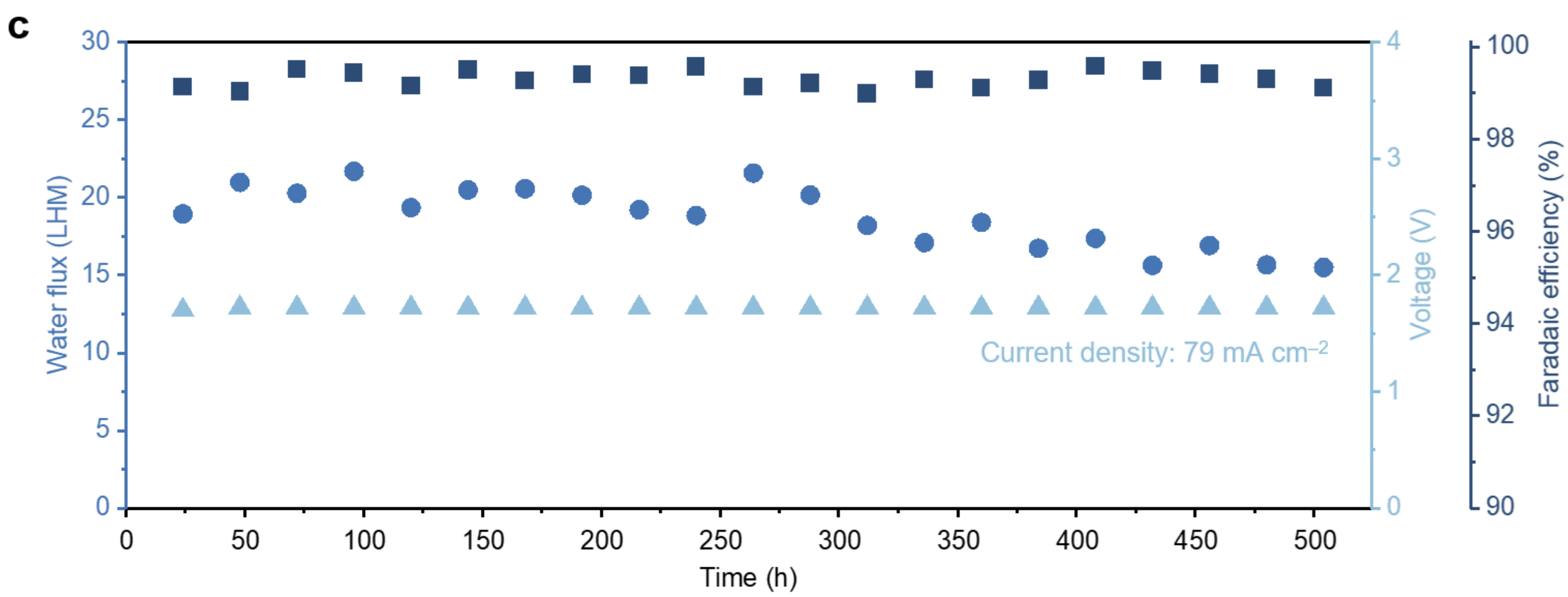

Fig. 3 c Water flux, cell voltage, and Faradaic efficiency over the 500-h continuous operation of OMD-AWE fed with real seawater at 40°C

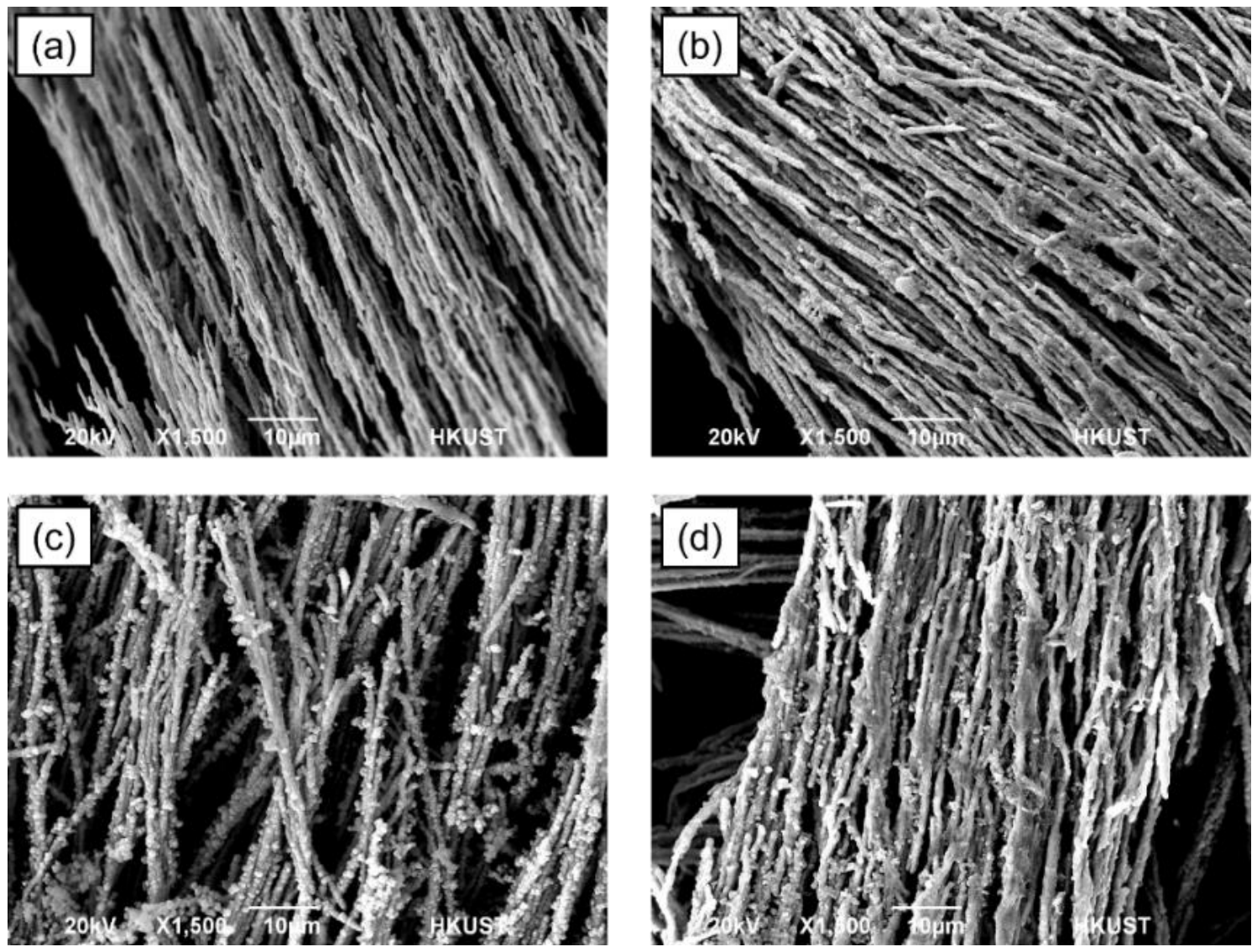

Supplementary Fig. 7 SEM images of the electrodes before and after the 500-h operation. a pristine Ni nanowire cathode; **b** Ni nanowire cathode after 500 h operation; **c** pristine NiFe anode; and **d** NiFe anode after 500 h operation

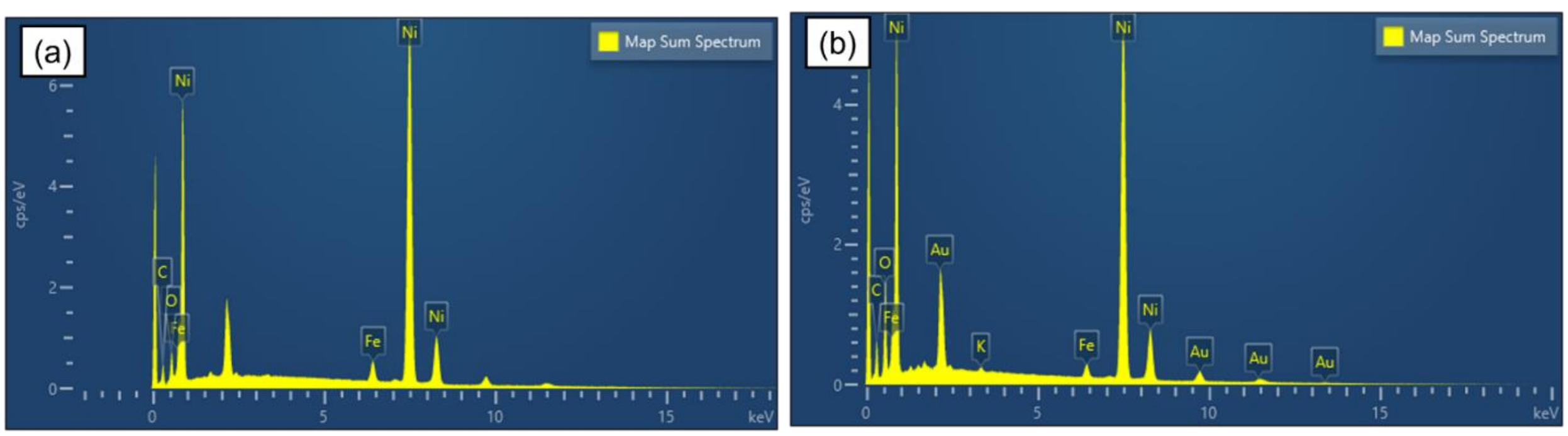

Supplementary Fig. 6 EDX spectrum of the electrodes. a the Ni nanowire cathode after 500-h operation; and **b** the NiFe anode after 500-h operation.

We thank the reviewer for the insightful comment regarding operational flexibility. This is indeed an advantage of our system. Our physically decoupled but thermally connected design provides the flexibility the reviewer suggests This modular architecture allows for either the OMD or AWE to be taken offline temporarily for routine maintenance (e.g., membrane cleaning) without requiring a complete system shutdown like seawater electrolysis reported in *Nature* 612, 673–678 (2022). When the unit is brought back online, the system is designed to automatically re-establish its thermal and ionic equilibrium. While there would be a short re-stabilization period, as discussed in our response, the system will find its new steady state and resume continuous operation.

These insights are added in the **Revised Manuscript**:

Lines 309–314: "In addition, our physically decoupled modular design provides crucial operational flexibility. This allows either the OMD or AWE to be taken offline for routine maintenance (e.g., membrane cleaning) without system failure. The ability to handle these

interruptions and return to stable operation is essential for robust, long-term operation beyond 500 h during real-world applications."

Comment 3: Framing and focus of the Introduction and Results part

While the authors aim to highlight the advantages of OMD over other methods, such as forward osmosis (FO) or reverse osmosis (RO), the Introduction devotes considerable space to reviewing FO and RO technologies. A more focused comparison with existing OMD or MD-based systems would be more appropriate and relevant to the manuscript's core claims.

Additionally, a substantial portion of the Results section focuses on performance of TFC membranes under OD conditions. While it is useful to illustrate the limitations of prior approaches, the level of detail dedicated to TFC membranes shifts attention away from the central contribution and may dilute the manuscript's focus.

A large portion of the discussions of mechanism in the Results section are not derived from the authors' experimental data, but rather looks like a literature review that would be more appropriately placed in the Introduction section.

Reply:

We thank the reviewer for these excellent suggestions about reconstruction of paper structure. We agree completely that our focus should be more aligned with the core claims regarding MD/OMD systems. The previous version provided an overly broad context on RO and FO, as well as some general MD mechanisms which are already well-established. Those diluted the focus.

We agree that TFC membranes shifts attention away from the central contribution. We have moved the FO water transport though TFC membranes and its related analysis on salt contamination to **Supplementary Note 1** and retained a concise sentence in **Introduction** to provide the necessary context as shown in the **Revised Manuscript**:

Lines 61–64: "However, its liquid-phase water transport mechanism threatens $FOWS_{AWE}$ stability because small amounts of salts cross FO membranes alongside water (Supplementary Note 1), which reduces osmotic driving force, degrades membranes, and compromises both water purification and electrolysis."

In addition, we have restructured the Results section to focus more directly on the comparison of OMD with existing MD-based systems and removed those MD mechanism discussions. After we proposed that a thermally driven MD system is necessary to overcome the water flux limitations of isothermal OD-AWE (*Nature* 612, 673–678 (2022)), but there is not study reported which MD configuration is most-suited for AWE integration, remaining a research gap. These include commonly used and well-established air gap MD (AGMD), direct contact MD (DCMD), and osmotic MD (OMD), which are distinguished by their permeate side by consisting of a gas layer, a water stream, or an osmotic agent, respectively[1–3]. Understanding their temperature-dependence

on the water flux, fouling, and wetting behaviour is critical on identifying the optimal integration design. Our work is the first to provide these new insights which are added to **the Revised Manuscript** **(Thermal trade-offs between water flux and stability of PTFE membranes)**:

Lines 122–163: "To identify the optimal thermal integration design for PTFE-AWE, we first investigated the effects of different MD configuration and temperature on the key performance metrics of PTFE membranes including water flux, wetting resistance, and fouling behaviours. The water flux tests were conducted using a 0.5 M sodium chloride (NaCl) feed solution at various temperatures up to 60°C against a permeate side maintained at room temperature (RT) of ~20°C, which consisted of either a 6 M KOH osmotic agent for OMD, deionised water for DCMD, or ambient air for AGMD, with the results compared in Fig. 2a. Water flux for all configurations increased with temperature due to the exponential rise in ΔP_v, confirming that thermally-driven MD enhances water production. OMD shows the highest water flux across all temperatures, e.g., at a representative temperature of 40°C, OMD achieved a water flux of 22.44 LHM, which was 34% higher than DCMD and over 4 times greater than AGMD. In standalone MD systems where external heating is a major cost, AGMD is preferred for its high thermal efficiency because its air gap serves as a thermal insulator that minimises conductive heat loss, despite water vapour slowly diffuses across it. However, AWE integration changes these priorities. The availability of free waste heat and high water demand for electrolysis make AGMD the least suitable option, while DCMD and OMD are the primary candidates which use a cold liquid permeate in direct contact with membranes to immediately condense water vapour for higher water flux.

Unlike DCMD entirely relying only on thermal gradients, OMD offers distinct advantages through its dual-driving-force mechanism. Through using a 6 M KOH solution as osmotic agent, OMD creates an additional, non-thermal 0.26 kPa vapour pressure depression on the permeate side, due to the reduced water activity. This supplementary force results in a water flux that is consistently over 5 LHM higher than DCMD under identical thermal conditions. This directly translates into two operational benefits. First, OMD can operate at a lower temperature to match DCMD's productivity (i.e., OMD produces 22.44 LHM water requires a feed temperature of 40°C whereas DCMD needs over 50°C), which reduces the risks of mineral scaling and membrane wetting. Second, the osmotic driving force makes OMD more resilient to thermal gradient loss caused by latent heat of evaporation. Each water molecule crossing the membrane must first absorb latent heat to vaporise at the hot feed surface, then release this heat upon condensing at the cooler permeate. This intrinsic heat transfer is not an inefficiency but rather the core engine of the process, as vapour transport inherently cools the feed while heating the permeate. This would cause both temperature polarisation at the membrane surface and axial temperature drop along its flow path (Fig. 2b), challenges that intensify with larger membrane areas and longer modules when scaling up the process. Although continuous reheating can compensate this heat loss, it is not an option for our AWE integration which relies on finite waste heat. This is precisely where the osmotic gradient becomes indispensable, providing consistent driving force even as thermal gradients weaken along the module. This osmotic backup ensures stable water flux, making OMD uniquely suited for

large-scale applications with limited or low-grade heat sources. Although OMD is overlooked for standalone water purification due to intensive energy consumption to regenerate its osmotic agent and separate the purified water, this issue is eliminated when integrated with AWE as the permeated water is directly consumed by electrolysis."

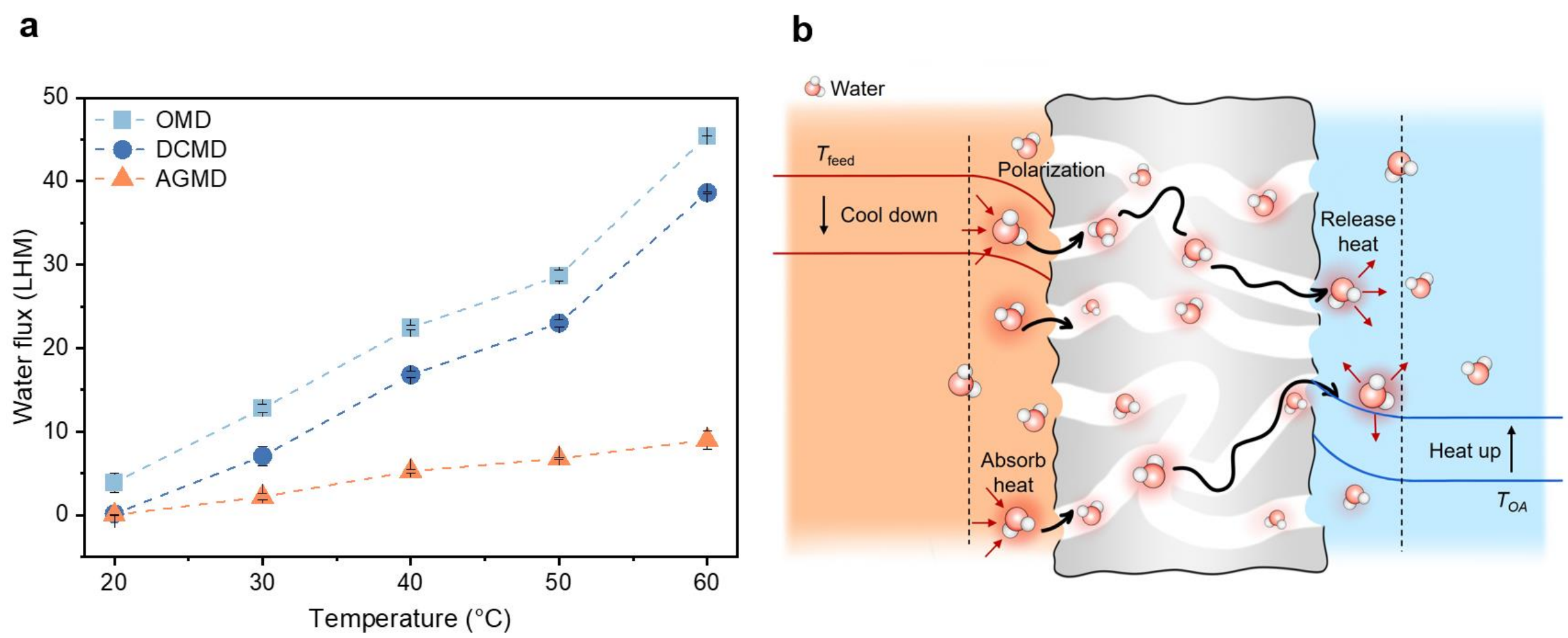

Fig. 2 a the comparison in water flux between OMD, DCMD, and AGMD at different feed temperatures; **b** a schematic showing thermal gradient loss from temperature polarisation at the membrane surface or axial temperature drop along its flow path due to latent heat transfer during water vapour transport.

References:

1. Yavuz, A. B., Karanikola, V., García-Payo, M. C. & Khayet, M. Osmotic distillation and osmotic membrane distillation for the treatment of different feed solutions. in Osmosis Engineering 245–278 (Elsevier, 2021).

2. Eykens, L., Hitsov, I., De Sitter, K., Dotremont, C., Pinoy, L. & Van der Bruggen, B. Direct contact and air gap membrane distillation: Differences and similarities between lab and pilot scale. *Desalination* **422**, 91–100 (2017).

3. Kakkar, R., Kaur, D. P. & Raj, S. Membrane Distillation for Sustainable Water Desalination: A Review of Principles, Materials, and Applications. *Water Air Soil Pollut* **236**, 24 (2025).

Comment 4: Missing experimental details and context

The manuscript reports impressive hydrogen production rates, but does not specify the actual membrane area or module configuration used in the OMD and OD setups. While the AWE stack itself appears to have an active area of 12 cm2 and 5 cells (lines 478–481). Considering the high current density applied, a substantial amount of water is needed, which in turn demands a high rate

of clean water production. Therefore, clarification of the membrane size and system scale of the OMD is essential.

Reply:

We sincerely thank the reviewer for this critical comment and for highlighting the need for more detailed experimental context.

These details of membrane module are added to our **Revised Manuscript Methods:**

Lines 555–561: "The membrane module comprises a custom-made cross-flow membrane cell featuring two symmetric chambers for feed and permeate separated by a single flat membrane sheet with an effective contact area of 1 cm^2 (1 × 1 cm). A thin-film composite (TFC) membrane (Porifera, 051303) was used for FO tests, while a PTFE membrane (0.45 µm pore size, 65 µm thickness) was used for MD/OD tests. For both feed and permeate sides, the respective feed and permeate solutions were recirculated from 1 L tanks through the cell by a peristaltic pump operated at a flowrate of 50 ml min^{-1}."

These details of our AWE stack are added to our **Revised Manuscript Methods:**

Lines 613–617: "The AWE electrolyser is a 5-cell alkaline stack (LBE-5SC, Light Bridge Inc.). Each cell contains series-connected bipolar nickel-based composite electrodes including a Ni nanowire-based cathode (4.4 cm diameter, JUNA TECH) and a NiFe-based anode (5.1 cm diameter, JUNA TECH). The two electrodes are separated by a 500 µm thick diaphragm (ZIRFON PERL UTP 500)."

We also tested our AWE stack's current-voltage (I-E) curve as provided in **Supplementary Fig. 3.**

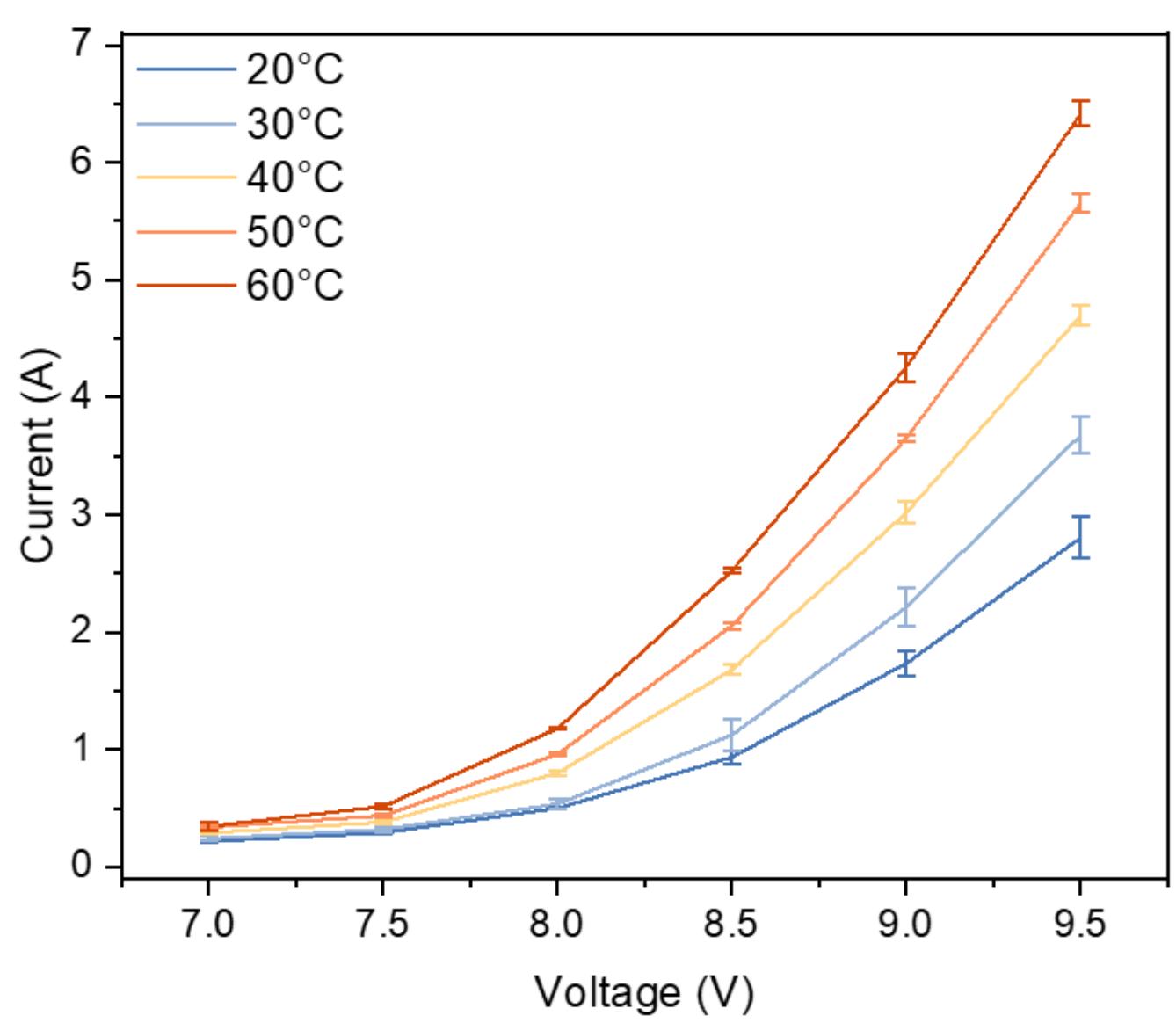

Supplementary Fig. 3. Current-voltage curves of our AWE electrolyser with 6 M KOH electrolyte at different operating temperatures.

The reviewer is correct about the AWE relies on the rate of clean water production to produce H_2, and this is the reason why the low water flux of the previous isothermal OD-AWE system limited its H_2 output. To match the water production via OMD and the water consumption via electrolysis, we described a water balance by a system-level metric J_m as mentioned in Comment 2. More details can refer to the **Revised Manuscript Line 234–268: Ultrafast and stable H_2 production via OMD-AWE integration,** where the revised contexts are highlighted in blue.

Due to the fixed geometry of our custom-made membrane cell (1 cm^2 area), the AWE operating current was used as the adjustable parameter to ensure a perfect water balance. For our optimized OMD configuration (40°C, 6 M KOH), the stable water flux of 22.44 LHM requires J_m of 1.34 A cm^{-2}, which means the required current for AWE is determined to be 1.34 A according to the 1 cm^2 membrane area. It is important to note that this approach is adaptable. In a different engineering scenario where AWE optimal current is usually fixed, our model could be used to determine the required membrane area to match that condition. The detail modelling of water balance and related results with comparison to other competing systems are presented in the **Revised Manuscript:**

Lines 269–284: "Guided by this model, the suggested OMD configuration was then integrated with AWE to determine H_2 output and operational stability. Our OMD-AWE with 0.5 M NaCl feed at 40°C and a 6 M KOH osmotic agent, which shows a water flux of 22.44 LHM, driven by 4.6 kPa ΔP_v, requires J_m of 1.34 A cm^{-2} (Fig. 3a). Due to the fixed geometry of our custom-made membrane cell (S = 1 cm^2), the AWE current was used as the adjustable parameter to ensure the water balance and determined to be 1.34 A. This approach is also adaptable for the other scenario where AWE optimal current is fixed to determine the needed membrane area. For subsequent continuous operation, the H_2 production flux (i.e., H_2 production rate per m^2 of membrane) was measured by water displacement and compared with other competing integrated systems (Fig. 3b). The OMD-AWE exhibited the highest H_2 production flux of 60.2 kg day^{-1} m^{-2}, which was over 25 times higher than that of isothermal SDE-DSE (or OD-AWE), attributed to a 20-fold increase in ΔP_v from heating the feed to 40°C. The H_2 flux was also around 4 times higher than that of previous record-high $FOWS_{AWE}$ when fed with 0.5 M NaCl, demonstrating that the combined thermal and osmotic gradient provides a far more potent driving force for water transport than the osmotic potential alone in liquid-phase systems."

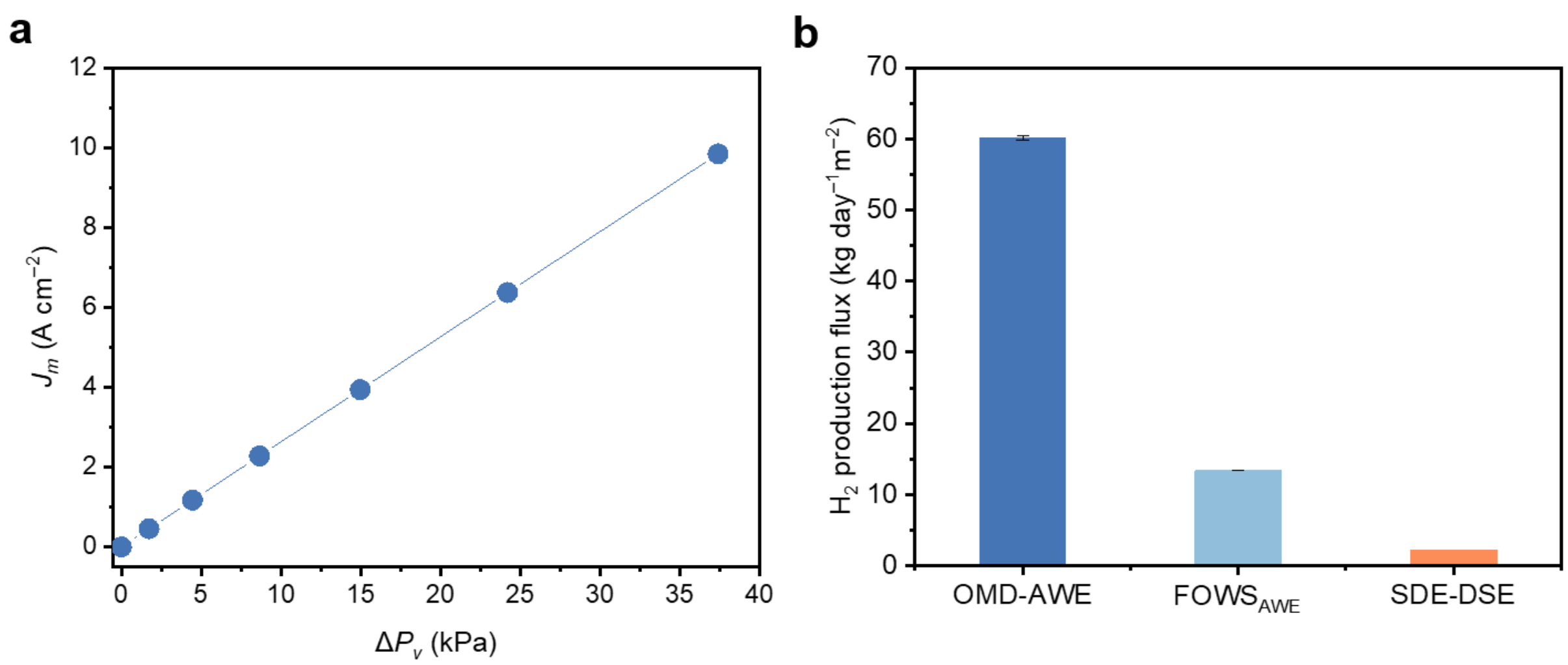

Fig. 3 Key performance metrics of OMD-AWE. a J_m as a function of ΔP_v to maintain water-hydrogen balance for continuous OMD-AWE operation; **b** comparative H_2 production flux of OMD-AWE, $FOWS_{AWE}$, and SDE-DSE (also called OD-AWE in this work);

Comment 5: Additional comments

The title and abstract could be revised to better reflect the full scope of the work, including the TFC membrane and osmotic distillation.

Please write out the full term "osmotic membrane distillation (OMD)" at first mention in the abstract (line 26).

The manuscript currently lacks a clearly labeled "Introduction" section, which may affect readability.

The sentence on lines 44–45 regarding energy losses from RO is unclear and sounds not logical. Please consider revising for clarity and logic flow.

Reply:

We thank the reviewer for the careful reading and these helpful suggestions to ensure the title and abstract accurately reflect the full scope of our work. We have revised both the title and abstract to more clearly emphasize the core novelty of our work: the precisely engineered thermal symbiosis between OMD and AWE and the leading performance outcomes that this unique thermal integration enables.

The revised **Title** now is: "Thermally symbiotic osmotic membrane distillation-electrolysis unlocks efficient and stable hydrogen production directly from seawater"

The revised **Abstract** is: "Integrating water purification membranes with electrolysis for in-situ hydrogen (H_2) production from seawater offers a rapid pathway to net-zero, but is limited by salt

crossover and insufficient water production in existing approaches. Here we overcome these limitations with a thermally symbiotic system that integrates osmotic membrane distillation (OMD) with alkaline water electrolysis (AWE). Through a self-sustaining thermal loop design, the AWE's waste heat is converted into OMD's thermal driving force, enhancing water vapor transport while completely blocking salt passages, and simultaneously providing passive cooling to maintain AWE optimal temperatures. This thermal symbiosis results in a record-high H_2 production rate of 60.2 kg day^{-1} m^{-2} with continuous 500-h stable operation, delivering best-in-class energy efficiency of 51.05 kWh kg$_{(H2)}^{-1}$ and competitive costs of \$5.72 kg$_{(H2)}^{-1}$. Our thermal-water-hydrogen model provides a flexible design framework, enabling precise matching of OMD and AWE at any configuration while determining optimal operational windows for thermal self-sufficiency at any scale. This system establishes a new benchmark for membrane-integrated electrolysis, demonstrating both technical excellence and economic viability towards a sustainable hydrogen economy."

In addition, we have defined the full term "osmotic membrane distillation (OMD)" and other terms at its first mention and added the "Introduction" heading. The claims on RO's energy consumption are revised from **the revised Manuscript**:

Former text in Lines 44–45: "Although RO consumes only 1% of total system energy, this energy is permanently lost, whereas hydrogen stores recoverable energy" was revised as:

Lines 42–45: "Although RO's energy consumption is only 1% compared to water electrolysis, this energy is permanently lost, whereas the majority of energy supplied to electrolysis is not lost but rather converted and stored as chemical potential energy within the H_2 product itself."

Responses to comments of Reviewer 2:

Direct seawater electrolysis has garnered significant attention, thereby promoting the development of chlorine-resistant and selective electrocatalysts as well as membrane separators. This manuscript submitted by Song et al. integrated hydrophobic polytetrafluoroethylene (PTFE) membranes with alkaline electrolysis enabling sustainable hydrogen production from seawater by selectively permitting water vapor transport while blocking salt penetration. Enhanced dual osmotic/vapor-pressure gradients achieve a hydrogen output of 52.7 kg day^{-1} m^{-2}, quadrupling conventional hydrophilic membrane performance while maintaining system durability. Thermal self-circulation utilizing electrolyzer waste heat via dual heat exchangers eliminates external energy inputs, reducing energy consumption to 47.8 kWh kg^{-1} while co-generating purified water. This integrated approach synergistically addresses renewable hydrogen scalability and freshwater scarcity with efficiency rivaling pure-water electrolysis systems. Therefore, I recommend publishing this manuscript after minor revisions. Below is a list of specific comments and questions the authors should address before publication.

Reply:

We thank the reviewer for the supportive and positive review. We are delighted that you recognized the key strengths of our integrated approach, particularly the high hydrogen output, the thermal self-circulation, and the overall system efficiency. As requested, we have addressed all of the minor points and questions. A detailed point-by-point response is provided below, and all corresponding changes have been made in the revised manuscript.

Comment 1:

Will the surface structure and micro-pore structure of the PTFE membrane gradually undergo irreversible changes in multiple long-term cycles? How to evaluate its lifespan and regeneration performance?

Reply:

We thank the reviewer for this critical question about PTFE membrane long-term performance. Conventional membrane operations face challenges from wetting, scaling, and frequent cleaning, which cause irreversible membrane degradation. Our work demonstrates that operating OMD at an optimized feed temperature of 40°C preserves membrane surface and micro-pore structures, maintaining salt rejection for the electrolyzer. This strategy significantly reduces the rate of fouling, the need for aggressive regeneration, and the risk of irreversible microstructural changes, thereby suggesting a substantially prolonged operational lifespan. This contrasts with conventional water purification approaches that favor higher temperatures (>60°C) to maximize water flux under a "hotter is better" principle[1,2]. This 40°C operation strategy was supported by our newly added experiments including **i) liquid entry pressure (LEP) tests** for evaluating the PTFE membrane's

resistance to wetting, **ii) accelerated wetting and scaling tests and regeneration**, and iii) **a 500-h long continuous operation** of the integrated OMD-AWE system, which are summarized below:

1. **Prevention of irreversible wetting:** At the optimal 40°C, the membrane maintains a high LEP of over 300 kPa for seawater, providing a robust safety margin against pore wetting (Fig. 2c). Crucially, our accelerated tests confirm that operating at 40°C extends the stable operation period against surfactant-induced wetting by five times compared to operation at 60°C (Fig. 2d), directly mitigating a primary mechanism of irreversible failure. Membrane regeneration through pure water backwashing and oven drying restored performance, However, the 60°C operation requires more frequent regeneration cycles, which not only consumes energy and purified water but also increases the risk of irreversible membrane damage as each cycle may leave residual surfactants that accelerate subsequent re-wetting.
2. **Control of Scaling:** Irreversible changes are often caused by severe scaling that damages the membrane or becomes difficult to remove. Gypsum is especially the most difficult one, forms compact and adherent scale layers that are resistant to simple acid cleaning. Through operating at 40°C, gypsum scaling is slower and the formed scale is superficial and can be completely removed with an ethylenediaminetetraacetic acid (EDTA) wash, recovering initial flux over multiple cycles (Fig. 2e). In contrast, operation at 60°C leads to rapid, deep-seated scaling that is harder to remove and drastically increases the frequency of chemical cleaning.
3. **Long lifespan:** We validated the membrane's durability through a continuous 500-h test where the OMD unit was integrated with an AWE electrolyzer. Throughout this extended operation, the system exhibited stable water flux, constant cell voltage, Faradaic efficiency >99%, and rejection of major seawater salts > 99.9% (Figs. 3c–d). This stable performance, combined with our regeneration protocol for reversible changes, demonstrates membrane durability under real operating conditions and suggests its lifespan is likely to extend far beyond 500 h.

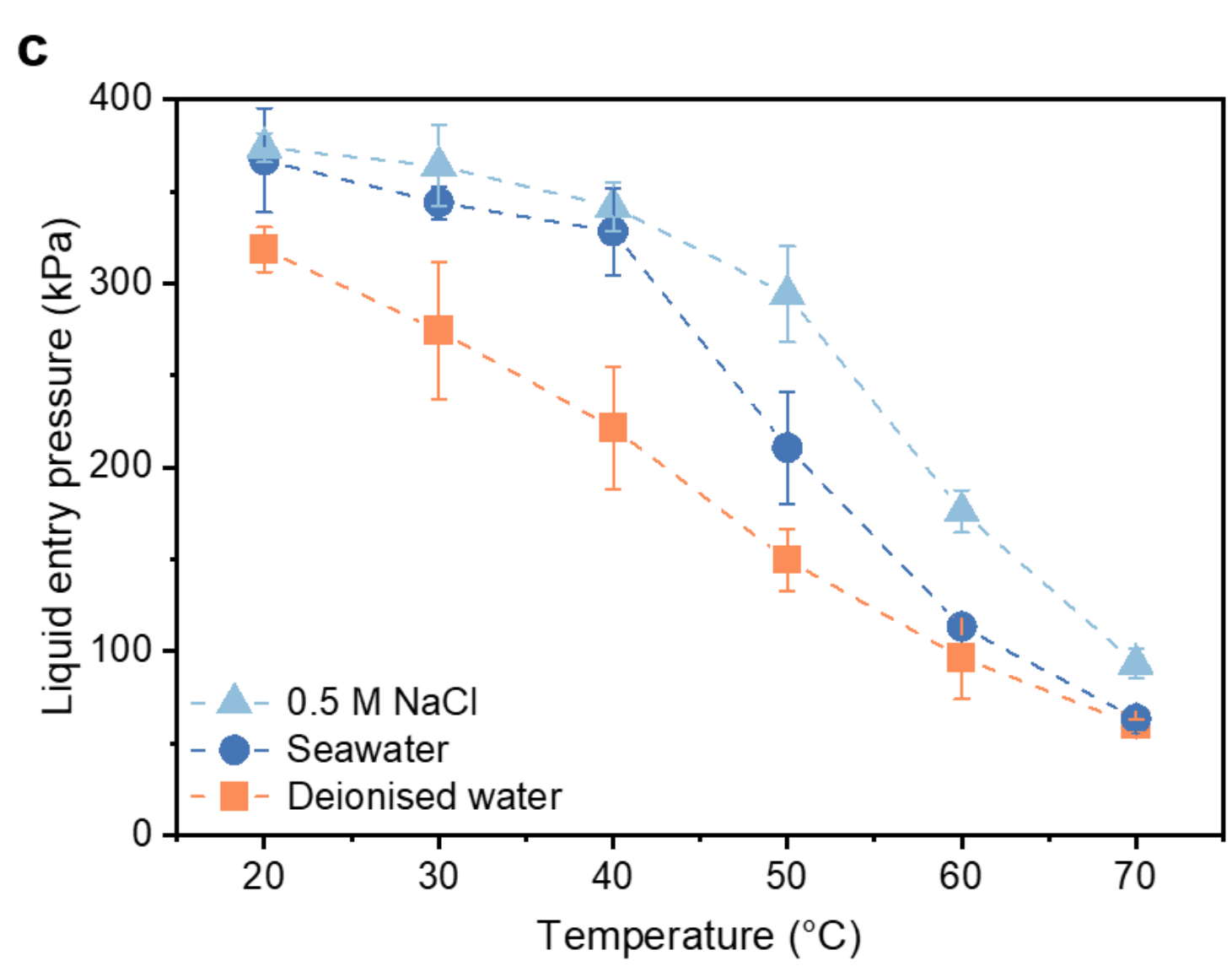

Fig. 2 c LEP as a function of feed temperature tested with pure water, 0.5 M NaCl, real seawater.

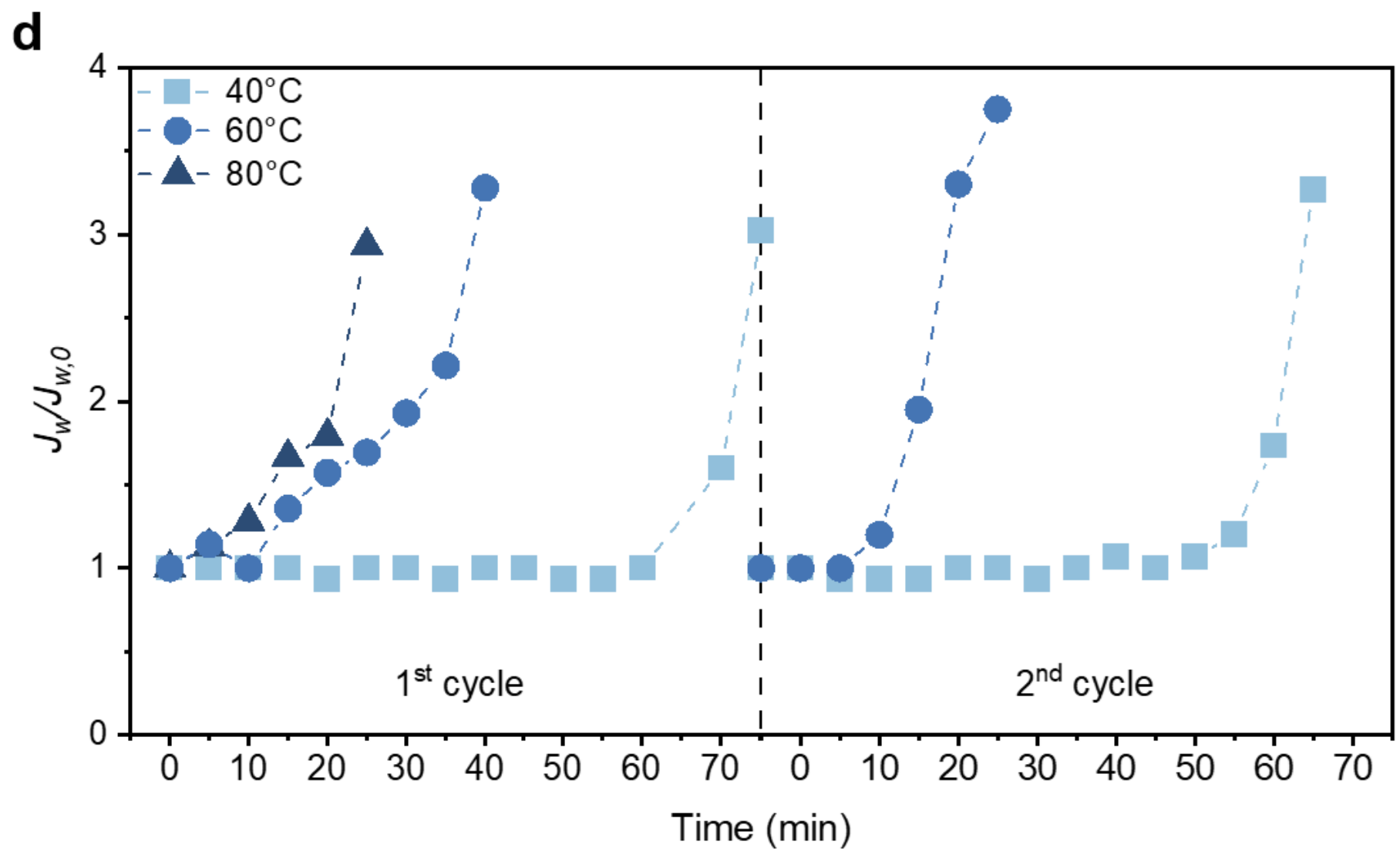

Fig. 2 d the ratio of real-time water flux to initial water flux ($J_w/J_{w,0}$) at different feed temperatures during accelerated wetting tests using 0.5 M NaCl feed containing 0.01 mM SDBS and 6 M KOH as osmotic agent.

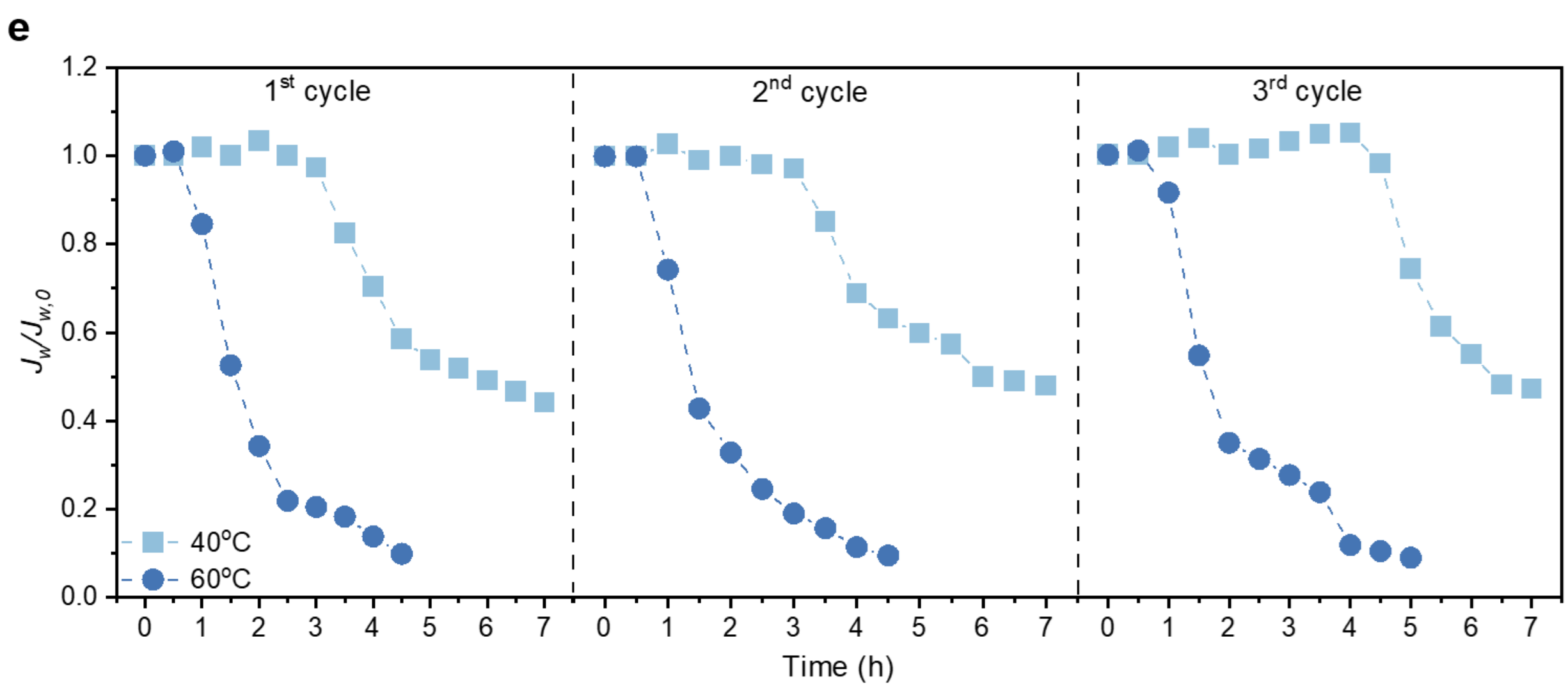

Fig. 2 e $J_w/J_{w,0}$ at different feed temperatures during accelerated scaling tests using a feed containing 30 mM Na_2SO_4 and 30 mM $CaCl_2$ and 6 M KOH as osmotic agent. All error bars represent standard deviation from two independent replicates.

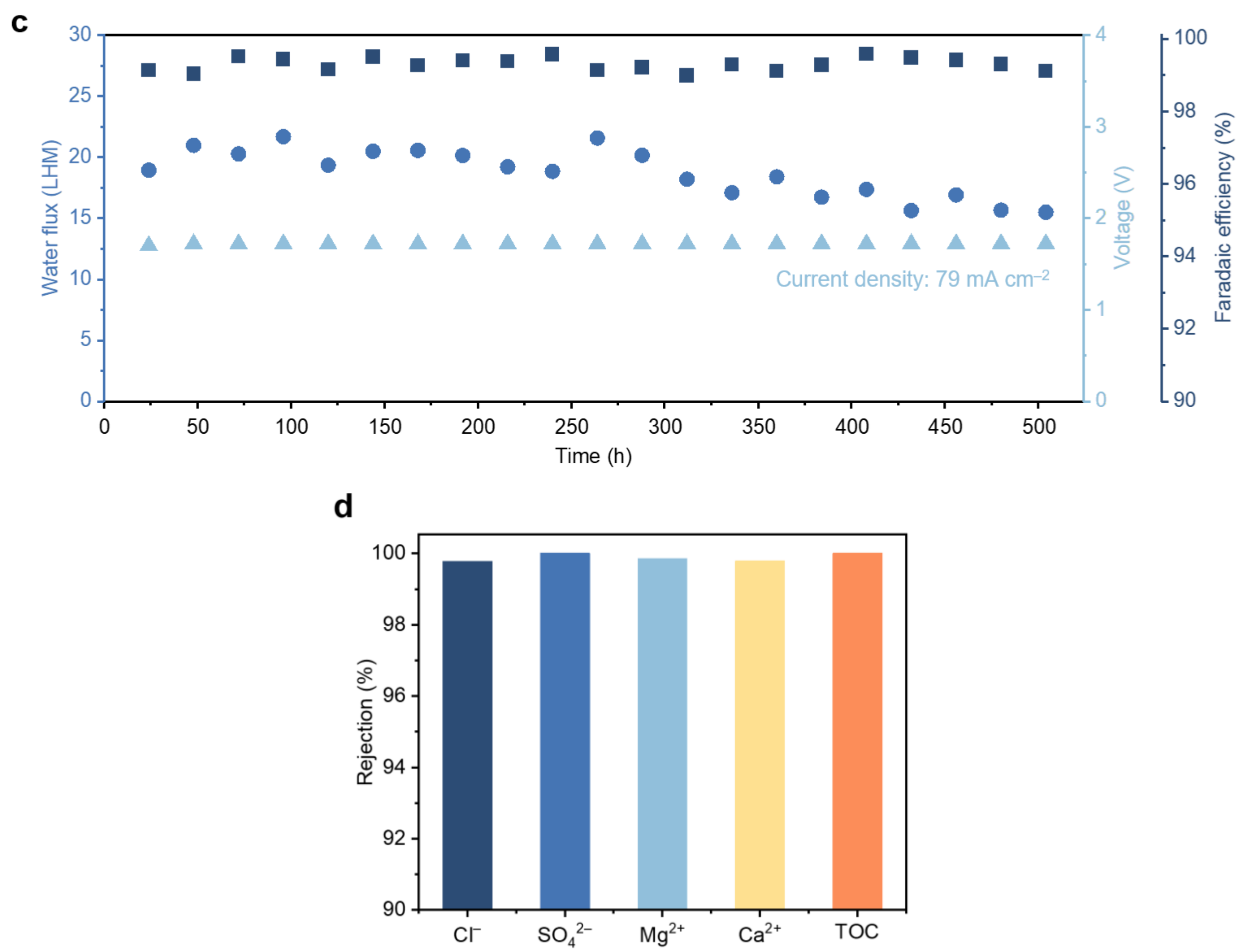

Fig. 3 c a 500-h continuous OMD-AWE operation tracking water flux, cell voltage, and Faradaic efficiency (Conditions: [feed] = seawater, [KOH] = 6 M, T_{feed} = 40°C, T_{KOH} = 20°C); **d** the rejection of chloride (Cl^-), sulfate (SO_4^{2-}), calcium (Ca^{2+}), magnesium (Mg^{2+}), and total organic carbon (TOC) at the end of the 500-h operation

The detailed analysis and discussion about temperature-dependent membrane wetting and scaling and regeneration are now available in the **revised Manuscript (Thermal trade-offs between water flux and stability of PTFE membranes)**:

Lines 164–220: "To ensure stability of the selected OMD, a key challenge is preventing membrane wetting, where liquid feed penetrates its hydrophobic pores and compromises salt rejection. The PTFE membrane's resistance to wetting was evaluated by measuring its LEP (i.e., the minimum pressure required to force liquid through) at various temperatures for pure water, a seawater-equivalent 0.5 M NaCl solution, and real seawater (Fig. 2c). In general, the LEP for all these test liquids is highly sensitive to temperature, dropping over 75% with increasing liquid temperature from 20°C to 70°C, due to the decreased water surface tension and thermal softening of the PTFE polymer allowing the pores to stretch and enlarge under pressure. A reduced LEP indicates increased susceptibility to wetting and reduced pressure tolerance, risking operational failure. This

forces the system to be operated at lower temperatures to maintain a safe wetting margin, which limits the water flux. However, the presence of dissolved salts in NaCl and seawater increases LEP by 17% over pure water at 20°C, while providing better temperature stability up to 40°C with values maintaining above 300 kPa. These salt ions, surrounded hydration shells, are repelled from the hydrophobic PTFE surface, creating a more ordered water layer at the interface which increases the contact angle and balances out the softening. A tipping point was observed at 40°C where the LEP began to drop sharply for both saline liquids, since the thermal energy becomes sufficient to overcome the salt-induced interfacial stability, shifting the primary failure mechanism to polymer softening. However, seawater showed a more rapid LEP decline than NaCl at high temperatures, likely due to natural surfactants reaching their Kraft points (typically 15–50°C for most alkyl-chain ionic surfactants (C10–C16 chains). Above these temperatures, the surfactants become highly soluble and form micelles instead of crystals or gels, allowing them to adsorb to the liquid-vapour interface at the pore entrances where they lower the local surface tension and enable liquid permeation at a lower pressure.

To validate these LEP findings, surfactant-accelerated wetting tests were conducted using 0.5 M NaCl spiked with 0.1 mM sodium dodecylbenzenesulfonate (SDBS), a common C12 alkyl-chain ionic surfactant, at 40°C, 60°C, and 80°C (Fig. 2d). The membrane performance was monitored through the ratio of real-time water flux to initial water flux ($J_w/J_{w,0}$), where a ratio at ~1.0 indicates stable operation and a sharp increase signals wetting. At 80°C, immediate membrane wetting occurred, while 60°C maintained stability for only 10 min before wetting. Operation at 40°C extended the stable period by 5 times compared to 60°C. Membrane regeneration through pure water backwashing and oven drying restored performance for both 40°C and 60°C. However, the 60°C operation requires more frequent regeneration cycles, which not only consumes energy and purified water but also increases the risk of irreversible membrane damage as each cycle may leave residual surfactants that accelerate subsequent re-wetting. These findings support 40°C as an optimal feed temperature, maintaining wetting resistance with reduced surfactant-induced issue.

Accelerated scaling tests using gypsum-supersaturated solutions (30 mM calcium chloride ($CaCl_2$) and 30 mM sodium sulfate (Na_2SO_4)) were conducted to examine scaling behaviour at different temperature. Gypsum was selected as a model scalant as its crystallisation is predominantly temperature-dependant, exhibits minimal sensitivity to pH adjustments, and forms more compact and adherent scale layers that are resistant to simple acid cleaning than softer and more manageable $CaCO_3$ scales. As shown in Fig. 2e, the membrane at 40°C maintained a stable water flux for around 200 min before this predictable scaling began to impede water transport. Importantly, the scale was fully removed by a 30-min chemical wash with 0.05 M chelating agent ethylenediaminetetraacetic acid (EDTA), recovering the initial flux over three cycles, demonstrating reversible scaling at 40°C. In contrast, at 60°C, scaling behaviour is markedly more aggressive due to the inverse solubility of gypsum and kinetic acceleration of crystal nucleation and growth at the membrane interface, leading to rapid scaling and a sharp flux decline within 100 min. Furthermore, the higher temperature promotes scale formation deeper within the membrane

pores rather than just surface crystallisation, reducing cleaning efficiency and potentially requiring longer cleaning times or higher EDTA concentrations for full recovery. The scaling characteristics, combined with LEP analysis and wetting tests, further reinforce 40°C as the optimal OMD temperature for AWE integration, offering the best balance between water flux and membrane stability to ensure zero salt contamination. This contrasts with drinking water MD systems that prioritises maximum output over 60°C while occasional salt passage is acceptable for human health."

The 500-h continues test and detailed analysis are added to the **Revised Manuscript**:

Lines 285–314: "The long-term stability of OMD-AWE was evaluated through a 500-h continuous test with real seawater as shown in Fig. 3c. The OMD process demonstrated a stable water flux of 20.11 ± 0.98 LHM for the first 300 h before gradually declining to 16.67 ± 0.94 LHM by the end of the test, likely due to the initial stages of fouling formation. The electrochemical performance also remained stable with cell voltage stabilised at 1.72 V and Faradaic efficiency remained >99% throughout the entire experiment, confirming that neither seawater impurities nor initial fouling compromise the electrolyser kinetics or separator conductivity. Post-operational analysis was conducted to investigate the cause of the water flux decline and to assess component durability using scanning electron microscopy (SEM) and energy-dispersive X-ray spectroscopy (EDX). The PTFE membrane exhibited signs of surface fouling after 500 h of use, showing a thickened morphology in SEM images (Supplementary Fig. 4). EDX analysis confirmed the presence of organic fouling indicated by an increased carbon content with a reduced F/C atomic ratio from 0.94 to 0.89 (Supplementary Table 5). Localised spots of inorganic scaling composed of magnesium, calcium, and potassium were also identified (Supplementary Fig. 5). While this combination of fouling and scaling might have contributed to the observed water flux reduction, they can be both preventable with antiscalants or basic pre-filtration (e.g., sand filters) and readily removed by standard water, acid or chelating agent washing. Crucially, these surface deposits did not compromise the membrane's core functionality, as it maintained exceptional ion rejection rates over 99% for all major seawater constituents (Fig. 3d). Analysis of the electrolyser electrodes confirmed their pristine condition and complete protection from seawater contaminants. Both SEM and EDX revealed that the Ni nanowire cathode and NiFe anode retained their structural integrity and original elemental composition, with no surface fouling or traces of contaminants such as calcium, magnesium, or silica (Supplementary Figs. 6–7, Supplementary Table 6). The integrated OMD-AWE system also maintained a stable concentration for the KOH osmotic agent throughout the 500-h test (Supplementary Fig. 8). In addition, our physically decoupled modular design provides crucial operational flexibility. This allows either the OMD or AWE to be taken offline for routine maintenance (e.g., membrane cleaning) without system failure. The ability to handle these interruptions and return to stable operation is essential for robust, long-term operation beyond 500 h during real-world applications."

References:

1. Kim, HW., Jang, A. & Jeong, S. Operational strategy preventing scaling and wetting in an intermittent membrane distillation process. *npj Clean Water* **6**, 50 (2023).
2. López-Porfiri, P., Ramos-Paredes, S., Núñez, P. *et al.* Towards the technological maturity of membrane distillation: the MD module performance curve. *npj Clean Water* **6**, 18 (2023).

Comment 2:

In the current research system, apart from Ni-based alloy electrodes, how does the author consider the diversity of electrode materials and the compatibility with the membrane?

Reply:

We thank the reviewer for this insightful question for electrode material diversity and compatibility with membranes. The core of our integrated system is the completely blocking of salts for clean water supply to electrolyzers and the use of a concentrated liquid electrolyte as an osmotic agent for the OMD process. Its powerful osmotic gradient enables OMD a higher water flux compared to purely thermal MD processes, allowing operation at lower and more practical temperatures. These properties make our system highly adaptable but also defines its ideal application space. Our system is inherently adaptable to any electrolyzer technology that utilizes a concentrated liquid electrolyte. The chemically stable PTFE membrane acts as a robust barrier, effectively preventing salt crossover and protecting the electrolyzer's internal components, regardless of the specific electrode materials used. This flexibility allows for the integration with various catalyst materials developed for alkaline, neutral, and even acidified electrolysis as far as they can provided osmotic gradient. Our OMD-AWE with Ni-based cathode and NiFe-based anodes is a good example with its ability to handle 6 M KOH for enhanced water production and water electrolysis. This contrasts with other integrated systems with FO where a thin-film composite (TFC) membranes can only tolerate much lower concentrations (1 M KOH), limiting its performance[1]. However, there are other types of electrolysis like PEM and AEM where they require pure water as their feed[2], which cannot provide the osmotic gradient for water production. Integrating with PEM or AEM electrolyzers remains technically possible by using less efficient DCMD processes due to the the absence of a concentrated liquid electrolyte. In addition, reliance of PEM systems on expensive precious metal catalysts, such as iridium oxide and platinum, runs counter to our goal of developing a low-cost system. Therefore, we prioritized AWE for this foundational study. Its technological maturity and the use of low-cost and abundant non-precious metal catalysts make it an ideal partner for PTFE membranes. The combination of AWE's robust, cost-effective nature with the performance gain from using its concentrated KOH electrolyte makes the AWE-OMD integration a particularly compelling and economically viable pathway for hydrogen production from non-freshwater sources.

The following insights has been added to the **Revised Manuscript**:

Lines 483–486: "Other four systems consume around 3 times more BoP electricity on cooling, while $FOWS_{AWE}$ and SDE-DSE requires 7% and 14% more electricity for water splitting due to

the FO membrane's intolerance to KOH concentrations above 1 M and temperature constraints, respectively."

Lines 530–538: "The system offers broad compatibility with any electrolyser that utilises a concentrated liquid electrolyte, enabling the use of diverse electrode materials, from non-precious metal catalysts in AWE to those developed for other alkaline, neutral, or acidified electrolysis. This versatility is enabled by the chemically inert PTFE membrane and completely isolation of the electrolyser from seawater contaminants. The continuous supply of high-purity water decouples seawater utilisation challenges from the electrolyser development constrained by salt-tolerant materials, allowing research to focus entirely on the key commercial barriers of cost and durability in proven pure-water systems. This focus will accelerate industrial adoption and substantially reduce the cost of green H_2."

Reference:

1. Scheibel Cassol, G., Shang, C., An, A. K., Khanzada, N. K., Ciucci, F., Manzotti, A., Westerhoff, P., Song, Y. & Ling, L. Ultra-fast green hydrogen production from municipal wastewater by an integrated forward osmosis-alkaline water electrolysis system. Nat Commun 15, 2617 (2024).
2. El-Shafie, M. Hydrogen production by water electrolysis technologies: A review. Results in Engineering 20, 101426 (2023).

Comment 3:

Will the microorganisms, organic pollutants or colloids existing in real seawater affect the membrane flux or cause pollution? Is additional pretreatment necessary?

Reply:

We thank the reviewer for this critical question regarding the impact of real seawater constituents on membranes. Indeed, organic pollutants, microorganisms, and colloids can affect long-term membrane performance by causing organic fouling and biofouling[1,2]. Soluble organics (e.g., humic acids) can adsorb onto the hydrophobic membrane surface, reducing its ability to repel liquid water. This can lower the vapor pressure gradient and lead to a decline in water flux. Meanwhile, microorganisms can colonise the membrane surface, forming a hydrated biofilm[3]. This layer acts as a secondary barrier, increasing resistance to vapor transport and exacerbating flux decline.

Our 500-h continuous operation of OMD-AWE with untreated real seawater confirmed this. SEM images show thickened morphology PTFE membrane polymers (Supplementary Fig. 4) and EDX detected an increased carbon signal on the membrane surface (Supplementary Table 5), likely due to the combined effects of organic fouling and biofouling. However, key performance metrics such as water flux, cell voltage, Faradaic efficiency, and salt rejection remained stable (see Comment 1), suggesting that the initial stage of fouling did not compromise system stability. In additions, such fouling is manageable and reversible. Simple backwashing is sufficient to remove the foulant

layer when observing a slight water flux reduction. Although a small amount of residual fouling may accumulate and become difficult to remove over time, a chemical cleaning procedure can effectively restore membrane hydrophobicity and water flux[1]. For a full-scale system, a low-cost pre-filtration step (e.g., a sand filter or cartridge filter) would be a practical addition. It would significantly reduce the frequency of backwashing, ensuring high operational uptime with minimal complexity.

Beyond biological and organic fouling, we have also conducted an extensive study on other key seawater constituents, such as surfactants and inorganic scalants. As detailed in our response to Comment 1, a central finding of our work is the identification of 40°C as the optimal operating temperature. This moderate temperature, enabled by the high driving force of the OMD process, simultaneously mitigates two key risks: surfactant-induced wetting and inorganic scaling. It operates below the critical threshold where natural surfactants become highly soluble and adsorb to the membrane pores. Furthermore, this 40°C temperature significantly reduces the scaling propensity of salts like $CaSO_4$ compared to conventional high-temperature membrane distillation, thereby enhancing the membrane's operational lifespan. Therefore, we are confident that the combination of minimal pretreatment (e.g., filtration) and our optimized, low-temperature OMD operation provides a robust strategy to ensure sustained performance with minimal operational complexity.

These are added to the **Revised Manuscript:**

Lines 291–303: "Post-operational analysis was conducted to investigate the cause of the water flux decline and to assess component durability using scanning electron microscopy (SEM) and energy-dispersive X-ray spectroscopy (EDX). SEM images revealed thickened morphology of the PTFE membrane after 500 h of use, suggesting slight fouling (Supplementary Fig. 4). EDX analysis confirmed the presence of organic fouling indicated by an increased carbon content with a reduced F/C atomic ratio from 0.94 to 0.89 (Supplementary Table 5). Localised spots of inorganic scaling composed of magnesium, calcium, and potassium were also identified (Supplementary Fig. 5). While this combination of fouling and scaling might have contributed to the observed water flux reduction, they can be both preventable with antiscalants or basic pre-filtration (e.g., sand filters) and readily removed by standard water, acid or chelating agent washing. Crucially, these surface deposits did not compromise the membrane's core functionality, as it maintained exceptional ion rejection rates over 99% for all major seawater constituents (Fig. 3d)."

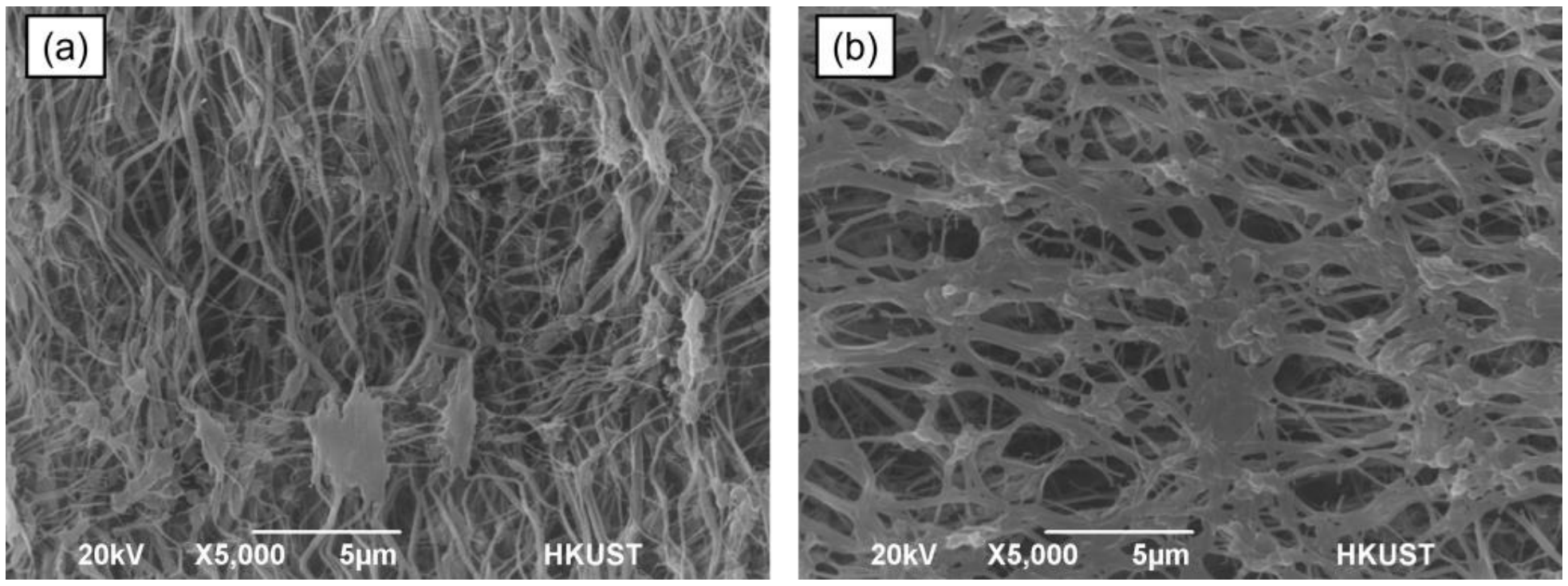

Supplementary Fig. 4 Scanning electron microscopy (SEM) images of the PTFE membrane surface morphology. **a** pristine membrane; and **b** the membrane after 500-h operation. Conditions: [Feed] = seawater, [KOH] = 6.0 M, T_{feed} = 40°C, T_{KOH} = 20°C.

Supplementary Table 5. EDX surface elemental composition analysis of the PTFE membrane before and after the 500 h continuous operation.

PTFE membrane	Element	Atom (%)
Pristine	C	46.30
	F	49.23
	Others	4.47
	F/C ratio	0.94
After 500 hours	C	49.74
	F	44.33
	Others	5.95
	F/C ratio	0.89

Reference:

1. Naidu, G., Jeong, S., Kim, S.-J., Kim, I. S. & Vigneswaran, S. Organic fouling behavior in direct contact membrane distillation. *Desalination* 347, 230–239 (2014).
2. Kakkar, R., Kaur, D. P. & Raj, S. Membrane Distillation for Sustainable Water Desalination: A Review of Principles, Materials, and Applications. *Water Air Soil Pollut* 236, 24 (2025).
3. Gryta, M. The assessment of microorganism growth in the membrane distillation system. *Desalination* 142, 79–88 (2002).

Comment 4:

How does the author consider the scale-up of system integration, such as the increase in membrane area, and whether it will bring new problems in heat transfer, mass transfer, etc.?

Reply:

We appreciate the reviewer's critical question about scaling up our integrated system. We have indeed considered these challenges, and the OMD process was specifically chosen for its inherent resilience to the heat and mass transfer issues that intensify with larger membrane areas. In any thermally driven MD process, the latent heat of vaporization presents a fundamental challenge[1]. Each water molecule crossing the membrane must first absorb latent heat to vaporize at the hot feed surface, then release this heat upon condensing at the cooler permeate[2]. This intrinsic heat transfer is not an inefficiency but rather the core engine of the process, as vapor transport inherently cools the feed while heating the permeate. This causes temperature polarization at the membrane surface and a significant axial temperature drop along the flow path (Fig. 2b), compromising water flux and subsequent H_2 production. These challenges intensify with larger membrane areas and longer modules. Although conventional MD systems can compensate for this latent heat loss with continuous reheating to maintain the thermal gradient, that is not a viable option for our integration, which is designed to operate solely on the finite waste heat from the AWE. This is precisely where the osmotic driving force from the 6 M KOH becomes indispensable for large-scale applications. It provides consistent vapor transport independent of thermal gradients, maintaining stable water flux even when thermal driving force weakens along larger membrane modules. This osmotic "backup" makes OMD uniquely robust against thermal inefficiencies that typically limit thermal MD scaling, making our integrated design inherently suitable for large-scale applications with limited heat sources.

We also did a direct quantitative assessment of the membrane at a practical, industrial scale of a 1 MW AWE system. A 1 MW electrolyzer producing around 20 kg(H_2) h^{-1} which requires a supply of 200 kg h^{-1} of clean water (based on the common industry benchmark of 10 kg of water per kg of H_2 produced). Our OMD system's water flux is 20 kg m^{-2} h^{-1}, this demand can be met with only 10 m^2 of membrane area. Modern membrane modules (e.g., spiral-wound) have high packing densities. A module containing 10 m^2 of membrane has a small physical footprint, conservatively estimated to be less than 1 m^2 of ground space. When compared to the ~15–20 m^2 footprint of the 1 MW AWE and its balance of plant, the OMD's contribution to the total system footprint is very small.

This compact membrane footprint stems from our thermal circulation design's excellent heat and mass transfer characteristics. Our counter-intuitive strategy of low water recovery enhances scalability by enabling higher water flux. This approach fundamentally differs from conventional standalone membrane processes, where high recovery is essential due to costly pretreatment, extensive pumping and heating energy, and complex brine disposal requirements. For example, RO requires pretreatment (sand filtration, ultrafiltration, chemical dosing) and energy-intensive high-pressure pumps. Similarly, standalone MD requires significant external heating to maintain 60-90°C feed temperatures[3]. Therefore, a higher water recovery and larger membranes areas they used means getting more clean water out, and the cost for per unit of product water drops significantly. Their design is therefore optimized for maximum water resource efficiency. Our OMD-AWE system's limiting resource is not the water, but the finite waste heat from the AWE

electrolyzer. Our design is therefore optimized for maximum system-level energy efficiency and stability. This paradigm shift is precisely why a low water recovery (<2.5%) becomes a powerful asset (suggested by our thermal balance model in the **Revised Manuscript Lines 340–462 Thermal self-circulation improves OMD-AWE energy efficiency**). The high seawater flowrate serves as an efficient heat transfer engine, capturing AWE's waste heat and maintaining sufficient thermal energy for stable water production to overcome the latent heat loss of vaporization. Conversely, a low-flowrate (high-recovery) design faces a critical trade-off that makes it unsuitable for this integration. If this low flowrate were to absorb the full waste heat, its temperature would rise far above the optimal 40°C, creating unacceptable risks of fouling and wetting. If, on the other hand, it were only heated to the safe 40°C, it would carry an insufficient thermal load. This heat would be rapidly consumed by vaporization, causing a steep temperature drop along the membrane. The resulting collapse of the driving force would either require an impractically large membrane area or, more likely, lead to a complete system failure.

Therefore, except the osmotic gradient provided by OMD itself, the OMD's low recovery design is another key insight for ensuring the system's scalability. We strategically trade a negligible amount of low-pressure pumping energy for massive gains in energy efficiency and operational stability. This confirms that our design is not constrained by membrane area and is fully scalable for practical, large-scale applications.

The insight of osmotic gradient against latent heat loss is added to the **revised Manuscript**:

Lines 148–163: "Second, the osmotic driving force makes OMD more resilient to thermal gradient loss caused by latent heat of evaporation. Each water molecule crossing the membrane must first absorb latent heat to vaporise at the hot feed surface, then release this heat upon condensing at the cooler permeate. This intrinsic heat transfer is not an inefficiency but rather the core engine of the process, as vapour transport inherently cools the feed while heating the permeate. This would cause both temperature polarization at the membrane surface and axial temperature drop along its flow path, challenges that intensify with larger membrane areas and longer modules when scaling up the process. Although continuous reheating can compensate this heat loss, it is not an option for our AWE integration which relies on finite waste heat. This is precisely where the osmotic gradient becomes indispensable, providing consistent driving force even as thermal gradients weaken along the module. This osmotic backup ensures stable water flux, making OMD uniquely suited for large-scale applications with limited or low-grade heat sources. Although OMD is overlooked for standalone water purification due to intensive energy consumption to regenerate its osmotic agent and separate the purified water, this issue is eliminated when integrated with AWE as the permeated water is directly consumed by electrolysis."

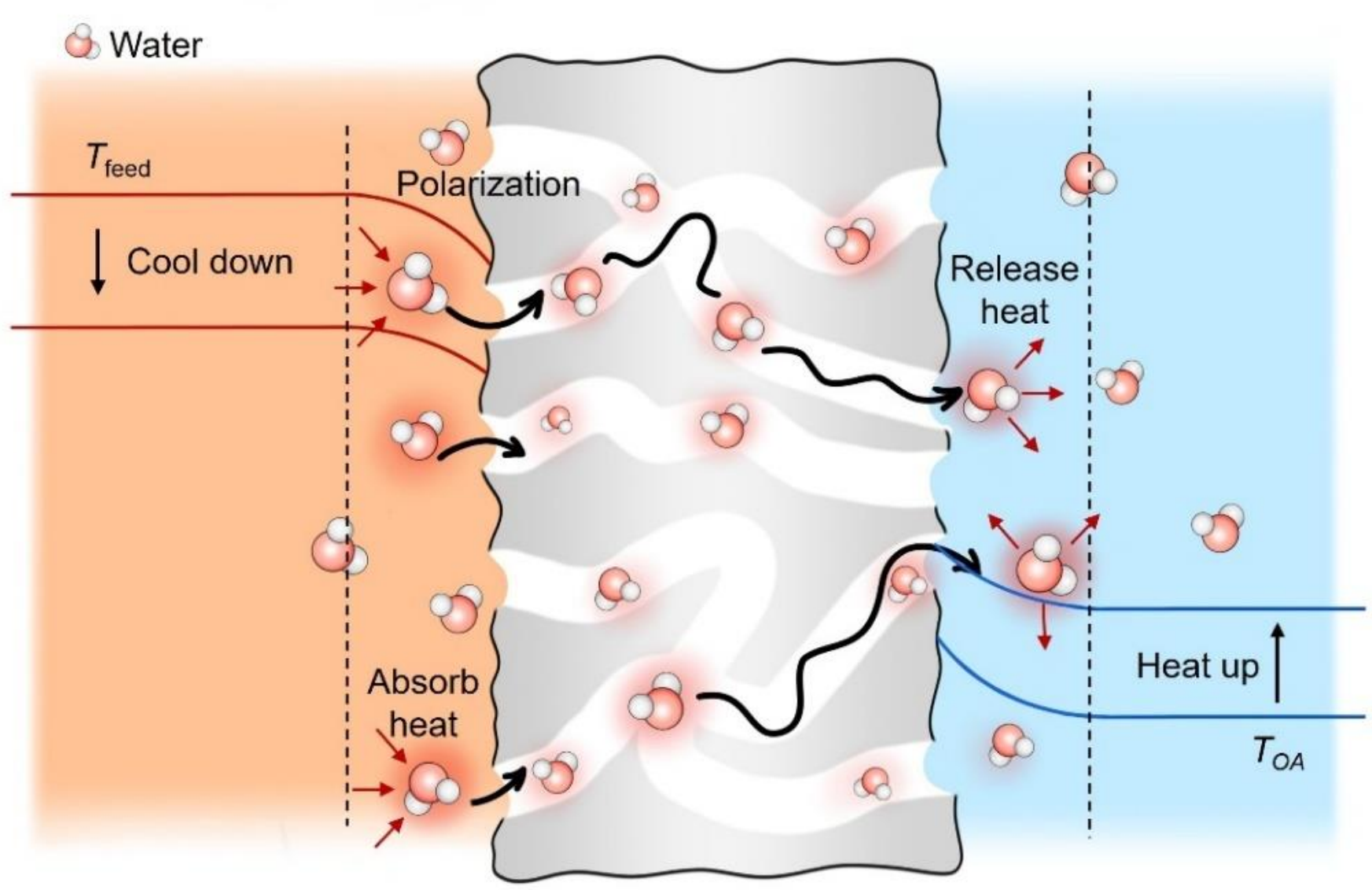

Fig. 2 b schematic showing thermal gradient loss from temperature polarization at the membrane surface or axial temperature drop along its flow path due to latent heat transfer during water vapor transport.

The insight of our low water recovery operation for capturing and productively use AWE's waste heat is added in our **Revised Manuscript**:

Lines 445–462: "Our strategic use of low RRs is the key that unlocks the thermal symbiosis, a design choice that is the direct opposite of the standalone desalination paradigm. In standalone systems, high water recovery is paramount as their primary costs and limiting resources are the pretreated feed water, extensive pumping and heating energy, and the high-salinity brine disposal. This forces them into a low-flowrate regime that is incapable of capturing large-scale, low-grade heat sources. Our design is optimised for maximum system-level energy efficiency and stability. This paradigm shift is precisely why a low RR becomes a powerful asset. By using abundant, untreated seawater in a high-flowrate, low-recovery loop, we create a powerful heat transfer engine. This allows the feed seawater to capture and productively use the AWE's waste heat as primary driver for efficient and stable water production at a flux over 20 LHM. The high water flux minimises the required membrane area to just 10 m^2 per MW of AWE capacity, resulting in a compact physical footprint (<1 m^2) by using high packing-density membrane modules that are negligible compared to the electrolyser installation. Another advantage of the low RR design is the simplification of brine management. It allows to produce brine with minimal salinity increase of <5% (Supplementary Fig. 14), falling within natural coastal salinity variations and enabling environmentally safe, direct ocean discharge without costly treatment processes. Ultimately, this design trades a negligible increase in low-pressure pumping energy for substantial gains in energy efficiency, operational stability, and simplified brine management."

Reference:

1. Martínez-DíEz, L. & Vázquez-González, M. I. Temperature and concentration polarization in membrane distillation of aqueous salt solutions. J Memb Sci 156, 265–273 (1999).
2. Zhao, S., Wardhaugh, L., Zhang, J. & Feron, P. H. M. Condensation, re-evaporation and associated heat transfer in membrane evaporation and sweeping gas membrane distillation. J Memb Sci 475, 445–454 (2015).
3. López-Porfiri, P., Ramos-Paredes, S., Núñez, P. *et al.* Towards the technological maturity of membrane distillation: the MD module performance curve. *npj Clean Water* **6**, 18 (2023).

Comment 5:

It is recommended to provide the full name of OMD upon its first appearance in the abstract.

Reply:

We appreciate the reviewer's reminder. All terms, including osmotic membrane distillation (OMD), have been defined at their first mention.

Responses to comments of Reviewer 3:

This manuscript proposed a membrane-integrated seawater electrolysis process of OMD-AWE to produce hydrogen with water electrolysis in alkaline condition, addressing the challenge in H2 production from non-freshwater sources. They provide that the integrated design uses only 0.4–1.1 kWh m–3 for water purification, and maintains energy efficiency comparable to commercial pure-water electrolyzer. This new process is interesting due to the utilization of waste heat from AWE processes; however, some critical issues have to be classified to improve its novelty.

Reply:

We sincerely thank the reviewer for these thoughtful comments and recognizing our OMD-AWE process's energy efficiency with waste heat utilization. Our manuscript has been enhanced with direct comparisons to state-of-the-art seawater electrolysis for better demonstrating our system's novelty. We also addressed critical MD challenges by our thermal design, supported by newly added wetting and scaling evaluation, a 500-h stability test, updated thermal balance modelling, and techno-economic analysis. All major changes are highlighted in blue in the revised manuscript and summarized in our point-by-point response.

Comment 1:

It is difficult to find significant progress with that a direct seawater electrolysis method for hydrogen production (Nature volume 612, pages673–678 (2022)), except for the harnessing waste heat from AWE for thermal self-circulation.

Reply:

We thank the reviewer for these insightful comments for clarifying the novelty of our work. The direct seawater electrolysis system reported in *Nature* 612, 673–678 (2022) combines isothermal OD with AWE in a physically coupled design for simultaneous water purification and electrolysis. While its water vapor transport mechanism through hydrophobic PTFE membranes ensures complete salt rejection, it presents critical limitations:

1. **Extremely low water flux that constrains its H_2 output.** The H_2 production relies on the water production as per mole of water consumed by electrolysis much equal to per mole of water produced from membranes for continuous operation. However, the water vapor flux across membranes via isothermal OD is insufficient limited by osmotic gradients creating only 0.24 kPa vapour pressure gradient (ΔP_v), This leads to a H_2 production rate of only 0.09% compared to liquid water transport FO-based systems[1,2], thus requiring significant large membrane area and installation footprint.
2. **Poor electrolyzer performance.** Due to the direct coupling of membrane and electrolyzer, AWE is only allowed to operate at room temperatures and is thus 25% less energy efficient than its optimal 60–90°C. If increasing the electrolyte temperature, its vapor pressure will exceed that of feed, leading to reverse vapor transport. In addition, pressurized H_2 production

is impossible as operating AWE above PTFE membrane's liquid entry pressure (LEP) at 200–500 kPa causes membrane wetting and compromises salt rejection[3].

3. **Significant energy penalty for overcoming the low water flux barrier.** The easiest and most effective approach to enhance the water flux is introducing a thermal gradient combined with the existing osmotic gradient, a process known as osmotic membrane distillation (OMD). For example, heating the feed from 20 to 40°C enables a 20-fold increase in ΔP_v and consequently 25 times higher water flux. However, it requires additional heating energy of 0.023 kWh for heating per kg of seawater from 20°C to 40°C and another 0.833 kWh per kg of produced water to maintain the thermal gradient against heat loss during vapor transport due to latent heat of vaporization. Thus, producing per kg of H_2 requires at least 10.6 kWh of energy for OMD water production, corresponding to over 22.6% increases relative to the baseline 47.1 kWh kg$(H_2)^{-1}$ needed for water splitting (details see our **Revised Manuscript Lines 315–329**). This energy consumption is also 17–70 times higher than that by conventional RO, making the external heating option economically unfeasible.

Consequently, scaling up this OD-AWE system for practical application would require substantial increases in the membrane area and power input, creating significant barriers to economic viability.

Our key innovation is **a thermally integrated design** that physically separates the membrane from the AWE electrolyzer while maintaining their thermal connection for symbiotic operation. This thermal integration converts AWE's waste heat, which is an operational burden requiring costly cooling, into a valuable asset that drives water vapor transport through OMD using the PTFE membrane. In return, OMD provides AWE with both passive cooling and a continuous supply of high-purity water. This allows both the OMD and AWE to operate under their respective optimal conditions simultaneously, without over-pressure and reverse flux issues. Such symbiosis is achieved by the strategic selection of OMD's 40°C feed temperature and 6 M KOH osmotic agent (as detailed in the newly added result "**Thermal trade-offs between water flux and stability of PTFE membranes**" in the **Revised Manuscript Lines 121–220**) and precisely designed heat exchange loops that allow the system for thermal self-sufficiency (as detailed in "**Thermal self-circulation improves OMD-AWE energy efficiency**" in the **Revised Manuscript Lines 340–462**). This low-temperature OMD operation provided sufficient water flux over 20 L h^{-1} m^{-2} (LHM) for H_2 production while significantly reduced the risks of PTFE membrane scaling and wetting for maintaining excellent salt rejection over time. Its unique dual-driving-force ensures stable water production that is more resilient to latent heat losses during water vapor transport. Due to the enhanced water flux via energy-free OMD and the AWE's high temperature operation under the thermal loops, this system delivered record-high 60.2 kg day^{-1} m^{-2} of H_2 production rate that is 25 times higher than that from the Nature paper, while maintaining stability over 500 h of continuous operation, achieving best-in-class energy consumption of 51.05 kWh kg$(H_2)^{-1}$ and a market-leading cost of \$5.72 kg$(H_2)^{-1}$, comparable to pure water electrolysis.

We have revised the manuscript to demonstrate our core innovation compared to *Nature* 612, 673–678 (2022), particularly the **Introduction**:

Lines 64–110: "Integrating PTFE membranes to AWE (PTFE-AWE), also known as self-dampening electrolyte-assisted direct seawater electrolysis (SDE-DSE), eliminates salt passages by allowing only water vapour transport through their hydrophobic membrane pores, maintaining stability over 3,200 h. However, this approach produced H_2 at a rate of 0.09% of $FOWS_{AWE}$ due to insufficient water supply, limited by its isothermal osmotic distillation (OD) creating a small 0.24 kPa vapour pressure gradient (ΔP_v). More critically, the direct coupling of OD with AWE creates inherent constraints for system optimisation and scaling, as both optimal water production and AWE conditions are not allowed. Heating the feed to activate thermally driven membrane distillation (MD) can improve ΔP_v by >20 times for enhanced water vapor transport (Supplementary Fig. 2), but its energy consumption is 17–70 times higher than RO, making it economically unfeasible. Furthermore, operating AWE at high temperatures raises KOH's vapour pressure (P_v) above that of the feed, causing reverse vapour transport. Pressurised H_2 production is also impossible, as operating above PTFE's liquid entry pressure (LEP, 200–500 kPa) leads to liquid penetration and membrane wetting. Membrane-integrated electrolysis that simultaneously achieves high yield, long-term stability, and practical efficiency remains an unresolved challenge.

We hypothesise that thermal integration can create a symbiotic relationship between the PTFE membrane module and AWE, where each directly solves critical challenges of the other, allowing both to operate under optimal conditions without over-pressure and reverse flux issues. The waste heat from AWE, typically an operational burden requiring costly cooling, perfectly matches MD's massive thermal demand. This synergy transforms two perceived weaknesses into strategic advantages by converting AWE's waste heat into free energy to drive MD water purification, while MD's thermal absorption capacity acts as a heat sink to cool AWE, enabling its stable and efficient operation under controlled temperatures. This approach is expected to create energy-efficient PTFE-AWE that maximises H_2 output, prevents degradation from overheating and salt contamination. Therefore, selecting the optimal MD configuration presents a critical design choice, requiring a comparison among commonly used air gap MD (AGMD), direct contact MD (DCMD), and osmotic MD (OMD) These are distinguished by their permeate side, which consists of a gas layer, a water stream, and an osmotic agent, respectively. The temperature-dependent nature of these processes required systematic investigation of thermal effects on water flux, fouling, and wetting for identifying the optimal integration design.

Here we present a thermally symbiotic OMD-AWE system in which OMD and AWE are connected by self-sustaining thermal loops (Fig. 1), achieving a remarkable seawater H_2 output of 60.2 kg day^{-1} m^{-2}, 4 times higher than $FOWS_{AWE}$, while maintaining stability over 500 h. Such symbiosis is achieved by the strategic selection of OMD (driven by 40°C feed and 6 M KOH osmotic agent) and precisely designed heat exchange loops that capture waste heat from AWE to drive OMD while provide cooling for AWE to maintain its optimal temperatures. This low-temperature OMD operation provided sufficient water flux over 20 L h^{-1} m^{-2} (LHM) for H_2 production while significantly reduced the risks of PTFE membrane scaling and wetting for maintaining excellent salt rejection over time. Its unique dual-driving-force ensures stable water

production that is resilient to latent heat losses during water vapour transport. The thermally symbiotic integration eliminates OMD external heating and active AWE cooling while enabling high-efficiency, salt-free AWE operation, achieving a best-in-class energy consumption of 51.05 kWh kg(H_2)$^{-1}$ and a market-leading cost of \$5.73 kg(H_2)$^{-1}$ comparable to pure water electrolysis. Supported by a scalable and modular design, these findings establish OMD-AWE as a high-performance, cost-competitive, and sustainable solution to water-energy nexus challenges."

Reference:

1. Veroneau, S. S. & Nocera, D. G. Continuous electrochemical water splitting from natural water sources via forward osmosis. *Proceedings of the National Academy of Sciences* **118**, (2021).
2. Scheibel Cassol, G., Shang, C., An, A. K., Khanzada, N. K., Ciucci, F., Manzotti, A., Westerhoff, P., Song, Y. & Ling, L. Ultra-fast green hydrogen production from municipal wastewater by an integrated forward osmosis-alkaline water electrolysis system. *Nat Commun* **15**, 2617 (2024).
3. Rácz, G., Kerker, S., Kovács, Z., Vatai, G., Ebrahimi, M. & Czermak, P. Theoretical and Experimental Approaches of Liquid Entry Pressure Determination in Membrane Distillation Processes. *Periodica Polytechnica Chemical Engineering* **58**, 81–91 (2014).

Comment 2:

Comparing with RO process for desalination, MD and OMD processes can save large energy undoubtedly due to the use of porous hydrophobic membranes. Despite many advantages in membrane technology, challenges remain for MD's large-scale industrial adoption, including high energy consumption, flux decline due to fouling, and economic feasibility. (Water Air Soil Pollut (2025) 236:24)

Reply:

We thank the reviewer for raising these important points regarding the general challenges of MD adoption. We agree that these are critical considerations, and we have addressed each of the three critical barriers in our revised manuscript.

High energy consumption is the primary challenge we solve. As discussed in Comment 1, OMD requires a significant energy input for external heating, which can be 17–70 times higher than RO and would increase the total energy demand by over 20% compared to electrolysis alone. Our thermally symbiotic design eliminates this critical barrier. By using the AWE's waste heat to drive the OMD process, we make the water purification step a nearly zero-external-energy process and continuously supply clean water to AWE. Our thermal modelling confirms that the waste heat from commercial AWE stacks is sufficient to maintain a thermally self-sustaining operation. The only additional electricity required beyond water electrolysis is a minor 1.75 kWh kg(H_2)$^{-1}$ for running pumps. This makes the energy consumption of our OMD-AWE seawater electrolysis directly comparable to that of pure water electrolysis. A detailed discussion of this thermal integration is

presented in the **Revised Manuscript Lines 340–462** (**Thermal self-circulation improves OMD-AWE energy efficiency**) and a full techno-economic analysis (TEA) is presented in **Lines 472–504** (**Comparative techno-economic and environmental assessment**) which confirms that this thermal integration makes OMD-AWE a cost-competitive system.

We agree that membrane fouling and wetting are major challenges that critically affect the long-term stability of any MD-based system. Our work addresses this challenge through a strategic evaluation of the system's operational philosophy. Instead of pursuing the highest possible water flux at elevated temperatures over 60°C for standalone MD purification, our primary goal of the OMD-AWE integration is to ensure the absolute prevention of salt crossover to protect electrolyzer. Therefore, we strategically selected a lower operating temperature of 40°C for OMD operation. This represents a deliberate trade-off, accepting a slightly lower water flux in exchange for significantly enhanced system stability with reduced fouling and wetting issues. This strategy is supported by a comprehensive experimental analysis of the membrane's failure thresholds. Our liquid entry pressure (LEP) tests found that the PTFE membrane's wetting resistance decreased significantly at temperatures above 40°C, while surfactant-accelerated wetting tests and gypsum-accelerated scaling tests both confirmed that the risks of fouling and wetting increase substantially at higher temperatures. The detailed analysis is provided in the **Revised Manuscript Lines 164–220** within the "**Thermal trade-offs between water flux and stability of PTFE membranes**". The success of this operational strategy is validated by our 500-h continuous test of OMD-AWE with real seawater. The stable performance metrics including consistent water flux, stable cell voltage, Faradaic efficiency exceeding 99%, and salt rejection over 99% throughout entire testing period provide evidence of the system's reliability. Furthermore, post-operational SEM and EDX analysis of the membranes and electrodes confirmed their structural and chemical integrity, proving that operating OMD at 40°C is an effective strategy to mitigate fouling and ensure the long-term stability required for integrated electrolysis. A detailed result analysis and discussion about the 500-h test is provided in **Comment 3** and also added to the **Revised Manuscript Lines 285–314.**

The economic challenge is also addressed by the thermally symbiotic design that converts AWE's waste heat to drive OMD while simultaneously providing passive cooling for AWE. By eliminating the energy used for external heating for OMD and active cooling for AWE while enabling high-efficiency, salt-free AWE operation, our thermally symbiotic OMD-AWE seawater electrolysis achieving a best-in-class energy consumption of 51.05 kWh $kg(H_2)^{-1}$ and a market-leading cost of \$5.72 $kg(H_2)^{-1}$, comparable to pure water electrolysis. These are demonstrated through a detailed TEA analysis in the **Revised Manuscript Lines 472–504 Comparative techno-economic and environmental assessment.**

By systematically addressing these barriers, we believe our work provides a compelling and practical pathway for the large-scale industrial adoption of membrane-integrated electrolysis.

Comment 3:

This manuscript has completed a continuous 72 h test of OMD-AWE treating real seawater to demonstrated stable performance, the results is not enough to proof its durability, especial for biofilm fouling with seawater which usually make the original hydrophobic membrane become hydrophilic one.

Reply:

We thank the reviewer for this important comment. We agree that a 72-hour test is insufficient to demonstrate long-term durability. Therefore, we have conducted a 500-h continuous test with real seawater to evaluate membrane performance and electrolyzer stability. Leveraging our strategic 40°C operating temperature for OMD, the OMD-AWE demonstrated a stable water flux of 20.11 ± 0.98 LHM for the first 300 h before gradually declining to 16.67 ± 0.94 LHM by the end of the test, likely due to the initial stages of fouling formation. The electrochemical performance also remained stable with cell voltage stabilised at 1.72 V and Faradaic efficiency remained >99% throughout the entire experiment (Fig. 3c), confirming that neither seawater impurities nor initial fouling compromise the electrolyser kinetics or separator conductivity. Post-operational analysis was conducted to investigate the cause of the water flux decline and to assess component durability using scanning electron microscopy (SEM) and energy-dispersive X-ray spectroscopy (EDX). SEM images revealed thickened morphology of the PTFE membrane after 500 h of use, suggesting slight fouling (Supplementary Fig. 4)[1]. EDX analysis confirmed the presence of organic fouling indicated by an increased carbon content with a reduced F/C atomic ratio from 0.94 to 0.89 (Supplementary Table 5). Localised spots of inorganic scaling composed of magnesium, calcium, and potassium were also identified (Supplementary Fig. 5). While this combination of fouling and scaling might have contributed to the observed water flux reduction, they can be both preventable with antiscalants or basic pre-filtration (e.g., sand filters) and readily removed by standard water, acid or chelating agent washing, as evidenced by our scaling tests. Crucially, these surface deposits did not compromise the membrane's core functionality, as it maintained exceptional ion rejection rates over 99% for all major seawater constituents (Fig. 3d). Analysis of the electrolyser electrodes confirmed their pristine condition and complete protection from seawater contaminants. Both SEM and EDX revealed that the Ni nanowire cathode and NiFe anode retained their structural integrity and original elemental composition (Supplementary Fig. 7 and Supplementary Table 6), with no surface fouling or traces of contaminants such as calcium, magnesium, or silica (Supplementary Fig. 6). The results confirm the system's long-term stability, which is a direct outcome of our strategic decision to operate OMD with mild 40°C feed. This temperature was chosen specifically to mitigate fouling and ensure the reliability required for integrated electrolysis.

We have included the above analysis in the **Revised Manuscript Lines 285–314**, while the main performance results are presented in **Figs. 3c–d**. The supporting SEM and EDX characterizations can be found in **Supplementary Figs. 4–7** and **Supplementary Tables 5–6**.

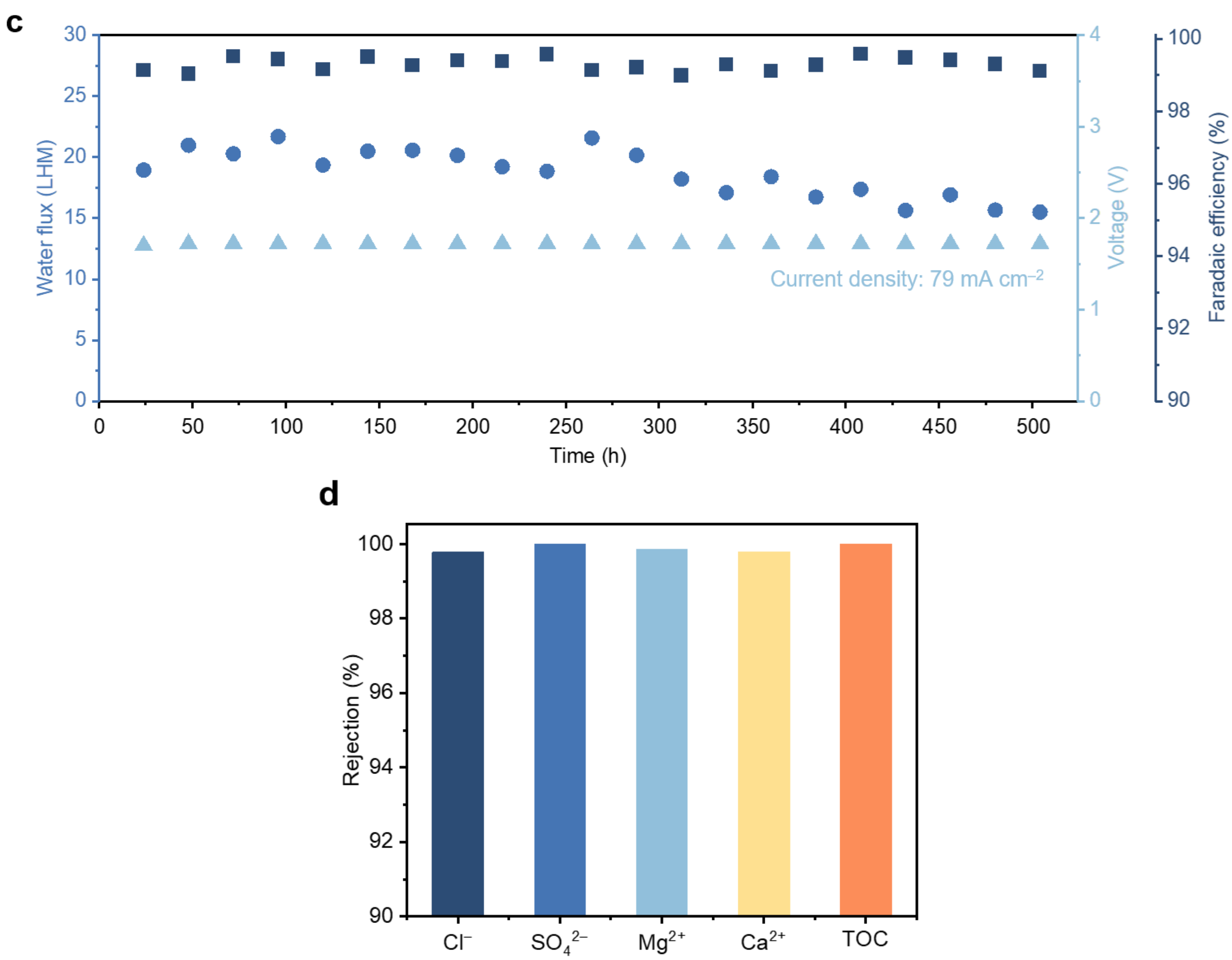

Fig. 3 c A 500-h continuous OMD-AWE operation tracking water flux, cell voltage, and Faradaic efficiency (Conditions: [feed] = seawater, [KOH] = 6 M, T_{feed} = 40°C, T_{KOH} = 20°C); **d** the rejection of chloride (Cl^-), sulfate (SO_4^{2-}), calcium (Ca^{2+}), magnesium (Mg^{2+}), and total organic carbon (TOC) at the end of the 500-h operation

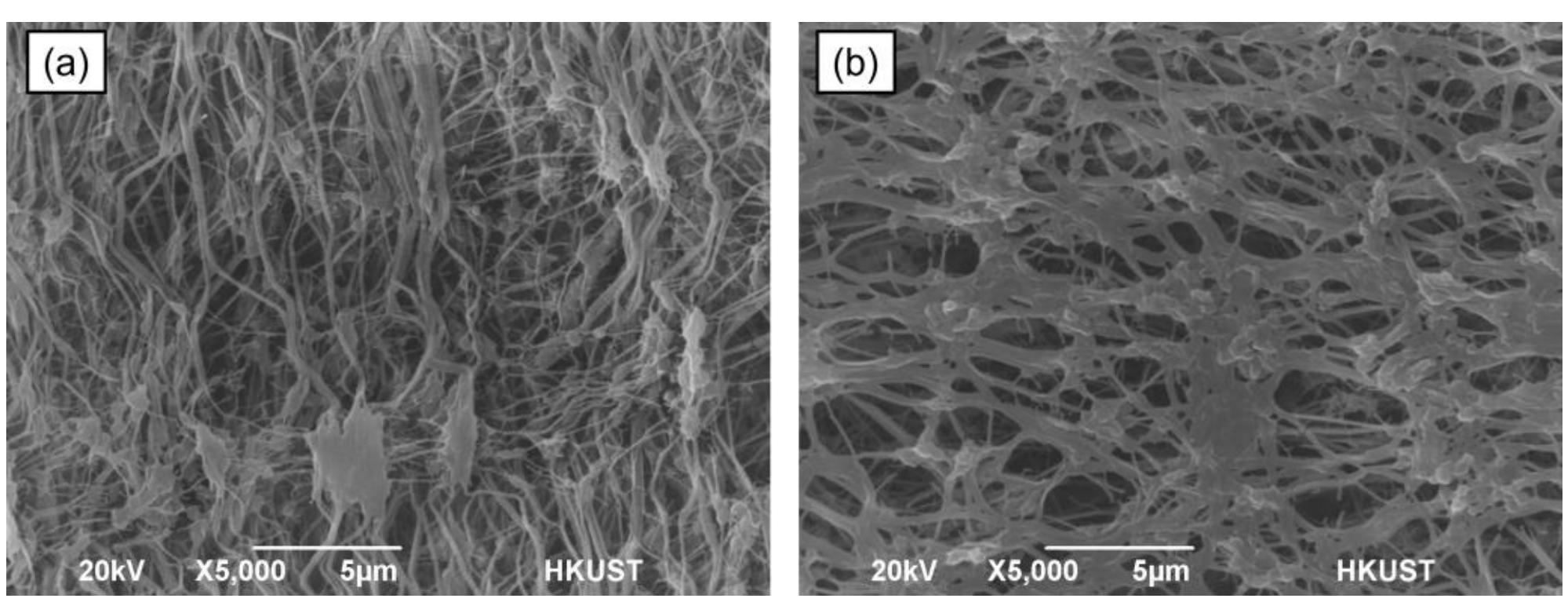

Supplementary Figure 4. SEM images of the PTFE membrane surface morphology. **a** pristine PTFE membranes; and **b** the membrane after the 500-h operation.

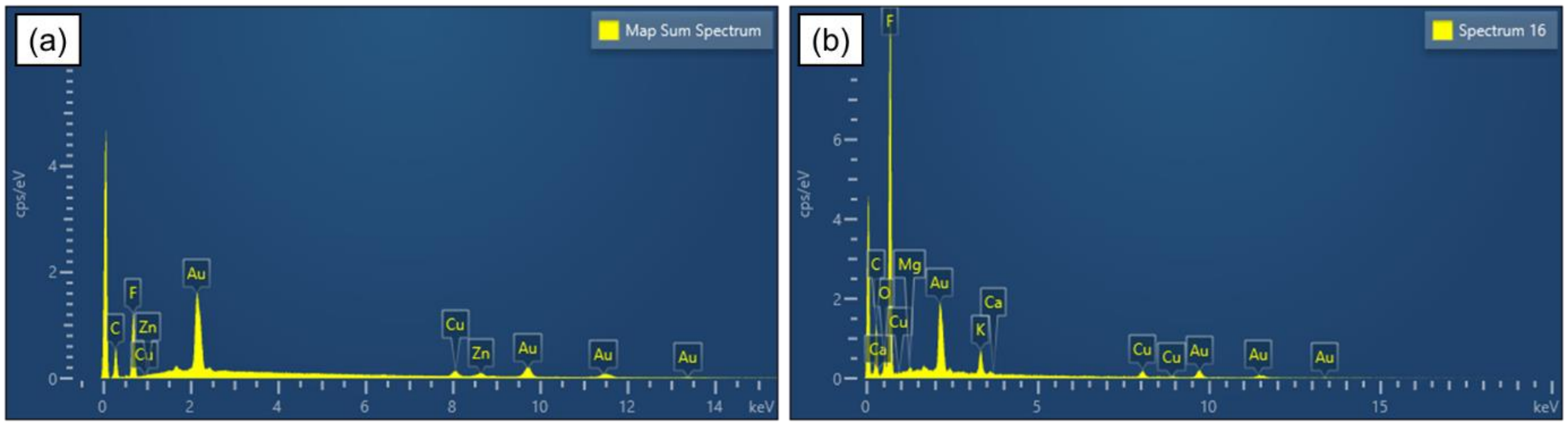

Supplementary Fig. 5 Energy-dispersive X-ray spectroscopy (EDX) spectrum of the PTFE membrane. EDX spectrum showing the surface elemental composition of **a** the pristine PTFE membrane; and **b** the PTFE membrane after 500 h of operation.

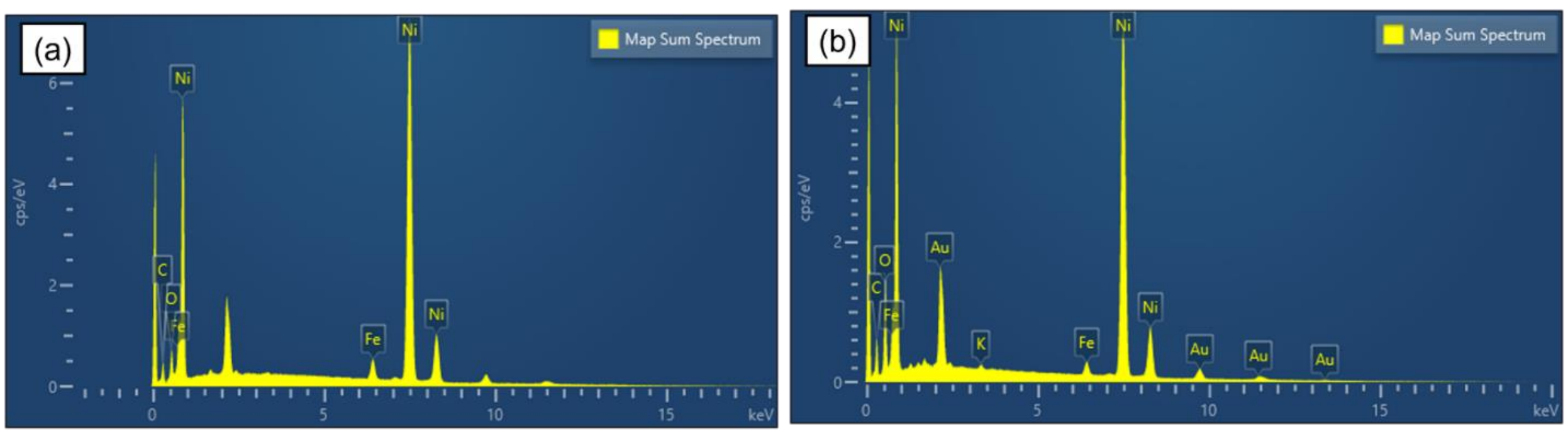

Supplementary Fig. 6 EDX spectrum of the electrodes after the 500-h operation showing no impurities. **a** Ni nanowire cathode and **b** NiFe anode.

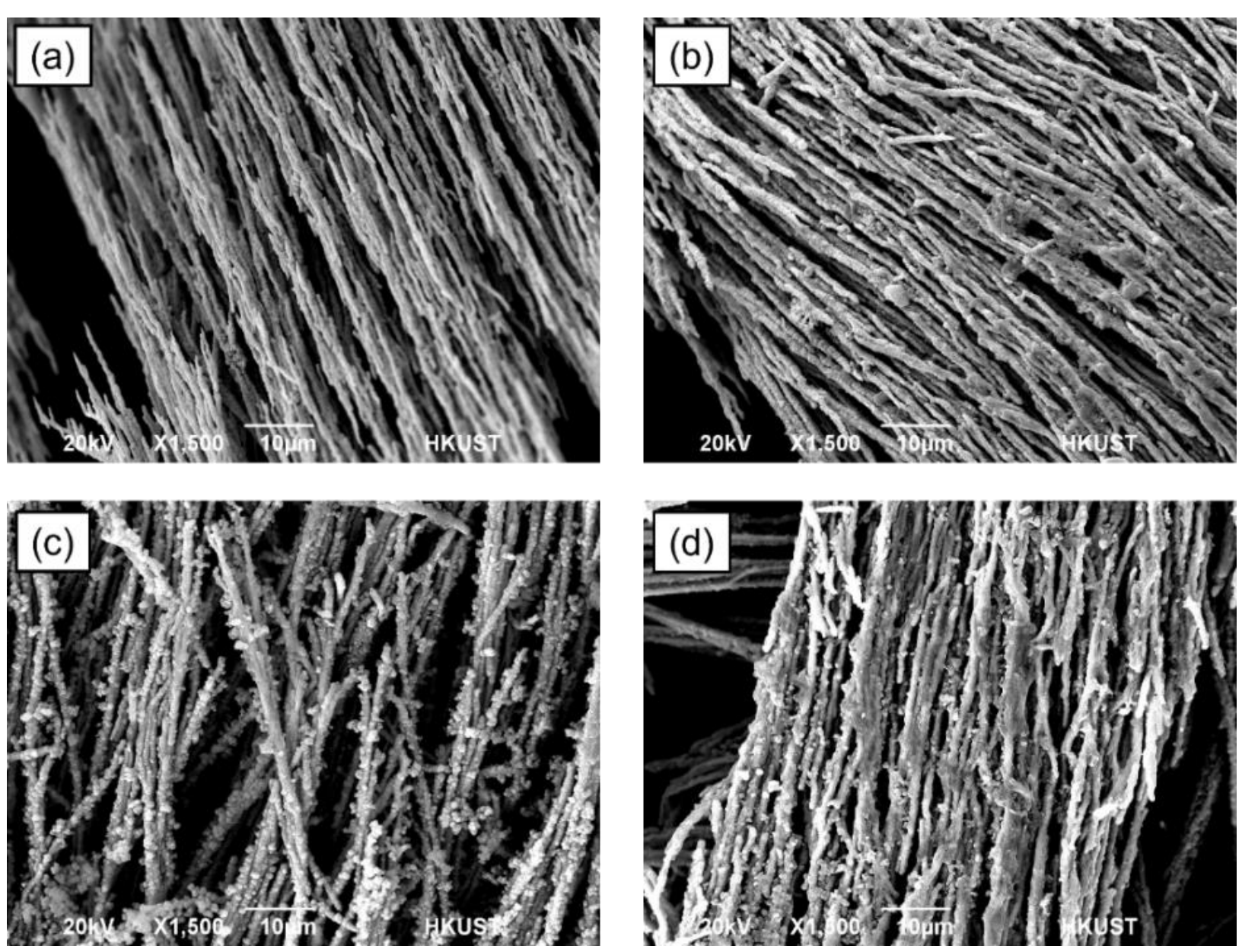

Supplementary Fig. 7 SEM images of the electrodes before and after the 500-h operation. **a** pristine Ni nanowire cathode; **b** Ni nanowire cathode after 500 h operation; **c** pristine NiFe anode; and **d** NiFe anode after 500 h operation.

Supplementary Table 5. EDX surface elemental composition analysis of the PTFE membrane before and after the 500-h continuous operation.

PTFE membrane	Element	Atom (%)
Pristine	C	46.30
	F	49.23
	Others	4.47
	F/C ratio	0.94
After 500 hours	C	49.74
	F	44.33
	Others	5.95
	F/C ratio	0.89

Supplementary Table 6. Surface elemental composition of electrodes before and after 500-h use.

Electrode	Elements	Pristine electrodes Atom (%)	Electrodes after 500 h Atom (%)
Anode	Ni	93.23	92.13
	Fe	6.77	7.86
Cathode	Ni	99.66	96.90
	Fe	0.34	3.10

Reference:

1. Naidu, G., Jeong, S., Kim, S.-J., Kim, I. S. & Vigneswaran, S. Organic fouling behavior in direct contact membrane distillation. *Desalination* **347**, 230–239 (2014).

Comment 4:

The flow rate and heat balance need to be evaluated for OMD-AWE system, heat flux from conduction between the hydrophobic membrane, and that from heat exchanger before electrolyzer.

Reply:

We sincerely thank the reviewer for this critical and insightful comment. The reviewer is absolutely correct that a complete evaluation of the system's heat balance, especially the heat flux across the membrane, was missing from the former manuscript. We recognize that this is a crucial aspect for validating our thermal self-circulation concept, and we appreciate for pointing this omission out.

The heat flow across membrane could be quantified by considering the water vapor transport and thermal conduction of membrane. Except the direct conduction by membranes, water molecule crossing the membrane must first absorb latent heat to vaporise at the hot feed surface, then release this heat upon condensing at the cooler permeate. This brings intrinsic heat transfer that is not an inefficiency but rather the core engine of the process. To determine the heat flow through the PTFE membrane via OMD (Φ_{OMD}), we need to quantify both the heat flow carried by vapour from feed to permeate (Φ_{vapour}) and that by direct membrane conduction (Φ_{mc}). Φ_{vapour} can be determined from our water production as a function of vapor pressure gradient via latent heat of vaporization (ΔH_v, 2400 kJ kg^{-1}). Φ_{mc} depends on the overall heat transfer coefficient (U) and axial temperature gradient (ΔT_{OMD}) along the module length considering counter-current flow. Obtaining Φ_{OMD} allows to determine the temperature changes of feed seawater and KOH osmotic agent (OA) after OMD.

On the other hand, heat exchangers operate entirely through conduction, unlike PTFE membranes primarily relying on latent heat transfer. Their performance depends on specific design parameters (type, area, materials, etc.), and typically, a first-principles calculation (i.e., $\Phi = U \cdot S \cdot LMTD$) is used to design heat exchangers for known heat duties. However, our model's purpose at this stage is to explore the required heat duties and confirm whether AWE waste heat supports thermal self-sustainability, rather than designing specific hardware. Therefore, we employed a standard system-level energy balance approach to determine the conductive heat flow from heat exchangers which equals to the heat lost by the hot fluid stream multiplied by defined heat exchange efficiency, and equals to the heat gained by the cold fluid stream. This energy balance methodology enables us to quickly find out the required conductive heat transfer with controlled temperature setpoint (e.g., the OMD feed at 40°C or AWE outlet at 60°C) for any given flow rates, proving whether viable operational windows exist. Once feasibility is established, heat exchangers can be designed in subsequent engineering phases to meet these defined thermal requirements. We believe this approach is appropriate for a system-level feasibility study.

We appreciate the reviewer for guiding the thermal modelling, and the detailed modelling process is updated in our **Revised Manuscript Thermal self-circulation improves OMD-AWE energy efficiency:**

Lines 352–403: "To assess if the waste heat can sustain thermal self-circulation, we quantify the system's thermal balance as demonstrated in Fig. 4b. The waste heat flow generated from AWE (Φ_{AWE}) due to electrical inefficiency is captured by the KOH stream passing through the electrolyser (flowrate, q_{OA}), increasing its temperature from inlet ($T_{AWE,in}$) to outlet ($T_{AWE,out}$) as expressed in Eq. 4. This captured heat maintains the AWE's optimal operating temperature, with $T_{AWE,out}$ controlled as an engineering setpoint to prevent overheating depended on materials' thermal tolerance (set 60°C in this work).

$$\Phi_{AWE} = SEC_t \left(\frac{1-\gamma}{\gamma}\right) r_{H_2} \eta = c_{KOH} q_{OA} \left(T_{AWE,out} - T_{AWE,in}\right) \quad (4)$$

$$r_{H_2} = c_m\theta\Delta P_v \cdot \rho \frac{M_{H_2}}{M_{H_2O}} S_{OMD} \quad (5)$$

where SEC_t is the theoretical SEC to split water, at 39.4 kWh kg^{-1} based on high heat value (HHV) of H_2, $(1-\gamma)/\gamma$ represents AWE inefficiency, r_{H2} is the H_2 production rate as a function of water flux (determined by c_m, θ, and ΔP_v), water density (ρ), molar mass (M) of H_2 and water, and OMD membrane area (S_{OMD}) (Eq. 5), c_{KOH} is the specific heat capacity of 6 M KOH (3.50 kJ kg^{-1} K^{-1}), and η is the percentage of available waste heat transferred from the AWE stack to the KOH stream, considered 90%. The two heated KOH streams, flowing at equal flowrates, then heat the feed (with flowrate q_{feed} and temperature T_{feed}) from RT to the 40°C target OMD inlet temperature ($T_{OMD,feed,in}$) (Eq. 6), and heat the recirculated KOH leaving the OMD module at $T_{OMD,OA,out}$ to AWE inlet at $T_{AWE,in}$ (Eq. 7).

$$\Phi_{Hx,1} = c_{KOH}\frac{q_{OA}}{2}\left(T_{AWE,out} - T_{cool,1}\right) \cdot \varepsilon = c_{seawater}q_{feed}\left(T_{OMD,feed,in} - T_{feed}\right) \quad (6)$$

$$\Phi_{Hx,2} = c_{KOH}\frac{q_{OA}}{2}\left(T_{AWE,out} - T_{cool,2}\right) \cdot \varepsilon = c_{KOH}q_{OA}\left(T_{AWE,in} - T_{OMD,OA,out}\right) \quad (7)$$

where $c_{seawater}$ is the specific heat capacity of seawater (4.02 kJ kg^{-1} K^{-1}), Φ_{hx} is the heat flow through heat exchangers, T_{cool} is the KOH temperature exiting the heat exchangers, and ε is the heat exchange efficiency (considered 90% for counter-flow). The heated seawater enters OMD and drive water vapour transport, where the heat flow through the membrane via OMD (Φ_{OMD}) consists of the portion carried by vapour (Φ_{vapour}) and that by direct membrane conduction (Φ_{mc}), as expressed in Eqs. 8–10.

$$\Phi_{OMD} = \Phi_{vapour} + \Phi_{mc} = \left(c_m\theta\Delta P_v\Delta H_v + U\Delta T_{OMD}\right) \cdot S_{OMD} \quad (8)$$

$$\Delta T_{OMD} = \frac{\left(T_{OMD,feed,in} - T_{OMD,OA,out}\right) - \left(T_{OMD,feed,out} - T_{OMD,OA,in}\right)}{\ln\left[\left(T_{OMD,feed,in} - T_{OMD,OA,out}\right)/\left(T_{OMD,feed,out} - T_{OMD,OA,in}\right)\right]} \cdot \theta \quad (9)$$

$$\frac{1}{U} = \frac{1}{h_{feed}} + \frac{t}{k_{membrane}} + \frac{1}{h_{OA}} = \frac{1}{h_{feed}} + \frac{t}{k_{PTFE} \cdot \frac{2(1-\varphi) + 3\varphi(k_{air}/k_{PTFE})}{3 - \varphi + 2\varphi(k_{air}/k_{PTFE})}} + \frac{1}{h_{OA}} \quad (10)$$

Φ_{vapour} is proportional to water flux (which is a function of c_m, θ, and ΔP_v) via latent heat of vaporisation (ΔH_v, 2400 kJ kg^{-1}) and OMD's membrane area ($S_{OMD} = 1$ cm^2 with our setup). Φ_{mc} depends on the overall heat transfer coefficient (U) and axial temperature gradient (ΔT_{OMD}) along the module length considering counter-current flow and adjusted by a θ of 0.6 (Eq. 9). U incorporates the convective heat transfer (h) of both feed and OA (set at 1,000 W m^{-2} K^{-1} for laminar flow), and the membrane conductive resistance which equals its thickness (t, 65 µm) divided by effective thermal conductivity ($k_{membrane}$) (Eq. 10). The $k_{membrane}$ accounts for both the conduction through PTFE polymers (k_{PTFE}, 0.25 W m^{-1} K^{-1}) and pore-filling air (k_{air}, 0.026 W m^{-1} K^{-1}) weighted by the membrane porosity (φ, 0.8). Obtaining Φ_{OMD} allows to determine the temperature changes of feed and OA after OMD through Eq. 11.

$$\Phi_{OMD} = c_{seawater} q_{feed} (T_{OMD,feed,in} - T_{OMD,feed,out}) = c_{KOH} q_{OA} (T_{OMD,OA,out} - T_{OMD,OA,in}) \quad (11)$$

Additionally, the OMD feed outlet stream retains heat above RT, which can be utilised for additional clean water production through a secondary DCMD module (process design shown in Supplementary Fig. 10). As the warm stream cools to RT, the released heat flow via DCMD (Φ_{DCMD}) drives surplus water vapour transport and thermal conduction as expressed by Eq. 12. Based on this available heat, we can calculate the required DCMD membrane area (S_{DCMD}) with the corresponding surplus water flux ($J_{w,DCMD}$)."

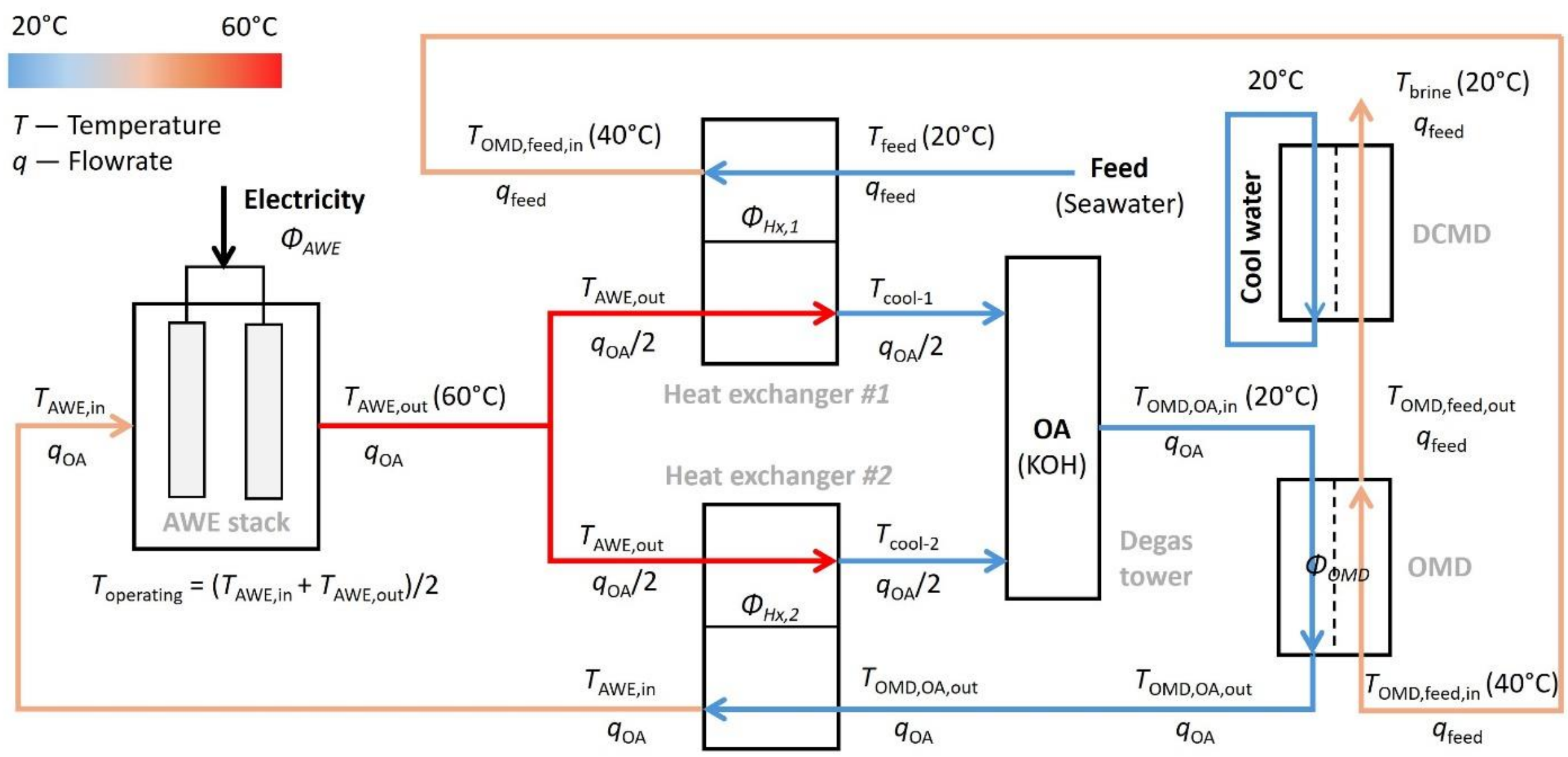

Fig. 4 b Schematic diagram showing thermal balance and fluid flow paths of OMD-AWE, with key temperatures and flowrates indicated at critical points throughout the thermal self-circulation loop.

Supplementary Table 7. Key operating parameters for OMD-AWE thermal self-circulation.

γ	$T_{AWE,in}$	$T_{AWE,out}$	$T_{AWE,avg}$	q_{OA}	q_{feed}	$T_{OMD,OA,out}$	$T_{OMD,feed,out}$	$T_{OMD,feed,in}$	$T_{OMD,OA,in}$
/	°C	°C	°C	kg h^{-1}	kg h^{-1}	°C	°C	°C	°C
0.5	40.1	60	50	0.397	0.346	24.2	35.8	40	20
0.6	40.1	60	50	0.238	0.207	26.2	33.8	40	20
0.7	42.6	60	51.3	0.155	0.135	28.4	31.6	40	20
0.8	44.7	60	52.4	0.079	0.069	32.3	27.7	40	20
0.9	48.3	60	54.2	0.023	0.02	38.8	21.2	40	20

(Condition: S_{OMD} = 1 cm^2)

By solving Eqs. 4–12 with input $T_{OMD,feed,in}$ at 40°C, $T_{AWE,out}$ at 60°C, and both T_{feed} and $T_{OMD,OA,in}$ at 20°C, we established the thermal balance for self-circulation as a function of the AWE stack efficiency (γ) as demonstrated in Figs. 4c–f (key operating parameters are listed in **Supplementary Table 7).** Our revised heat flux modelling, particularly when adding the membrane heat transfer,

yields different results from our previous analysis. Supplementary Fig. 11a illustrates how the heat flow input from the AWE stack, the two heat exchangers, and the OMD module changes with γ of AWE stack. Generally, as γ increases, the overall heat flow from the AWE stack to the other components decreases. At lower efficiencies ($\gamma = 0.5$), the AWE stack provides substantial heat, which the heat exchangers effectively transfer to the feed and recirculating OA stream. However, with increasing γ, the reduced waste heat from AWE leads to a decrease in heat flow through both heat exchangers and thus OMD. This reduced heat supply to the OMD directly results in an inability to maintain a sufficient ΔT_{OMD}, consequently impacting water flux. Supplementary Fig. 11b further details the heat transfer within OMD, revealing that Φ_{vapour} is the dominant mechanism, accounting for over 75% of the total heat flow through the membranes. Consistent with the overall reduction in OMD heat flow, both Φ_{vapour} and Φ_{mc} decrease with increasing γ.

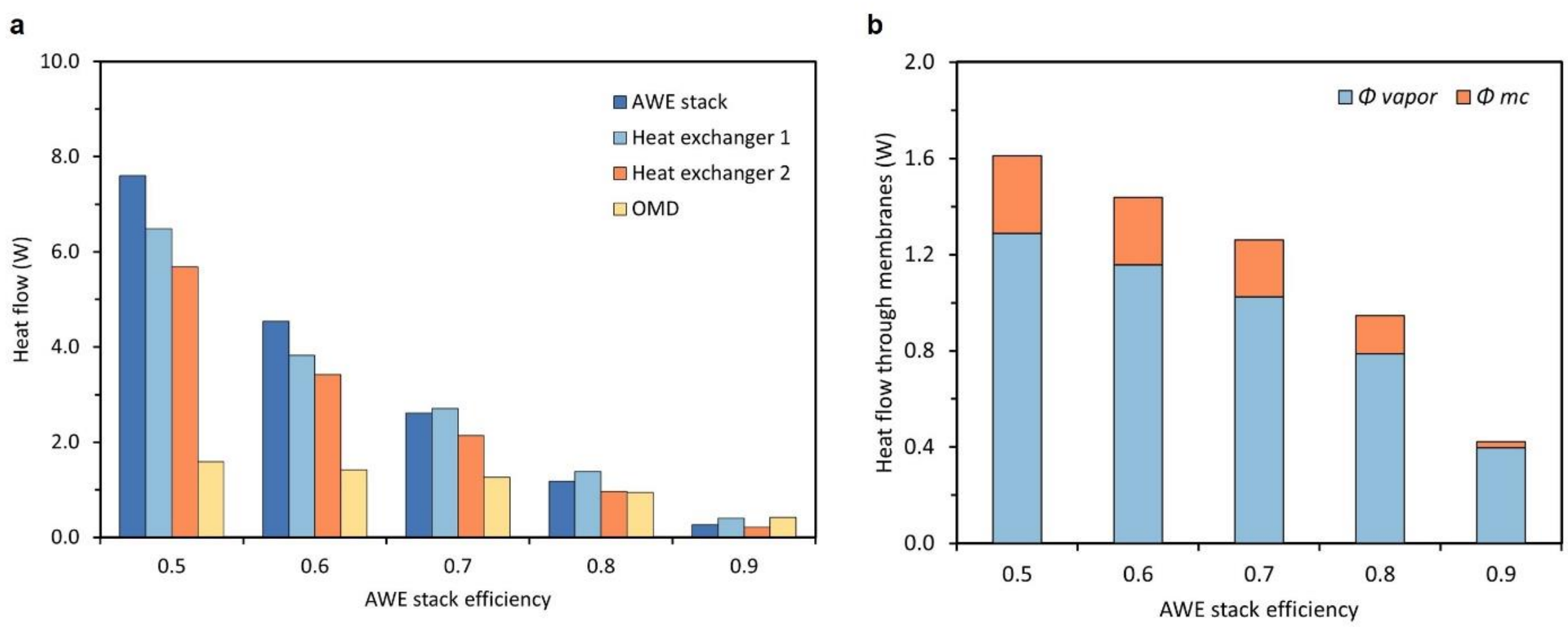

Supplementary Fig. 11. a Heat flow from AWE stack, the two exchangers, and OMD membrane module changed with AWE stack efficiency (γ), and **b** the difference between heat flow from vapor transport (Φ_{vapor}) and that from membrane conduction (Φ_{mc}).

Obtaining the heat flow of each unit enables us to visualize the heat flow profile of the OA stream and feed stream along their respective pathways as shown in Figs. 4c–d. Relative heat flow refers to the heat flow of each stream compared to its initial condition at RT. Starting from the degas tower, the OA stream gains heat from the OMD module and then significantly from the heat exchanger #2 and AWE stack, leading to its peak value. Subsequently, it loses heat as it passes through the two heat exchangers. The remaining residual heat is then dissipated at the degas tower. The feed stream supplied at RT first gains heat from heat exchanger #1, where it recovers heat from the hotter OA stream. It then supplies heat to the OMD module for vapor transport, causing a reduction in its heat flow. The residual heat from feed after OMD could be used for secondary DCMD water production. These profiles demonstrate thermal balance, with heat flow returning to baseline levels at the end of each path, supporting our design's potential for self-circulation.

Achieving the self-circulation depends on creating a well-defined operational window for q_{feed} and q_{OA}. Their flowrates cannot be too high, as the system is constrained by the total amount of waste

heat available from AWE. Exceeding a threshold prevents $T_{AWE,out}$ and $T_{OMD,feed,in}$ from reaching their respective 60°C and 40°C setpoints. On the other hand, their flowrates cannot be too low. A lower q_{OA} increases its residence time in the AWE cell, causing OA to absorb excessive heat beyond the materials' thermal tolerance. Similarly, a lower q_{feed} increases residence time in heat exchangers, causing feed temperature to exceed the optimal 40°C and creating fouling and wetting risks. While reducing the heat exchange area could control the feed at 40°C, the resulting insufficient thermal load would be quickly consumed by vaporisation, making ΔT_{OMD} to collapse and thus compromising water flux for H_2 production or, more likely, lead to a complete system failure. Therefore, such trade-off reveals optimal q_{feed} and q_{OA} and their corresponding RRs (Fig. 4e). Note that these flowrates are reported for a 1 cm^2 membrane area and scale linearly with membrane size. A less efficient AWE stack (γ = 0.5) provides abundant waste heat, allowing for higher flowrates that effectively maintain ΔT_{OMD} which is slightly reduced from the ideal 20.0°C to 15.8°C (Fig. 4f), resulting in an acceptable 12.3% water flux penalty compared to the ideal 22.44 LHM (Fig. 4e). Conversely, a highly efficient AWE (γ = 0.9) produces less waste heat, forcing the system to operate at 94.0% lower flowrates (Fig. 4e) which make OMD highly susceptible to temperature changes, reducing ΔT_{OMD} to 1.2°C and limiting the water flux to only 6.0 LHM (Figs. 4f). These findings validate the effectiveness of our design and suggest a significant opportunity for current commercial AWE stacks, which typically operate at γ of 0.65–0.80. Within this real-world range, our system is thermally self-sustaining while maintaining a low water flux penalty. Even at high γ of 0.9, the thermally symbiotic OMD-AWE still achieves 5.6 times higher H_2 yields than the isothermal SDE-DSE.

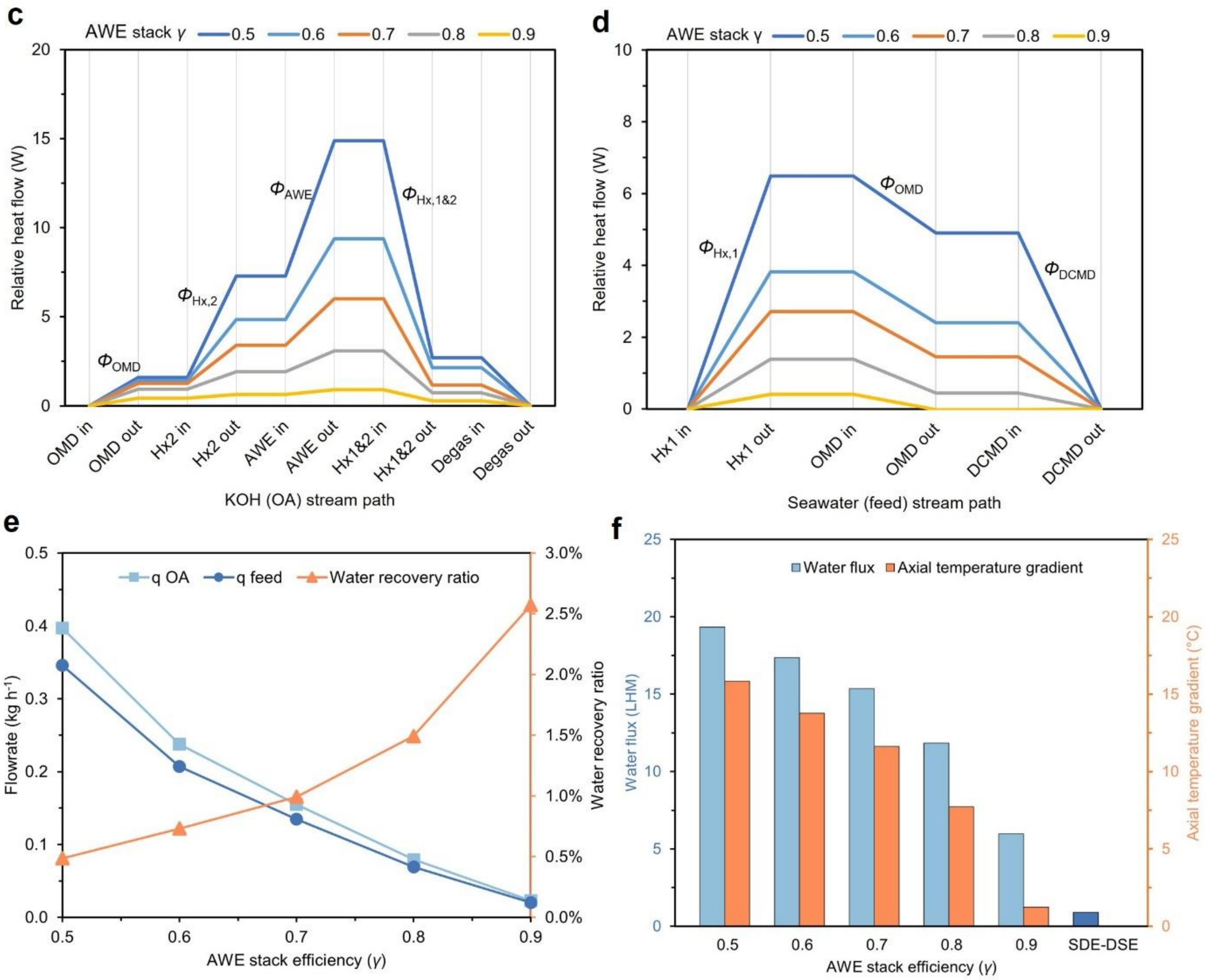

Fig. 4 c–**d** Relative heat flux of OA and feed streams compared to their initial RT conditions when circulating within their respective pathways, **e.** q_{feed}, q_{OA}, and water recovery ratio as a function of AWE stack γ, and **d**. the actual water flux and ΔT_{OMD} as a function of γ.

The above newly obtained results from the updated model and discussion are added to the **Revised Manuscript Lines 404–462**, including corresponding figures and tables in both main texts and supplementary.

Responses to comments of Reviewer 4:

The manuscript demonstrates that a hydrophobic PTFE based osmotic membrane–distillation (OMD) module, thermally coupled to an alkaline water electrolysis (AWE) stack, delivers a record hydrogen flux of 52.7 kg day^{-1} m^{-2} while rejecting salts almost completely. Although this flux greatly exceeds that of prior PTFE/OD systems, the advance appears incremental rather than conceptual because (i) PTFE mediated vapor phase desalination is well established; (ii) recovery of low grade electrolyzer heat has already been modelled; and (iii) no techno economic, life cycle, or >100 h durability data are provided to show competitiveness against RO + AWE or FO AWE at scale. Consequently, the novelty and impact fall short of the usual Nat Commun standard. The specific issues below, if addressed, could significantly strengthen the work.

Reply:

We thank the reviewer for these insightful comments, which challenged us to better articulate the conceptual novelty of our work. We have undertaken a major revision to address the core concern that the advance appeared incremental. The manuscript is now reframed to highlight our core innovation: a new design philosophy of thermal symbiosis achieved from several counter-intuitive operational principles, representing a genuine departure from conventional thinking. We have also performed the substantial new work to prove the practical viability of our system. The revised manuscript now includes a comprehensive membrane wetting/fouling evalution, a 500-h continous test, a full Techno-Economic Analysis and Life Cycle Assessment, demonstrating our system's stability and competitiveness against established benchmark.

Given the extensive revisions, we have provided concise summaries of the key changes in our point-by-point response. For detailed explanations and the new supporting data, we respectfully refer the reviewer to the full revised manuscript, where all major changes have been highlighted in blue. We believe the work now represents a significant conceptual advance that meets the high standards of novelty and impact expected by Nature Communications.

Comment 1:

Clarify the genuine transport advance (pp. 11–13) — Heating the feed to raise ΔPv in PTFE OMD is intuitive and widely practised in membrane distillation desalination. The manuscript should identify any new physical insight beyond this well-known strategy.

Reply:

We sincerely thank the reviewers for their valuable comments, which have given us the opportunity to clarify the core innovation of our work. We agree that heating the feed in MD is a known principle. The genuine advance and new physical insights of our work lie not in the components themselves, but in a new design philosophy for co-optimizing water purification and electrolysis within a thermally symbiotic system. This new philosophy leads to three counter-intuitive design

principles that directly reverse the conventional wisdom of standalone membrane processing. It is this conceptual shift, rather than simple optimization, that enabled us to overcome the limitations of previous systems and achieve breakthrough performance.

To highlight the novelty of our work, we first provide a direct comparison with the current state-of-the-art system for in situ seawater electrolysis, as reported in *Nature* 612, 673–678 (2022). Their system combines isothermal OD with AWE in a physically coupled design for simultaneous water purification and electrolysis[1]. While its vapor transport mechanism through hydrophobic PTFE membranes ensures complete salt rejection and long-term stability, the limited osmotic gradients result in insufficient water production that constrains H_2 output. More critically, this direct coupling creates inherent constraints for optimization and scaling, as optimal conditions for PTFE membrane's water vapor transport and AWE are not allowed. For instance, AWE can only operate at room temperatures rather than its optimal 60–90°C, since increasing the electrolyte temperature leads to reverse vapor transport due to higher vapor pressure than feed. Pressurized H_2 production is also impossible as operating above PTFE membrane's liquid entry pressure (LEP, 200–500 kPa[2]) causes membrane wetting and compromises salt rejection. Although heating the feed to introduce a thermal gradient can enhance water flux, this approach requires 17–70 times more energy than conventional reverse osmosis (RO). Scaling up this system would require substantial increases in membrane areas and energy input, creating significant barriers to economic viability. **Our key innovation is a thermal integration design** that physically separates the PTFE membrane module from the AWE electrolyzer while maintaining their thermal connection for thermally symbiotic operation. This thermal integration converts AWE's waste heat, which is an operational burden requiring costly cooling, into a valuable asset that drives water vapor transport through OMD using the PTFE membrane. In return, the OMD provides the AWE with both passive cooling and a continuous supply of high-purity water. This allows both OMD and AWE to operate under their respective optimal conditions simultaneously, without over-pressure and reverse flux issues. Due to the enhanced water production and the AWE's optimal temperature operation, this system delivered record-high 60.2 kg day^{-1} m^{-2} of H_2 production rate that is 25 times higher than that from the Nature paper, while maintaining stability for 500-h continuous operation with superior energy and cost metrics. We have revised the manuscript, **Introduction and Abstract**, to clarify that our core innovation lies not in membrane technology optimization, but in system-level design.

The key to achieve this thermal symbiosis is three **"counter-intuitive" operational principles** of membranes that reverse the conventional wisdom of standalone water purification. These principles are tailored specifically to the demands of an integrated electrolyzer, prioritizing system-level performance over component-level optimization.

1. NOT "The hotter, the better". Conventional MD-based water systems follow a "hotter is better" principle, typically operating over 60°C to maximize water flux and water recovery[3–5]. However, such high temperatures increase membrane scaling, wetting risks, and potential salt crossover. While these risks are manageable in drinking water production, where certain levels of benign salts are acceptable and temporary shutdowns for membrane maintenance are feasible, the requirements

change when purifying water for electrolyzer that has zero tolerance for salts. Even a minor wetting event that would be manageable in water treatment can introduce sufficient salt to cause irreversible damage to electrolyzer through corrosion and catalyst degradation. Any cost saved by pushing for a higher water flux and recovery is paid for dearly in catastrophic damage to the far more expensive electrolyzer. This fundamental economic and operational trade-off completely redefines the process optimum. The goal is no longer maximizing flux at any cost, but ensuring the absolute, long-term stability of the entire system. In this work, we identified 40°C OMD temperature for AWE integration, rather than pursuing maximum possible temperatures, maintaining sufficient water flux while operating safely below membrane failure thresholds. Supporting data from water flux measurements, LEP tests, and wetting/scaling tests confirming this 40°C temperature choice are detailed in **Revised Manuscript Lines 121–220** **Thermal trade-offs between water flux and stability of PTFE membranes**.

2. MD's high energy demand is NOT weakness. Our second design principle challenges the perception that MD's massive thermal energy demand is an economic weakness. This view is only correct when analysing the system in isolation. For a standalone OMD unit, producing 1 kg of H_2 would require an additional at least 10.6 kWh of external heating for water, corresponding to a ~23% energy penalty that makes the concept economically unviable. However, our thermal symbiotic design reveals that this high energy demand is not a weakness, but the key to unlocking a perfect thermal synergy. The AWE's waste heat is an energy liability requiring costly cooling, and the OMD's high thermal demand are not two separate problems, but a single, elegant solution. Our thermal self-circulation design creates a perfect match between them, converting the AWE's waste heat into the primary driver for the OMD process, simultaneously providing passive cooling for the electrolyser. By eliminating the external heating and cooling requirement, this design makes our seawater electrolysis system as energy-efficient as pure water electrolysis. The modelling and discussion about the use of AWE waste heat are provided in **Revised Manuscript Lines 340–462** **Thermal self-circulation improves OMD-AWE energy efficiency**, and the related energy and cost analysis compared to other competing systems is presented in **Manuscript Lines 472–504** **Comparative techno-economic and environmental assessment**.

3. LOW water recovery is better. Perhaps the most significant departure from conventional wisdom is our third design principle: using a low water recovery to unlock the system full potential. Standalone membrane systems are constrained by the high costs of water pretreatment, extensive heating and pumping energy, and brine disposal, forcing them to prioritize high recovery to be economically viable. This locks them into a low-flowrate regime that is incapable of absorbing and productively using a large-scale, low-grade heat source like an electrolyzer's waste heat. Our system design inverts this paradigm. By identifying the AWE's waste heat as the true limiting resource, we optimize for system-level energy efficiency instead of water resource efficiency. We employed a low-recovery (<2.5%), high-flowrate loop that acts as a powerful heat transfer engine. This high flow rate efficiently captures the AWE's waste heat and creates a large thermal reservoir to maintain OMD's thermal driving force. This approach not only enables a higher, more stable

flux with a smaller membrane footprint but also yields the critical secondary benefit of simplified, environmentally safe brine management. This insight is discussed in **Revised Manuscript Lines 417–462.**

We have summarized these new insights in the **Revised Manuscript** Conclusion:

Lines 519–526: "We present a thermally symbiotic OMD-AWE system to produce green H_2 directly from seawater. The system is designed under a new philosophy that reverses conventional membrane principles: i) a milder temperature is optimal for stability, ii) high energy demand is an asset rather than a liability, enabling full absorption and utilization of electrolyser waste heat, and iii) low water recovery creates a powerful heat transfer engine for high, stable water flux. The convergence of these three counter-intuitive principles enables a record-high H_2 production rate with excellent operational stability over 500 h, offering a cost-competitive and sustainable pathway for direct seawater electrolysis."

References:

1. Xie, H., Zhao, Z., Liu, T., Wu, Y., Lan, C., Jiang, W., Zhu, L., Wang, Y., Yang, D. & Shao, Z. A membrane-based seawater electrolyser for hydrogen generation. *Nature* **612**, 673–678 (2022).
2. Rácz, G., Kerker, S., Kovács, Z., Vatai, G., Ebrahimi, M. & Czermak, P. Theoretical and Experimental Approaches of Liquid Entry Pressure Determination in Membrane Distillation Processes. *Periodica Polytechnica Chemical Engineering* **58**, 81–91 (2014).
3. Lawson, K. W. & Lloyd, D. R. Membrane distillation. *Journal of Membrane Science* **124,** 1–25 (1997).
4. Kim, HW., Jang, A. & Jeong, S. Operational strategy preventing scaling and wetting in an intermittent membrane distillation process. *npj Clean Water* **6**, 50 (2023).
5. López-Porfiri, P., Ramos-Paredes, S., Núñez, P. *et al.* Towards the technological maturity of membrane distillation: the MD module performance curve. *npj Clean Water* **6**, 18 (2023).

Comment 2:
Provide a full techno economic comparison — Include levelised cost of hydrogen (LCOH), CAPEX/OPEX breakdowns, and life cycle (LCA) metrics versus (i) OMD AWE, (ii) FO AWE (Nat Commun 15, 2617, 2024), (iii) RO + EDI + AWE, and (iv) direct seawater electrolysis. Recent MD AWE TEA work already reports 1.3 US$ kg^{-1} H_2; the present study must benchmark against this figure.

Reply:

We thank the reviewer for this valuable suggestion. In response, we have now developed a detailed techno-economic analysis (TEA) and life cycle assessment (LCA) comparing our OMD-AWE process with the following seawater-to-hydrogen systems: (i) a conventional RO and ion exchange (IEX)-pretreated AWE (RO+IEX-AWE), (ii) $FOWS_{AWE}$ (*Nat. Commun.* 15, 2617, 2024), (iii) the

self-dampening electrolyte-assisted direct seawater electrolysis (SDE-DSE, from *Nature* 612, 673–678, 2022, which is also called OD-AWE in our work), and (iv) direct electrolysis of untreated seawater (DSE)[3].

Regarding the benchmark figure of 1.3 US$ kg^{-1} H_2 from recent MD-AWE TEA work[4], we note a key methodological difference. That study incorporates a return on investment (ROI) analysis based on the revenue from selling H_2, but does not disclose the assumed H_2 selling price. To ensure a transparent and directly comparable assessment of production costs, we have adhered to the conventional TEA methodology, which reports the LCOH based solely on the costs of production, without incorporating revenue from product sales.

The detailed analysis and discussion is now available in our **Revised Manuscript** **Comparative techno-economic and environmental assessment**:

Lines 472–504: "The thermally symbiotic OMD-AWE offers a cost-competitive and sustainable pathway for seawater H_2 production, supported by a comprehensive analysis of its energy consumption, techno-economic analysis (TEA), and life-cycle assessment (LCA) as shown in Fig. 5 (details see Supplementary Notes 3–4). The OMD-AWE was compared against four competing systems: i) conventional RO and ion exchange (IEX)-pretreated AWE (RO+IEX-AWE), ii) $FOWS_{AWE}$, iii) SDE-DSE, and iv) direct electrolysis of untreated seawater (DSE). As shown in Fig. 5a, OMD-AWE achieves the lowest SEC of 51.05 kWh $kg(H_2)^{-1}$ due to the near-zero energy burden for water purification and the elimination of external active cooling systems from our thermally symbiotic design. The only electricity addition beyond water splitting is 1.75 kWh $kg(H_2)^{-1}$ from electricity balance-of-plant (BoP) for running pumps, making it even competitive with commercial pure-water electrolysis (51.1–66.7 kWh $kg(H_2)^{-1}$). Other four systems consume around 3 times more BoP electricity on cooling, while $FOWS_{AWE}$ and SDE-DSE requires 7% and 14% more electricity for water splitting due to the FO membrane's intolerance to KOH concentrations above 1 M and temperature constraints, respectively. Since energy represents the primary operational cost. OMD-AWE is identified as the most cost-effective choice, revealed by TEA which uses the levelised cost of H_2 (LCOH) as the objective function. It shows the lowest LCOH of \$5.73 $kg(H_2)^{-1}$, remaining 6.3%, 12.2%, 17.8%, and 52.8% lower than RO+IEX-AWE, $FOWS_{AWE}$, SDE-DSE, and DSE, respectively (Fig. 5b). Beyond the energy savings, the LCOH for $FOWS_{AWE}$ could be underestimated, as potential Cl^- crossover from seawater could damage the electrolyser and reduce its lifetime. The comparison with DSE highlights the critical importance of durability. The prohibitively high LCOH of DSE is driven by high capital costs (e.g., noble metal catalysts), operation and maintenance (O&M), and a shortened system lifetime that necessitates frequent component replacement. The energy and cost efficiency of OMD-AWE yield comprehensive environmental benefits as demonstrated by LCA (Fig. 5c). When compared to the conventional RO+IEX-AWE and $FOWS_{AWE}$, it generates 5–11% and 4–6% lower environmental impacts, respectively. The reductions are even greater when compared to SDE-DSE (10–37%), including the reductions in global warming potential (15%), marine eutrophication (18%), and stratospheric ozone depletion (37%), benefited from energy-efficient operating conditions and

smaller membrane areas due to high water flux. The most substantial improvement is seen against DSE with impacts reduced by 17–660%, as our system avoids inherent inefficiency, operational complexity, and the environmental burden of noble metal catalysts."

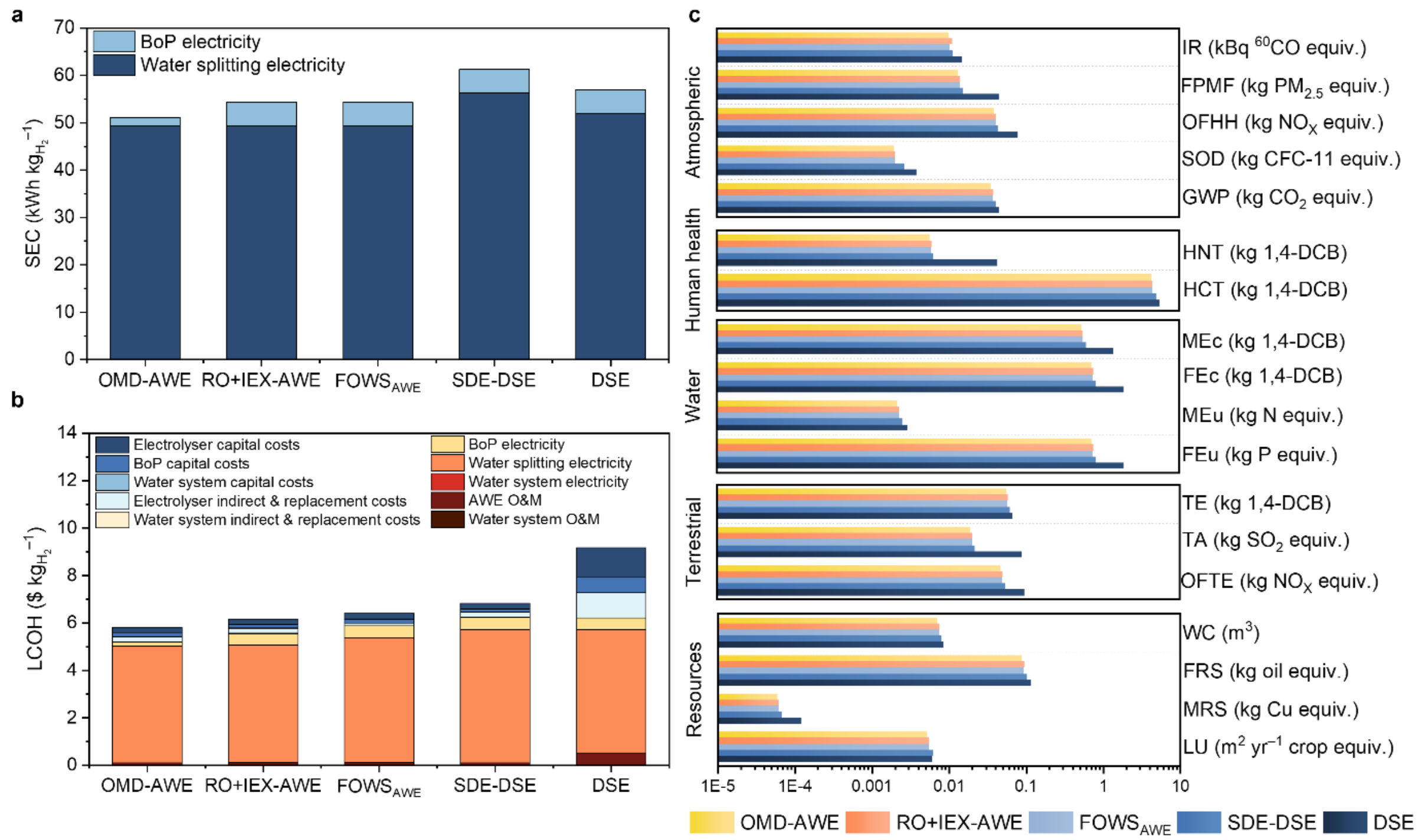

Fig. 5 Techno-economic and environmental assessment of OMD-AWE. a The comparison in SEC between OMD-AWE and other seawater-to-hydrogen systems: RO+IEX-AWE, $FOWS_{AWE}$, SDE-DSE, DSE; **b** Breakdown of LCOH from OMD-AWE compared against the other four competing systems; and **c** the life cycle environmental impacts (IR, ionising radiation; FPMF, fine particulate matter formation; OFHH, ozone formation–human health; SOD, stratospheric ozone depletion; SOD, stratospheric ozone depletion; GWP, global warming potential; HNT, human non-carcinogenic toxicity; HCT, human carcinogenic toxicity; MEc, marine ecotoxicity; FEc, freshwater ecotoxicity; MEu, marine eutrophication; FEu, freshwater eutrophication; TE, terrestrial ecotoxicity; TA, terrestrial acidification; OFTE, ozone formation–terrestrial ecosystems; WC, water consumption; FRS, fossil resource scarcity; MRS, mineral resource scarcity LU, land use).

The methodology of TEA and LCA are provided in **Supplementary Note 3 and 4**, respectively.

Reference:

1. Scheibel Cassol, G., Shang, C., An, A. K., Khanzada, N. K., Ciucci, F., Manzotti, A., Westerhoff, P., Song, Y. & Ling, L. Ultra-fast green hydrogen production from municipal wastewater by an integrated forward osmosis-alkaline water electrolysis system. *Nat Commun* **15**, 2617 (2024).

2. Xie, H., Zhao, Z., Liu, T., Wu, Y., Lan, C., Jiang, W., Zhu, L., Wang, Y., Yang, D. & Shao, Z. A membrane-based seawater electrolyser for hydrogen generation. *Nature* **612**, 673–678 (2022)
3. Yu, L., Ning, M., Wang, Y., Yuan, C. & Ren, Z. Direct seawater electrolysis for hydrogen production. *Nat Rev Mater* (2025).
4. Xu, X., Zhao, Z., Song, C., Xu, L., & Zhang, W. Seawater Membrane Distillation Coupled with Alkaline Water Electrolysis for Hydrogen Production: Parameter Influence and Techno-Economic Analysis. *Membranes* **15**, 60 (2025).

Comment 3:
Demonstrate long term stability — The current 72 h test is insufficient. Please provide ≥500 h continuous operation on real seawater, tracking water flux, cell voltage, Faradaic efficiency and post mortem SEM/EDX of both membrane and electrodes to substantiate durability claims.

Reply:

We thank the reviewer for this important comment. We agree that a 72-hour test is insufficient to demonstrate long-term durability. Therefore, we have conducted a 500-hour continuous test with untreated real seawater to evaluate membrane and electrolyzer stability. As shown in Fig. 3c, the system demonstrated robust operation: the water flux remained stable for over 300 hours before a gradual decline due to to initial fouling (Supplementary Fig. 4), while the cell voltage stabilized at 1.72 V and Faradaic efficiency remained >99% throughout the entire experiment. Furthermore, the integrated OMD-AWE system maintained a stable 6 M KOH draw solution concentration throughout the 500-hour test (Supplementary Fig. 8).

Post-test analysis confirmed that the water flux decline was caused by reversible surface fouling and scaling on the PTFE membrane, which can be mitigated with standard cleaning protocols. Crucially, this fouling did not compromise the membrane's exceptional ion rejection (>99%) as shown in Fig. 3d or the electrolyzer's integrity. Both SEM and EDX revealed that the Ni cathode and NiFe anode retained their structural integrity and original elemental composition (Supplementary Fig. 7 and Supplementary Table 6), with no surface fouling or traces of contaminants such as calcium, magnesium, or silica (Supplementary Fig. 6).

The 500-h continuous operation test and related analyse are added in the **Revised Manuscript** and corresponding Supplementary figures and tables:

Lines 285–314: "The long-term stability of OMD-AWE was evaluated through a 500-h continuous test with real seawater as shown in Fig. 3c. The OMD process demonstrated a stable water flux of 20.11 ± 0.98 LHM for the first 300 h before gradually declining to 16.67 ± 0.94 LHM by the end of the test, likely due to the initial stages of fouling formation. The electrochemical performance also remained stable with cell voltage stabilised at 1.72 V and Faradaic efficiency remained >99% throughout the entire experiment, confirming that neither seawater impurities nor initial fouling

compromise the electrolyser kinetics or separator conductivity. Post-operational analysis was conducted to investigate the cause of the water flux decline and to assess component durability using scanning electron microscopy (SEM) and energy-dispersive X-ray spectroscopy (EDX). The PTFE membrane exhibited signs of surface fouling after 500 h of use, showing a thickened morphology in SEM images (Supplementary Fig. 4). EDX analysis confirmed the presence of organic fouling indicated by an increased carbon content with a reduced F/C atomic ratio from 0.94 to 0.89 (Supplementary Table 5). Localised spots of inorganic scaling composed of magnesium, calcium, and potassium were also identified (Supplementary Fig. 5). While this combination of fouling and scaling might have contributed to the observed water flux reduction, they can be both preventable with antiscalants or basic pre-filtration (e.g., sand filters) and readily removed by standard water, acid or chelating agent washing. Crucially, these surface deposits did not compromise the membrane's core functionality, as it maintained exceptional ion rejection rates over 99% for all major seawater constituents (Fig. 3d). Analysis of the electrolyser electrodes confirmed their pristine condition and complete protection from seawater contaminants. Both SEM and EDX revealed that the Ni nanowire cathode and NiFe anode retained their structural integrity and original elemental composition, with no surface fouling or traces of contaminants such as calcium, magnesium, or silica (Supplementary Figs. 6–7, Supplementary Table 6). The integrated OMD-AWE system also maintained a stable concentration for the KOH osmotic agent throughout the 500-h test (Supplementary Fig. 8). In addition, our physically decoupled modular design provides crucial operational flexibility. This allows either the OMD or AWE to be taken offline for routine maintenance (e.g., membrane cleaning) without system failure. The ability to handle these interruptions and return to stable operation is essential for robust, long-term operation beyond 500 h during real-world applications."

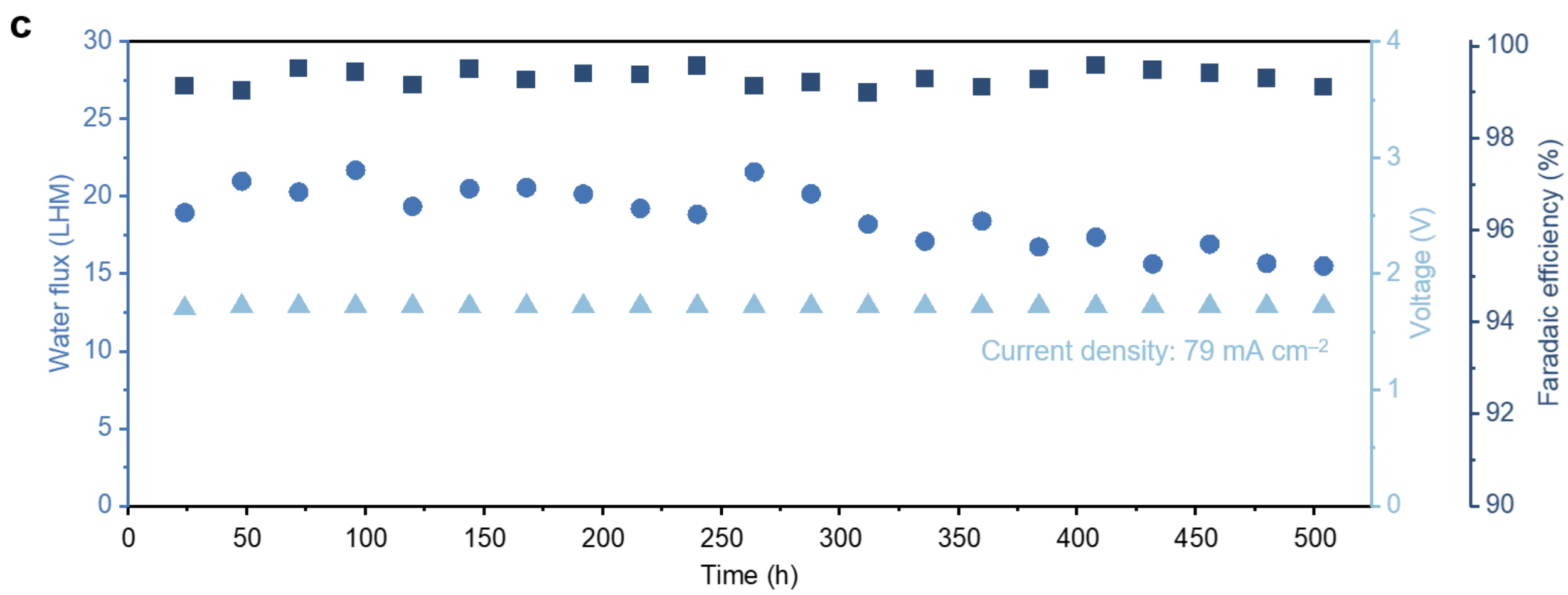

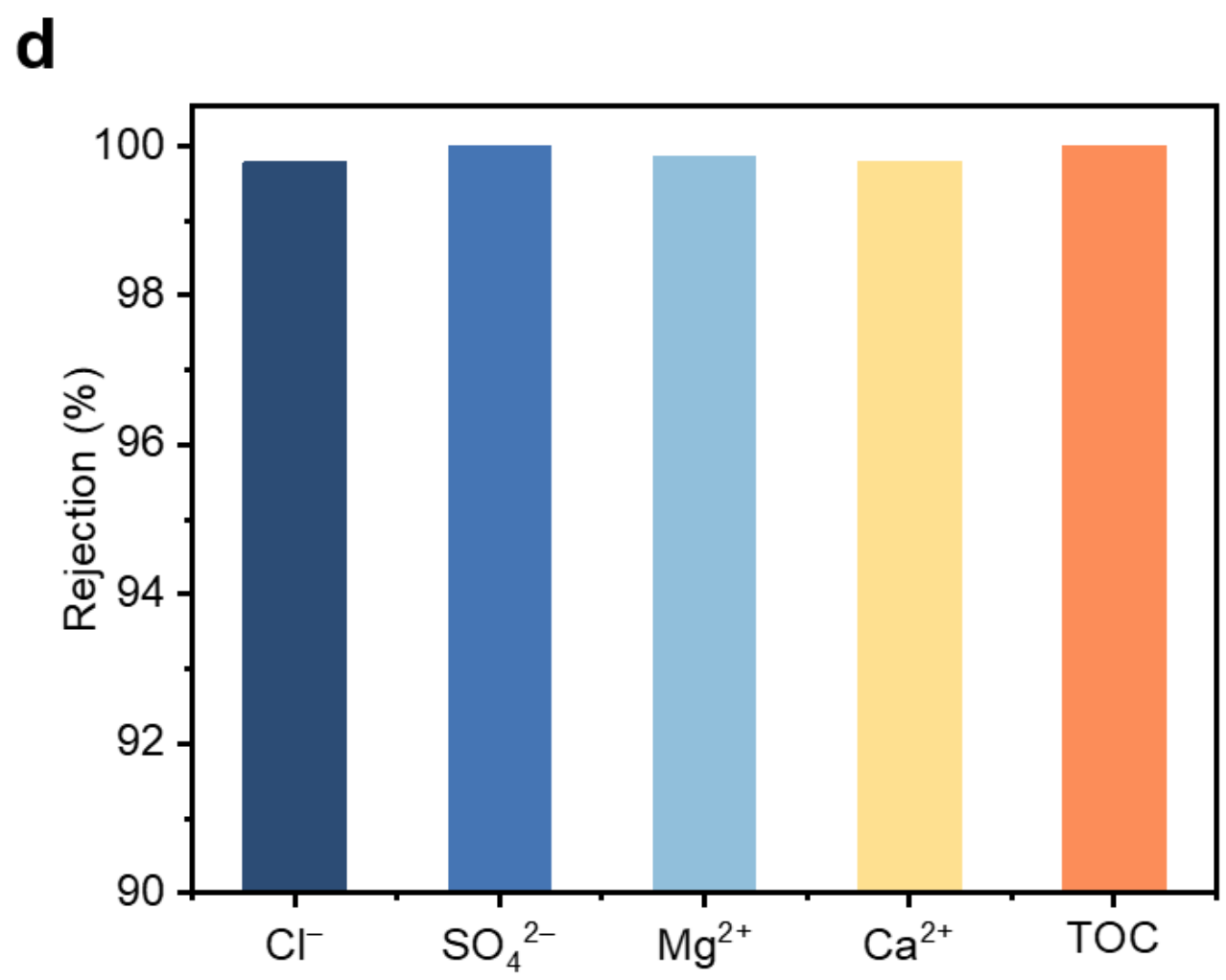

Fig. 3 c A 500-h continuous OMD-AWE operation tracking water flux, cell voltage, and Faradaic efficiency; **d** the rejection of chloride (Cl^-), sulfate (SO_4^{2-}), calcium (Ca^{2+}), magnesium (Mg^{2+}), and total organic carbon (TOC) at the end of the 500-h operation

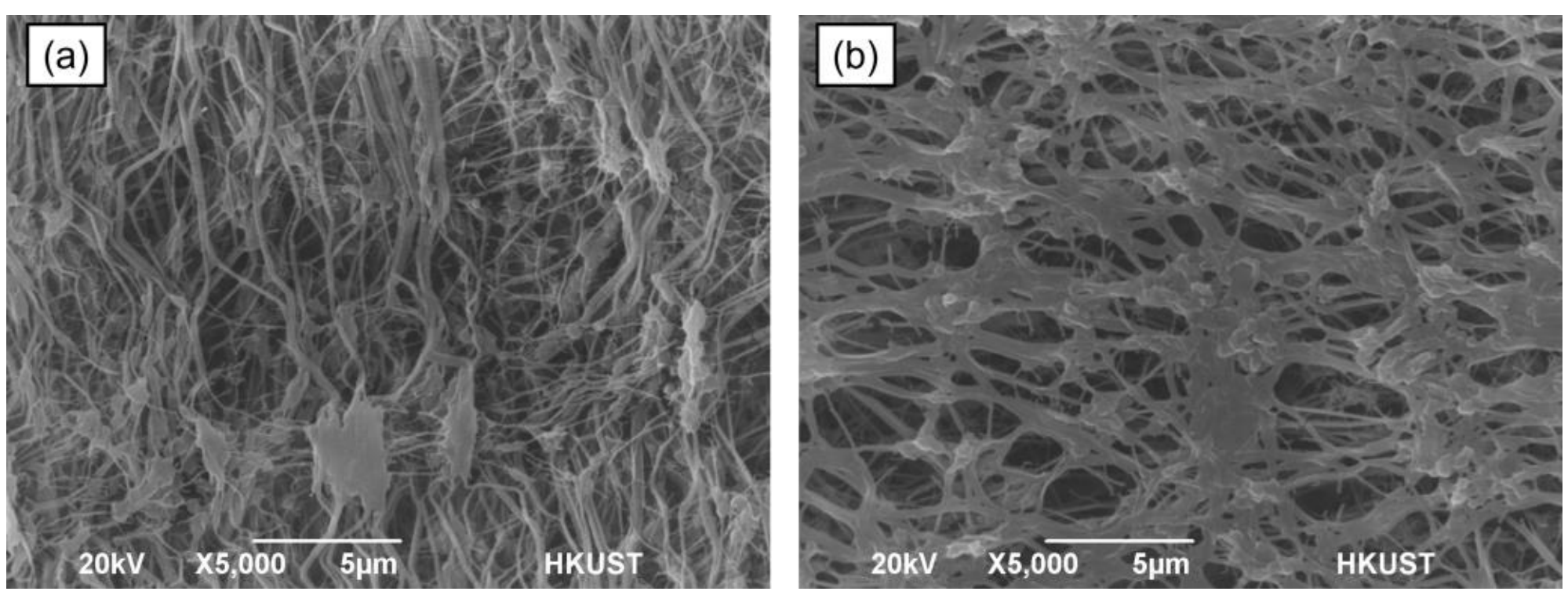

Supplementary Figure 4. SEM images of the PTFE membrane surface morphology. **a** pristine PTFE membranes; and **b** the membrane after the 500-h operation.

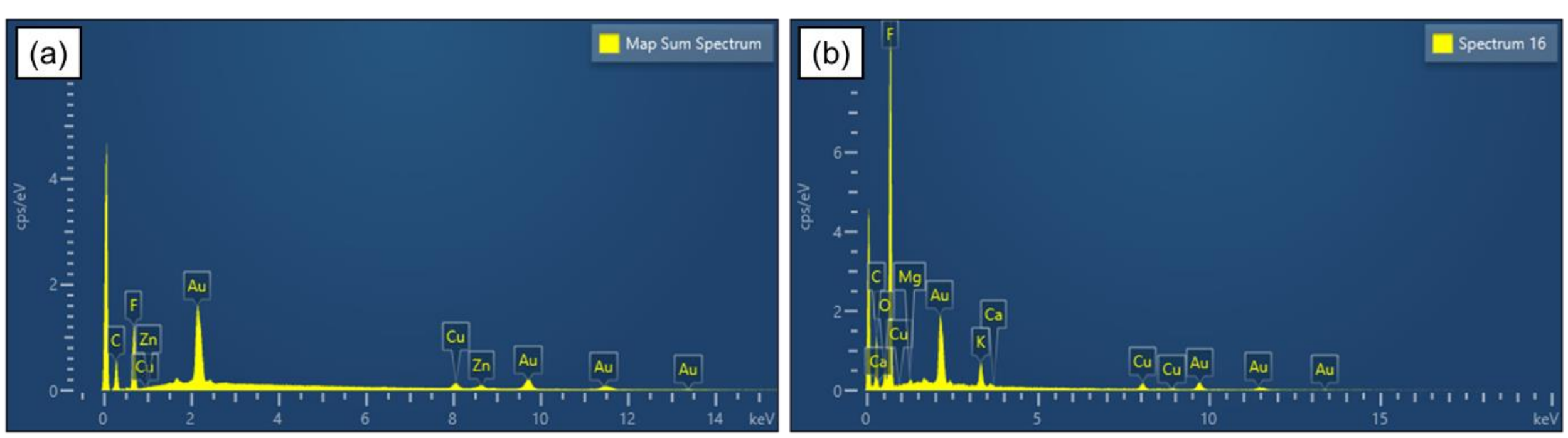

Supplementary Fig. 5 Energy-dispersive X-ray spectroscopy (EDX) spectrum of the PTFE membrane. EDX spectrum showing the surface elemental composition of **a** the pristine PTFE membrane; and **b** the PTFE membrane after 500 h of operation.

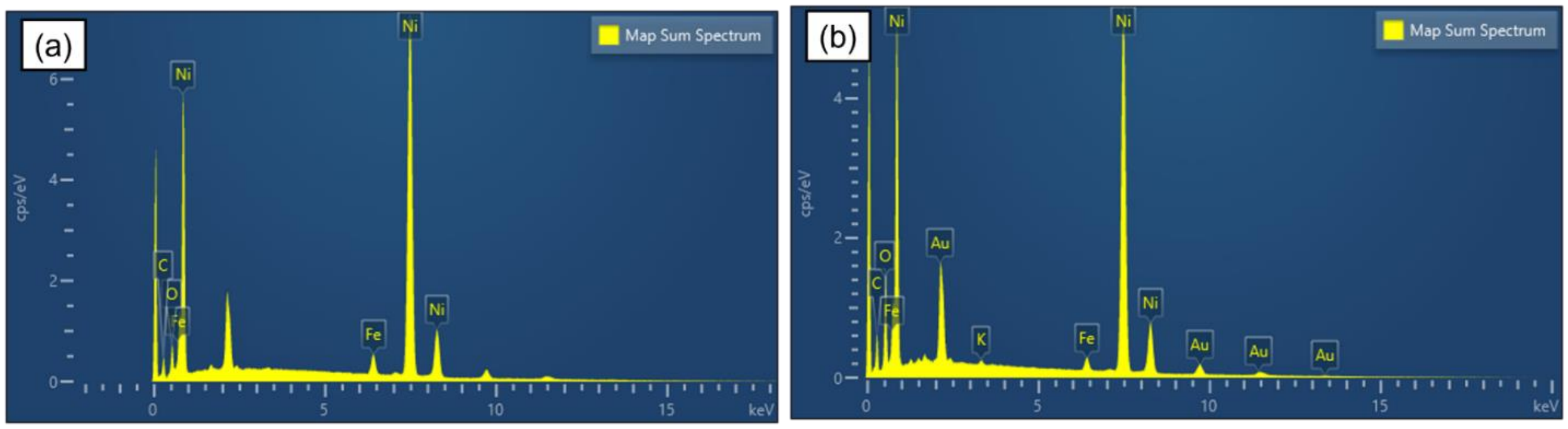

Supplementary Fig. 6 EDX spectrum of the electrodes after the 500-h operation showing no impurities. a Ni nanowire cathode and **b** NiFe anode.

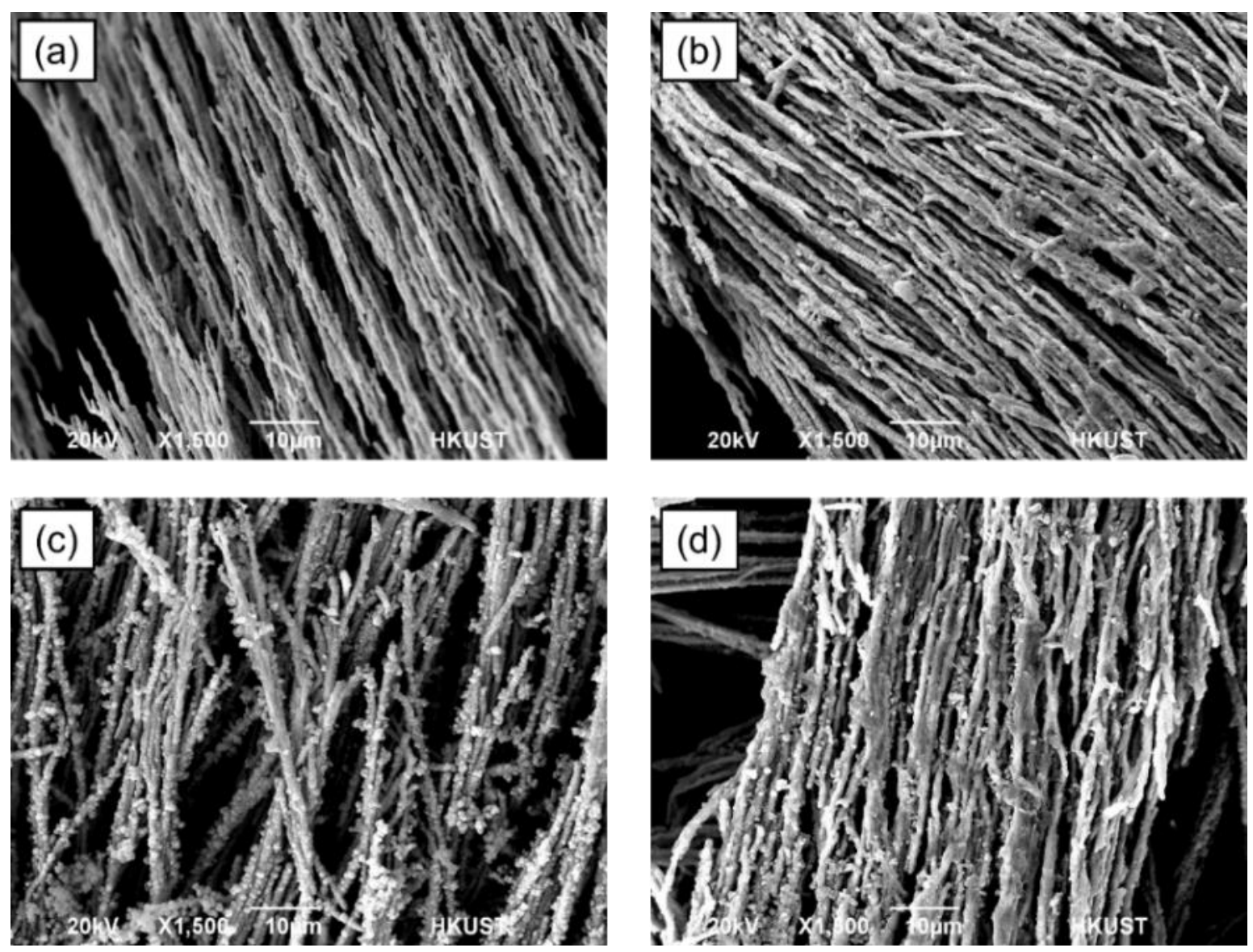

Supplementary Fig. 7 SEM images of the electrodes before and after the 500-h operation. **a** pristine Ni nanowire cathode; **b** Ni nanowire cathode after 500 h operation; **c** pristine NiFe anode; and **d** NiFe anode after 500 h operation.

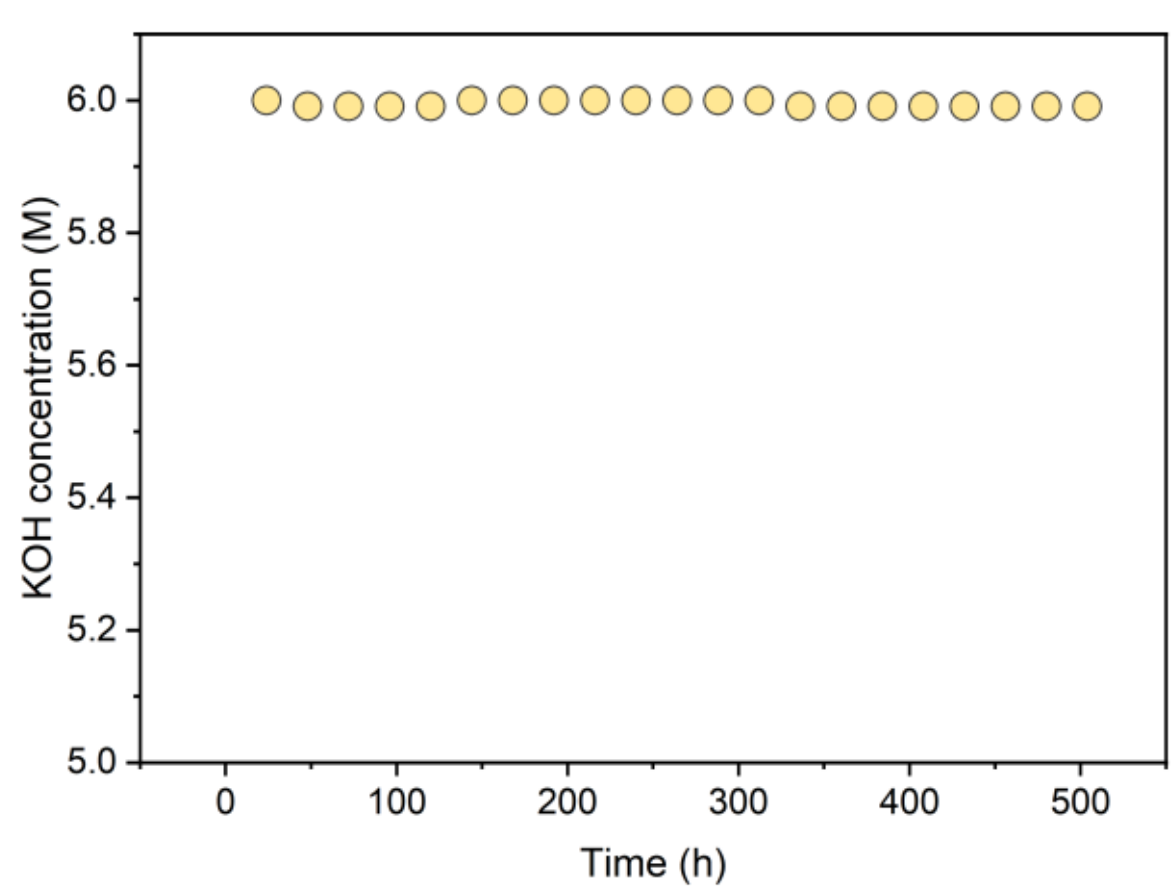

Supplementary Fig. 8 The change in the concentration of KOH osmotic agent during the 500-hour continuous operation of OMD-AWE.

Supplementary Table 5. EDX surface elemental composition analysis of the PTFE membrane before and after the 500-h continuous operation.

PTFE membrane	Element	Atom (%)
Pristine	C	46.30
	F	49.23
	Others	4.47
	F/C ratio	0.94
After 500 hours	C	49.74
	F	44.33
	Others	5.95
	F/C ratio	0.89

Supplementary Table 6. Surface elemental composition of electrodes before and after 500-h use.

Electrode	Elements	Pristine electrodes Atom (%)	Electrodes after 500 h Atom (%)
Anode	Ni	93.23	92.13
	Fe	6.77	7.86
Cathode	Ni	99.66	96.90
	Fe	0.34	3.10

Comment 4:

Quantify brine management and recovery — Report overall water recovery ratio, brine salinity (>70 g L^{-1}) disposal strategy, and associated energy/CO_2 equivalent burden.

Reply:

We thank the reviewer for raising the practical brine management issue, which is a major challenge for conventional desalination systems. A key advantage of our integrated system is its inherently simplified and environmentally benign approach to brine handling. Our system is intentionally operated at a low water recovery ratio (Fig. 4e). This design choice is not a limitation but a core feature that enables thermal self-circulation from AWE's waste heat (see details in our **Revised Manuscript Lines 340–462** **Thermal self-circulation improves OMD-AWE energy efficiency**). This mode of operation results in a brine with a minimal salinity increase of less than 5% compared to the feed seawater (Supplementary Fig. 14). This allows for direct ocean discharge, because the salinity falls within the range of natural coastal salinity variations, and far below the high-salinity

threshold of 70 g L^{-1} that is typical of high-recovery systems and poses significant disposal challenges. This approach is environmentally safe and eliminates the need for costly and energy-intensive brine treatment or dilution processes that are required for high-salinity brines. As a result, the associated energy and CO_2 equivalent burden for brine disposal is negligible. No additional pumping, mixing, or treatment processes are required. This simplified, zero-burden brine management, combined with the system's thermal self-sufficiency and ability to produce surplus water, makes it particularly well-suited for decentralized or offshore on-site operations.

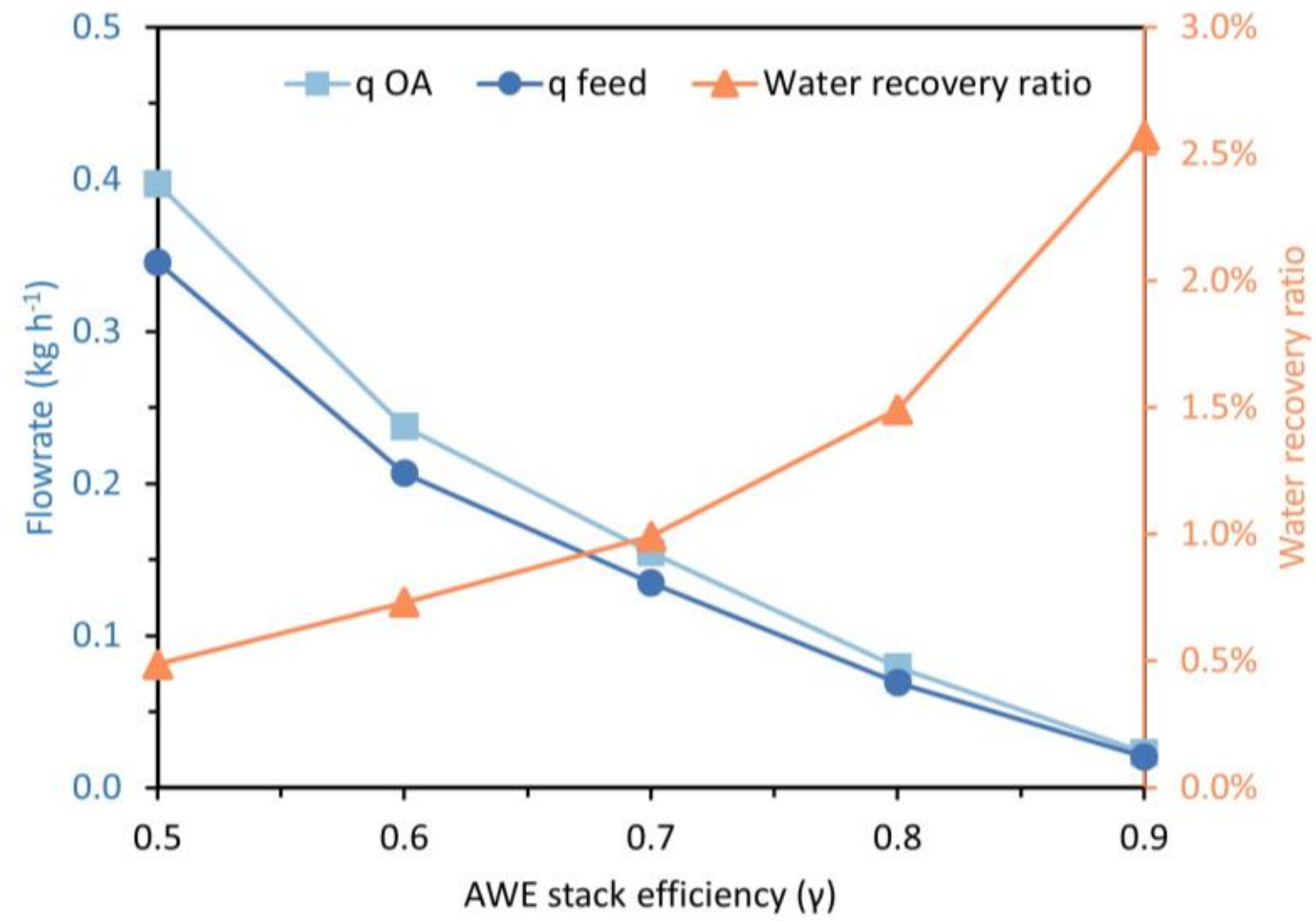

Fig. 4 e The flowrate of feed (q_{feed}) and osmotic agent (q_{OA}) with corresponding water recovery ratio as a function of AWE stack efficiency (γ).

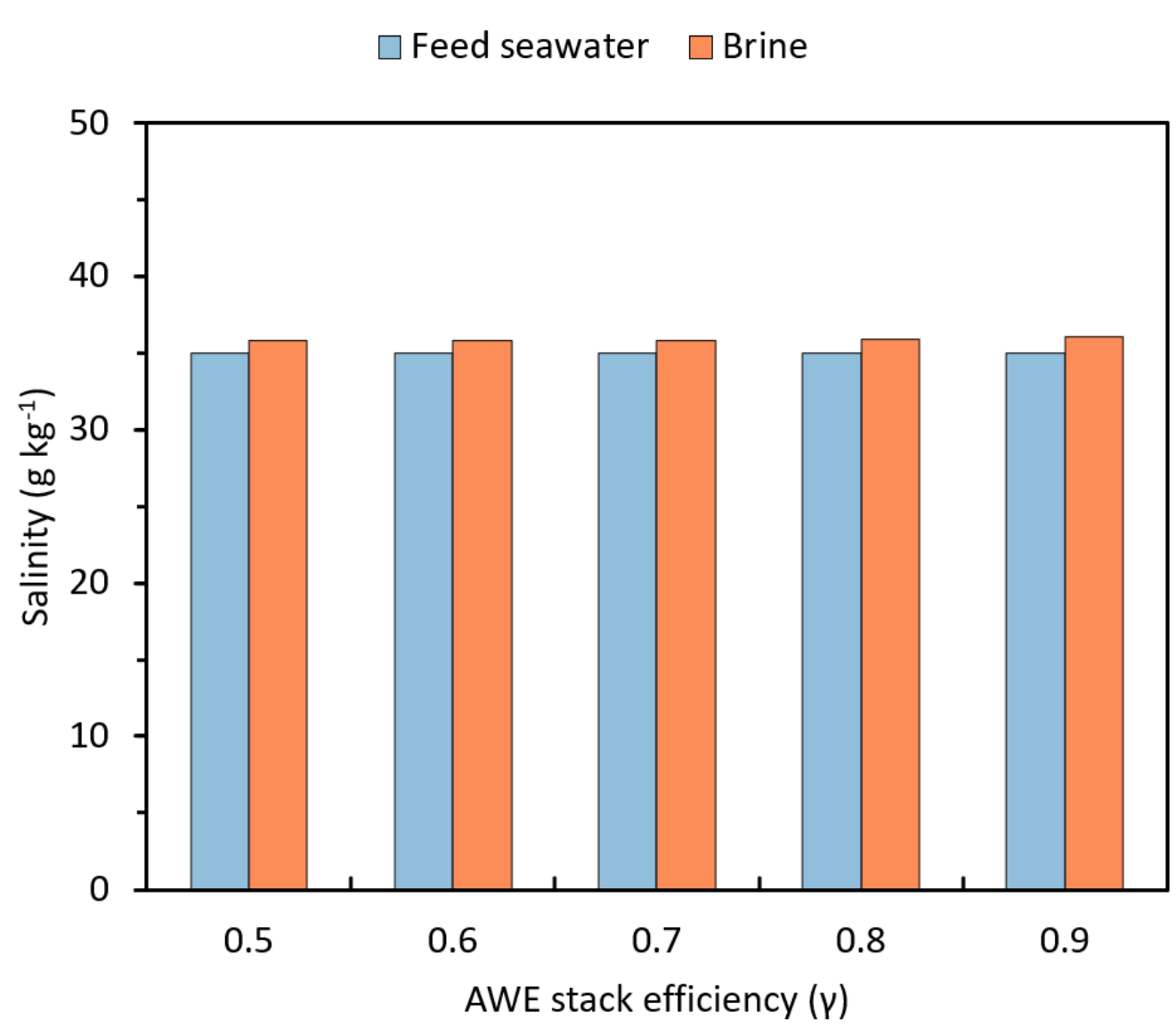

Supplementary Fig. 14 Salinity changes from feed seawater (standard 35 g kg^{-1}) to brine at designed water recovery rates.

These added in the **Revised Manuscript**:

Lines 457–460: "Another advantage of the low RR design is the simplification of brine management. It allows to produce brine with minimal salinity increase of <5% (Supplementary Fig. 14), falling within natural coastal salinity variations and enabling environmentally safe, direct ocean discharge without costly treatment processes."

Comment 5:

Membrane wetting resistance (Fig. 3b–d) — Supply the liquid entry pressure (LEP) of the PTFE membrane at 40 °C and discuss the impact of surfactants/organics present in natural seawater.

Reply:

We thank the reviewer for this critical suggestion. In direct response, we have now included the Liquid Entry Pressure (LEP) of the PTFE membrane across a temperature range of 20°C to 70°C and expanded our discussion on the impact of surfactants and organics present in natural seawater.

The membrane's resistance to wetting was quantified by measuring LEP, which is defined as minimum pressure required to force liquid through the hydrophobic membrane pores. The LEP was measured using a custom-built apparatus, as shown in Supplementary Fig. 15. The system consisted of a high-pressure N_2 cylinder equipped with a pressure regulator to slowly control gas feed, a sealed tube holding the PTFE membrane, and a pressure gauge to monitor the applied pressure. For each measurement, a dry membrane sample was mounted in the tube with its feed side filled with the test liquid while the permeate side opens to atmosphere. N_2 gas was gradually released to the tube, increasing the applied pressure. The permeate surface was continuously monitored, and the LEP was recorded as the pressure at the exact moment the first liquid droplet was observed.

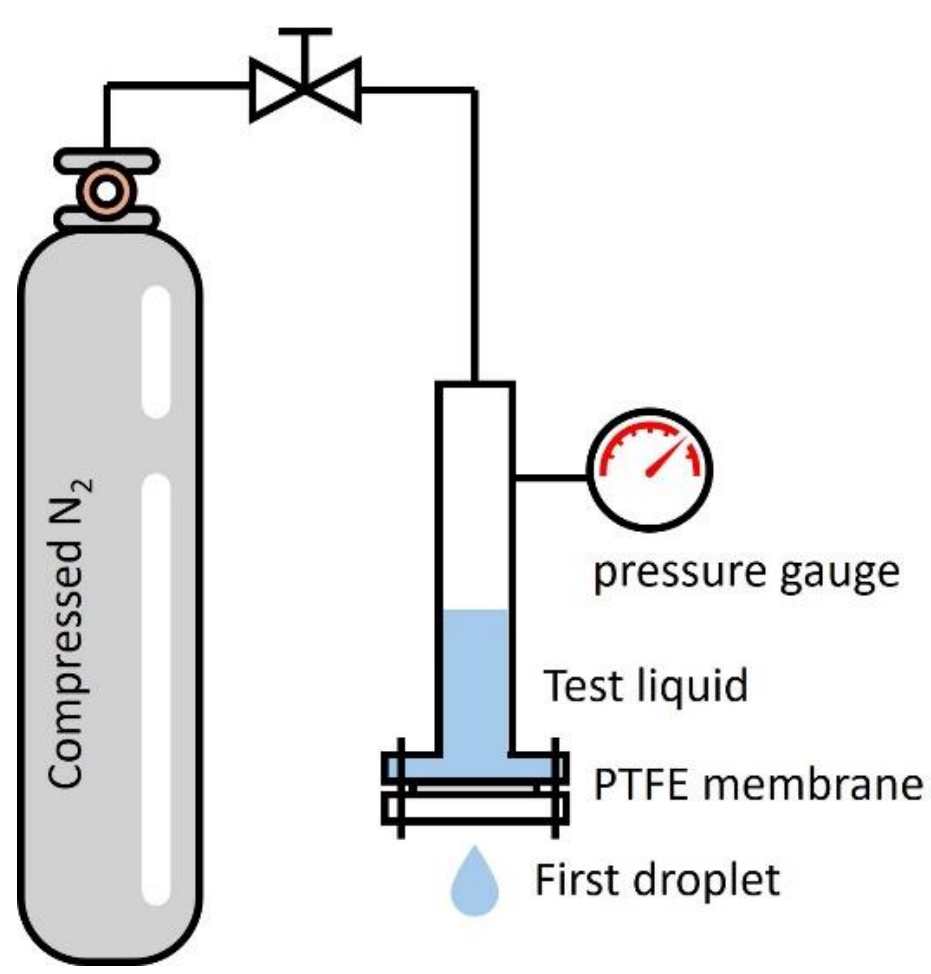

Supplementary Fig. 15 The setup for measuring liquid entry pressure (LEP) of membranes.

We evaluated the effect of temperatures on the LEP of PTFE membranes contacting with different liquids including pure water, seawater, and a seawater-equivalent 0.5 M NaCl solution (Fig. 2c). In general, the LEP for all these test liquids is highly sensitive to temperature, dropping over 75% with increasing liquid temperature from 20°C to 70°C, due to the decreased water surface tension and thermal softening of the PTFE polymer allowing the pores to stretch and enlarge under pressure[1]. A reduced LEP indicates increased susceptibility to wetting and reduced pressure tolerance, risking operational failure[2]. This forces the system to be operated at lower temperatures to maintain a safe wetting margin, which limits the water flux. However, the presence of dissolved salts in NaCl and seawater increases LEP by 17% over pure water at 20°C, while providing better temperature stability up to 40°C with values maintaining above 300 kPa. These salt ions, surrounded hydration shells, are repelled from the hydrophobic PTFE surface, creating a more ordered water layer at the interface which increases the contact angle and balances out the softening[3]. A tipping point was observed at 40°C where the LEP began to drop sharply for both saline liquids, since the thermal energy becomes sufficient to overcome the salt-induced interfacial stability, shifting the primary failure mechanism to polymer softening. However, seawater showed a more rapid LEP decline than NaCl at high temperatures, likely due to natural surfactants reaching their Kraft points (typically 15–50°C for most alkyl-chain ionic surfactants (C10–C16 chains)[4]) Above these temperatures, the surfactants become highly soluble and form micelles instead of crystals or gels, allowing them to adsorb to the liquid-vapour interface at the pore entrances where they lower the local surface tension and enable liquid permeation at a lower pressure.

These are added to the **Revised Manuscript Lines 164–187,** and **Methods Lines 573–583**. The setup for measuring LEP is provided in **Supplementary Fig. 15.**

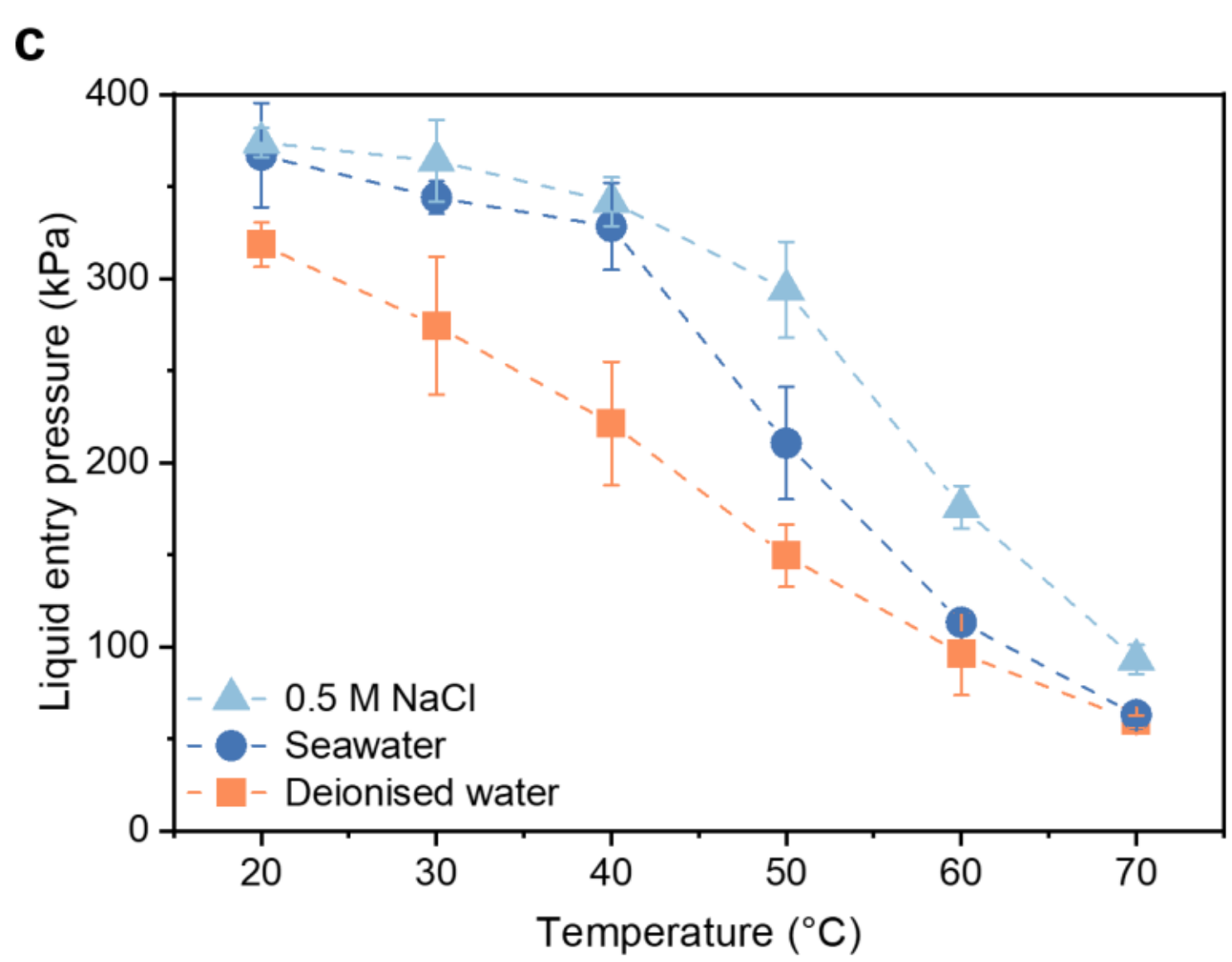

Fig. 2 c The effect of temperature on LEP tested with pure water, seawater, and 0.5 M NaCl.

Reference:

1. Saffarini, R. B., Mansoor, B., Thomas, R. & Arafat, H. A. Effect of temperature-dependent microstructure evolution on pore wetting in PTFE membranes under membrane distillation conditions. *J Memb Sci* **429**, 282–294 (2013).
2. Hou, Y., Shah, P., Constantoudis, V., Gogolides, E., Kappl, M. & Butt, H.-J. A super liquid-repellent hierarchical porous membrane for enhanced membrane distillation. *Nat Commun* **14**, 6886 (2023).
3. Kaya, O. Hydrophobic Membrane Wettability: Effects of Salinity and Temperature. *Membranes (Basel)* **15**, 58 (2025).
4. Chang, Q. Surface of Solution. in *Colloid and Interface Chemistry for Water Quality Control* 161–174 (Elsevier, 2016). doi:10.1016/B978-0-12-809315-3.00009-8.

Comment 6:
Thermodynamic reference data (Fig. 4b) — Add the water vapour pressure of 1 M KOH at 20 °C to aid comparison.

Reply:

We thank the reviewer for this comment. Following the optimization with 6 M KOH (detailed in Comment 10), we have updated Supplementary Fig. 2a to show vapor pressure curves as a function of temperature for three key streams: pure water, seawater-equivalent 0.5 M NaCl, and 6 M KOH for easy comparison of each case with varied temperatures. The Supplementary Fig. 2b includes a schematic illustrating the vapor pressure gradients between feed and permeate sides for three major systems in our manuscript: i) isothermal osmotic distillation (OD) with feed at RT (20°C) and 6 M KOH osmotic agent at RT, ii) osmotic membrane distillation (OMD) with feed at 40°C and 60°C and 6M KOH osmotic agent at RT, and iii) direct contact membrane distillation (DCMD) with feed at 40°C and 60°C and cooling water at 20°C at the permeate side.

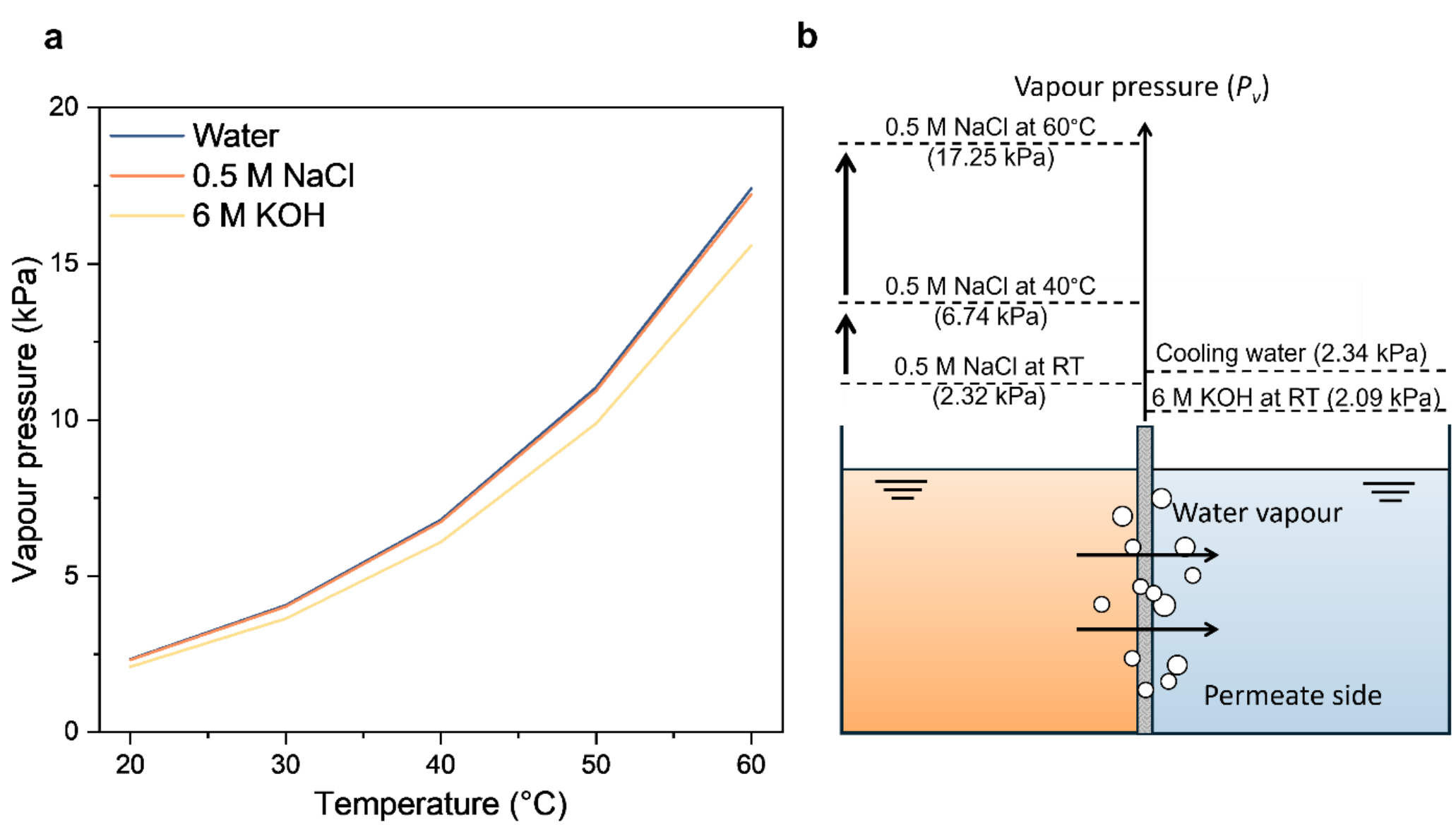

Supplementary Fig. 2 The comparison of vapour pressure (P_v) for **a** pure water, 0.5 M NaCl, and 6 M KOH at different temperatures; and **b** the schematic allowing us to calculate the vapour pressure gradient (ΔP_v) for i) isothermal osmotic distillation (OD) with feed at RT (20°C) and 6 M KOH osmotic agent at RT, ii) osmotic membrane distillation (OMD) with feed at 40°C and 60°C and 6M KOH osmotic agent at RT, and iii) direct contact membrane distillation (DCMD) with feed at 40°C and 60°C and cooling water at 20°C at the permeate side.

Comment 7:
Ensure consistent units and notation — Use SI units (kg m^{-2} h^{-1}) throughout; if "LHM" is retained, define it on first use.

Reply:

We have now defined all acronyms upon their first use in the manuscript. We have retained "LHM" as it is a standard unit of flux in membrane science, but it is now properly defined as "litre per hour per square meter (L h^{-1} m^{-2})" at its first occurrence.

Comment 8:
Disclose raw electrochemical data — Provide full E–I curves for the five cell AWE stack in the

Reply:

We appreciate the reviewer's suggestion to provide a more complete electrochemical dataset. Our AWE electrolyser is a 5-cell alkaline stack (LBE-5SC, Light Bridge Inc.). Each cell contains series-connected bipolar nickel alloy-based electrodes including a Ni nanowire-based cathode (4.4 cm diameter, JUNA TECH) and a NiFe-based anode (5.1 cm diameter, JUNA TECH). The two electrodes are separated by a 500 μm thick diaphragm (ZIRFON PERL UTP 500). Accordingly, the current-voltage (I-E) curves for the AWE stack at different operating temperatures have been provided in Supplementary Fig. 3. The description for our electrolyser is revised in the **Revised Manuscript Methods Lines 613–617.**

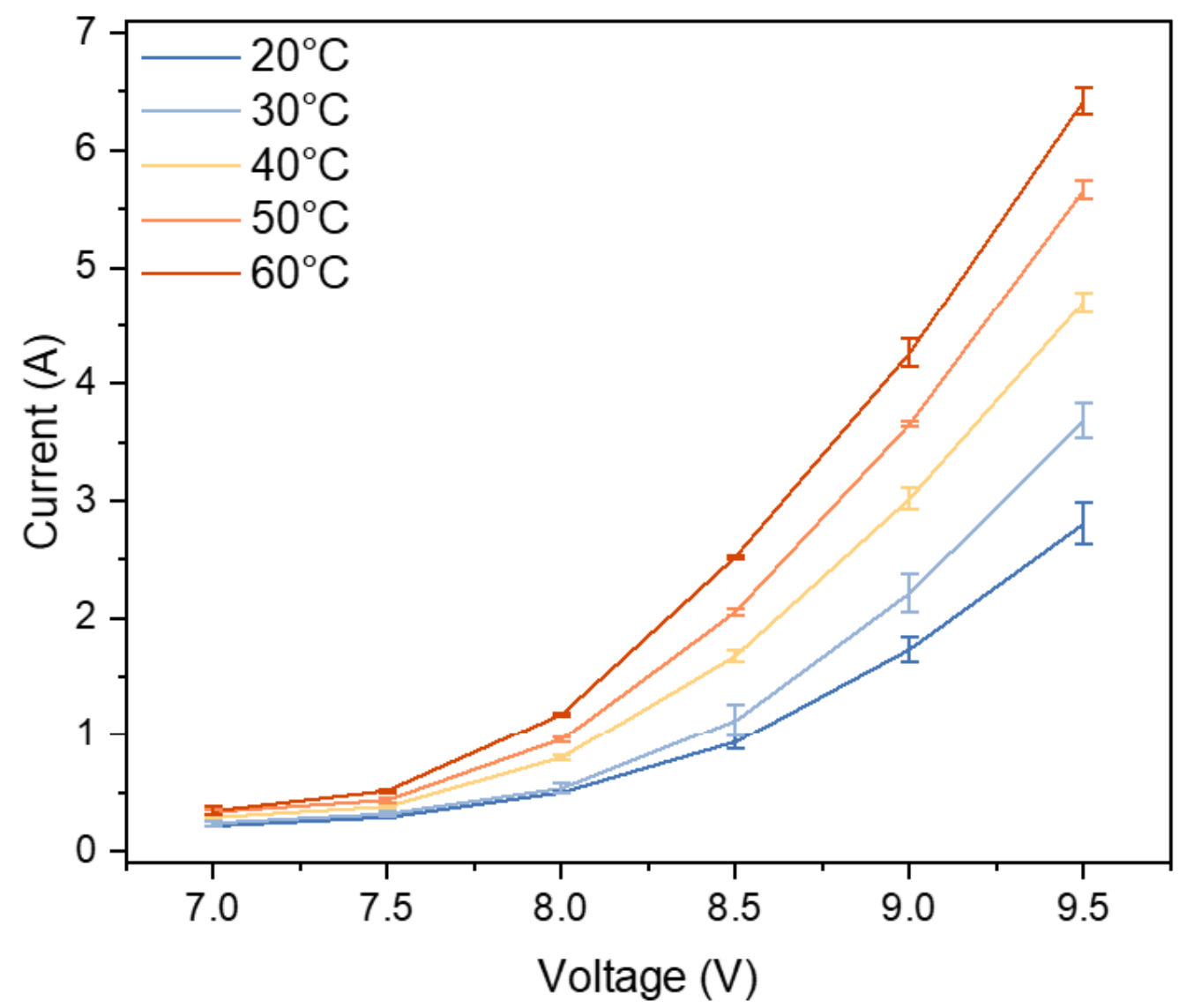

Supplementary Fig. 3 Temperature-dependent current-voltage (I-E) curves of the electrolyser with 6 M KOH electrolyte.

Comment 9:
Diagnose initial voltage rise (Fig. 4e) — Identify whether the early stage degradation originates in the OMD to AWE integration (e.g., temperature, pressure, electrolyte dilution) or within the AWE stack itself.

Reply:

We thank the reviewer for the concern about 4.5% voltage increase at startup. We attribute this initial voltage increase not to permanent electrochemical degradation, but a temporary system-level instability during the startup and equilibration phase. This increase was likely because OMD temporarily produce more water than the AWE consumes it during startup. This leads to a dilution of the KOH electrolyte, which in turn increases ohmic resistance and thus the operating voltage. Once the system reaches its stable thermal and ionic equilibrium, the water production and consumption balance, and the voltage stabilizes. This trend is different from typical degradation behaviour which shows continuous voltage increase over time. This effect was particularly pronounced in our initial experiments where we used a 1 M KOH. At this low concentration, the system's ohmic resistance is more sensitive to changes in dilution. In our revised manuscript, we have addressed this instability by operating with an optimal 6 M KOH which is not only more efficient but also less sensitive to the temporary system-level instability.

Our extended 500-h stability test (as demonstrate in **Comment 3**) provides the most compelling evidence that our AWE stack did not undergo degradation.

Comment 10:
Justify the choice of 1 M KOH — Because conventional AWE commonly operates at higher KOH concentrations for improved conductivity, explain the rationale for selecting 1 M despite the likely performance penalty.

Reply:

We thank the reviewer for this important point. The reviewer is correct that AWE performance is better at high KOH concentrations. Our initial use of 1 M KOH was to provide a direct benchmark against the $FOWS_{AWE}$ study. To address your valid concern and demonstrate our system's full potential, we have now repeated the key experiments with an optimal 6 M KOH electrolyte. The manuscript has been thoroughly updated with these new results. Accordingly, the discussion of TFC membranes has been moved to Supplementary Note 1 to improve the manuscript's clarity and focus.

Comment 11:
Foot print versus productivity — Provide a quantitative assessment of system footprint as a function of hourly H_2 output and water recovery ratio. OMD's inherent low recovery may demand large membrane areas; please evaluate this at practical scales.

Reply:

We thank the reviewer for this critical question regarding the OMD's lower water recovery and system's footprint and scalability. However, our work reveals that the OMD's low water recovery ratio is a strategic design choice that, counter-intuitively, enhances scalability and minimizes the system's physical footprint due to a higher water flux and thus smaller membrane areas.

This is because our water system operates under a fundamentally different paradigm under an AWE-integration scenario than conventional standalone membrane processes. In standalone membrane systems (e.g., RO, thermal MD), high water recovery is paramount because their primary costs and limiting resources are the pretreated feed water and the high-salinity brine disposal. For example, RO requires lots of pretreatment processes like sand filtration, ultrafiltration, chemical dosing, and temporary water storage systems, as well as requires high-pressure pumps which account for the main energy cost. Standalone MD requires excessive external heating for desirable 60–90°C feed temperatures. Therefore, a higher water recovery means getting more clean water out, and the cost for per unit of product water drops significantly. Their design is therefore optimized for maximum water resource efficiency. Our OMD-AWE system's limiting resource is not the seawater, but the finite waste heat from the AWE electrolyzer. Our design is therefore optimized for maximum system-level energy efficiency and stability. This paradigm shift is precisely why a low water recovery (<2.5%) becomes a powerful asset (suggested by our thermal

balance model detailed in the **Revised Manuscript Lines 340–462 Thermal self-circulation improves OMD-AWE energy efficiency**). A high seawater flowrate, dictated by the low recovery, acts as a powerful heat transfer fluid. It allows to capture more waste heat from the AWE through a heat exchange process, providing sufficient thermal energy to overcome the latent heat loss of vaporization. This enables to maintain the temperature gradient along the entire membrane for high and stable water production to meet the electrolyzer's water demand. Conversely, a low-flowrate (high-recovery) design faces a critical trade-off that makes it unsuitable for this integration. If this low flowrate were to absorb the full waste heat, its temperature would rise far above the optimal 40°C, creating unacceptable risks of fouling and wetting. If, on the other hand, it were only heated to the safe 40°C, it would carry an insufficient thermal load. This heat would be rapidly consumed by vaporization, causing a steep temperature drop along the membrane. The resulting collapse of the driving force would either require an impractically large membrane area or, more likely, lead to a complete system failure. In addition, another key advantage of the low-recovery operation is the simplification of brine management. The resulted brine salinity is only increased by <5%, falling within natural coastal salinity variations and enabling environmentally safe, direct ocean discharge without costly treatment processes.

To provide a direct quantitative assessment, we evaluated the system at a practical, industrial scale of a 1 MW AWE system. A 1 MW electrolyzer producing around 20 kg(H_2) h^{-1} which requires a supply of 200 kg h^{-1} of clean water (based on the common industry benchmark of 10 kg of water per kg of H_2 produced). Our OMD system's water flux is 20 kg m^{-2} h^{-1}, this demand can be met with only 10 m^2 of membrane area. Modern membrane modules (e.g., spiral-wound) have high packing densities. A module containing 10 m^2 of membrane has a very small physical footprint, conservatively estimated to be less than 1 m^2 of ground space. When compared to the ~15–20 m^2 footprint of the 1 MW AWE and its balance of plant[1], the OMD's contribution to the total system footprint is very small. Our LCA shows that membrane footprint contributes negligibly to overall environmental impact compared to AWE.

Therefore, the OMD's low recovery design is a key insight. We strategically trade a negligible amount of low-pressure pumping energy for massive gains in thermal efficiency, operation, stability, and simplified brine management. This confirms that our design is not constrained by membrane area and is fully scalable for practical, large-scale applications with minimal environmental impacts.

These insights are presented in our **Revised Manuscript**:

Lines 445–462: "Our strategic use of low RRs is the key that unlocks the thermal symbiosis, a design choice that is the direct opposite of the standalone desalination paradigm. In standalone systems, high water recovery is paramount as their primary costs and limiting resources are the pretreated feed water, extensive pumping and heating energy, and the high-salinity brine disposal. This forces them into a low-flowrate regime that is incapable of capturing large-scale, low-grade heat sources. Our design is optimised for maximum system-level energy efficiency and stability. This paradigm shift is precisely why a low RR becomes a powerful asset. By using abundant,

untreated seawater in a high-flowrate, low-recovery loop, we create a powerful heat transfer engine. This allows the feed seawater to capture and productively use the AWE's waste heat as primary driver for efficient and stable water production at a flux over 20 LHM. The high water flux minimises the required membrane area to just 10 m^2 per MW of AWE capacity, resulting in a compact physical footprint (<1 m^2) by using high packing-density membrane modules that are negligible compared to the electrolyser installation. Another advantage of the low RR design is the simplification of brine management. It allows to produce brine with minimal salinity increase of <5% (Supplementary Fig. 14), falling within natural coastal salinity variations and enabling environmentally safe, direct ocean discharge without costly treatment processes. Ultimately, this design trades a negligible increase in low-pressure pumping energy for substantial gains in energy efficiency, operational stability, and simplified brine management."

Reference:

1. Green hydrogen cost reduction. (2020). https://www.irena.org/publications/2020/Dec/Green-hydrogen-cost-reduction

Comment 12:
Interpretation of the Surplus Water Index (SWI) — While a high SWI signals good thermal integration potential, it does not guarantee that OMD water flux meets electrolyser demand. Clarify how SWI interacts with actual flux requirements and recovery ratios in sizing the membrane module.

Reply:

We thank the reviewer for this excellent question, which has prompted us to reconsider and clarify the role of the Surplus Water Index (SWI) in our work. We now realize that our previous manuscript overstated the importance of SWI, and we apologize if this created confusion.

SWI is not a core design principle for our thermal integration. The key to our thermal integration is the operational regime defined by a high flow rate and a low water recovery ratio, which allows the seawater to act as an efficient heat transfer engine to capture and utilize the AWE's waste heat (as we discussed in **Comments 1 and 11**). The SWI is simply an ancillary benefit that arises once this optimal operational window has been achieved. It quantifies the residual heat in the outlet stream of OMD, which could be used for secondary water production (Supplementary Fig. 10), but it does not participate in the primary OMD-AWE thermal design. To be more specific, the low recovery ratio is a core design input that enables the optimal thermal integration, the water flux is a key performance metric used for sizing the primary OMD module, while SWI is an ancillary benefit that quantifies the potential for additional water production via a secondary stage.

Furthermore, in re-evaluating the system considering the reviewer 3's comments, we identified an oversight in our initial thermal model (we had not fully accounted for heat loss across the OMD membrane). Our corrected model shows that the potential for surplus water is much more modest

than we previously estimated (SWI is now 1–4 depend on the stack efficiency, Supplementary Fig. 12). This makes the surplus water more suitable for internal uses, such as membrane washing, rather than a significant commercial byproduct.

To ensure the manuscript now accurately reflects this corrected understanding, we have removed the overly optimistic discussion and results for SWI from **former main text Lines 385–396**. We have replaced it with a brief, technical definition of SWI and its calculation **Lines 394–403** to provide context, with the modest results moved to **supplementary Figs. 10 and 12**. We have also revised title, abstract, conclusion, and main narrative to concentrate exclusively on the central concepts, i.e., thermally symbiotic design with the three counter-intuitive design principles.

Former text in Lines 385–396: "Another advantage of thermal self-circulation is its ability to produce surplus clean water for commercial or industrial applications when the OMD operates at higher water recovery ratios. To quantify this capability, we developed a Surplus Water Index (SWI) defined as the ratio between the maximum qfeed that can be heated by AWE waste heat and the minimum required for H2 production. Fig. 5e illustrates how SWI varies as functions of AWE's γ and OMD's water recovery ratio. Specifically, when SWI is greater than 1, the system enables the generation of more clean water than needed for H_2 production without additional energy input. For example, with AWE at 60% efficiency (γ = 0.6) and OMD at 20% water recovery, the system can process 15.2 times more feed water than minimally required. Even highly efficient AWE systems (γ = 0.9) operating at 15% recovery can produce twice the water needed for H2 production. This surplus capacity creates valuable secondary water output for commercial or industrial applications while maintaining thermal self-sufficiency (Supplementary Fig. 9)." was revised as:

Lines 394–403: "As the warm stream cools to RT, the released heat flow via DCMD (Φ_{DCMD}) drives surplus water vapour transport and thermal conduction as expressed by Eq. 12. Based on this available heat, we can calculate the required DCMD membrane area (S_{DCMD}) with the corresponding surplus water flux ($J_{w,DCMD}$). The system's ability to co-produce clean water is quantified by a Surplus Water Index (SWI), establishing a baseline of 1 for the water used in electrolysis and adding to it the ratio of the surplus DCMD water to the primary OMD water (Eq. 13). An SWI above 1 indicates a net surplus of clean water, demonstrating simultaneous H_2 and clean water production.

$$\Phi_{DCMD} = c_{seawater} q_{feed} \left(T_{OMD,feed,out} - RT\right) = \left[c_m \theta \Delta P_v \Delta H_v + U \frac{\left(T_{OMD,feed,out} - 20°C\right)}{2}\right] \cdot S_{DCMD} \quad (12)$$

$$Surplus\ Water\ Index\ (SWI) = 1 + \frac{J_{w,DCMD} S_{DCMD}}{J_{w,OMD} S_{OMD}} \quad (13)$$

Lines 543–545: "In addition, the thermally self-sustaining design, combined with its ability to produce surplus water and simplified brine management, makes the system well-suited for decentralised and offshore on-site operations."

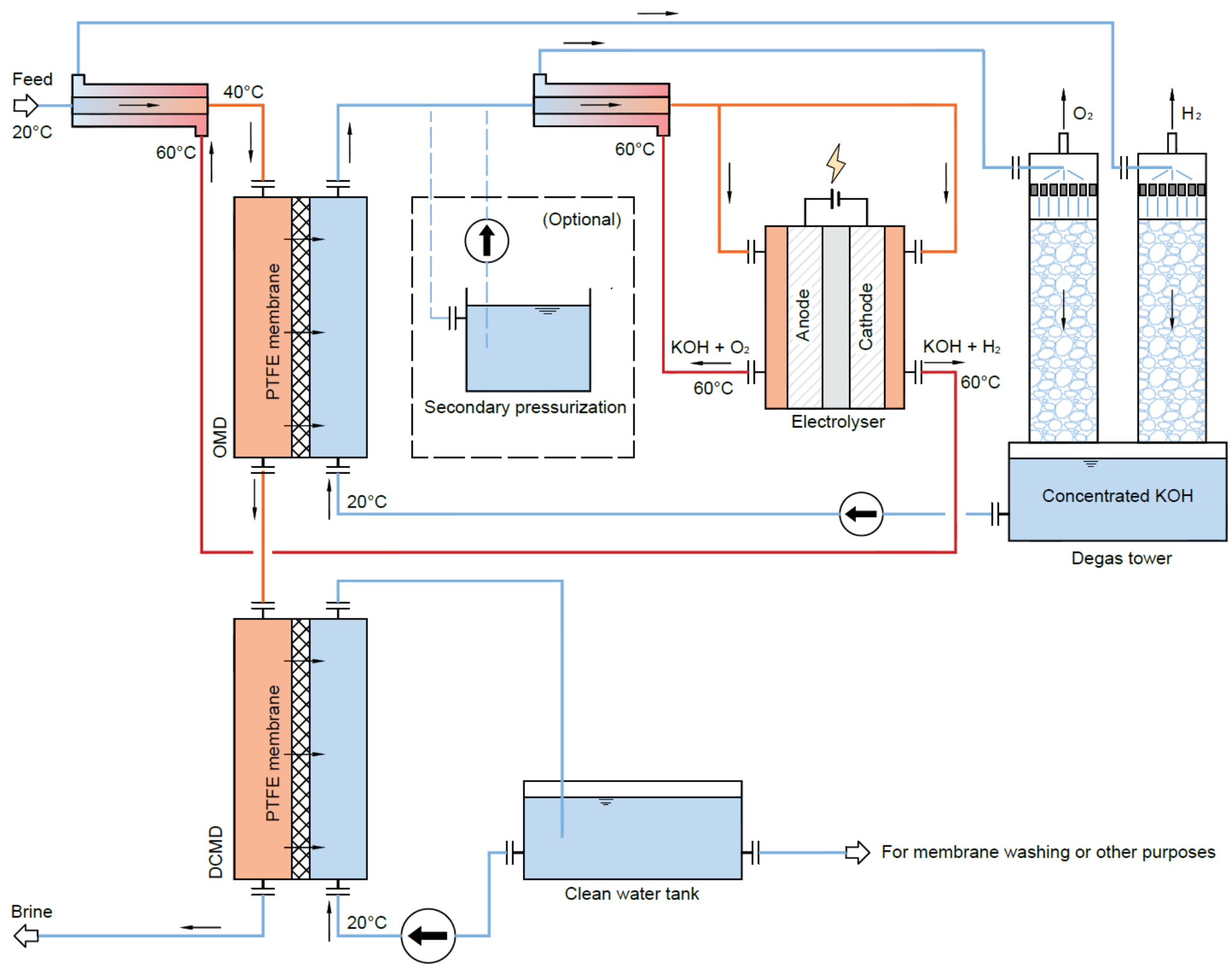

Supplementary Fig. 10 Process design showing secondary DCMD process utilizing residual heat from OMD feed outlet for additional water production

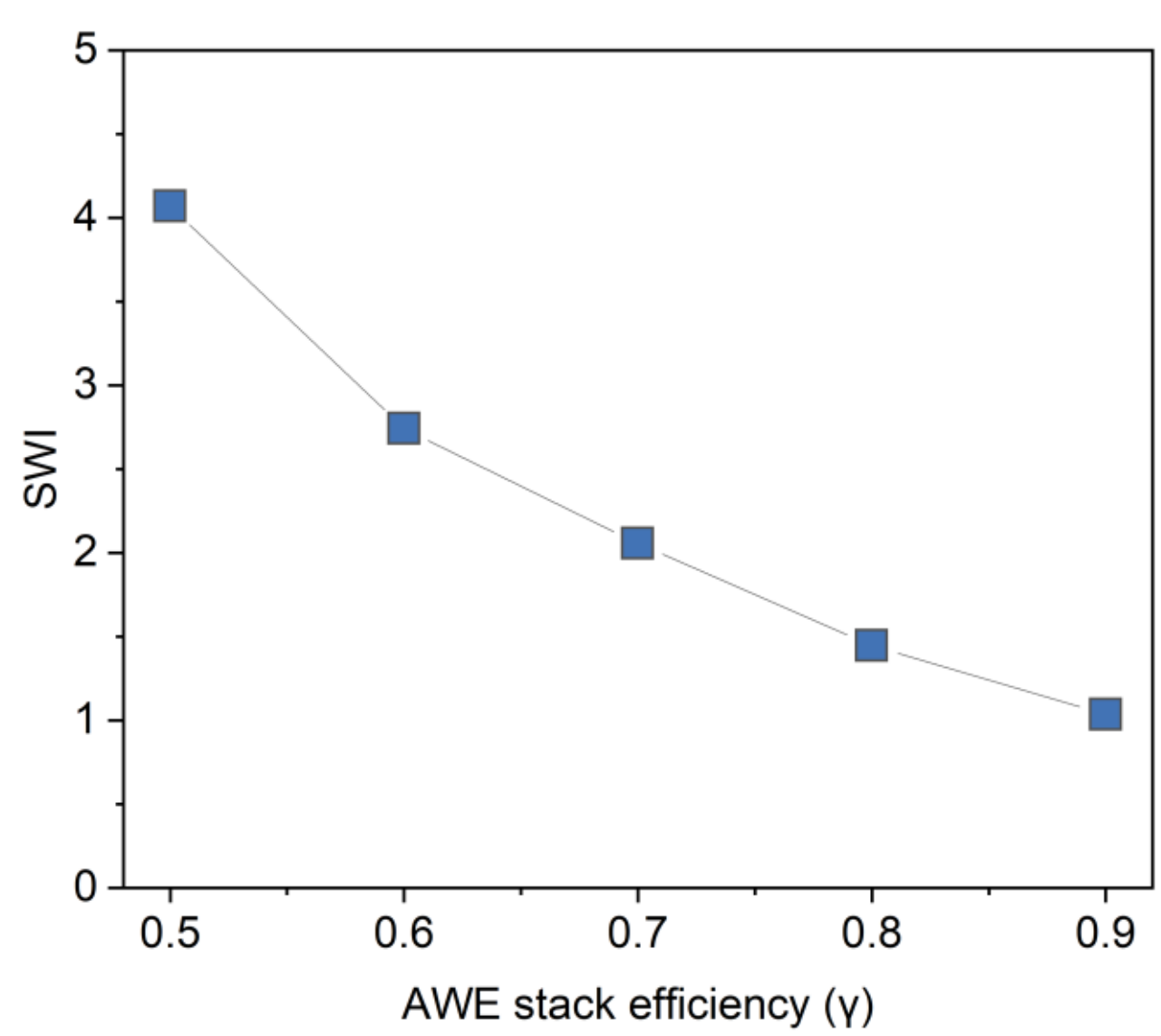

Supplementary Fig. 12 Surplus Water Index (SWI) as a function AWE stack efficiency (γ).

Comment 13:

Define all acronyms at first mention (e.g., “LHM”, “SEC”).

Reply:

We have now checked and defined all acronyms upon their first use in the manuscript.

Former Title: Thermally symbiotic osmotic membrane distillation-electrolysis unlocks efficient and stable hydrogen production directly from seawater

Authors: Gabriela Scheibel Cassol, Chii Shang, Paul Westerhoff, Yinghao Song

Manuscript ID: NCOMMS-25-36631B

Responses to comments of Reviewer 1:

Thank you very much for your detailed responses, which have addressed most of my concerns and clarified my questions. I appreciate the careful effort and time you have invested in providing such thorough explanations. The previously misleading phrasing has been revised, and the current description of the innovation is more accurate and appropriately reflects the novelty of the work. However, I still have a few remaining questions, for which I would sincerely appreciate further clarification.

Comment 1:

Since I am not a specialist in water treatment, my understanding is based on the literature I have reviewed. In most publications, the flux is typically reported in units of LMH. Could you please confirm whether the notation LHM is appropriate in your context?

Reply:

We appreciate the careful observation. Although LHM and LMH are dimensionally equivalent, we used LHM in our previous publication [1] and carried it over to this manuscript. We acknowledge that LMH is the more widely accepted standard and we have now updated all units to LMH for consistency with standard practice.

Reference:

1. Scheibel Cassol, G., Shang, C., An, A. K., Khanzada, N. K., Ciucci, F., Manzotti, A., Westerhoff, P., Song, Y. & Ling, L. Ultra-fast green hydrogen production from municipal wastewater by an integrated forward osmosis-alkaline water electrolysis system. Nat Commun 15, 2617 (2024).

Comment 2:

Thank you very much for providing the detailed experimental procedures. Given that the active area of your OMD module is 1 cm^2 and the reported flux is 20 LMH, the corresponding permeate production rate is approximately 0.033 mL min^{-1}. Considering that your AWE system consists of a five-cell stack, I would like to inquire about the approximate water feed rate for AWE. Specifically, is the permeate production of the OMD module sufficient to sustain the water demand of the stack?

Reply:

We appreciate your feedback. Yes, the production rate of membrane was sufficient to sustain the AWE stack, because the system was operated based on the principle of water balance.

The water production rate is determined by water flux (~22 LMH for 0.5 M NaCl as feed, or at ~20 LMH for seawater) multiplying by the membrane area (*S*), whereas the water consumption

depends on the AWE current (i). Matching the water production and demand is achieved by coordinating the two variables of S and i. Due to the fixed geometry of our custom-made membrane cell ($S = 1$ cm^2), the AWE current was utilised as the easier adjustable parameter to establish water balance in our current setup. Based on the membrane area-specific current (J_m) of 1.34 A cm^{-2}, an AWE current of 1.34 A was required to consume the produced water at a rate of 0.037 mL min^{-1} with 1 cm^2 membrane at water flux of 22 LMH. However, as you observed, our 5-cell AWE stack has not reached its maximum capacity (i.e., the applied current density is 0.089 A cm^{-2} which is much lower than the 0.5 A cm^{-2} for a standard value). Therefore, if fully reaching its capacity, it would require a current of 7.6 A (15.2 cm^2 of electrode area × 0.5 A cm^{-2}). To sustain this higher demand, the membrane area would need to be scaled up to 5.67 cm^2 (7.6 A/1.34 A cm^{-2}).

While the 1-cm^2 membrane module limited the total current in our specific experiment, the results demonstrate the validity of water balance principle, which supports experimental reproducibility with different setups and scalability for larger system matching. For real-world engineering, the design procedure typically begins by defining the target electrolyser power, followed by sizing the membrane unit (e.g., calculating the required number of spiral-wound modules) to match the specific water production and consumption requirements.

These clarifications are added to **the newly revised Manuscript**:

The former texts in Line 273–276: "Due to the fixed geometry of our custom-made membrane cell (S = 1 cm2), the AWE current was used as the adjustable parameter to ensure the water balance and determined to be 1.34 A. This approach is also adaptable for the other scenario where AWE optimal current is fixed to determine the needed membrane area." has been revise as:

Line 286–293: "Due to the fixed geometry of our custom-made membrane cell ($S = 1$ cm^2) which produced water at a rate of 0.037 mL min^{-1}. To strictly maintain the system's water balance, the AWE current was used as the adjustable parameter and set to 1.34 A to exactly consume this produced volume. Consequently, this 5-cell AWE stack operated at a partial current density of 0.089 A cm^{-2}. If operating this stack at an industrial current density of 0.5 A cm^{-2} (which requires a total current of 7.6 A), the membrane would scale to 5.67 cm^2. This validates the water balance principle, demonstrating that in real-world engineering, the membrane area can be flexibly sized to match any target electrolyser capacity."

Comment 3:

In addition, the IV curve is commonly referred to as the polarization curve. Thank you very much for providing the additional electrochemical characterization data. These measurements are highly valuable for assessing and interpreting the performance of the cell.

Reply:

Thank you for the clarification regarding terminology and the positive feedback. As suggested, we have replaced “IV curve” with “polarization curve” throughout the text.

Comment 4:

Could you please elaborate further on the thermal management strategy implemented in your system? I am particularly interested in whether the outlet water temperature of the AWE (reported as 60 °C) can be maintained at approximately the same temperature upon reaching the inlet of the heat exchanger. In typical laboratory setups, even when the reservoir is heated to 100 °C, the water temperature at the cell inlet often decreases to around 60 °C even with thermal insulation applied along the flow path, due to unavoidable heat losses.

Reply:

Thanks for your insightful comment regarding thermal management. We agree that in laboratory-scale setups, heat loss is a critical factor due to the high surface-area-to-volume ratio of pipes and low flow rates. We performed a heat transfer model to address this concern. Our analysis confirms that heat loss is significant at the laboratory scale, but it becomes negligible at the industrial scale (comparison shown in Supplementary Table 3).

Supplementary Table 3 The comparison in thermal loss between experimental setup and industrial application

Feature	Experimental setup (8 W AWE)	Industrial application (1 MW AWE)
Flowrate	0.184 kg h^{-1}	21,395 kg h^{-1}
Pipes with thermal insulation	25# PharMed BPT Tubing wrapped with 5 mm thick Aluminum-clad Expanded Polyethylene (EPE) foam, L = 0.5 m, k_{ins} = 0.038 W m^{-1} K^{-1}	DN65 PVC pipe wrapped with 20 mm thick Aluminum-clad polyurethane (PU) foam, L = 20 m, k_{ins} = 0.024 W m^{-1} K^{-1}
Thermal resistance	11 K W^{-1}	0.19 K W^{-1}
Temperature drop	15°C (drop from 50 to 35°C)<br>40°C (drop from 100 to 60°C)	0.007°C<br>(0.2°C if no thermal insulation)

For our experimental setup, considering the AWE electrolyser works at a current density of 0.5 A cm^{-2} and thus cell voltage of 2.02 V (based on our polarisation curves) with outlet temperature ($T_{AWE,out}$) at 50°C (recommended by our newly revised manuscript). To maintain thermal self-circulation, the required flowrate for the KOH osmotic agent (OA) is 0.184 kg h^{-1}. We modelled the heat loss for a 0.5 m long transfer line for delivering the KOH using PharMed BPT Tubing (25#, inner diameter (d_{pipe}) = 4.8 mm and outer diameter (d_{pipe}’) = 8 mm). We consider thermal

insulation by wrapping the pipe with flexible Aluminum-clad Expanded Polyethylene (EPE) foam at a thickness of 5 mm, showing a thermal conductivity (k_{ins}) of 0.038 W m^{-1} K^{-1}. Thus, the temperature drop (ΔT_{drop}) as the KOH transfers from the outlet of electrolyser to the inlet of heat exchangers could be determined by using Eqs. R1–2.

$$\Delta T_{drop} = \frac{T_{AWE,out} - T_{amb}}{R_{th} \cdot c_{KOH} \cdot q_{OA}} \quad (R1)$$

$$R_{th} = \frac{\ln\left(\frac{d_{ins}}{d_{pipe}}\right)}{2\pi \cdot k_{ins} \cdot L} \quad (R2)$$

where c_{KOH} is the specific heat capacity of KOH at 3500 J kg^{-1} K^{-1}, q_{OA} is its flowrate at 0.184 kg h^{-1}, T_{amb} is ambient temperature at 20°C, and R_{th} is the thermal resistance which is a function of the outer diameter of the insulation layer (d_{ins} =2×5 + 8 = 18 mm), the pipe's inner diameter (d_{pipe}), k_{ins} and transferring distance (L, at ~0.5 m). Therefore, the calculated R_{th} is around 11 K W^{-1}, and thus ΔT_{drop} is 15°C (i.e., dropping from 50°C to 35°C) even with insulation applied. If $T_{AWE,out}$ is 100°C, the drop becomes 40°C consistent with your estimation. We acknowledge that achieving thermal self-circulation at this laboratory-scale is thermodynamically impossible, and we faced the same limitation during our experiments, where the heat loss was too great to sustain the thermal loop. This is also the reason why we utilised thermal modeling in the manuscript to validate our system's feasibility, rather than relying on empirical temperature measurements for this specific section.

However, the thermal insulation would work perfectly for an industry large scale. Considering scaling our 8.6 W lab unit (2.02 V and 4.25 A) up to a 1 MW industrial plant. The flowrate of KOH becomes 116,279 times (q_{OA} = 21,3950 kg h^{-1}). If maintaining a standard engineering practice for flow velocity at ~1.5 m s^{-1}, we can select a DN65 pipe (the inner and outer diameter is 62.7 and 73 mm) wrapped with Aluminum-clad polyurethane (PU) foam (k_{ins} = 0.024 W m^{-1} K^{-1}, which is less flexible but offers better thermal resistance than the EPE foam) at a thickness of 20 mm,. The pipe is assumed to be 20 m long. By applying the model with these industrial parameters, the temperature of KOH in this scale only drops by 0.007°C, which is negligible. Even without thermal insulation by using only PVC pipes (k_{ins} = 0.2 W m^{-1} K^{-1}), the temperature drop is only 0.2°C. Our comparative analysis reveals that the observed temperature drop is specific to the laboratory setup. For real-world applications, standard insulation methods like Aluminum-clad PU foam are sufficient to maintain the thermal profile.

These are added to **newly revised manuscript:**

Lines 417–418: "Both pipes and heat exchangers are wrapped with aluminum-clad polyurethane foam insulation, resulting in negligible heat losses" and Supplementary Note 4.

Comment 5:

Since the focus of the manuscript has been shifted to a thermally integrated system, I would like to suggest including more discussion on the utilization of thermal energy. Providing additional details or analysis on how heat is recovered, distributed, and applied within the system would strengthen the manuscript and better highlight its relevance and impact. Specifically, I would appreciate more information regarding the design of the heat exchanger. The reported heat exchange efficiency appears to be exceptionally high, and I am curious about the specific design features that enable such effective heat transfer.

Reply:

We really appreciate your suggestion. Adding the heat exchanger design significantly strengthens our manuscript, which improves model accuracy and even allows us to predict system performance under dynamic operating conditions once heat exchangers are determined (see newly added section "Dynamic self-regulation under intermittent renewable energy input" if you have interests).

To address this, we must first clarify a common terminological distinction, where "efficiency" and "effectiveness" are often confused but fundamentally different. The heat exchanger effectiveness (ε) is actually the design parameter we should use. It is the ratio of the actual heat transfer (Φ_{actual}) to the maximum thermodynamically possible heat transfer (Φ_{max}) which would occur if the heat exchanger had infinite surface area (as shown in Eq. R3) [1].

$$\varepsilon = \frac{\Phi_{actual}}{\Phi_{max}} = \frac{C_h(T_{h,in} - T_{h,out})}{C_{min}(T_{h,in} - T_{c,in})} = \frac{C_c(T_{c,out} - T_{c,in})}{C_{min}(T_{h,in} - T_{c,in})} \quad (R3)$$

Where subscripts h and c represent hot and cold fluids, respectively, T is temperature, C represents the heat capacity rate (W K^{-1}) obtained from the product of flowrate (q) and specific heat capacity (c), C_{min} denotes the smaller of the two heat capacity rates (i.e., C_{min} = min[C_h,C_c]). The value of ε depends on the heat exchanger's flow arrangement, heat transfer area, and turbulence, with values typically ranging from 0.60 (shell-and-tube) to 0.95 (plate). On the other hand, thermal efficiency (η) refers to the ratio of heat output to heat input, which represents how well insulated the unit is from the environment. For industrial application, this is not a primary design parameter because it is typically near 100% with standard insulation practices, similar to the pipe case in Comment 4.

Therefore, we revised the two equations as shown in Eqs. R4 and 5 (corresponding to Eqs. 6 and 7 in Manuscript) for simulating the thermal balance of the heat exchanger 1 (Hx,1) and 2 (Hx,2), respectively.

$$\Phi_{Hx,1} = \varepsilon \cdot c_{KOH}\frac{q_{OA}}{2}(T_{AWE,out} - T_{feed}) = c_{seawater}q_{feed}(T_{OMD,feed,in} - T_{feed}) = c_{KOH}\frac{q_{OA}}{2}(T_{AWE,out} - T_{cool,1}) \quad (R4)$$

$$\Phi_{Hx,2} = \varepsilon \cdot c_{KOH}\frac{q_{OA}}{2}(T_{AWE,out} - T_{OMD,OA,out}) = c_{KOH}q_{OA}(T_{AWE,in} - T_{OMD,OA,out}) = c_{KOH}\frac{q_{OA}}{2}(T_{AWE,out} - T_{cool,2}) \quad (R5)$$

Where q_{feed} and q_{OA} are the flowrate of seawater feed and KOH osmotic agent (OA), respectively; $c_{seawater}$ and c_{KOH} are the specific heat capacity of seawater and KOH, respectively; the temperature of each hot or cool streams refers to Fig. 4b. To characterise the heat exchanger design, we used the Number of Transfer Units (*NTU*) method. It represents the dimensionless thermal size of the exchanger and is calculated as a function of ε and heat capacity rate ratio (C_r) which is the ratio of the minimum to maximum heat capacity rates (Eq. R6). By applying a standard overall heat transfer coefficient (U) of 2,000 W m^{-2} K^{-1} typical for liquid-to-liquid system, we determined normalised heat transfer area (R_{hx}) which is the ratio of heat exchanger area (S_{hx}) required to achieve the target *NTU* to the membrane area (S) as shown in Eq. R7.

$$NTU = \frac{1}{C_r - 1}\ln\left(\frac{\varepsilon - 1}{\varepsilon C_r - 1}\right) = \frac{1}{\frac{C_{min}}{C_{max}} - 1}\ln\left(\frac{\varepsilon - 1}{\varepsilon\frac{C_{min}}{C_{max}} - 1}\right) \quad (R6)$$

$$R_{hx} = \frac{S_{hx}}{S} = \frac{NTU \cdot C_{min}}{U \cdot S} \quad (R7)$$

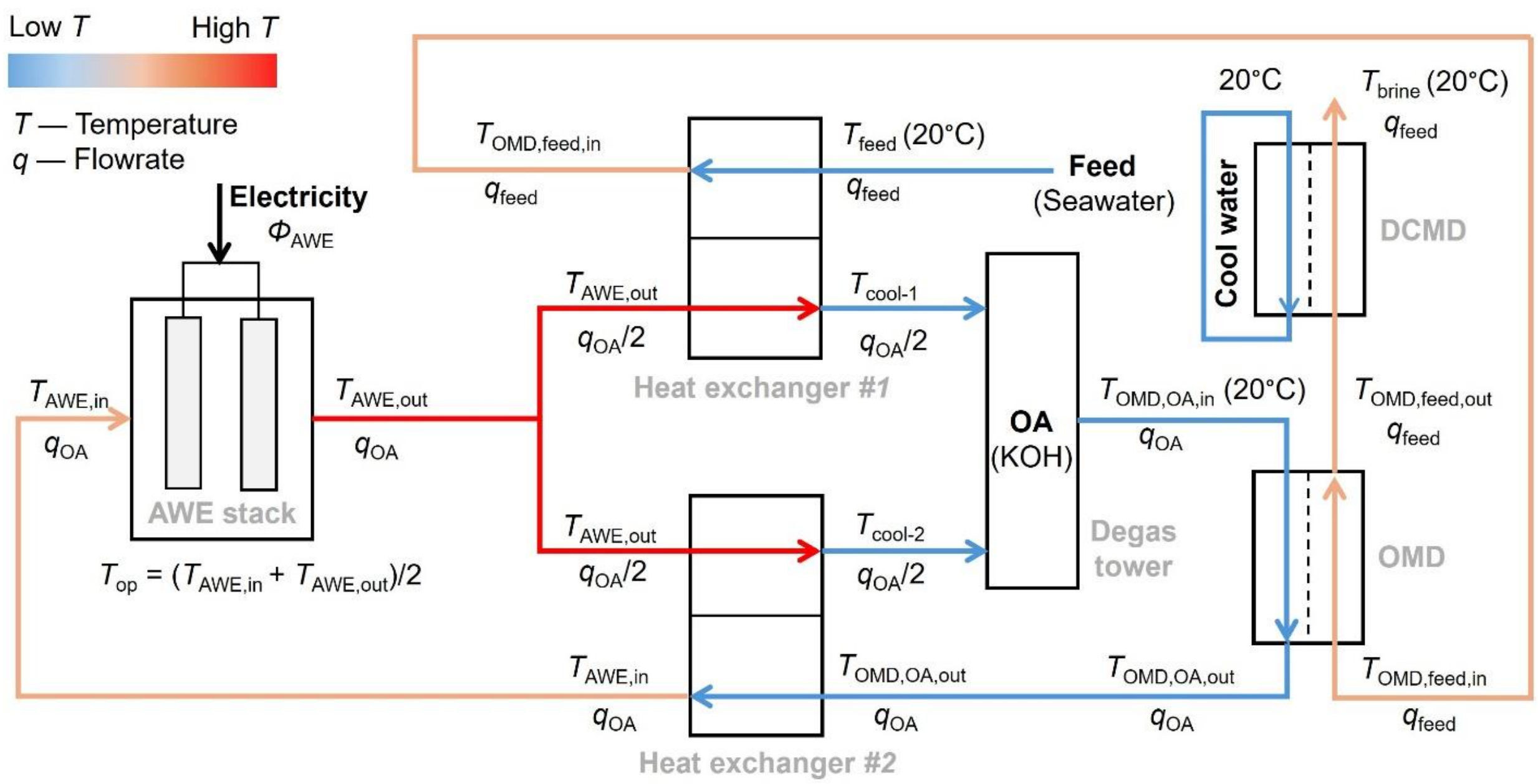

Fig. 4b. Schematic diagram showing the thermal balance and stream flow paths.

Incorporating these equations into our model, we could evaluate the impact of ε on the thermally symbiotic OMD-AWE system considering ε ranging value from 0.6 to 0.95 and determine design parameters for heat exchangers (Supplementary Fig. 3). The simulation considers a single-cell electrolyser operated at a current density of 0.5 A cm^{-2} (using a Ni nanowire-based cathode and NiFe-based anode separated by a ZIRFON diaphragm, same as our experimental setup) integrated with 1-cm^2 membrane. We set the AWE outlet temperatures ($T_{AWE,out}$) at 50°C and the OMD feed temperature ($T_{OMD,feed,in}$) at 40°C. The results reveal that reducing ε from 0.95 to 0.60 leads to a 26% reduction in both water flux and H_2 production flux (Supplementary Fig. 3a), due to reduced

heat recovery, which compromises the temperature gradient across the OMD unit. At lower effectiveness levels, AWE operates at a slightly lower temperature, resulting in a higher operating voltage that generates additional waste heat to compensate for the reduced thermal recovery (Supplementary Fig. 3b). Despite this voltage increase, the specific energy consumption (*SEC*) shows no significant increase. This is because the system output (water and hydrogen production) scales down with the thermal limits, ensuring that the energy input per unit of product remains stable. Additionally, to maintain the target OMD feed temperature of 40°C under limited heat transfer (i.e., reduced ε), the system flowrates must be reduced (Supplementary Fig. 3c). While necessary for thermal balance, this reduction limits the latent heat transfer potential, contributing to the lower water flux. From a heat exchanger design perspective, the sizing analysis highlights a clear trade-off between performance and compactness (Supplementary Fig. 3d). An effectiveness of $\varepsilon = 0.60$ corresponds to a *NTU* of 1.1–1.6 and requires a heat exchanger area less than half the size of the membrane ($R_{hx} < 0.5$). This configuration is highly compact and easily achievable with low-cost shell-and-tube exchangers. Achieving $\varepsilon = 0.9$ requires an *NTU* of 3.4–4.6, corresponding to R_{hx} of 1.53–2.08 (i.e., 53–108% larger than the membrane area). Although this requires high-performance plate-type heat exchangers, the footprint remains comparable to the membrane area, ensuring the system remains spatially viable. We also provided the inlet and outline temperature of the two heat exchangers for your reference (Supplementary Table 2). These results suggest that while high-effectiveness heat exchangers maximise H_2 yields, the system functions well even with lower-effectiveness alternatives. This flexibility allows optimisation based on space and cost constraints rather than requiring premium heat exchanger designs.

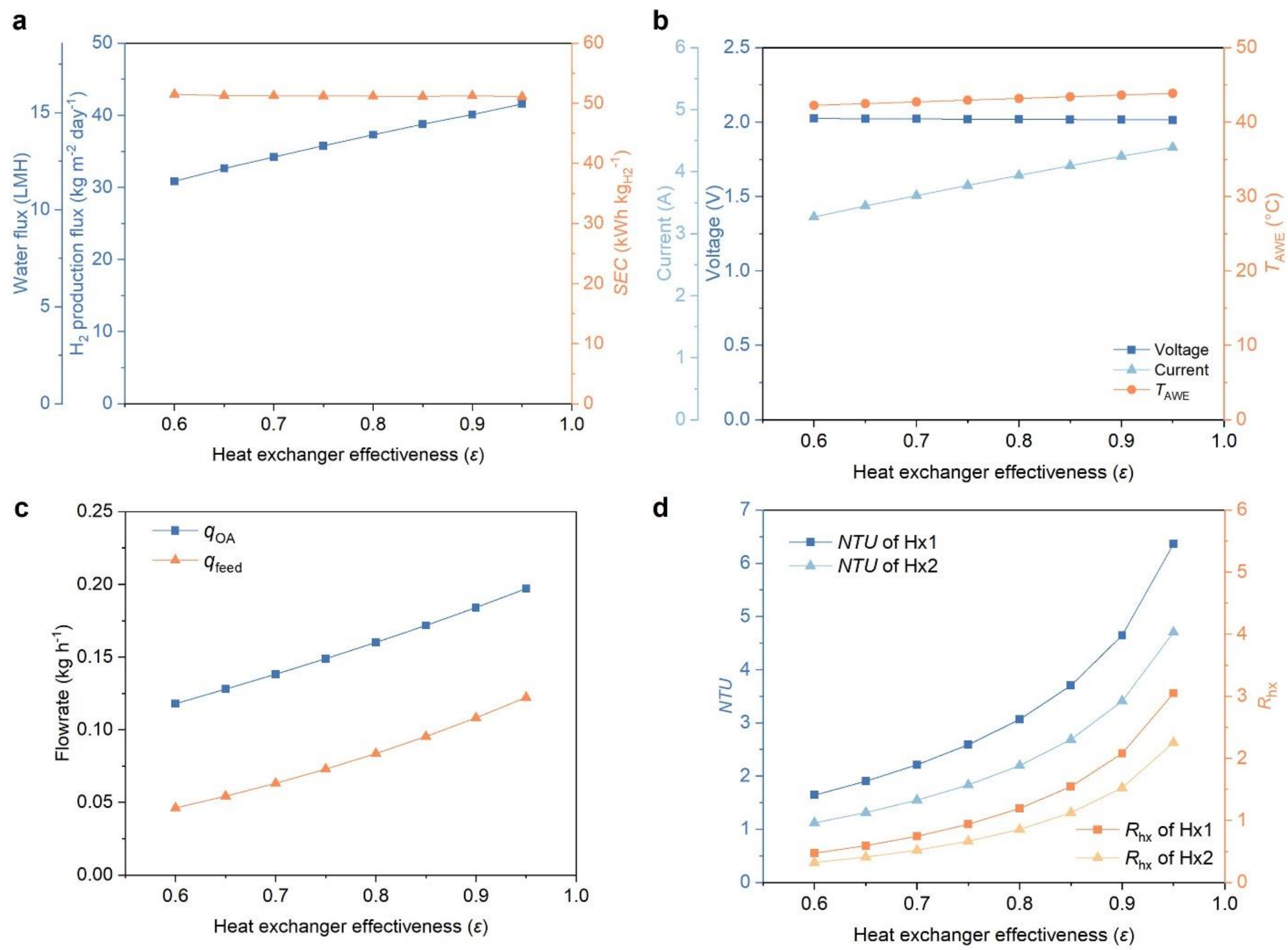

Supplementary Fig. 3 The impact of heat exchangers. a water flux, H_2 production flux, and SEC)of the thermally symbiotic OMD-AWE as a function of ε; **b** the current, voltage, and operating temperature of AWE as a function of ε; **c** the flowrate of KOH osmotic agent (q_{OA}) and that of feed seawater (q_{feed}) as a function of ε; and **d** *NTU* and R_{hx} as a function of ε. (Conditions: The simulation considers a single-cell electrolyser operated at a current density of 0.5 A cm^{-2} using a Ni nanowire-based cathode and NiFe-based anode separated by a ZIRFON diaphragm)

Supplementary Table 2 The inlet and outline temperature of heat exchangers (Hx1 and Hx2).

ε	Temperatures in Hx1 (°C)				Temperatures in Hx2 (°C)			
	$T_{AWE,out}$	T_{cool-1}	$T_{OMD,feed,in}$	T_{feed}	$T_{AWE,out}$	T_{cool-2}	$T_{AWE,in}$	$T_{OMD,OA,out}$
0.95	50.0	21.5	40.0	20.0	50.0	27.8	37.7	26.7
0.9	50.0	23.0	40.0	20.0	50.0	29.2	37.3	26.9
0.85	50.0	24.5	40.0	20.0	50.0	30.5	36.8	27.1
0.8	50.0	26.0	40.0	20.0	50.0	31.8	36.4	27.3
0.75	50.0	27.5	40.0	20.0	50.0	33.1	35.9	27.4
0.7	50.0	29.0	40.0	20.0	50.0	34.3	35.4	27.6
0.65	50.0	30.5	40.0	20.0	50.0	35.5	35.0	27.7
0.6	50.0	32.0	40.0	20.0	50.0	36.7	34.5	27.8

Finally, to examine the thermal efficiency (η), we estimated the heat loss for a 1 MW-scale OMD-AWE system by scaling up our 8.56 W lab unit (2.02 V,4.25 A). Based on a stack efficiency of 73.4%, the 1 MW system generates ~266 kW of waste heat. Referring to the sizing analysis in Supplementary Fig. 3d, the required internal heat exchange areas for Hx1 and Hx2 are 24.3 m^2 and 17.8 m^2, respectively (for $\varepsilon = 0.9$). Assuming a standard plate heat exchanger packing density of 50 m^2 m^{-3}, the corresponding unit volumes are 0.49 m^3 and 0.36 m^3. We modeled these units with Length (L) × Width (W) × Height (H) of 1.0 × 0.5 × 1.0 m for Hx1 and L × W × H = 1.0 × 0.5 × 0.7 for Hx2, resulting in external surface areas of 4.0 m^2 and 3.1 m^2, respectively. We further assumed thermal insulation using Aluminum-clad polyurethane (PU) foam (k_{ins} = 0.024 W m^{-1} K^{-1}) with a thickness (d_{ins}) of 20 mm. The heat loss through heat exchangers ($\Phi_{loss,Hx}$) was calculated using Eq. R8:

$$\Phi_{loss,Hx} = \frac{k_{ins} \cdot S_{hx} \cdot \left(T_{AWE,out} - T_{amb}\right)}{d_{ins}} \quad (R8)$$

Assuming an ambient temperature (T_{amb}) of 20°C, the calculated $\Phi_{loss,Hx}$ for Hx1 and Hx2 are 144 W and 111 W, respectively. These losses represent <0.05% of the 266-kW waste heat, confirming that thermal efficiency approaches 100% at the industrial scale.

These are revised in the **newly revised manuscript:**

Lines 404–417: "The two heated KOH streams, flowing at equal flowrates, then heat the feed (with flowrate q_{feed} and temperature T_{feed}) from RT to the target OMD inlet temperature ($T_{OMD,feed,in}$), and heat the recirculated KOH leaving the OMD module at $T_{OMD,OA,out}$ to AWE inlet at $T_{AWE,in}$ through two heat exchangers (Hx1 and Hx2), respectively (Eqs. 6–7).

$$\Phi_{Hx,1} = \varepsilon \cdot c_{KOH} \frac{q_{OA}}{2}\left(T_{AWE,out} - T_{feed}\right) = c_{seawater} q_{feed}\left(T_{OMD,feed,in} - T_{feed}\right) = c_{KOH} \frac{q_{OA}}{2}\left(T_{AWE,out} - T_{cool,1}\right) \quad (6)$$

$$\Phi_{Hx,2} = \varepsilon \cdot c_{KOH} \frac{q_{OA}}{2}\left(T_{AWE,out} - T_{OMD,OA,out}\right) = c_{KOH} q_{OA}\left(T_{AWE,in} - T_{OMD,OA,out}\right) = c_{KOH} \frac{q_{OA}}{2}\left(T_{AWE,out} - T_{cool,2}\right) \quad (7)$$

where $s_{eawater}$ is the specific heat capacity of seawater (4.02 kJ kg^{-1} K^{-1})[39,40], Φ_{hx} is the heat flow through heat exchangers, T_{cool} is the KOH temperature exiting the heat exchangers, and ε is the heat exchanger effectiveness (i.e., the ratio of actual heat transfer to maximum possible heat transfer), >90% using a counter-flow plate-type configuration). The impact of ε on system performance is detailed in Supplementary Note 3." with details in Supplementary Note 3: Heat exchanger design and effectiveness analysis

References:

1. Teke, İ., Ağra, Ö., Atayılmaz, Ş. Ö. & Demir, H. Determining the best type of heat exchangers for heat recovery. *Appl. Therm. Eng.* **30**, 577–583 (2010).

Comment 6:

In Figure 1, regarding Heat Exchanger 2, could you please clarify the outlet temperature of the cold stream? Is it possible for it to be heated up to 60°C, since it flows directly into the AWE after passing through the heat exchanger? This seems unlikely, as theoretically the cold stream temperature should not exceed that of the hot stream.

Reply:

We sincerely thank you for this careful observation. Your thermodynamic intuition is correct; the cold stream exiting heat exchanger #2 ($T_{AWE,in}$) cannot, and does not, exceed the temperature of the incoming hot stream ($T_{AWE,out}$).

To revise this, first, we need to clarify that in the newly revised manuscript, we recommend the AWE outlet temperature (i.e., $T_{AWE,out}$) at ~50°C instead of 60°C, because we found that $T_{AWE,out}$ approaching to the target OMD's feed inlet temperature ($T_{OMD,feed,in}$) of 40°C would result in a higher H_2 production flux since AWE operated at lower temperatures could generate more waste heat (Fig. 4c). Therefore, to answer your questions regarding the outlet temperature of the cold stream in Heat Exchanger 2, we use 50°C as an example rather than 60°C.

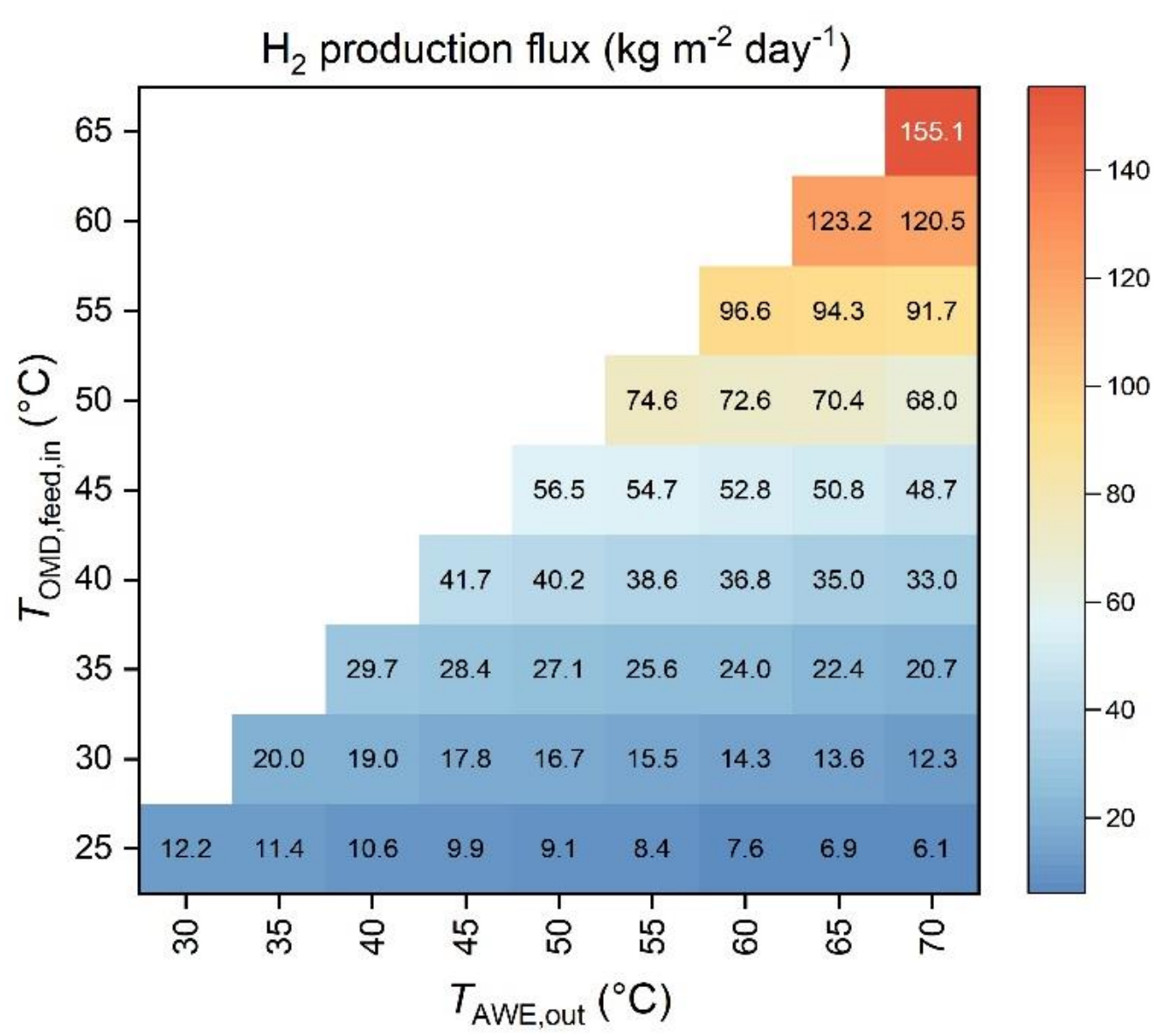

Fig. 4c H_2 production flux as functions of AWE outlet temperature ($T_{AWE,out}$) and OMD's feed inlet temperature ($T_{OMD,feed,in}$).

Within the heat exchanger #2, the hot stream cools down from $T_{AWE,out}$ to T_{cool-2}, while the cold stream is heated from $T_{OMD,OA,out}$ to $T_{AWE,in}$. The final temperature of this cold stream ($T_{AWE,in}$) is strictly governed by the heat exchanger effectiveness (ε, with its impacts provided in Comment 5) and the available waste heat, which varies with the AWE stack efficiency (γ). As detailed in our updated model, when $T_{AWE,out}$ is controlled at 50°C, the $T_{AWE,in}$ only reaches between 35.1°C and 41.5°C as γ increases from 0.5 to 0.9. This confirms that the cold stream is always below the hot stream temperature, perfectly adhering to thermodynamic limits.

Therefore, regarding the former Figure 1, the temperatures are not meant to represent a stream-by-stream temperature map, but are simplified illustrations of the engineering setpoints. However, we agree that this should be explicitly clear to the reader. Thus, we have updated **Figure 1** to display the detailed stream temperatures across the heat exchangers. In the revised Figure 1, we have added specific notations: a single asterisk (*) denotes fixed engineering setpoints, while a double asterisk (**) indicates temperature ranges that vary depending on the AWE stack efficiency.

Supplementary Table. The inlet and outlet temperature of Heat exchanger #2 changed with γ.

γ	$T_{AWE,in}$	$T_{OMD,feed,out}$	$T_{AWE,out}$	T_{cool-2}
/	°C	°C	°C	°C
0.5	35.1	23.0	50.0	25.7
0.6	35.8	24.2	50.0	26.8
0.7	36.8	26.0	50.0	28.4
0.8	38.2	28.6	50.0	31.0
0.9	41.5	33.1	50.0	34.8

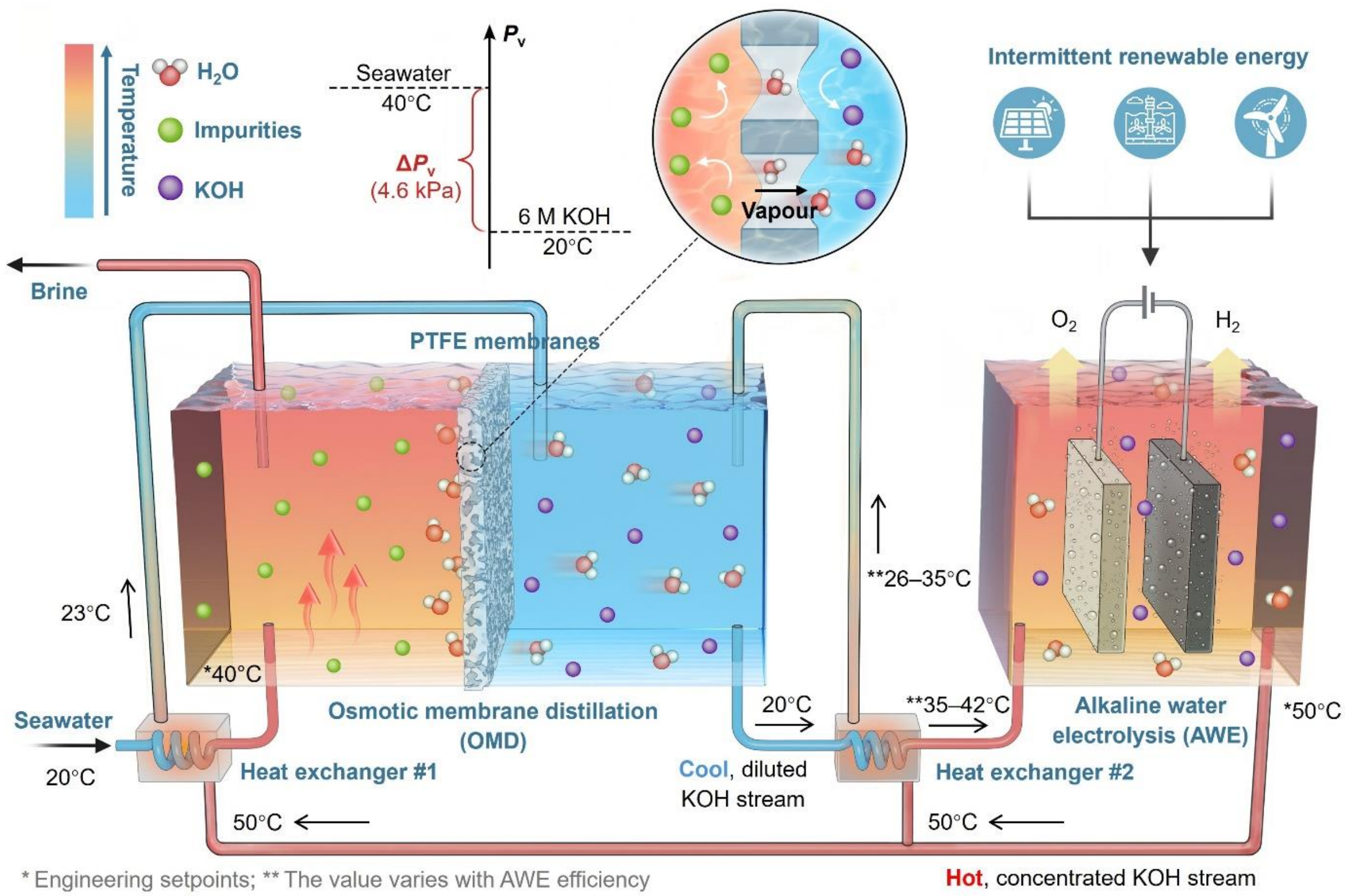

Fig. 1 Direct green H_2 production from seawater via a thermally symbiotic OMD-AWE system. The design utilises precisely designed self-sustaining heat exchange loops to convert the waste heat from AWE into the primary driver of the OMD process. This thermal integration enables OMD operation at an optimal 40°C feed with 6 M KOH as osmotic agent to deliver high water flux with minimal fouling and wetting risks. In return, the OMD provides passive cooling and a continuous supply of pure water, enabling AWE to operate at 50°C for efficient H_2 production.

These are added to the **newly revised manuscript**:

Lines 97–98: "Simultaneously, defining AWE's thermal compatibility is essential for maximising overall system efficiency."

Lines 397–402: "

$$\Phi_{AWE} = (U - U_{th})i_{cell}N_{cell} = SEC_t\left(\frac{1-\gamma}{\gamma}\right)r_{H_2} = c_{KOH}q_{OA}\left(T_{AWE,out} - T_{AWE,in}\right) \quad (4)$$

U_{th} (1.48 V) represents the thermoneutral voltage for water electrolysis, which translates to theoretical *SEC* (SEC_t) of 39.4 kWh kg^{-1} based on high heat value (HHV) of H_2. U could be determined from AWE polarisation curves as functions of i_{cell} and T_{op}. γ represents electrolyser stack efficiency which equals to U_{th}/U."

Lines 436–449: "By solving Eqs. 4–11 with treating $T_{OMD,feed,in}$ and $T_{AWE,out}$ as input varibles, we simulated how thermal integration improves H_2 yields of a commercial single-cell AWE electrolyser (0.5 A cm^{-2}, nanowire/NiFe electrodes and ZIRFON diaphragm) integrated with a 1-cm^2 membrane (Fig. 5c). Increasing $T_{OMD,feed,in}$ boosts H_2 flux due to increased water supply, while increasing $T_{AWE,out}$ decreases H_2 flux at any fixed $T_{OMD,feed,in}$ because higher AWE temperatures improve electrochemical kinetics but reduces waste heat generation. To maintain $T_{OMD,feed,in}$, in despite reduced heat input, the system must decrease OA flowrate, which results in a larger temperature drop across the membrane due to latent heat transfer and reduces water flux. Our earlier trade-off analyses established that operating OMD above 40°C risks membrane scaling and wetting; therefore, $T_{OMD,feed,in}$ must be capped at 40°C to ensure long-term durability. Along the 40°C feed boundary, the system still delivers exceptional H_2 fluxes over 40 kg m^{-2} day^{-1} with $T_{AWE,out}$ controlled at ~50°C. This demonstrates that AWE does not need high temperatures but a 5–10°C higher temperature represents the thermodynamic sweet spot that balances high H_2 yields with membrane stability."

Lines 459–462: "As OMD relies on AWE waste heat, a critical concern is whether this thermally symbiotic architecture can be sustained across highly efficient electrolysers and different configurations. Electrolysers vary widely in electrode materials, cell architectures, and current densities, which dictate their overall γ and available waste heat."

Responses to comments of Reviewer 2:

In this article, the authors reported a thermally symbiotic osmotic membrane distillation-electrolysis (OMD-AWE) system for direct hydrogen production from seawater. The work featured a clever thermal management design that converted waste heat from the electrolysis process into the driving force for membrane distillation, simultaneously addressing water supply and system cooling challenges. The concept was novel and held significant application potential. However, the mechanistic explanations at the material level and the experimental support for key conclusions remained relatively weak. Therefore, the manuscript could be reconsidered for publication Nature communication after major revision.

Q1: The authors should conduct an in-depth analysis and clarify the impact of fluctuating potentials in practical operating environments—such as those caused by the intermittency of renewable energy power supply—on the system's stability. The current study was conducted under constant current/potential conditions, which does not align with the actual production scenarios for most green hydrogen. The referenced article (Nature. 2025, 639, 360–367. and Nat. Sustain. 2025, https://doi.org/10.1038/s41893-025-01665-y.) elucidated the corrosion issues associated with potential fluctuations in seawater electrolysis. Consequently, evaluating the system's performance degradation, membrane wetting risks, and electrode corrosion behaviour under dynamic potential conditions is of critical importance.

(1) If intermittent square or sawtooth wave potentials simulating renewable energy sources (such as photovoltaic power generation) are applied, what observable degradation would occur in the system's hydrogen production Faraday efficiency, water flux across the membrane module, and ion rejection rate?

(2) Potential fluctuations may accelerate passivation/dissolution of electrode surfaces or cause localized overheating of PTFE membranes. Please provide corresponding experimental evidence or theoretical simulations to demonstrate the chemical and physical stability of the system's core materials (Ni/NiFe electrodes and PTFE membranes) under dynamic operating conditions.

(3) Please discuss whether your thermal-water-hydrogen model possesses the capability to incorporate fluctuating potential as an input parameter and predict system performance (such as hydrogen production rate and thermal balance). If not, how should the model be improved to accommodate such practical operating conditions?

Reply:

We really thank you for raising this critical issue regarding the impact of intermittent power inputs. First, regarding the material stability, our membrane unit and electrolyser are physically decoupled, and intermittent power would not affect the membrane's intrinsic properties, such as permeability and salt rejection. In addition, since we used commercial AWE components, the system would face the same general challenges under intermittency as any standard AWE system [1]. While catalyst stability is indeed a prerequisite for long-term operation, these are inherent challenges to the AWE

technology itself rather than specific to our water-energy coupling mechanism. These issues can be mitigated by advanced catalyst designs, like redox-mediated self-healing anodes [2] or dynamic passivation cathodes [3] as mentioned in the two literatures. Our system is compatible with these intermittency-resistant catalysts due to the chemical inertness of PTFE against different electrolyte environments, e.g., acidic or alkaline media (see Comment 9). Additionally, standard system-level solutions, such as battery buffering [4], can be implemented to smooth fluctuations.

Crucially, your comment has inspired us to re-evaluate the system's dynamic response, revealing a unique advantage of the thermally symbiotic OMD-AWE, i.e., its ability to maintain water and thermal balance under intermittent power input. In membrane-integrated electrolysis, the most significant risk is the cumulative mismatch between water production and water consumption[5–7], leading to either system dry-out or overflow. While buffer tanks can temporarily mitigate this issue, chronic asynchronous operation inevitably leads to catastrophic system failure. If water production exceeds consumption (e.g., during power lulls), the continuous influx of water causes electrolyte dilution. This not only increases ohmic resistance but also overflows the closed-loop system and collapses the osmotic driving force required for subsequent high-power operation. Conversely, if consumption exceeds production (e.g., during peak power), the system suffers from progressive dry-out. The continuous removal of water causes KOH concentration to spike, leading to corrosion, localised overheating, and KOH crystallisation that blocks electrolyser flow channels. Managing this mismatch via frequent shutdown and restart cycles is destructive to hydrophobic membranes. Shutdown creates stagnation, high-salinity boundary layers at the membrane where salts quickly become supersaturated and crystallise on and within pores, forming $CaCO_3$ and gypsum that may cause irreversible scaling. Frequent hydraulic shocks from pump restarts cause mechanical fatigue in the polymer matrix, lowering liquid entry pressure (LEP) and inducing pore wetting. Therefore, maintaining water balance is crucial for long-term operation. Rather than relying on destructive on/off control or buffer tanks, our OMD-AWE system achieves dynamic self-regulation. Since the OMD thermal driving force (waste heat) is phase-locked to the electrolysis, the system naturally adjusts water production in exact proportion to electrolyser demand, ensuring continuous and synchronous operation without frequent shutdowns. Following your suggestions, we have updated our model to treat power as the input variable. The enhanced model now not only guides steady-state design for system build-up but also allows to predict water and thermal balance and system performance. Our dynamic analysis, detailed below, confirms that the system maintains stable operation even under highly variable renewable energy inputs based on actual weather conditions.

First, we defined the system's optimal configuration. We considered a commercial single-cell AWE electrolyser operated at a current density of 0.5 A cm^{-2} (using a Ni nanowire-based cathode and NiFe-based anode separated by a ZIRFON diaphragm) integrated with 1-cm^2 membrane unit. We set AWE outlet temperatures ($T_{AWE,out}$) at 50°C and the OMD's feed temperature ($T_{OMD,feed,in}$) at 40°C in our model, which then generated the optimal configurations including AWE's rated power (8.56 W), current (4.25 A), and voltage (2.02 V), heat exchanger parameters (e.g., the Number of Transfer Units (*NTU*) which represents the dimensionless thermal size of the

exchanger), and other operating parameters such as flowrate and temperature of each stream for steady-state operation. These are summarised in Supplementary Fig. 4. We then transitioned the model from a static design framework to a dynamic simulation tool to account for intermittent renewable energy. A Maximum Power Point Tracking (MPPT) DC-DC converter is usually used to optimise power transfer between the source (e.g., PV panel, wind turbine) and electrolyser. This MPPT converter dynamically optimises voltage and current levels to match the electrolyser's polarisation curve as input power fluctuates, ensuring maximum utilisation. Therefore, to predict system performance under varying power (P), the relationship between AWE's voltage (U, V), cell current (I_{cell}, A), and operating temperature (T_{op}, °C) needs to be established, which could be derived from fitting the varied power with polarisation curves of electrolysis. Using our electrode materials with 7.5 cm^2 area as an example, U as a function of I_{cell} and T_{op} were established as shown in Eq. R9 which was further incorporated into the MPPT power fitting with a typical conversion efficiency (η_{MPPT}) at 95% from the output power of source power (P_s) to that of AWE (P_{AWE}) (Eq. R10).

$$U = \left(0.00003{T_{op}}^2 - 0.0026T_{op} + 0.2248\right)\ln I_{cell} + \left(0.00003{T_{op}}^2 - 0.0062T_{op} + 1.9314\right) \quad (R9)$$

$$P_s \cdot \eta_{MPPT} = P_{AWE} = U \cdot I_{cell} \quad (R10)$$

These value of U and I_{cell} were then connected to the AWE's waste heat generation (Φ_{AWE}) (Eq. R11).

$$\Phi_{AWE} = (U - U_{th})I_{cell}N_{cell} \quad (R11)$$

where U_{th} (at 1.48 V) represents the thermoneutral voltage for water electrolysis, N_{cell} is the number of electrolyser cell (at 1 for a single-cell electrolyser). By incorporating these equations into our model, we simulated the dynamic thermal responses to varied power inputs. Using P_{AWE} as the input variable, the model calculates the current/voltage relationship and stream temperatures while other operating parameters (e.g., flowrate, *NTU*) remain constant. These results further determine system performance like water flux, H_2 production flux, and specific energy consumption (*SEC*), i.e., energy consumption for producing per kg of H_2.

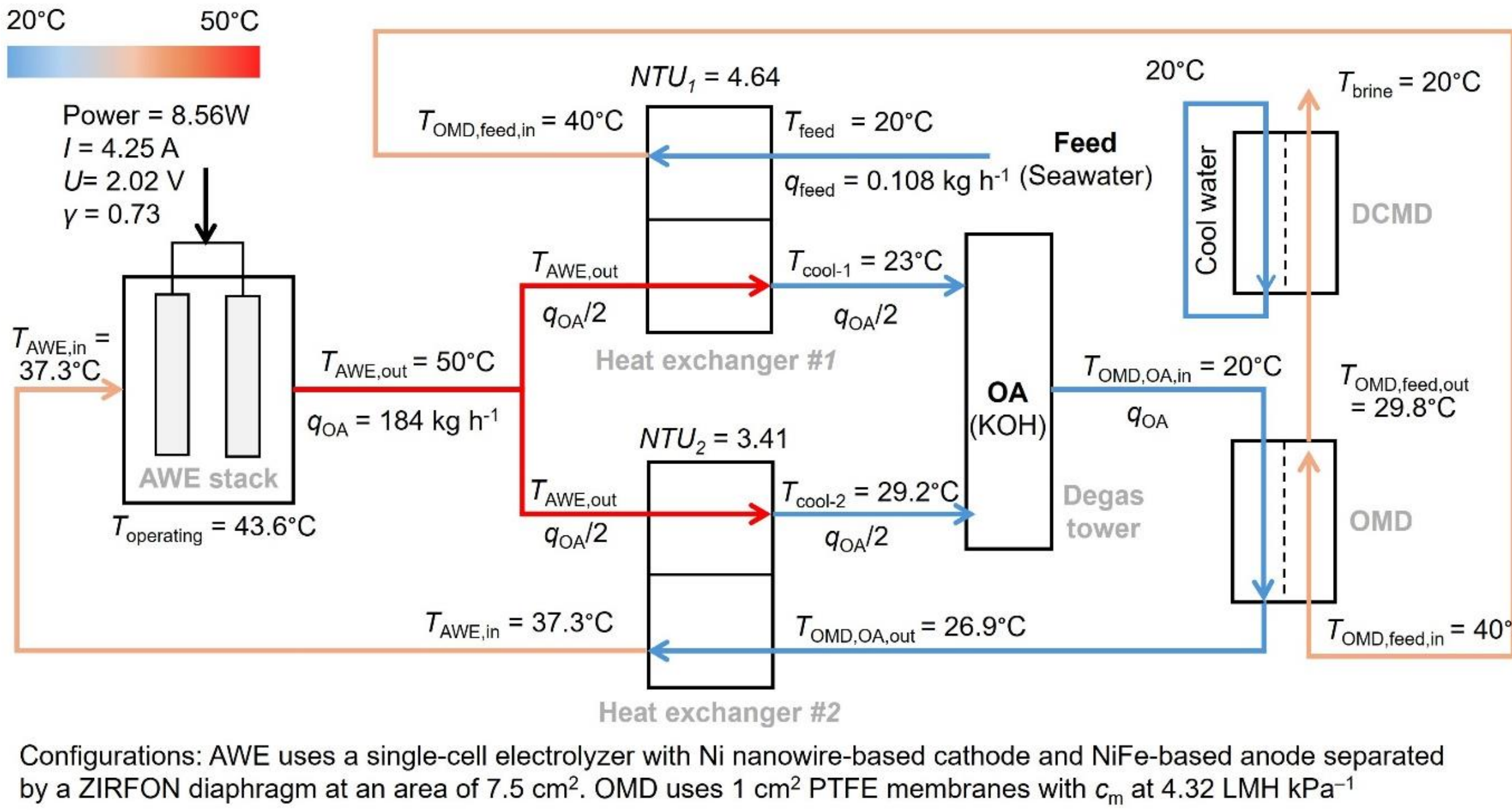

Supplementary Fig. 4 The designed OMD-AWE configuration and operating parameters used for dynamic response simulation under intermittent power input.

To evaluate the system's stability under intermittent power input, we first simulated the system's thermal responses where $T_{AWE,out}$ and $T_{OMD,feed,in}$ are treated as representative dependent variables as the power input decreases from the rated condition (8.56 W) down to zero (Fig. 5a). At rated power, the electrolyser operates at high current density, generating significant waste heat that maintains $T_{AWE,out}$ and $T_{OMD,feed,in}$ at their set values of 50°C and 40°C, respectively. As the power input drops, the AWE's current reduces proportionally (see inserted figure), which leads to a direct reduction in water consumption based on Faraday's Law where mass flux is proportional to current. Simultaneously, the lower power input reduces heat generation in AWE, causing the system to cool down naturally towards the ambient 20°C. This temperature drop reduces the thermal gradient across membranes, thereby lowering the water production. Since the reduced current also requires less water for electrolysis, the water supply via OMD passively scales down to match the AWE demand. This synchronisation demonstrates the system's self-regulating feedback mechanism, which manages power intermittency without requiring active thermal control. As shown in Fig. 5b, H_2 production flux decreases linearly with reduced power input, while energy efficiency slightly improves at partial loads. This efficiency improvement occurs because the lower current density significantly reduces ohmic voltage losses. Although the cooled system increases kinetic overpotentials slightly, the reduction in resistive losses dominates, resulting in a 25% decrease in *SEC*. This behaviour demonstrates that the system naturally transitions to a higher-efficiency operating mode during intermittent power conditions.

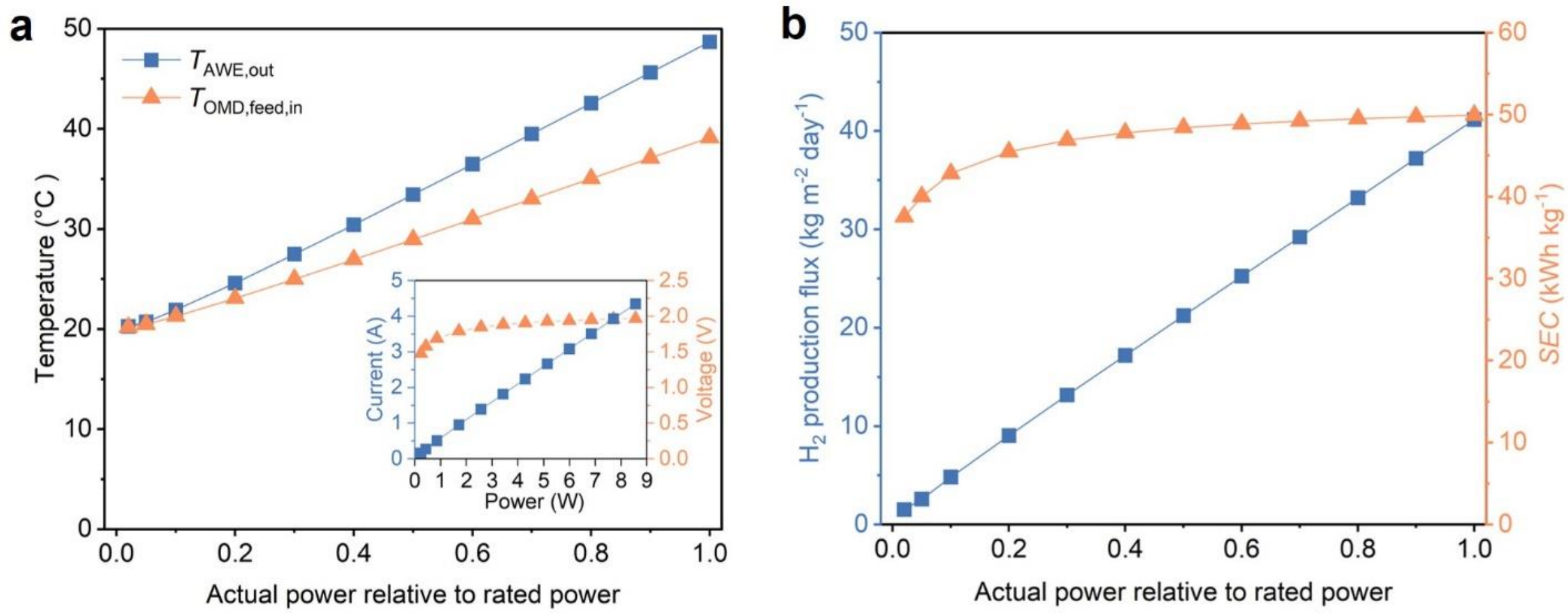

Fig. 5 The dynamic response of thermally symbiotic OMD-AWE under intermittent power input. a the changes in $T_{AWE,out}$ and $T_{OMD,feed,in}$ as a function of the AWE power; **b** the changes in H_2 production flux and *SEC* as a function of the AWE power; **c** the comparison in water balance between OMD-AWE and PVT-MD-AWE under intermittent solar power input.

To examine if the system can maintain stable operation under realistic intermittent power input, we evaluated its water balance under realistic solar and wind power scenarios. For solar energy, we integrated a standard PV module coupled with an MPPT algorithm. The simulation determines the PV cell temperature (T_{PV}, °C) as a function of ambient air temperature (T_{amb}, °C) and solar irradiance (G, W m^{-2}), using the nominal operating cell temperature of PV (T_{NOCT}, °C) under T_{amb} of 20°C and G of 800 W m^{-2} (typically 48°C), as shown in Eq. R12.

$$T_{PV} = T_{amb} + \frac{G}{800}(T_{NOCT} - 20) \quad (R12)$$

This temperature is used to calculate its instantaneous efficiency (η_{PV}) by comparing T_{PV} to its standard 25°C value using Eq. R13, where η_{stc} is the rated PV efficiency at 25°C (considering 20%) and β is the temperature coefficient which defines how much power is lost per °C (typically at 0.35% °C^{-1}).

$$\eta_{PV} = \eta_{stc}[1 - \beta(T_{PV} - 25)] \quad (R13)$$

Finally, the actual power delivered to the AWE (P_{AWE}) is obtained from the incident solar energy adjusted for both η_{PV} and η_{MPPT} as shown in Eq. R14, where the PV area (S_{PV}) is 0.043 m^2 based on the AWE's 8.56 W rated power under standard conditions (1000 W m^{-2}) and η_{stc} of 20%.

$$P_{AWE} = P_{PV} \cdot \eta_{MPPT} = G \cdot S_{PV} \cdot \eta_{PV} \cdot \eta_{MPPT} \quad (R14)$$

Using actual weather data from Hong Kong Observatory on 4th Feb. 2026 (Supplementary Fig. 5), we generated a P_{AWE} profile over an operational day (7:00–19:00) characterised by significant intermittency between 9:30 and 12:00 due to cloud cover as shown in Fig. 5c. The OMD-AWE's stability was indicated by a water balance ratio calculated by the water production rate through

OMD divided by the water consumption rates of AWE, both of which were obtained by fitting the P_{AWE} profile to our model. The water consumption rate is proportional to the applied current while water production rate is determined by vapor pressure gradient across the membrane as described in our Manuscript Lines 235–245. For comparison, a traditional photovoltaic thermal MD systems (PVT(MD)) was simulated under the same weather conditions using the model from Ali, A. S. & Bounahmidi (2025)[8]. As shown in Fig. 5c, our thermally symbiotic OMD-AWE exhibits a stable water balance ratio of ~1 during 9:00–17:00, despite significant power fluctuations during morning cloud cover. This indicates a strong synchronisation between water supply and demand. Even when power input changes abruptly, the waste heat generation adjusts with the current, allowing OMD production to track AWE consumption in real-time. However, outside the stable window (before 09:00 and after 17:00), the water production significantly exceeds consumption. This is because OMD's osmotic gradient enables continuous water production even with minimal thermal input, while AWE water consumption decreases substantially as the PV power reduces. To prevent excess water accumulation, feed supply should be suspended when power falls below 35% of rated power. These off periods can be used for system maintenance, including membrane cleaning and system flushing, ensuring optimal performance for subsequent cycles. In contrast, PVT(MD)-AWE shows asynchronous responses throughout the day. During morning hours, low ambient temperatures enhance PV electrical efficiency, leading to high water consumption, but insufficient thermal energy limits MD water production. At midday, high ambient temperatures maximise thermal output and water production but simultaneously reduce PV efficiency due to overheating, which reduces water consumption. This mismatch creates a parabolic water balance curve, with water balance ratios reaching up to 1.8. Similarly, we simulated a 24-h offshore wind power profile input to AWE by considering the proportional relationship between wind power and AWE power, and compared our OMD-AWE against a state-of-the-art isothermal OD-AWE system (Fig. 5d). As the wind power varies dramatically throughout the day, the OMD-AWE maintains a near-perfect water balance ratio of ~1, contrasting sharply with isothermal OD-AWE whose water balance ratio fluctuates wildly from 1 to over 10. This occurs because OD-AWE water production depends only on an osmotic gradient that remains constant regardless of power input. Over the 24-h cycle, this constant water flux causes cumulative water production in OD-AWE to exceed actual demand by ~100%. This surplus leads to severe electrolyte dilution and overflow of the closed-loop system. Since excess water mixes directly into the electrolyte, it cannot be simply discharged; extracting it requires additional energy-intensive separation processes. This creates unacceptable operational, energetic, and spatial burdens on footprint-constrained offshore wind platforms. These findings highlight the self-regulating capability of our OMD-AWE, proving it can maintain operational stability under intermittent renewable power inputs without external thermal infrastructure or complex control systems.

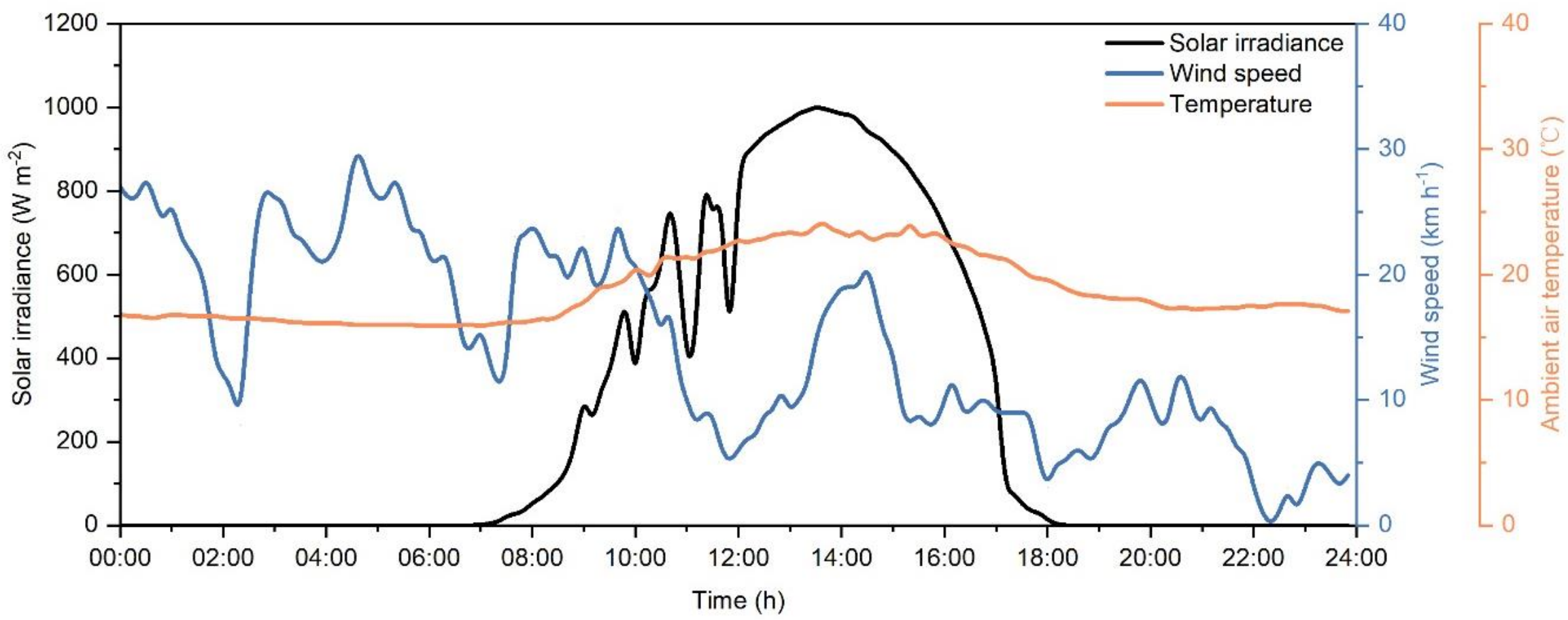

Supplementary Fig. 5 Solar irradiance, wind speed, and ambient air temperature of Hong Kong on 4th Feb. 2026 obtained from Hong Kong Observatory.

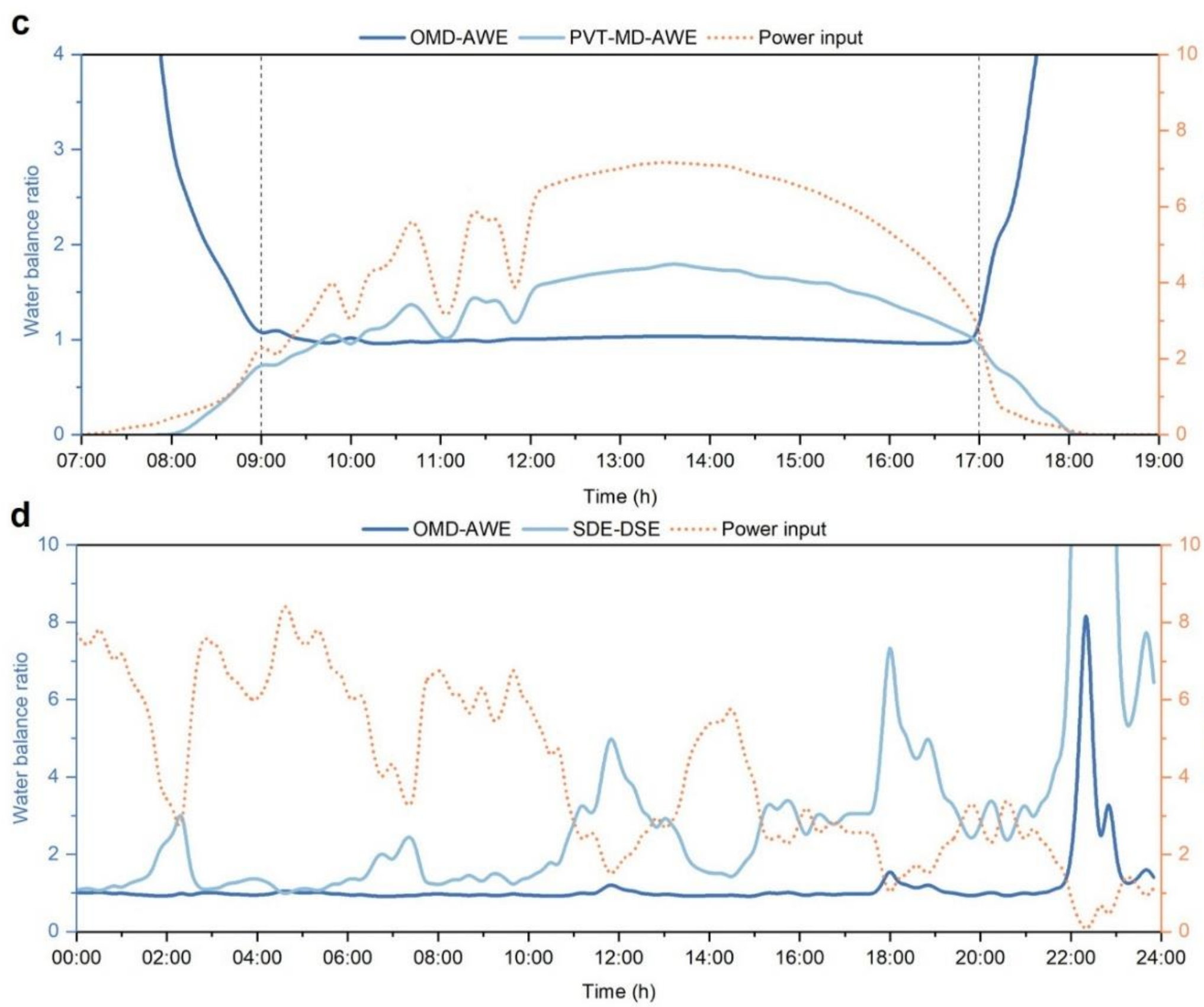

Fig. 5 The dynamic response of thermally symbiotic OMD-AWE under intermittent power input. c The comparison in water balance between OMD-AWE and PVT-MD-AWE under intermittent solar power input; **d** the comparison in water balance between OMD-AWE and SDE-DSE under intermittent wind power input.

We added a new section titled "**Dynamic self-regulation under intermittent renewable energy input**" to highlight these findings, with detailed methods provided in Supplementary Note 5.

These are added into **Abstract and conclusion**:

Lines 27–30: "This thermal symbiosis establishes a self-regulating mechanism that phase-locks thermal driving force to fluctuating electrical inputs, synchronising water supply with demand to overcome renewable intermittency."

Lines 670–672: "By buffering the fluctuations of renewable energy while co-producing surplus water and simplifying brine management, this system unlocks the potential for autonomous, decentralised, and offshore operations."

References:

1. Haoran, C., Xia, Y., Wei, W., Yongzhi, Z., Bo, Z. & Leiqi, Z. Safety and efficiency problems of hydrogen production from alkaline water electrolyzers driven by renewable energy sources. *Int. J. Hydrog. Energy* **54,** 700–712 (2023). https://doi.org/10.1016/j.ijhydene.2023.08.324
2. Li, A., Ooka, H., Kong, S. *et al.* Oxygen evolution electrocatalysis resilient to voltage fluctuations. *Nat Sustain* **8**, 1533–1540 (2025). https://doi.org/10.1038/s41893-025-01665-y
3. Sha, Q., Wang, S., Yan, L. *et al.* 10,000-h-stable intermittent alkaline seawater electrolysis. *Nature* **639**, 360–367 (2025). https://doi.org/10.1038/s41586-025-08610-1
4. Ursúa, A., Barrios, E. L., Pascual, J., Martín, I. S. & Sanchis, P. Integration of commercial alkaline water electrolysers with renewable energies: Limitations and improvements. *Int. J. Hydrog. Energy* **41,** 12852–12861 (2016). https://doi.org/10.1016/j.ijhydene.2016.06.071
5. Veroneau, S. S. & Nocera, D. G. Continuous electrochemical water splitting from natural water sources via forward osmosis. *Proceedings of the National Academy of Sciences* **118**, (2021).
6. Xie, H., Zhao, Z., Liu, T., Wu, Y., Lan, C., Jiang, W., Zhu, L., Wang, Y., Yang, D. & Shao, Z. A membrane-based seawater electrolyser for hydrogen generation. *Nature* **612**, 673–678 (2022).
7. Scheibel Cassol, G., Shang, C., An, A. K., Khanzada, N. K., Ciucci, F., Manzotti, A., Westerhoff, P., Song, Y. & Ling, L. Ultra-fast green hydrogen production from municipal wastewater by an integrated forward osmosis-alkaline water electrolysis system. *Nat Commun* **15**, 2617 (2024).
8. Ali, A. S. & Bounahmidi, T. Photovoltaic-thermal driven membrane distillation water desalination: Dynamic simulation in an actual weather condition. *Energy* **324,** 135943 (2025). https://doi.org/10.1016/j.energy.2025.135943

Q2: The authors attributed the flux decline to scaling, but the microstructural evolution of the PTFE membrane itself under long-term operation remains unclear. Could the authors comment on whether any irreversible alterations in polymer morphology—such as changes in crystallinity, pore size distribution, or surface roughness—were observed following the 500-hour test? Additional characterization data, for instance from XRD, BET analysis, or AFM, would be invaluable in clarifying the long-term structural stability of the membrane material under these operating conditions. This reference (J. Am. Chem. Soc. 2025, 147, 28, 24604–24614.) can be referred to.

Reply:

We thank the reviewer for raising this critical point regarding the microstructural evolution of the PTFE membrane. We fully agree that distinguishing between reversible scaling and irreversible polymer degradation is paramount for assessing its operational durability. To address this, we conducted a comprehensive post-mortem analysis of the PTFE membrane following the 500-h test (a now test without addition of EDTA or any other antiscalants). To ensure we were probing the intrinsic polymer structure rather than the accumulated foulant layer, the used membrane was cleaned (30 min in 0.05 M EDTA followed by a 10-min deionised water rinse) prior to characterisation. This cleaned membrane was compared directly against both the pristine membrane and the uncleaned membrane. Our characterisation involves using X-ray diffraction (XRD), atomic force microscopy (AFM), X-ray photoelectron spectroscopy (XPS), and scanning electron microscopy coupled with energy-dispersive X-ray spectroscopy (SEM-EDX), confirming the membrane experienced no irreversible microstructural or chemical changes.

SEM imaging of the cleaned membrane revealed a completely intact node-and-fibril morphology (Fig. 3e). Subsequent quantitative image analysis using ImageJ confirmed that the pore size distribution exhibited no statistically significant shift, indicating an absence of pore collapse or widening under long-term thermal and operational stress (Supplementary Fig. 16). Although a slight increase in smaller pores was observed, this likely reflects measurement artifacts from characterising PTFE's complex fibrous structure, or possibly minor residual fouling. EDX analysis of the uncleaned membrane identified inorganic scale and organic fouling, as evidenced by 2.32 atom% Ca, S, and O contents and a decreased F/C atomic ratio from 1.75 (pristine) to 1.53, respectively (Supplementary Fig. 14 and Supplementary Table 15). Following the EDTA cleaning, those Ca, S, and O contents dropped to 0 atom% and the F/C ratio recovered to 1.69, indicating the removal of all mineral scales and most carbonaceous deposits. To complete removal of organic foulants, we recommend implementing an additional cleaning protocol using high-pH alkaline cleaners, which are widely used for organic fouling control in membrane systems. XPS further confirmed this reversibility, with calcium, magnesium, carbon, and oxygen peaks diminished significantly after cleaning. Crucially, the C–F and elemental F peaks perfectly matched those of the pristine membrane, and no chlorine peaks were detected, confirming the absence of defluorination or chemical attack by chloride species (Supplementary Fig. 15). XRD analysis revealed virtually identical diffraction patterns for the pristine and cleaned membranes, with no new crystalline peaks detected (Fig. 3f). Quantitative analysis using the Hermans-Weidinger

method showed a negligible (<5%) reduction in crystallinity, proving that the continuous 20°C thermal gradient did not alter the polymer's crystalline structure. AFM analysis provided nanoscale insight, showing the average roughness (Ra) and root-mean-square roughness (Rq) of the cleaned membrane remained within a 5% margin of the pristine sample, ruling out any significant surface etching or morphological alteration (Fig. 3g). These findings demonstrate that the observed flux decline during the 500-h test was driven entirely by reversible fouling, and the PTFE membrane itself retained its structural integrity.

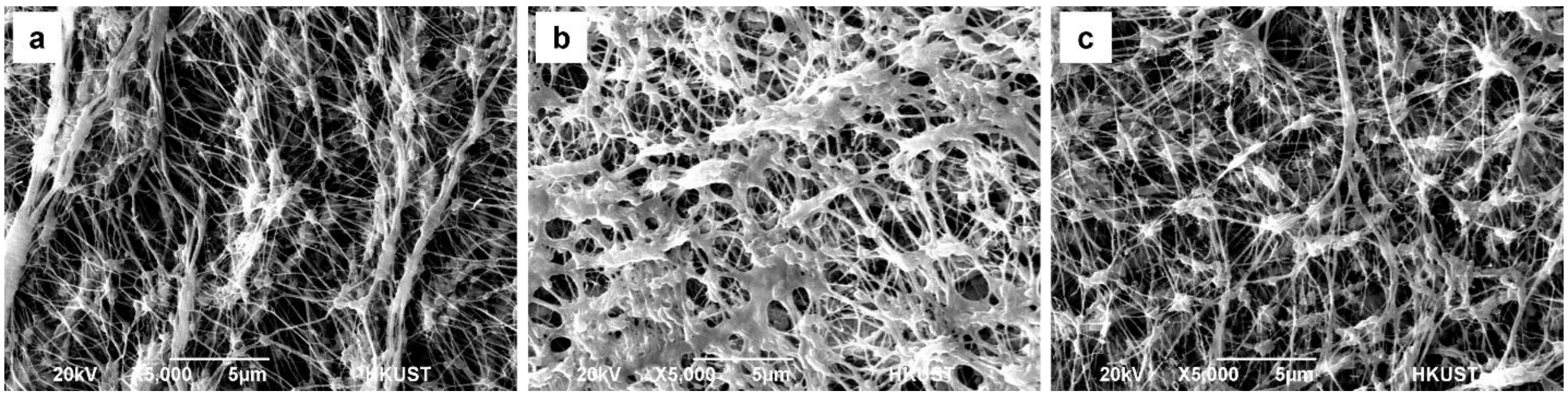

Fig. 3e SEM image showing the surface morphology of **a** the pristine PTFE membrane; **b** the uncleaned PTFE membrane after 500-h operation; and **c** the cleaned PTFE membrane after 500-h operation.

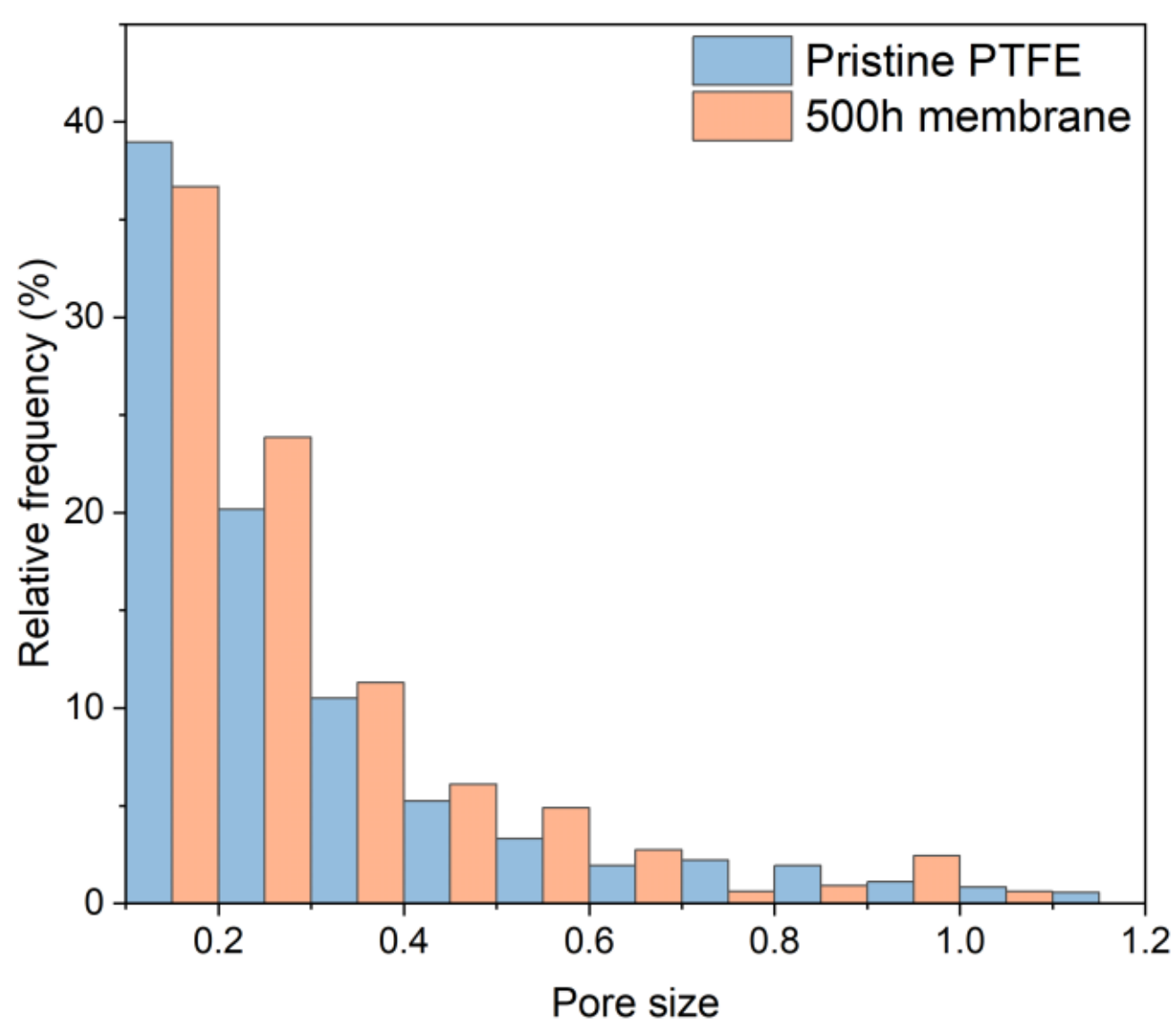

Supplementary Fig. 16 Pore size distribution the pristine PTFE membrane and the cleaned PTFE membrane after 500-h operation.

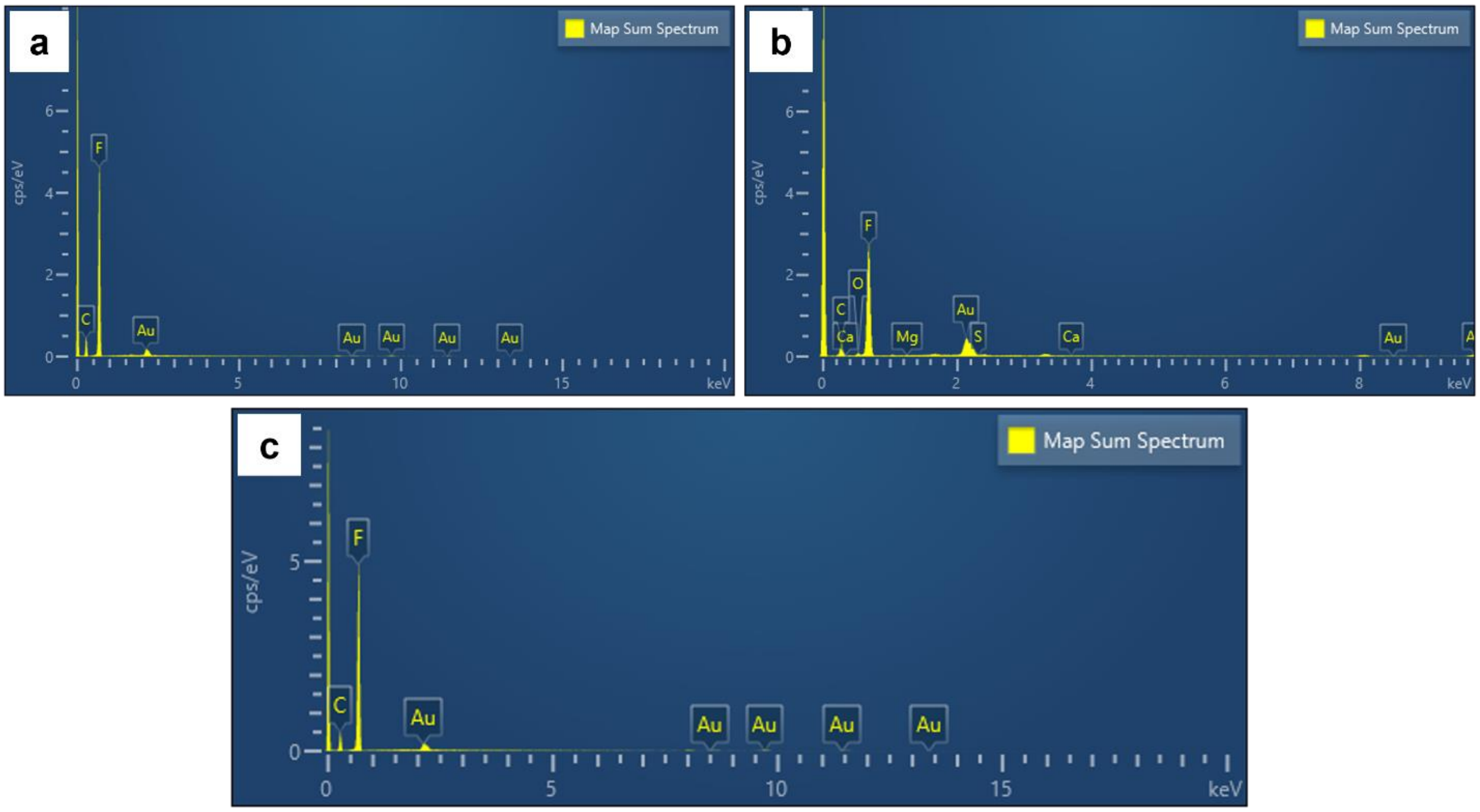

Supplementary Fig. 14 EDX spectrum showing the surface elemental composition of **a** the pristine PTFE membrane; **b** the uncleaned PTFE membrane after 500-h operation; and **c** the cleaned PTFE membrane after 500-h operation.

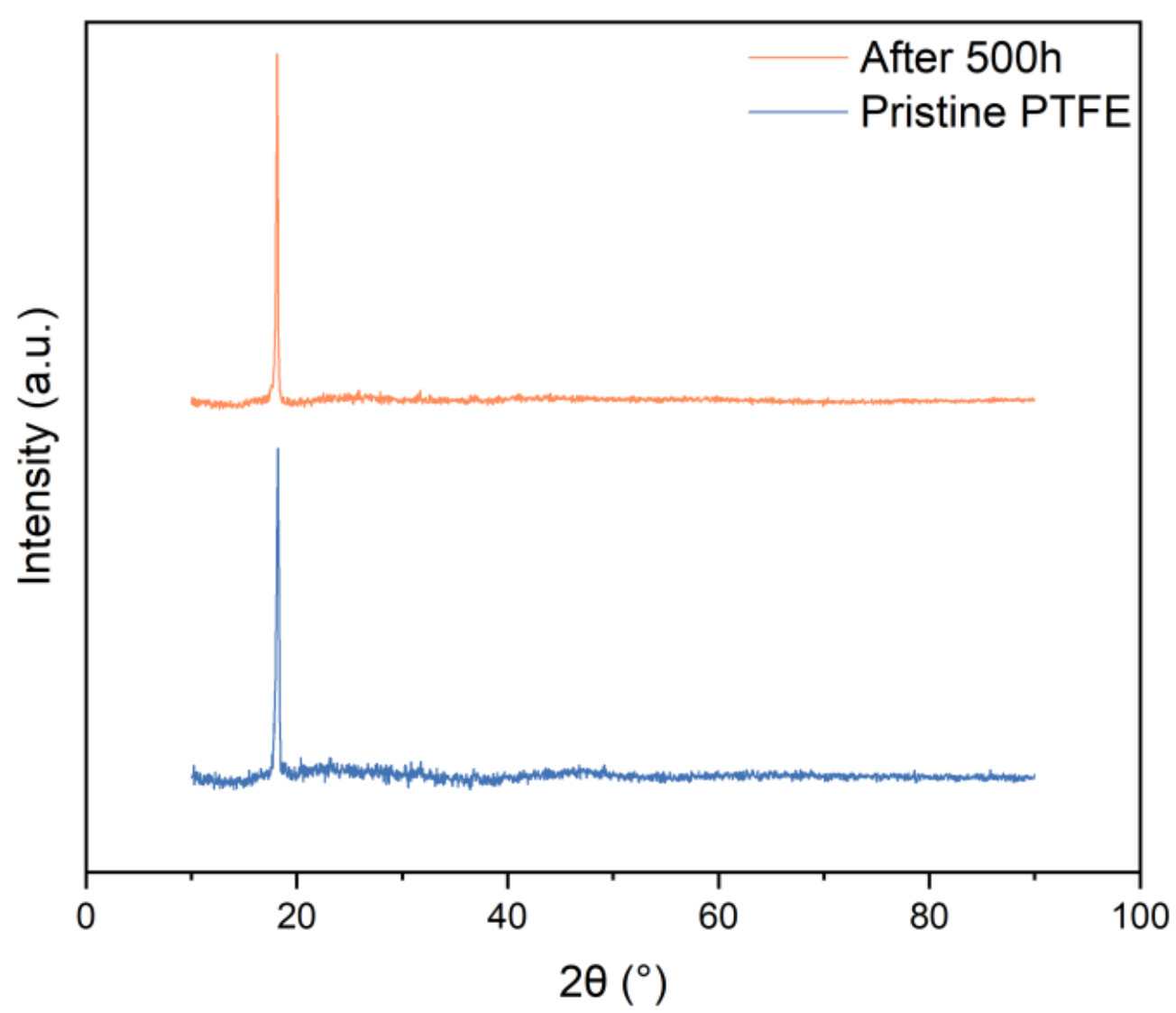

Fig. 3f XRD patterns of the pristine PTFE membrane and the cleaned PTFE membrane after 500-h operation.

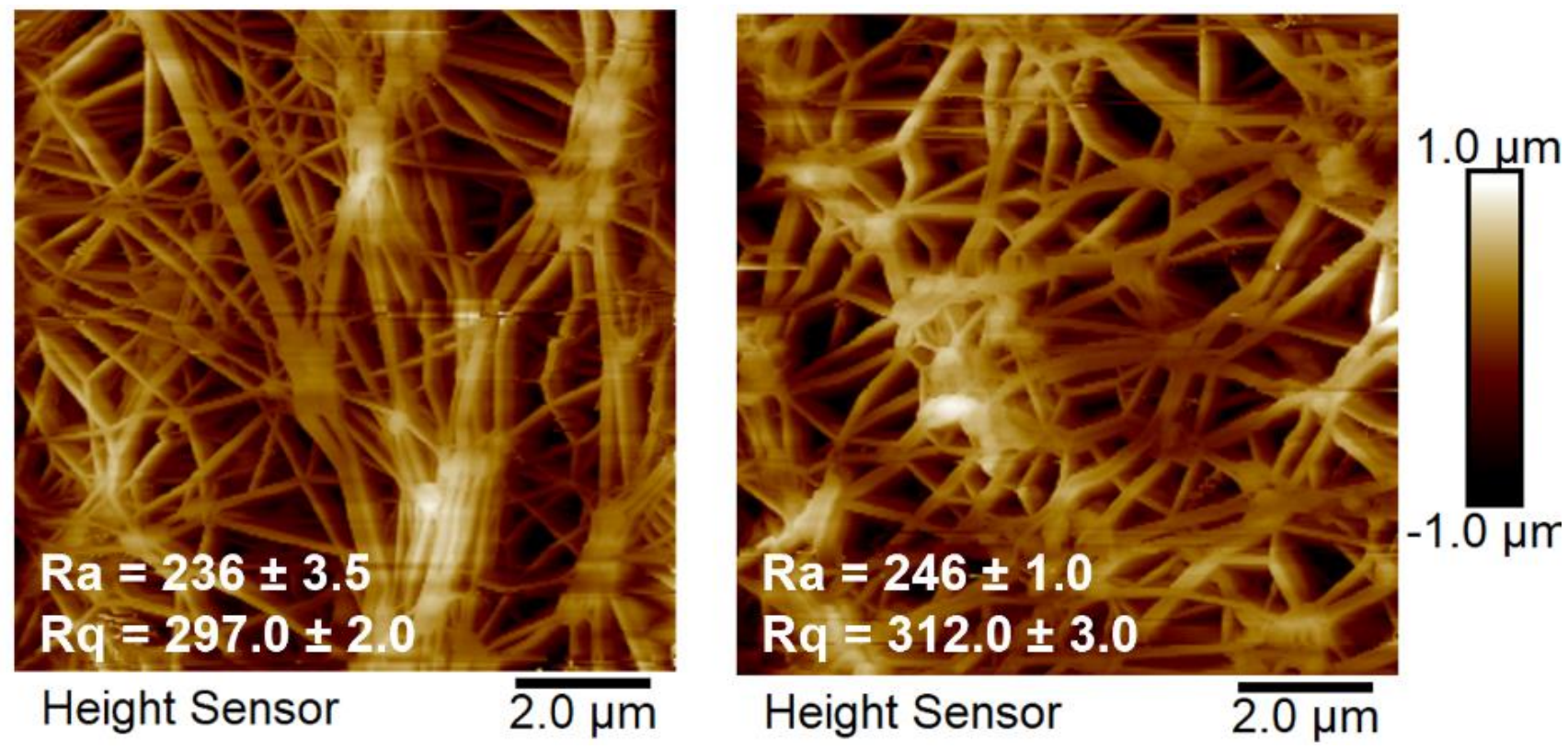

Fig. 3g AFM showing the surface morphology and roughness of a pristine PTFE membrane and the cleaned PTFE membrane after 500-h operation.

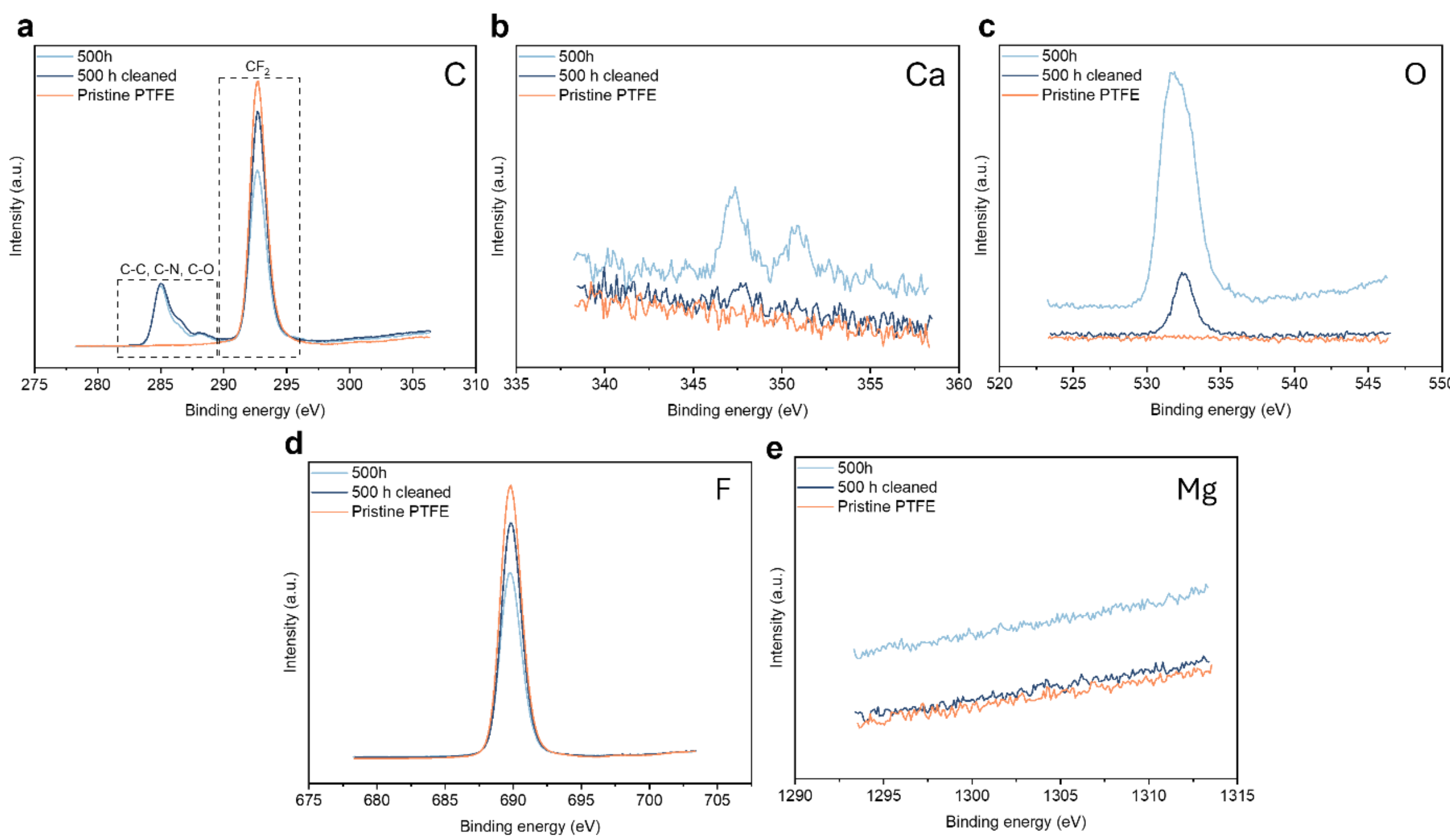

Supplementary Fig.15 High-resolution XPS spectrum of the pristine PTFE membrane, the PTFE membrane after 500-h operation without cleaning, and the cleaned PTFE membrane after the 500-h operation, showing the regions for **a** carbon (C); **b** calcium (Ca); **c** oxygen (O), **d** fluorine (F); and **e** magnesium (Mg).

Supplementary Table 15. EDX showing surface elemental composition of the pristine PTFE membrane and the PTFE membrane after 500 h of operation before and after cleaning.

PTFE membrane	Element	Wt%	Wt% Sigma	Atom (%)
Pristine	C	26.55	0.72	36.37

	F	73.45	0.72	63.63
	Others*	0	0	0
	F/C ratio	2.77	–	1.75
After 500 hours without cleaning	C	28.54	0.97	38.58
	F	69.13	0.97	59.09
	Others*	2.33	–	2.32
	F/C ratio	2.42	–	1.53
After 500 hours cleaned	C	27.28	0.35	37.24
	F	72.71	0.35	62.75
	Others*	0	0	0
	F/C ratio	2.67	–	1.69

*Others include Ca, Mg, S, and O.

We have integrated a summary of these key results into the **newly revised Manuscript**:

Lines 311–336: "The OMD demonstrated a stable water flux of 19.05 ± 0.41 LMH for the first 300 h before gradually declining to 17.79 ± 0.42 LMH by the end of the test (Fig. 3c). This minor decline is due to the initial fouling formation as suggested by scanning electron microscopy (SEM) characterisation of the of the uncleaned membrane surface after 500-h use (Fig. 3e). Energy-dispersive X-ray spectroscopy (EDX) identified this foulant layer as a mixture of inorganic scale (Ca, Mg, S, and O at 2.32 atom%) and carbonaceous deposits, evidenced by a decrease in the surface F/C atomic ratio from 1.75 to 1.53 (Supplementary Fig. 14 and Table 15). Given that the 500-h test utilised raw, biologically active seawater, these carbonaceous deposits might include marine microorganisms and their hydrophilic extracellular polymeric substances (EPS). Importantly, this mixed fouling proved highly reversible after EDTA-based cleaning. Post-cleaning EDX showed that the inorganic scaling elements dropped to 0 atom% and the F/C ratio recovered to 1.69, indicating the removal of all mineral scale and most carbonaceous deposits. X-ray photoelectron spectroscopy (XPS) confirmed this reversibility, showing significantly diminished Ca, Mg, C, and O peaks after cleaning (Supplementary Fig. 15). To remove residual organic foulants and prevent long-term biofilm accumulation, widely used high-pH alkaline cleaners could be used in addition to EDTA cleaning. Incorporating antiscalant chemicals into the feed or implementing basic prefiltration (e.g., sand filters) can also reduce scaling risk and extend cleaning intervals[37]. Beyond reversible fouling, the PTFE membrane exhibited exceptional thermomechanical and chemical stability. SEM and ImageJ analysis of the cleaned membrane revealed an intact node-and-fibril microstructure with no pore collapse or widening (Fig. 3e, Supplementary Fig. 16). X-ray diffraction (XRD) and atomic force microscopy (AFM) confirmed that bulk crystallinity and nanoscale roughness remained virtually unchanged (Figs. 3f–g), ruling out any polymer etching or degradation caused by microbial metabolism. XPS further verified chemical integrity, showing perfectly matched carbon–fluorine and elemental fluorine spectra with no evidence of defluorination or chlorine incorporation."

The methods of those characterisations are also updated in **Lines 775–783**: "Pore size distribution was analysed from SEM images using ImageJ software[45]. Membrane surface topography was further investigated by AFM (Dimension Nexus, Bruker). Bulk crystalline phases were identified by powder XRD (X'Pert Pro, Panalytical), while surface chemical composition was evaluated by XPS (Axis Supra+, Kratos Analytical Limited). The crystallinity of the PTFE membranes before and after the 500-h test was calculated using the Hermans-Weidinger method, as shown in Eq. 15[46].

$$X_c = \frac{I_c}{I_c + 1.8 I_a} \times 100\% \quad (15)$$

where X_c is the crystallinity of PTFE, I_c is the integral intensity of the crystalline area, and I_a is the integral intensity of the amorphous area."

Q3: The excellent ion rejection data confirms that the OMD process successfully isolates the electrolyser from bulk chloride and bromide. However, have you considered or detected the potential for volatile chlorine species (e.g., Cl_2, HOCl) to form at the anode and subsequently permeate back through the PTFE membrane as gases? Such reverse transport could potentially re-contaminate the feed side and, more critically, induce chemical degradation of the PTFE membrane itself upon long-term exposure. Please comment on this potential risk and any relevant experimental observations. These references (J. Am. Chem. Soc. 2024, 146, 8, 5324–5332. and Nat. Commun., 2023, 14, 4822.) can be referred to.

(1) Given the system's closed-loop nature, did your gas chromatography analysis of the produced oxygen stream detect any trace amounts of chlorine gas (Cl_2) or other volatile chlorinated species?

(2) Following the long-term test, was NMR analysis performed on the PTFE membrane to confirm whether any chemical degradation, such as defluorination or structural rearrangement, occurred? Such evidence would be crucial to verify the occurrence of chemical attack under operational conditions.

Reply:

We sincerely thank the reviewer for this thoughtful question and for directing us to the relevant references. The potential for parasitic chlorine evolution and subsequent reverse transport is indeed a critical consideration for any system interfacing seawater with electrochemistry. We have addressed the concerns through both thermodynamic analysis and rigorous empirical validation.

(1) Detection of chlorine species in the oxygen stream and electrolyte

The reviewer raises a valid concern regarding the potential for chloride to oxidise at the anode, form volatile chlorine gas (Cl_2), and permeate back through the membrane. However, our system is designed with multiple thermodynamic and physical safeguards that preclude this pathway. The OMD process rejects >99% of bulk chloride, and the AWE anode operates in a highly purified,

concentrated 6 M KOH electrolyte, minimising the chloride reactant available for the Chlorine Evolution Reaction (CER). Even if trace chloride were to reach the anode, CER remains highly improbable. As supported by Pourbaix analysis, in strongly alkaline media (6 M KOH, pH >14), the thermodynamic and kinetic driving forces on NiFe-based catalysts overwhelmingly favor the OER. Furthermore, any hypothetical trace Cl_2 generated in such strong alkaline medium would rapidly disproportionate into chlorate (ClO_3^-) and transient chlorite (ClO_2^-), preventing it from existing as a volatile gas capable of reverse transport. To definitively rule out chlorine generation, we tested both the O_2 gas stream and the KOH solution during our 500-h test. Gas chromatography (GC) confirmed that the anodic O_2 stream maintained >99% purity (Fig. 3c). To test for trace volatiles, we passed the O_2 stream through a pH 10 NaOH scrubber. Any trace Cl_2 gas would be immediately hydrolysed to hypochlorite (OCl^-). Subsequent analysis using the DPD colorimetric method yielded no detectable chlorine species. We analysed the KOH solution using ion chromatography (IC). The results confirmed the complete absence of both ClO_3^- and ClO_3^- (limits of detection: 0.001 mg L^{-1}), verifying that no parasitic chlorine chemistry occurred at the anode.

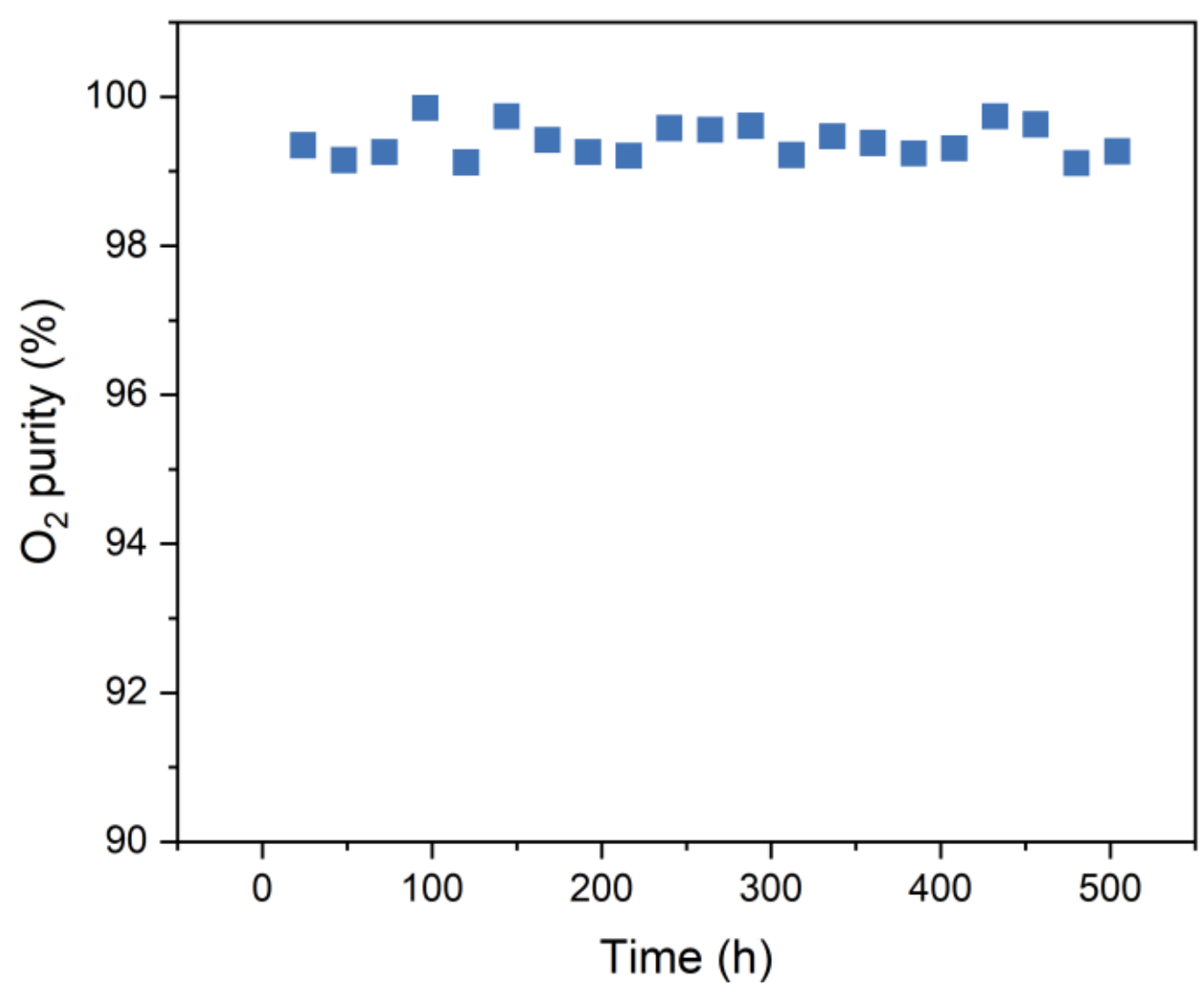

Fig. 3c O_2 purity during the 500-h continuous operation of the OMD-AWE.

(2) Assessment of PTFE membrane chemical degradation

Since no chlorine species are generated in the KOH loop, the permeate side of the PTFE membrane is not exposed to oxidative chemical attack. Nevertheless, to rigorously address the question regarding potential defluorination or structural rearrangement, we conducted comprehensive post-mortem characterisations after 500-h test. While NMR is a valuable technique for probing polymer structure, its application to bulk PTFE membranes is challenging due to the material's insolubility and high crystallinity, which often results in poorly resolved spectra. Instead, we employed highly sensitive surface and crystallographic techniques (SEM, EDX, XRD, and XPS), as detailed in our Reply to Q2. These analyses confirmed the absence of chlorine peaks, zero defluorination (with stable C–F spectra and F/C ratios), and an entirely intact bulk crystallinity and morphology. Finally, it is worth noting that the chemical inertness of PTFE is exceptionally high. Prior studies (Environ.

Sci. Technol. Lett. 2023, 10, 711–717 [2]) have demonstrated that PTFE membranes maintain their structural and desalination integrity even after prolonged exposure to aggressive free chlorine concentrations exceeding 3,000 mg/L. Therefore, the thermodynamic suppression of CER, the empirical absence of chlorine species in the system, and the confirmed chemical stability of the PTFE membrane demonstrate that the risk of degradation via reverse chlorine transport is negligible in our OMD-AWE system.

We have incorporated these discussions into the **newly revised Manuscript**:

Lines 304–336: "The electrochemical performance remained stable with cell voltage stabilised at 1.72 V under constant current of 1.13 A, Faradaic efficiency >99%, and O_2 purity >99% throughout the entire experiment. These results confirm the complete absence of parasitic chlorine evolution[34], which is suppressed because OMD rejected >99% salts (Fig. 3d), maintaining a highly alkaline 6 M KOH environment that thermodynamically favours oxygen evolution. The sustained >99% O_2 purity, even under low-current conditions, confirms no gas crossover[35], as the ZIRFON diaphragm prevents internal gas mixing and our degassing tower eliminates entrained microbubbles within the KOH loop. The OMD demonstrated a stable water flux of 19.05 ± 0.41 LMH for the first 300 h before gradually declining to 17.79 ± 0.42 LMH by the end of the test (Fig. 3c). This minor decline is due to the initial fouling formation as suggested by scanning electron microscopy (SEM) characterisation of the of the uncleaned membrane surface after 500-h use (Fig. 3e). Energy-dispersive X-ray spectroscopy (EDX) identified this foulant layer as a mixture of inorganic scale (Ca, Mg, S, and O at 2.32 atom%) and carbonaceous deposits, evidenced by a decrease in the surface F/C atomic ratio from 1.75 to 1.53 (Supplementary Fig. 14 and Table 15). Given that the 500-h test utilised raw, biologically active seawater, these carbonaceous deposits might include marine microorganisms and their hydrophilic extracellular polymeric substances (EPS). Importantly, this mixed fouling proved highly reversible after EDTA-based cleaning. Post-cleaning EDX showed that the inorganic scaling elements dropped to 0 atom% and the F/C ratio recovered to 1.69, indicating the removal of all mineral scale and most carbonaceous deposits. X-ray photoelectron spectroscopy (XPS) confirmed this reversibility, showing significantly diminished Ca, Mg, C, and O peaks after cleaning (Supplementary Fig. 15). To remove residual organic foulants and prevent long-term biofilm accumulation, widely used high-pH alkaline cleaners could be used in addition to EDTA cleaning[36]. Incorporating antiscalant chemicals into the feed or implementing basic prefiltration (e.g., sand filters) can also reduce scaling risk and extend cleaning intervals[37]. Beyond reversible fouling, the PTFE membrane exhibited exceptional thermomechanical and chemical stability. SEM and ImageJ analysis of the cleaned membrane revealed an intact node-and-fibril microstructure with no pore collapse or widening (Fig. 3e, Supplementary Fig. 16). X-ray diffraction (XRD) and atomic force microscopy (AFM) confirmed that bulk crystallinity and nanoscale roughness remained virtually unchanged (Figs. 3f–g), ruling out any polymer etching or degradation caused by microbial metabolism. XPS further verified chemical integrity, showing perfectly matched carbon–fluorine and elemental fluorine spectra with no evidence of defluorination or chlorine incorporation."

References:
1. Shen, W., Zheng, Y., Hu, Y., Jin, J., Hou, Y., Zhang, N., An, L., Xi, P. & Yan, C.-H. Rare-Earth-Modified NIS2 improves OH coverage for an industrial alkaline water electrolyzer. *J. Am. Chem. Soc.* **146**, 5324–5332 (2024). https://pubs.acs.org/doi/abs/10.1021/jacs.3c11861
2. Nguyen, D. T., Lopez, K. P., Lee, S., Lee, J., Hernandez, M. T. & Straub, A. P. Water Desalination via Pressure-Driven Distillation with Chlorine-Resistant and Large-Area Polymeric Membranes. *Environ. Sci. Technol. Lett.* **10**, 711–717 (2023).

Q4: The manuscript highlights the system's high hydrogen production flux. To better position this OMD-AWE technology within the existing landscape, could the authors provide a comparative analysis with state-of-the-art direct kW-scale seawater electrolyzers? A discussion focusing on key metrics such as system complexity, stability under real seawater feedstock, and the projected balance-of-plant requirements would be highly informative for assessing its practical competitiveness. These references (J. Am. Chem. Soc. 2025, 147, 20, 17190–17200. and Nat. Commun. 2024, 15, 10351.) can be referred to.

Reply:

We sincerely thank the reviewer for this suggestion and for directing us to these outstanding recent works (J. Am. Chem. Soc. 2025, 147, 17190; Nat. Commun. 2024, 15, 10351). Discussing these state-of-the-art kW-scale alkaline seawater electrolysers (ASWE) allows us to clearly position our OMD-AWE technology within the evolving landscape of seawater electrolysis. Fundamentally, the ASWE system and our OMD-AWE system represent two parallel, complementary paradigms for solving the seawater splitting challenge, i.e., the catalyst-centric paradigm and the isolationist paradigm. Below is a comparative analysis based on the requested metrics:

1. System complexity and stability

The impressive performance of ASWE was achieved by engineering sophisticated anode materials that are intrinsically resistant to chloride corrosion. While they demonstrate excellent stability for hundreds to thousands of hours, system complexity is heavily concentrated in the synthesis and scaling of these novel catalysts. Furthermore, their ultimate industrial viability hinges on these tailored catalysts surviving multi-year lifetimes (>10,000 h) under the fluctuating, harsh matrix of real seawater. Instead of forcing the electrolyser to resist seawater, our system completely isolates it from direct contact with seawater. This allows us to use standard, industrially mature AWE stacks with robust, low-cost Ni-based electrodes, whose multi-year longevity exceeding 80,000 hours is already well-established in industrial applications. For our system, stability is governed by the manageable, reversible fouling of the PTFE membrane (as demonstrated in our 500-h and 20-cycle tests), ensuring the electrolyser itself is permanently immune to chloride corrosion and electrode scaling. The complexity shifts to the thermal integration of OMD, leveraging mature technologies like hydrophobic PTFE membranes and heat exchangers.

2. Balance-of-Plant (BoP) requirements

ASWE operating in seawater typically requires dedicated chemical pretreatment, such as alkaline precipitation to remove scaling ions like Ca^{2+} and Mg^{2+}, to protect the electrodes. Additionally, it generates Joule heating, necessitating active cooling systems to prevent catalyst degradation. Our system achieves profound BoP simplification through thermal symbiosis. The OMD membrane rejects >99% of divalent scaling ions, eliminating the need for complex chemical pretreatment. AWE's waste heat, traditionally an operational burden requiring active chillers, is repurposed as the primary thermal driver for OMD. In return, OMD provides passive cooling to the AWE stack. This closed thermal loop eliminates both external heaters and active coolers, drastically lowering BoP energy consumption.

3. Practical competitiveness

Both pathways offer compelling economics. The referenced ASWE works report levelised costs of hydrogen (LCOH) of 1.96–2.82 USD kg^{-1} under favourable conditions (e.g., low electricity costs of ~0.02 USD kWh^{-1}). Our analysis projects a competitive LCOH range of 1.65–2.67 USD kg^{-1} for electricity costs of 0.02–0.04 USD kWh^{-1}. This cost-competitiveness stems from the use of low-cost, established AWE stacks, reduced BoP complexity, and the inherent affordability of the OMD process. Furthermore, our modular OMD-AWE architecture is completely agnostic to the electrolyser type, allowing it to seamlessly integrate future high-current-density commercial stacks as they evolve (details see Q9).

4. Synergy between the two paradigms

It is worth noting that the catalyst-centric paradigm and the isolationist paradigm are not mutually exclusive; rather, future systems could profoundly benefit from their synergy. For example, a ASWE system with advanced, chloride-resistant catalysts could achieve even greater durability and efficiency when paired with partial pre-purification (such as OMD). This would significantly reduce the contaminant burden on the anode, extending operational lifetimes while maintaining overall system simplicity.

In summary, while the referenced ASWE technologies represent brilliant advances in catalyst science, our OMD-AWE offers a parallel, system-engineering solution that circumvents the core material stability challenge. Its competitiveness lies in leveraging proven, low-cost industrial electrolyser hardware, ensuring inherent long-term stability via complete contaminant isolation, and simplifying BoP through innovative thermal integration. This makes OMD-AWE a compelling and de-risked pathway for applications where operational robustness, ease of maintenance, and compatibility with existing supply chains are paramount. We will incorporate this comparative discussion into the revised manuscript.

These insights are added to our **newly revised manuscript:**

Lines 613–631: "At current electricity prices ($0.1 kWh-1), it shows the lowest LCOH of $5.73 kg(H2)-1, remaining 6.3%, 12.2%, 17.8%, and 52.8% lower than RO+IEX-AWE, FOWSAWE, SDE-DSE, and DSE, respectively (Fig. 5b). At more favourable electricity costs used in other studies ($0.02–0.04 kWh^{-1}), our LCOH drops to $1.65–2.67 $kg(H_2)^{-1}$. The cost difference

compared to DSE highlights the critical importance of durability and system complexity. Traditional DSE suffers from prohibitively high LCOH due to expensive noble metal catalysts and shortened lifetimes. State-of-the-art kW-scale direct seawater electrolysers have shifted toward a catalyst-centric paradigm, engineering specialised materials to resist chloride attack, demonstrating stability for hundreds to thousands of hours. Their industrial viability depends on surviving multi-year lifetimes (>10,000 h) under the fluctuating, harsh matrix of real seawater, while still requiring chemical pretreatment and energy-intensive cooling. Our OMD-AWE offers a complementary isolationist paradigm that isolates electrolyser from seawater, decoupling stability from catalyst design. This enables use of mature, low-cost AWE components with established industrial longevities (>80,000 h) while eliminating active cooling and pretreatment. More importantly, these catalyst-centric and isolationist paradigms are not mutually exclusive; future systems could profoundly benefit from their synergy, by pairing advanced catalysts with membrane pre-purification to further reduce contaminant burdens and maximise industrial lifespans."

References:

1. Shen, W., Ye, Y., Hu, Y., Wu, H., Xia, Q., Xie, H., Li, Z., Zhang, N., An, L., Si, R., Xi, P. & Yan, C.-H. Corrosion protection of rare Earth for Kilowatt-Level alkaline seawater electrolyzer. *J. Am. Chem. Soc.* **147,** 17190–17200 (2025).https://doi.org/10.1021/jacs.5c02876

2. Sun, X., Shen, W., Liu, H., Xi, P., Jaroniec, M., Zheng, Y. & Qiao, S.-Z. Corrosion-resistant NiFe anode towards kilowatt-scale alkaline seawater electrolysis. *Nature Communications* **15,** 10351 (2024). https://www.nature.com/articles/s41467-024-54754-5

Q5: Safety is a paramount concern for electrolysis systems. The integrated system produces hydrogen and oxygen streams simultaneously. Could the authors please comment on the potential risk of hydrogen crossover into the oxygen stream? Specifically, have you measured the hydrogen-in-oxygen concentration, and does it remain well below the lower explosion limit (LEL) of 4% throughout the 500-hour operation? This is critical for assessing the system's operational safety and the adequacy of the gas separation design. This reference (Nat. Rev. Clean Technol. 2025, 1, 380–395.) can be referred to.

(1) Could you provide the actual gas chromatography data for both the hydrogen and oxygen streams, specifying the measured concentration of H_2 in O_2 and O_2 in H_2?

(2) Beyond the diaphragm in the AWE stack, does the PTFE membrane in the OMD module pose any risk of gas crossover between the seawater and KOH loops? Please evaluated its permeability to H_2 or O_2 gases under operational conditions

(3) The gravity-based degas tower is mentioned as the key gas-liquid separation unit. Could you elaborate on its design (e.g., dimensions, internals) and provide evidence of its separation efficacy? Specifically, how does it ensure that entrained electrolyte droplets containing dissolved H_2 or O_2 do not lead to gradual gas crossover in the recirculating loops?

Reply:

We fully agree that safety is the paramount concern. We have addressed these concerns regarding gas purity, potential gas crossover risks, and separator engineering as follows:

(1) We performed GC analysis on the collected O_2 and H_2 during the OMD-AWE operation. The results confirm that the purity of both exceeds 99% as shown in the below Supplementary Figure. As a result, the impurity concentrations (H_2 in O_2 and O_2 in H_2) are consistently below 1%. This value is much lower than LEL of 4%, confirming that the system operates with a significant safety margin. It is crucial to interpret these safety margins in the context of our operational current density. Our electrolyser was operated at 79 mA/cm^2 (constrained by the 1 cm^2 membrane unit in this lab setup). Although this is lower than common AWE values, it represents a more rigorous test for gas purity. As mentioned in Nat. Rev. Clean Technol. 2025, 1, 380–395, maintaining gas purity at low current densities is more challenging than at high currents. This is because the rate of gas evolution decreases with current, but the rate of diffusive gas crossover remains relatively constant, resulting in higher impurity levels during low-current operation. Therefore, the fact that our system maintained <1% H_2 crossover even at this low current density of 89 mA/cm^2, a range comparable to the emerging Decoupled Water Electrolysis (DWE) system [1]. If the system is safe against crossover at 79 mA/cm^2, it implies even higher safety margins would be achieved at higher current densities.

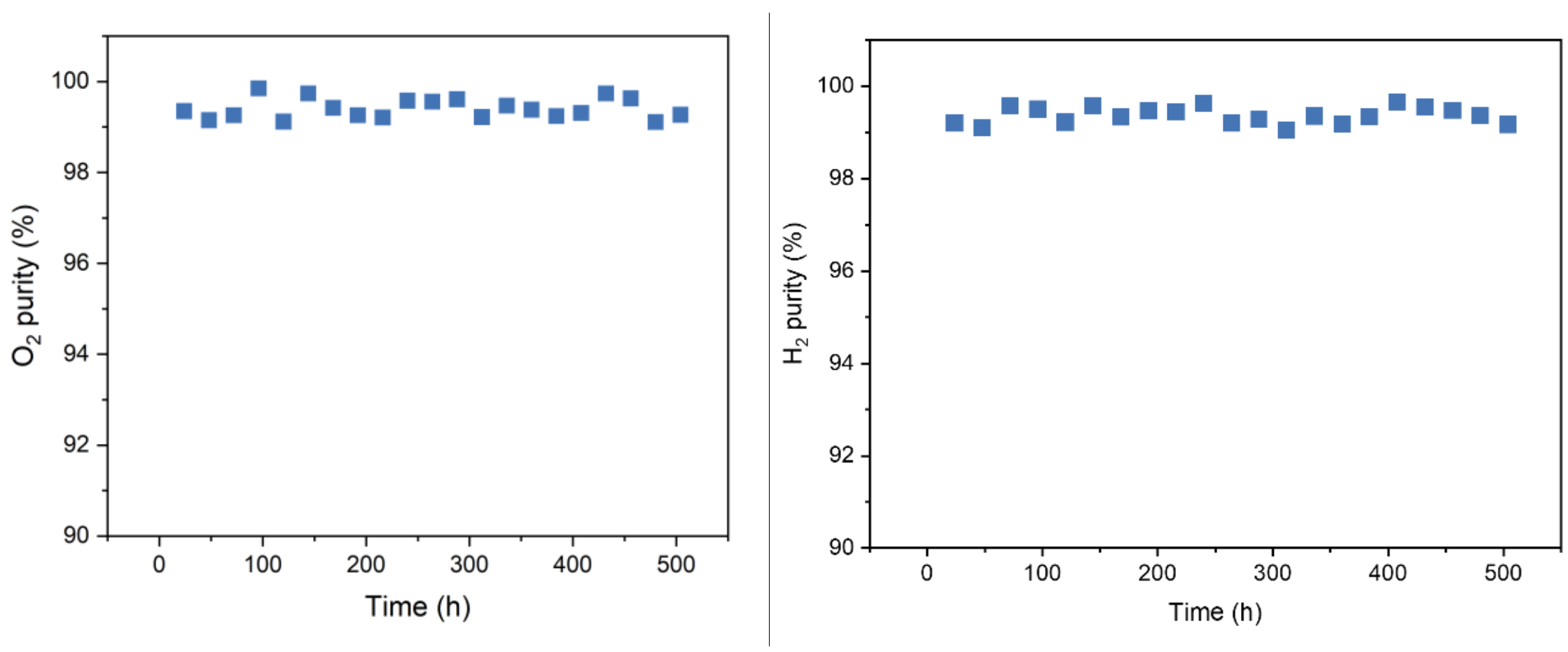

Supplementary Figs. O_2 and H_2 purity during the 500-h continuous operation of OMD-AWE.

(2) We evaluated the permeability of O_2 and H_2 through the PTFE membranes under operational conditions and confirmed that the risk of gas crossover is negligible due to solubility limits. The hydrophobic PTFE membrane acts as a strict barrier to the liquid phase, which prevents the direct transport of dissolved species; therefore, only dissolved gases released from seawater via thermal degassing could lead to cross-contamination. To evaluate this risk, we considered a worst-case scenario by first assuming the feed seawater is saturated with O_2 and H_2. Through heating, the seawater temperature is increased from 20°C to 40°C which reduces O_2 solubility from 7.4 to 5.4 mg/L, resulting in 2.0 mg/L of gaseous O_2 release to the OMD chamber before water production.

At the maximum feed flowrate of 0.334 kg/h (Fig. 4e), theoretical O_2 release reaches 0.7 mg/h. Even under the most conservative assumption of 100% O_2 permeation through the membrane, the O_2 impurity in the H_2 stream would be only 0.28% (calculated from 0.7 mg/h O_2 versus 250.8 mg/h H_2 production rate for per cm^2 of membrane). This impurity level remains well below the LEL of 4%. The actual O_2 permeation is even significantly lower as the majority of released O_2 exits from the OMD outlet, providing additional safety margins. Furthermore, the concentration of dissolved H_2 in natural seawater is negligible, $<10^{-6}$ mg/L. This extremely low concentration eliminates any significant driving force for H_2 permeation from seawater into the KOH loop. These quantitative analyses demonstrate the robust safety features of our PTFE membrane system against potential gas crossover. Our gas purity measurements confirmed both O_2 and H_2 product streams maintain purities above 99%, validating our analysis.

(3) To ensure high separation efficiency and prevent entrained gas bubbles from recirculating, we utilise a packed-bed degassing tower (detailed design shown in Supplementary Fig. 13) rather than a simple gravity tank. It employs a direct contact approach using high-surface-area Polypropylene (PP) structural packing. As the gas-rich KOH stream from the electrolyser enters the degassing tower, it flows over the packing material to create a thin liquid film; this drastically increases the gas-liquid interfacial area and reduces the diffusion path length, allowing even dissolved gases and microbubbles to release effectively into the headspace. The degassed liquid trickles down the packing path and collects at the bottom reservoir. The liquid level in the reservoir is maintained at a sufficient height to ensure that only fully degassed electrolyte trickles down to the return line. Therefore, the KOH returning to the stack remains free of entrained gas, preventing gas crossover. The 500-h stability of gas purity values directly confirms that our gas-liquid separation is efficient and that there is no progressive accumulation of impurities due to recirculation or crossover.

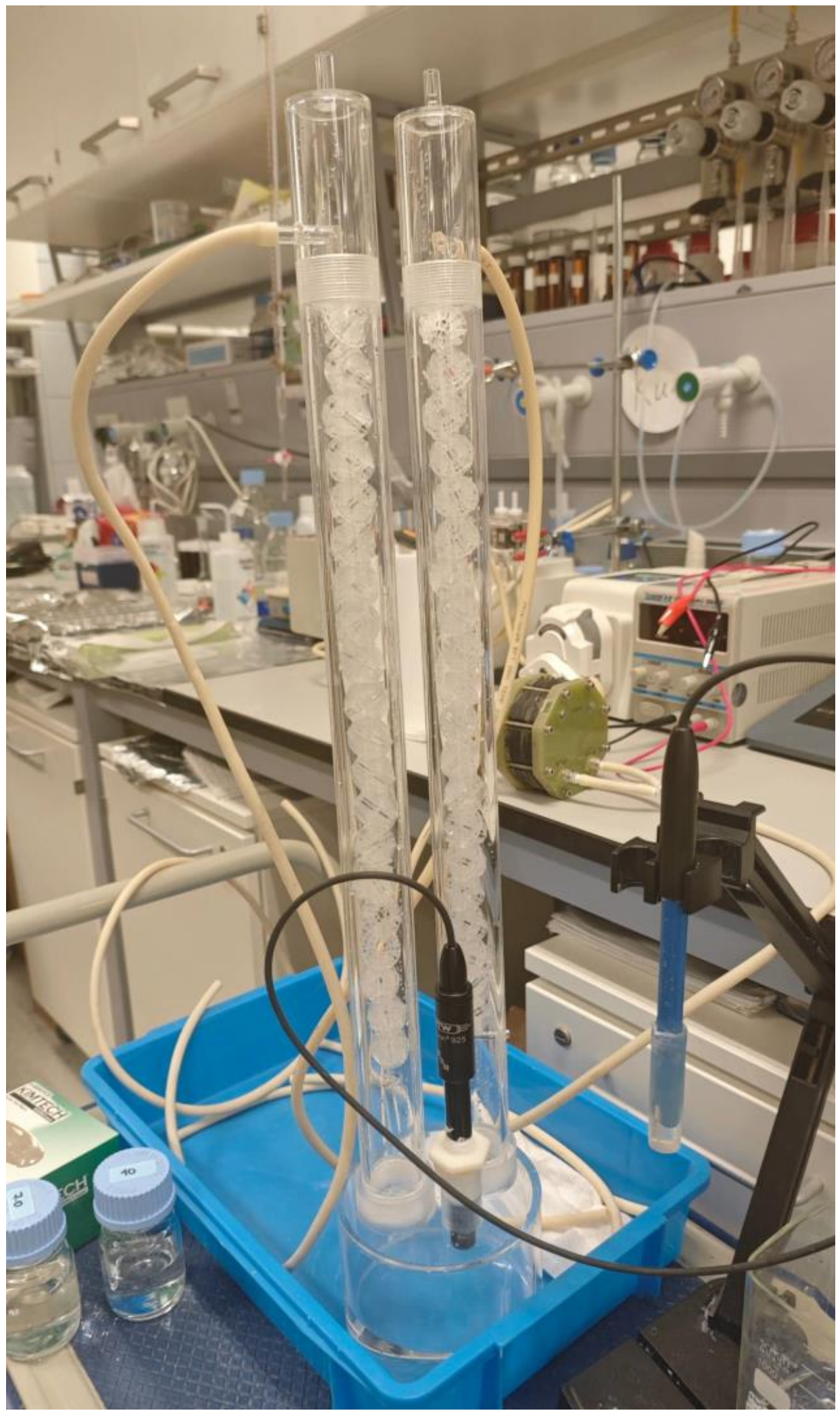

Supplementary Fig. 13 A packed-bed degassing tower used for H_2 and O_2 separation from KOH.

These are added to the **newly revised Manuscript**:

Lines 309–311: "The sustained >99% O_2 purity, even under low-current conditions, confirms no gas crossover, as the ZIRFON diaphragm prevents internal H_2/O_2 mixing and our degassing tower eliminates entrained microbubbles within the KOH loop."

The gas purity results are integrated into the **Fig 3c**, while the degassing tower design is provided in the Supplementary Fig. 13.

References:

1. Ruan, G., Todman, F., Yogev, G. *et al.* Technologies and prospects for decoupled and membraneless water electrolysis. *Nat. Rev. Clean Technol.* **1**, 380–395 (2025).

Q6: From a technology development perspective, could the authors please provide an outlook on the potential and challenges of integrating emerging hydrophobic materials (such as carbon nanotube membranes, graphene membranes, or organic-inorganic hybrid membranes) into the proposed system? Specifically, how might these advanced materials enhance water flux and antifouling performance, and what would be the key compatibility considerations (e.g., thermal stability under 40–60°C operation, chemical resistance to 6 M KOH, and mechanical integrity under flow conditions) for their practical implementation in this thermally symbiotic OMD-AWE system?

Reply:

We thank the reviewer for this insightful question regarding the future roadmap of membrane materials for the OMD-AWE system. While the commercial PTFE membranes used in this work provide a robust baseline, emerging membrane materials offer significant potential to break the current thermal trade-offs. The goals of developing emerging membrane materials involves improving membrane permeability for higher water flux and increasing hydrophobicity for stronger antifouling and anti-wetting ability. Unlike the tortuous pore pathways in conventional PTFE membranes, nanostructured materials like vertically aligned carbon nanotubes (CNTs) or laminar graphene oxides promote faster water vapor transport via low-friction atomic channels (known as slip-flow theory) [1]. This allows the system to achieve target water production rates at lower thermal gradients. Furthermore, integrating photothermal materials, e.g., Titanium Carbide MXene [2], enables localised interfacial heating under solar light, which amplifies the temperature gradient derived from AWE waste heat, widening the optimal operational window for thermal self-sufficiency. Regarding wetting and fouling resistance, emerging omniphobic architectures offer superior protection against complex seawater feeds [3]. They utilise specific re-entrant geometries such as Polystyrene nano-concave structures [4], hierarchical TiO_2 micro-flowers [5], or self-roughened nanofiber coatings [6], to sustain a metastable Cassie-Baxter state. These omniphobic architectures repel not just water but also low-surface-tension contaminants found in seawater (e.g., surfactants). Additionally, active wetting resistance can be achieved using electrically conductive CNT-composite membranes [1]. Applying a voltage to membranes induces electrostatic repulsion that further stabilises the liquid-vapor interface against pore intrusion

However, the practical implementation of these advanced materials in OMD-AWE faces unique compatibility challenges due to the 6 M KOH environment. Many materials reported in the literature, such as PVDF substrate [1] and silica nanoparticles [6], are unsuitable due to rapid dehydrofluorination and dissolution in hot alkaline conditions. While CNTs and graphene are chemically stable, the polymeric matrix binding them must also withstand hot KOH without swelling or delaminating. Therefore, future development must focus on alkali-stable alternatives, such as fluorinated matrices (PTFE) reinforced with chemically inert Titania or Zirconia nanoparticles [5], and ensuring that the interfacial bonding of these nanomaterials remains intact under long-term thermal and hydraulic stress.

These are added to the **newly revised Manuscript**:

Lines 449–452: "While PTFE membranes provide a robust baseline for OMD-AWE integration, emerging membrane materials offer opportunities for system upgrading, such as low-friction carbon nanotubes for faster vapor transport, photothermal coatings for improved thermal efficiency, and omniphobic surfaces for wetting resistance."

Reference:

1. Zhang, Y., Xing, J., Weci, G. et al. Advanced electrically conductive carbon nanotubes-PVDF composite membranes with electro-promoted water treatment performance. *Nat. Commun.* (2025). https://doi.org/10.1038/s41467-025-66260-3
2. Zhang, B., Wong, P.W., Guo, J. *et al.* Transforming $Ti_3C_2T_x$ MXene's intrinsic hydrophilicity into superhydrophobicity for efficient photothermal membrane desalination. *Nat Commun* **13**, 3315 (2022). https://doi.org/10.1038/s41467-022-31028-6
3. Lin, S., Nejati, S., Boo, C., Hu, Y., Osuji, C. O. & Elimelech, M. Omniphobic membrane for robust membrane distillation. *Environ. Sci. Technol. Lett.* **1**, 443–447 (2014). https://doi.org/10.1021/ez500267p
4. Guo, J., Jiang, M., Li, X. *et al.* Springtail-inspired omniphobic slippery membrane with nano-concave re-entrant structures for membrane distillation. *Nat Commun* **15**, 7750 (2024). https://doi.org/10.1038/s41467-024-52108-9
5. Aziz, M. H. A., et al. Enhanced omniphobicity of mullite hollow fiber membrane with organosilane-functionalized TiO_2 micro-flowers and nanorods layer deposition for desalination using direct contact membrane distillation. *J. Membr. Sci.* **607**, 118137 (2020). https://doi.org/10.1016/j.memsci.2020.118137
6. Deng, L., Ye, H., Li, X., Li, P., Zhang, J., Wang, X., Zhu, M. & Hsiao, B. S. Self-roughened omniphobic coatings on nanofibrous membrane for membrane distillation. *Sep. Purif. Technol.* **206**, 14–25 (2018). https://doi.org/10.1016/j.seppur.2018.05.035

Q7: The unique "self-dampening" operating environment, which provides a continuous supply of ultra-pure water, likely creates distinct catalyst working states compared to conventional electrolyzers. To uncover these differences, what in-situ/operando characterization techniques (e.g., in-situ Raman or X-ray absorption spectroscopy) could be employed to directly observe the dynamic surface structure and valence state of the NiFe anode and Ni cathode under actual operating conditions? Understanding this real-time dynamic process is crucial for custom-designing the most compatible and efficient catalysts specifically for this OMD-AWE system. These references (J. Am. Chem. Soc. 2025, 147, 44, 40807–40815. and Nat. Energy. 2025, 10, 880–889.) can be referred to.

Reply:

We thank the reviewer for this insightful suggestion. We have carefully studied the recommended references, particularly J. Am. Chem. Soc. 2025, 147, 40807–40815 [1], which demonstrates the power of in-situ techniques in revealing dynamic catalyst evolution. We agree that understanding real-time material changes is crucial for custom-designing the most compatible components for OMD-AWE. We would like to offer a different perspective regarding where these dynamic changes occur in our specific system.

Unlike direct seawater electrolysis (DSE), where the catalysts are directly exposed to a complex impurity matrix [2,3], our OMD-AWE physically separates the seawater from the electrodes using a PTFE membrane and thus catalysts operate in a "shielded" environment with pure and highly concentrated KOH. Therefore, the "distinct working state" of the catalyst in our system is actually a return to the standard state of conventional AWE. In this context, the value of employing in-situ techniques (e.g., operando Raman spectroscopy and X-ray photoelectron spectroscopy [1]) would be to experimentally validate this "shielding effect". For example, future studies could demonstrate that the NiFe anode surface in OMD-AWE maintains the characteristic spectral features of the active oxyhydroxide phase, in contrast to the chloride attack or carbonate precipitates observed in DSE. This would confirm that future catalyst design for OMD-AWE can focus on optimising intrinsic activity of standard AWE catalysts rather than engineering anti-corrosion properties.

We believe the reviewer's suggestion about in-situ monitoring is more critical for the membrane, as it operates at the interface where it experiences continuous interactions with seawater. Future in-situ monitoring could focus on membrane microstructural evolution using synchrotron-based X-ray scattering techniques. Wide-Angle X-ray Scattering (WAXS) can track real-time changes in polymer crystallinity and chain orientation, while Small-Angle X-ray Scattering (SAXS) can quantify pore sizes [4]. Additionally, Raman or Fourier Transform Infrared (FTIR) spectroscopy can monitor chemical changes under thermal and chemical stress by analysing vibrational spectra of functional groups in real time. These combined techniques enable early detection of structural changes before membrane failure occurs. Furthermore, in-situ light transmission imaging can track wetting propagation across the membrane surface, while Laser Induced Fluorescence can detect early pore breakthrough events [5,6]. Optical coherence tomography (OCT) enables to visualise real-time nucleation and growth of Mg/Ca scaling layers [7]. These in-situ monitoring techniques enable the custom-design of compatible materials for OMD-AWE, guiding the development of membrane materials with enhanced structural stability and anti-wetting properties needed for long-term operation.

These insights are added to the **newly revised Manuscript**:

Lines 453–458: "Future membrane development could prioritise engineering these functionalities with alkali-stable materials that withstand the hot KOH environment, guided by advanced in-situ/operando characterisations (e.g., Raman and X-ray scattering, optical visualisation techniques). These techniques will be crucial to verify the membrane's shielding effect on the catalyst's dynamic surface structure, while monitoring real-time membrane scaling and pore wetting under dynamic thermal and chemical stress."

Reference:

1. Shen, W., Peng, C., Xia, Q., Hu, Y., Sun, X., Ye, Y., Xie, H., Wu, S., Zhang, N., An, L., Zheng, Y., Sun, S., Xi, P. & Yan, C.H. Y-Doped CuS promotes selective electroreduction of CO_2 to ethanol. *J. Am. Chem. Soc.* **147**, 40807–40815 (2025). https://doi.org/10.1021/jacs.5c13850
2. Wang, R., Yang, Y., Guo, J. *et al.* Cathode catalyst layers modified with Brønsted acid oxides to improve proton exchange membrane electrolysers for impure water splitting. *Nat Energy* **10**, 880–889 (2025). https://doi.org/10.1038/s41560-025-01787-9
3. Guo, J., Zheng, Y., Hu, Z. *et al.* Direct seawater electrolysis by adjusting the local reaction environment of a catalyst. *Nat Energy* **8**, 264–272 (2023). https://doi.org/10.1038/s41560-023-01195-x
4. Tian, X., Jing, Z., Wang, J., Wei, Y., Tian, L., Wang, H. & Chang, N. Small and wide angle X-ray scattering to reveal nanoscale structure evolution of the polyamide RO membranes triggered by ethyl acetate. *J. Membr. Sci.* **693,** 122330 (2023). https://doi.org/10.1016/j.memsci.2023.122330
5. Jacob, P., Dejean, B., Laborie, S. & Cabassud, C. An optical in-situ tool for visualizing and understanding wetting dynamics in membrane distillation. *J. Membr. Sci.* **595,** 117587 (2020). https://doi.org/10.1016/j.memsci.2019.117587
6. Kiefer, F., Präbst, A., Rodewald, K. S. & Sattelmayer, T. Membrane scaling in Vacuum Membrane Distillation - Part 1: In-situ observation of crystal growth and membrane wetting. *J. Membr. Sci.* **590,** 117294 (2019). https://doi.org/10.1016/j.memsci.2019.117294
7. Bauer, A., Wagner, M., Saravia, F., Bartl, S., Hilgenfeldt, V. & Horn, H. In-situ monitoring and quantification of fouling development in membrane distillation by means of optical coherence tomography. *J. Membr. Sci.* **577,** 145–152 (2019). https://doi.org/10.1016/j.memsci.2019.02.006

Q8: The author mentioned that "Scaling behaviour was monitored over three consecutive cycles of 7 h each". For assessing industrial viability, this limited number of cycles is insufficient to evaluate long-term durability. It is critical to provide:

(1) It is imperative to provide data on water flux recovery rates across a substantially higher number of fouling-cleaning cycles (e.g., 10-20 cycles). Such an extended dataset is required to establish a definitive trend regarding the membrane's regenerative performance and long-term resistance to repeated scaling.

(2) A critical discussion is needed on whether the regeneration protocol, particularly with EDTA, induces any cumulative damage to the PTFE membrane's intrinsic properties.

Reply:

We sincerely thank the reviewer for this critical suggestion. To address your points, we performed an extended scaling test consisting of 20 consecutive cycles using a mixed feed solution at 40°C

containing 30 mM Na_2SO_4 and 30 mM $CaCl_2$ and an osmotic agent of 6 M KOH at 20°C. Each cycle involved 7 h of scaling followed by membrane cleaning through a 30-min flush with 0.05 M EDTA and a 10-min rinse with deionised water to assess regenerative capacity.

Over the 20-cycle scaling test, the PTFE membrane demonstrated minimal performance decline with exceptional regenerative capacity. The first 6 cycles showed complete flux recovery (i.e., the normalised water flux ratio ($J_w/J_{w,0}$) consistently recovering to 1.0 after cleaning), while even after 20 cycles under such harsh accelerated conditions, the membrane still maintained >95% of $J_w/J_{w,0}$ (Supplementary Fig. 6). SEM and EDX analysis confirmed the effectiveness of the EDTA-based cleaning. Large gypsum crystals were deposited on the membrane surface after 20 cycles but were removed after cleaning (Supplementary Fig. 7). EDX analysis showed an 84% reduction in the surface atomic concentrations of calcium (Ca), sulfur (S), and oxygen (O) which are key elemental markers of gypsum (Supplementary Fig. 8 and Table 13). The gradual reduction in flux recovery over the 20 cycles might be attributed to the residual 14% of these mineral phases. This behaviour is consistent with the PTFE membrane's small pore size, which provides excellent initial scaling resistance but may gradually retain microscopic crystal nuclei within the pore structures that are less accessible to cleaning. For future industrial deployment, this minor pore-entrapped scaling can be effectively mitigated through standard continuous antiscalant dosing or by heated EDTA cleaning. Maintaining >95% flux recovery after 20 accelerated cycles demonstrates robust resistance to irreversible fouling and confirms the membrane's suitability for long-term operation under normal scaling environments.

Regarding potential cumulative damage from the regeneration protocol, both theoretical analysis and empirical data confirm that EDTA cleaning does not compromise PTFE membrane properties. PTFE's exceptionally stable carbon-fluorine bonds make it inherently resistant to mild chelating agents like EDTA. Any cumulative degradation would decrease the membrane's liquid entry pressure (LEP), causing pore wetting, salt crossover, and varied water flux. However, our previous 500-h test with continuous EDTA dosing maintained highly stable water flux and near-perfect salt rejection. These results provide evidence that EDTA cleaning is safe and causes no cumulative damage to the membrane's integrity.

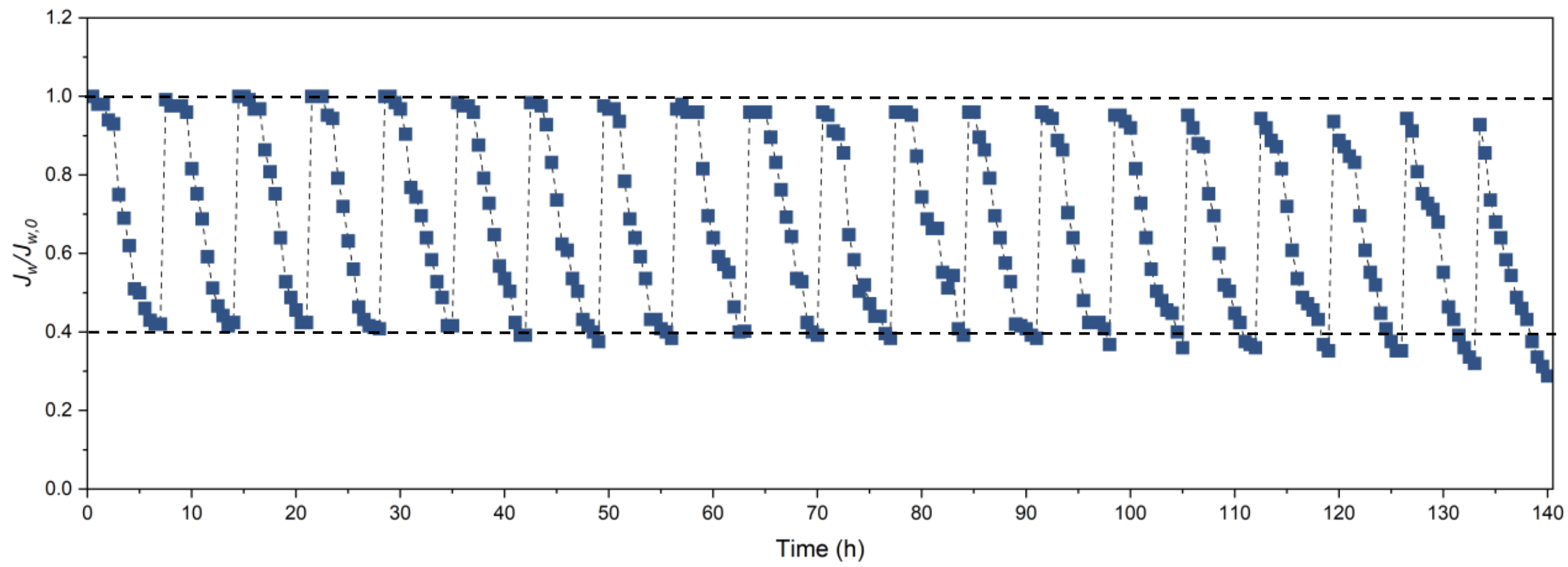

Supplementary Fig. 6 Accelerated scaling tests. $J_w/J_{w,0}$ over 20 cycles of 7 hours each using a feed containing 30 mM Na_2SO_4 and 30 mM $CaCl_2$ and 6 M KOH as osmotic agent. Conditions: T_{feed} = 40°C, T_{KOH} = 20°C.

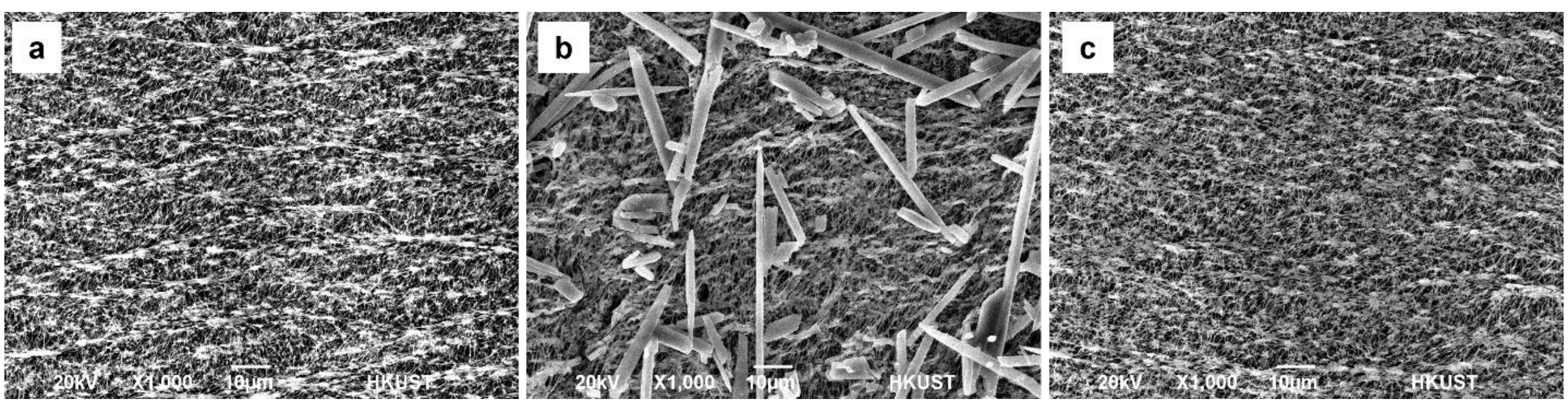

Supplementary Fig. 7 SEM images showing the surface morphology of **a** the pristine PTFE membrane; **b** the PTFE membrane after 20 cycles of accelerated scaling test (final cycle without cleaning); and **c** the PTFE membrane after cleaning following the 20th cycle. Conditions: [feed] = 30 mM Na_2SO_4 and 30 mM $CaCl_2$, [KOH] = 6.0 M, T_{feed} = 40°C, T_{KOH} = 20°C.

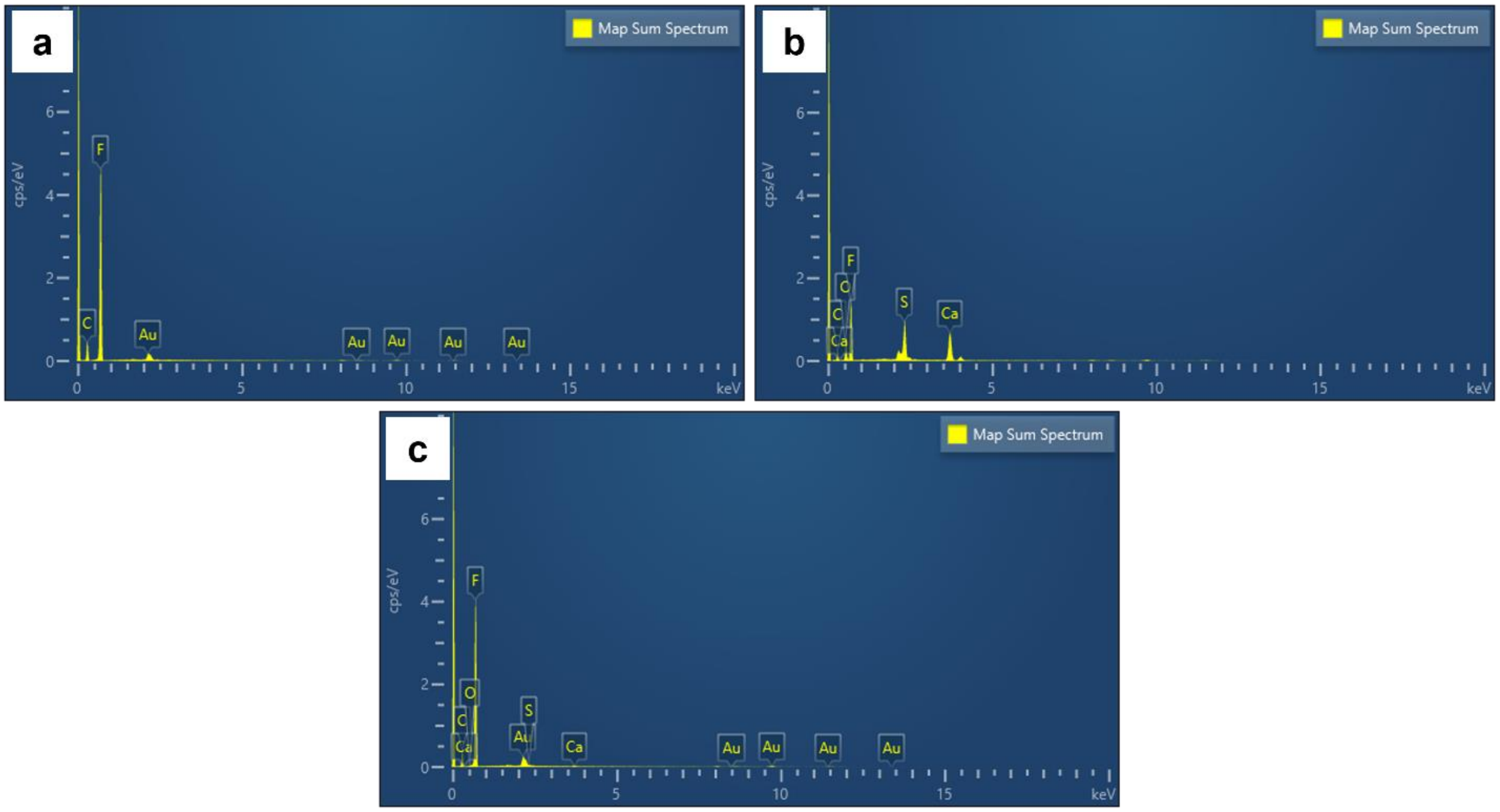

Supplementary Fig. 8 EDX spectrum showing the surface elemental composition of **a** the pristine PTFE membrane; **b** the PTFE membrane after 20 cycles of accelerated scaling test (final cycle without cleaning); and **c** the PTFE membrane after cleaning following the 20th cycle. Conditions: [feed] = 30 mM Na_2SO_4 and 30 mM $CaCl_2$, [KOH] = 6.0 M, T_{feed} = 40°C, T_{KOH} = 20°C.

Supplementary Table 13. Surface elemental composition analysis of the PTFE membrane during accelerated scaling tests using energy-dispersive X-ray spectroscopy (EDX).

PTFE membrane	Element	Wt%	Wt% Sigma	Atom (%)
Pristine	C	26.55	0.72	36.37
	F	73.45	0.72	63.63
	Others*	0	0	0
	F/C ratio	2.77	–	1.75
After 20 cycles (final cycle without cleaning)	C	18.88	0.92	28.25
	F	48.1	0.75	45.51
	Others*	33.02	–	26.23
	F/C ratio	2.55	–	1.61
After 20 cycles cleaned	C	25.36	0.84	35.55
	F	67.87	0.82	60.16
	Others*	6.77	–	4.30
	F/C ratio	2.68	–	1.69

*"Others" include Ca, S, and O.

These are added in our **newly revised Manuscript**:

Lines 214–217: "An extended 20-cycle test further confirmed this regenerability, maintaining near-complete flux recovery and demonstrating membrane scaling at 40°C is highly reversible (Supplementary 6–8 and Table 13). The chemical inertness of PTFE ensures that such EDTA treatments induce no cumulative damage over long-term operation."

The analysis of this 20-cycle scaling test is detailed in Supplementary Figs. 6–8 and Table 13.

Q9: The AWE utilized in this work, while robust, is typically limited to lower current densities compared to advanced AEM or PEM electrolyzers. Given that your OMD system demonstrates an exceptionally high water flux capable of supporting intense reactions, have you considered its integration with higher-current-density electrolyzers like AEM or PEM? What would be the key system-level adaptations required, particularly regarding thermal management (for PEM's lower operating temperature) and the chemical compatibility of the PTFE membrane with acidic (PEM) or low-alkalinity (AEM) environments? These references (Nat. Energy. 2024, 8, 264–272. and Nat. Catal. 2024, 7, 944–952.) can be referred to.

Reply:

We appreciate the reviewer's forward-looking question. Although our PTFE membrane system can integrate with AEM and PEM, these options are less favourable than AWE due to their lower water flux and reduced waste heat generation, as discussed below:

The PTFE membranes are chemically inert to both the acidic environments of PEM and the dilute alkaline/neutral environments of AEM. Unlike direct seawater electrolysis suffers from chloride attack and precipitate formation on catalysts [1], the membrane-integrated approach avoids these issues by ensuring only water vapor permeating to electrolysers. Therefore, it serves as a universal barrier against seawater impurities for AWE, AEM, and PEM, and no material adaptation is needed for the membrane itself.

However, integrating PEM and AEM would result in reduced water flux from PTFE membranes due to the lost osmotic driving force. The current AWE benefits from dual driving forces, i.e., the thermal gradient (ΔT) of 20°C and osmotic gradient from 6 M KOH, achieving water flux of ~22 LMH. AEM and PEM systems typically use dilute 1 M KOH or pure water, providing negligible osmotic contribution. With the thermal gradient alone, the system maintains a baseline flux of ~18 LMH (as shown in Fig. 2a, at 40°C feed). To integrate with AEM/PEM, the system must account for this 20% water flux reduction, which can be managed by slightly increasing the membrane areas. More importantly, since OMD relies on the waste heat from electrolysis to drive water vapor transport, the total thermal budget is the defining constraint for the system integration. To enable thermal self-circulation, the waste heat generated by electrolyser (Φ_{waste}) must exceed the latent heat required for OMD water vapor transport (Φ_{vapor}) (Eq. S6). Using 1 cm^2 membrane as an example. It produces 0.0022 kg h^{-1} of water at a flux of 22 LMH, while consuming the produced

water at a same rate requires 6.7 A total current for the electrolyser (i_{total}, distributed across 5 cells at 1.34 A each). Based on the thermoneutral voltage (U_{tn}) of 1.48 V, a minimum operating voltage (U) of 1.7 V is needed to generate sufficient Φ_{waste} for overcoming Φ_{vapor} of 1.47 W, calculated from the 0.0022 kg h^{-1} multiplied by latent heat of vaporisation (ΔH_v, 2400 kJ kg^{-1}).

$$\Phi_{waste} = i_{total} \cdot (U - 1.48) > \Phi_{vapour} \quad (S6)$$

Therefore, the inefficiency of AWE electrolyser is actually a thermal asset in the thermal symbiosis. The AWE typically operates at current densities of 0.2–0.5 A cm^{-2}, requiring voltages of 1.8–2.4 V (Supplementary Table below), which exceed the minimum threshold of 1.7 V. This generates sufficient waste heat of 2.1–6.2 W (versus Φ_{vapor} at 1.47 W), making the system easier to maintain the thermal gradient for OMD operation. However, high-efficiency PEM and AEM might operate below the 1.7 V threshold with lower thermal input, which would lead to reduced water flux and thus low Φ_{vapor}. While the thermal self-circulation may still be sustained, system performance would be compromised. To address this, these systems must operate at higher current densities, which raise the cell voltage via kinetic/ohmic overpotentials. For example, a PEM electrolyser operating at 1.5 A cm^{-2} might have a voltage of 1.6 V, increasing the current density to 3.0 A cm^{-2} (via a smaller electrode area) can increase the voltage to 2.0 V. Thus, PEM or AEM integration is feasible but needs careful evaluation of their specific constraints, particularly catalyst degradation, stack durability, and the overall cost of H_2 production, ensuring that the thermal benefits do not come at the expense of economic viability.

Supplementary Table. Different water electrolysis types and their working conditions.

Electrolysis type	Current density (A cm^{-2})	Cell Voltage (V)	Stack efficiency*
AWE	0.2–0.5	1.8–2.4	61.7–82.2%
AEM	1.0–2.0	1.8–2.2	67.3–82.2%
PEM	1.5–3.0	1.6–2.0	74.0–92.5%

*Stack efficiency is defined as the thermoneutral potential (U_{tn} = 1.48 V) over cell voltage.

To verify our hypothesis, we used our thermal-water-hydrogen model to evaluate the performance of PEM/AEM integrated with MD (MD-PEM/AEM) under the thermal self-circulation scenario, considering pure water as the electrolyte. Fig. 4f shows the H_2 production flux as a function of stack efficiency and voltage, bounded by two distinct operational limits. The top dashed line represents the H_2 flux (60.2 kg day^{-1} m^{-2}) by the OMD-AWE with external heating to maintain ΔT (20°C), while the bottom line represents the isothermal SDE-DSE (or OD-AWE) [2], which relies only on 6 M KOH osmotic driving force without thermal enhancement, yielding a H_2 flux of 2.42 kg m^{-2} day^{-1}. The curves between these bounds shows the H_2 flux with thermal self-circulation from AWE or PEM/AEM electrolyser's waste heat. As the cell voltage decreases (higher stack efficiency), less waste heat is generated, causing a corresponding decline in the H_2 flux for both systems. Across the voltage range, the thermally symbiotic OMD-AWE consistently outperforms those MD-PEM/AEM systems, confirming the additional osmotic driving force provided by the 6

M KOH used in AWE. In contrast, the PEM/AEM systems, utilising pure water, rely only on the thermal gradient, resulting in a lower H_2 flux and a faster decline as the thermal budget reduces. Mapping the typical operating conditions of different electrolysers onto the performance profiles reveals the operational advantages for AWE. The AWE operating voltage of 1.8–2.4 V corresponds to a H_2 flux of 30–45 kg day^{-1} m^{-2}, benefiting from both the thermal surplus and the osmotic boost from 6 M KOH. In contrast, the high-efficiency PEM/AEM with 1.6–2.2 V yields a lower flux of <35 kg day^{-1} m^{-2}. While PEM/AEM integration remains feasible, it requires larger membrane areas and operation at higher current densities to yield equivalent H_2 as AWE systems. This reveals an opportunity for mature AWE technology. Although AWE's lower electrical efficiency are viewed as drawbacks compared to PEM/AEM technologies, our thermally symbiotic architecture inverts this inefficiency into a powerful thermodynamic asset. Combined with AWE's low cost, high durability, and technological maturity, this makes it the optimal choice for membrane-integrated seawater electrolysis. In contrast, highly pursued PEM/AEM systems underperform in this integrated context, requiring higher current densities to match AWE's H_2 output.

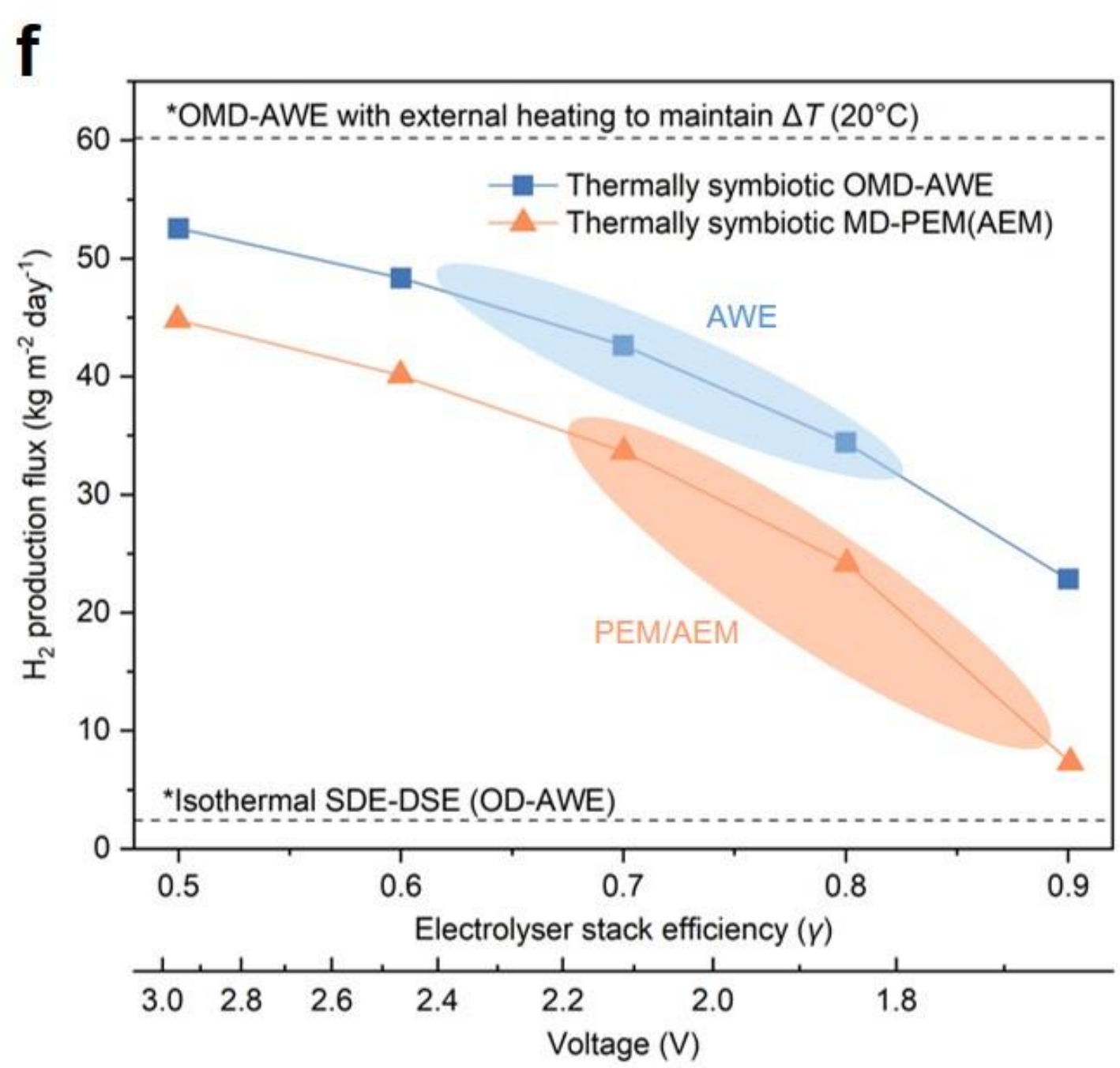

Fig. 4f The comparison in H_2 production flux between the thermally symbiotic OMD-AWE and MD-PEM/AEM systems as a function of electrolyser stack efficiency and cell voltage.

These discussions are added to the **newly revised Manuscript**:

Lines 503–518: "These findings suggest a significant opportunity for commercial AWE stacks (γ = 0.62–0.82). Within this real-world range, our system is thermally self-sustaining with H_2 fluxes of 30–45 kg m^{-2} day^{-1} (Fig. 4f) while co-producing 40% more clean water beyond electrolysis needs (Supplementary Figs. 24–25). Even under extreme high-efficiency scenario (γ = 0.9), OMD-AWE still achieves 5.6 times higher H_2 yields than the baseline SDE-DSE. Beyond AWE, this thermal

integration is adaptable to Proton Exchange Membrane (PEM) and Anion Exchange Membrane (AEM) electrolysis, as the chemically inert PTFE membrane is compatible with both acidic and dilute alkaline environments. However, MD-PEM and MD-AEM consistently underperform OMD-AWE due to the absent or less concentrated electrolyte for osmotic driving force. Typical PEM and AEM systems (γ = 0.74–0.93) yields less waste heat, resulting in lower H_2 fluxes (<35 kg m^{-2} day^{-1}). Although AWE's lower efficiency are viewed as drawbacks compared to PEM/AEM technologies, our thermally symbiotic architecture inverts this inefficiency into a powerful thermodynamic asset. Combined with AWE's low cost, high durability, and technological maturity, this makes it the optimal choice. In contrast, highly pursued PEM/AEM systems underperform in this integrated context, requiring higher current densities to yield equivalent H_2 as AWE systems. "

Reference:

1. Guo, J., Zheng, Y., Hu, Z. *et al.* Direct seawater electrolysis by adjusting the local reaction environment of a catalyst. *Nat Energy* **8**, 264–272 (2023). https://doi.org/10.1038/s41560-023-01195-x.
2. Xie, H., Zhao, Z., Liu, T., Wu, Y., Lan, C., Jiang, W., Zhu, L., Wang, Y., Yang, D. & Shao, Z. A membrane-based seawater electrolyser for hydrogen generation. *Nature* **612**, 673–678 (2022). https://doi.org/10.1038/s41586-022-05379-5

Q10: The author mentioned that "The manuscript clearly states that EDTA was dosed (0.5% w/v) into the seawater feed during the 500-h longevity test". The use of EDTA in the real seawater experiments, while practical for demonstrating long-term operation, potentially masks the inherent anti-fouling performance of the PTFE membrane and the true fouling challenges in natural environments. To accurately assess the system's robustness, the following clarifications are essential:

(1) Please clarify the duration for which the OMD-AWE system can operate stably using real seawater without the addition of any antiscalants or cleaning agents. This would establish a baseline for the membrane's intrinsic fouling resistance and inform the required frequency for maintenance cleaning in a practical scenario.

(2) The fouling analysis primarily identified inorganic scaling. However, real seawater contains a complex mixture of natural organic matter (NOM), such as humic acids, and inorganic salts. Was there any evidence of synergistic fouling, where NOM and inorganic ions (e.g., Ca^{2+}, Mg^{2+}) co-precipitate or form a mixed organic-inorganic foulant layer on the membrane surface that is more resistant to cleaning?

Reply:

We sincerely thank the reviewer for raising these critical points, which allow us to provide a more comprehensive assessment of our system's robustness. We fully agree that adding an antiscalant

masks the membrane's inherent anti-fouling properties, so we conducted a new 500-h test without the addition of EDTA or any other antiscalants and a comparison test regarding NOM.

(1) Our initial use of EDTA followed standard industrial practices to ensure operational longevity and minimise maintenance frequency. To establish the membrane's intrinsic fouling resistance, we conducted a new 500-h continuous tests using 5 µm filtered raw seawater without any antiscalants or cleaning agents. The results show that our OMD-AWE system still maintained stable operation for the entire 500 h duration without chemical additives (Fig. 3c). This establishes a robust baseline, proving that the membrane's hydrophobicity effectively manages the scaling propensity of real seawater over a highly practical maintenance interval. Although fouling and scaling accumulated during operation, post-mortem analyses confirmed complete reversibility through EDTA cleaning, while PTFE membrane itself also retained its structural integrity (detailed in Q2).

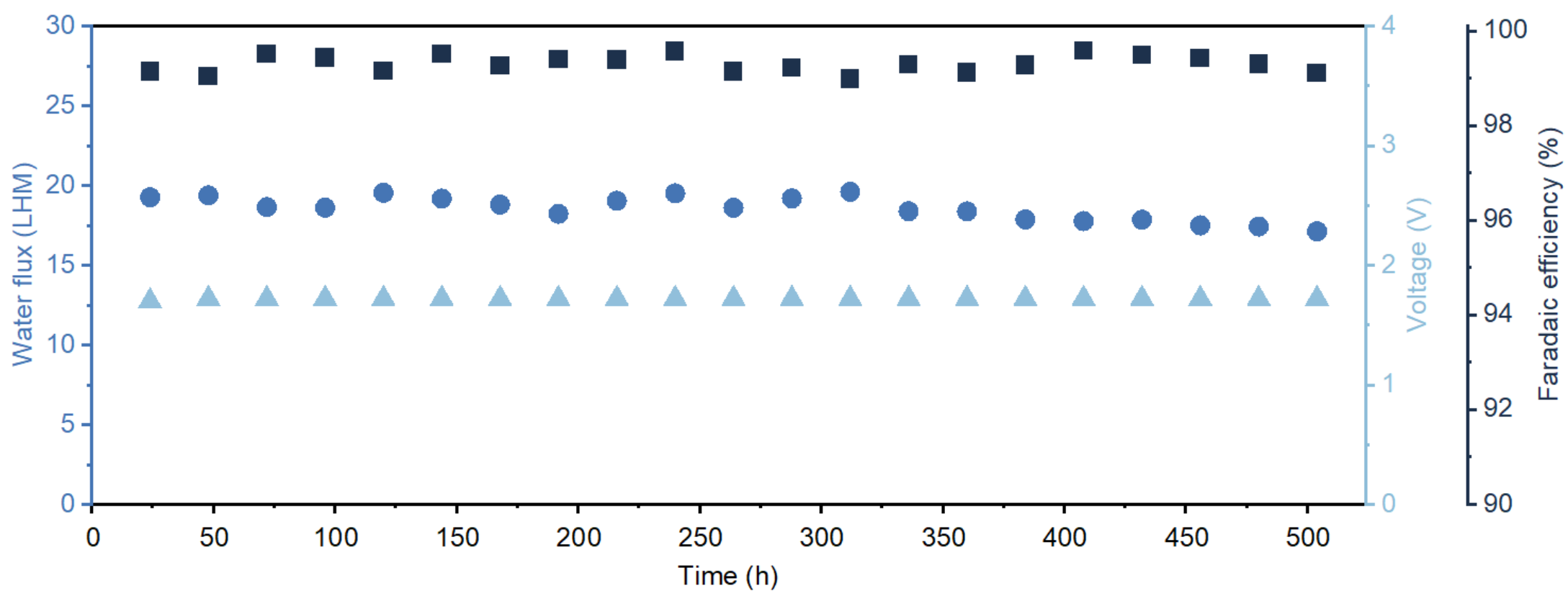

Fig. 3c A 500-h continuous OMD-AWE operation tracking water flux, cell voltage, and Faradaic efficiency without the addition of EDTA or any other antiscalants.

(2) The reviewer raises an excellent question regarding synergistic fouling (the co-precipitation of NOM and inorganic ions), which is a notorious problem in pressure-driven systems like RO. To investigate this in our thermally-driven system, we conducted scaling tests comparing the water flux decline between two feed conditions: (i) a gypsum-only solution and (ii) a solution containing gypsum with 10 mg/L Suwannee River NOM (SRNOM), as shown in Supplementary Fig. 9. Interestingly, contrary to the synergistic fouling observed in RO, we found that the presence of NOM actually mitigated inorganic scaling in OMD. After 7 hours of operation, the normalised water flux ($J_w/J_{w,0}$) was reduced to only 0.84 for the gypsum +SRNOM feed, doubled the value for the gypsum only. Such substantial difference suggests that organic matters interfered with gypsum crystallisation. This result is consistent with recent findings from Tong et al., (2023) [1] which revealed that NOM can adsorb to active crystal growth sites and increases the energy barrier for nucleation. This alters crystal morphology from sharp, invasive needles to rounded aggregates, thereby reducing the risk of pore wetting. The SEM and EDX analyses provide direct evidence for this organic-inorganic interaction. In the presence of SRNOM, gypsum crystals lost their sharp, invasive needle-like structures and instead formed rounded, composite aggregates that are far less likely to penetrate and wet the membrane pores (Supplementary Fig. 10). EDX analysis revealed

a dramatic 30-fold reduction in surface-bound inorganic scale, with the atomic concentrations of gypsum-associated elements (Ca, S, O) decreased from 34.23% to 1.17%, when SRNOM was present (Supplementary Fig. 11 and Table 14). In addition, the F/C atomic ratio, which serves as an indicator of the membrane surface coverage by carbonaceous deposits, decreased from the pristine value of 1.75 to 1.42 in the gypsum-only test and to 1.39 in the test with SRNOM. The slightly lower F/C ratio in the presence of SRNOM reflects the additional organic carbon contributed by the NOM itself, suggesting the formation of a mixed organic-inorganic layer where SRNOM is incorporated into the scaling matrix. Despite similar F/C ratios, the nature of fouling differed significantly. Gypsum-only testing showed extensive inorganic coverage (34.23%), while the gypsum + SRNOM testing yielded minimal inorganic deposition (1.17%) with organic matter as the dominant foulant.

These findings explain our 500-h seawater test results, where the EDX analysis revealed minimal mineral scaling on the membrane before cleaning. Because seawater naturally contains NOM, the organic matter acts as a natural crystallisation inhibitor. Therefore, rather than causing a severe synergistic collapse, the complex mixture in real seawater prevents hard inorganic scaling, making it easily cleaned and fully reversible during routine maintenance.

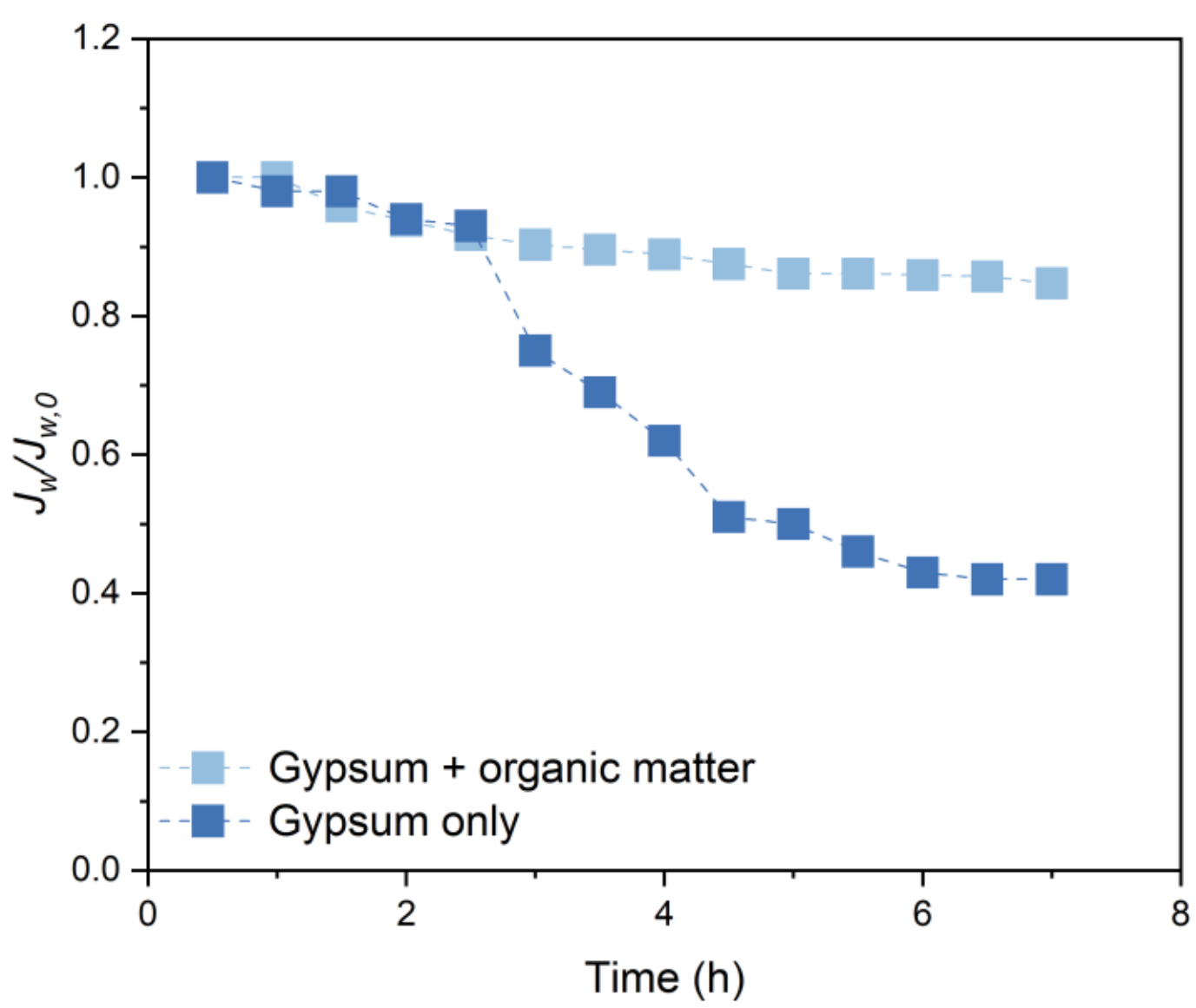

Supplementary Fig. 9 Normalised water flux during accelerated scaling tests in the presence and absence of organic matter. Conditions: [feed] = 30 mM Na_2SO_4 and 30 mM $CaCl_2$, [SRNOM] = 10 mg L^{-1} or 0 mg L^{-1}, [KOH] = 6.0 M, T_{feed} = 40°C, T_{KOH} = 20°C.

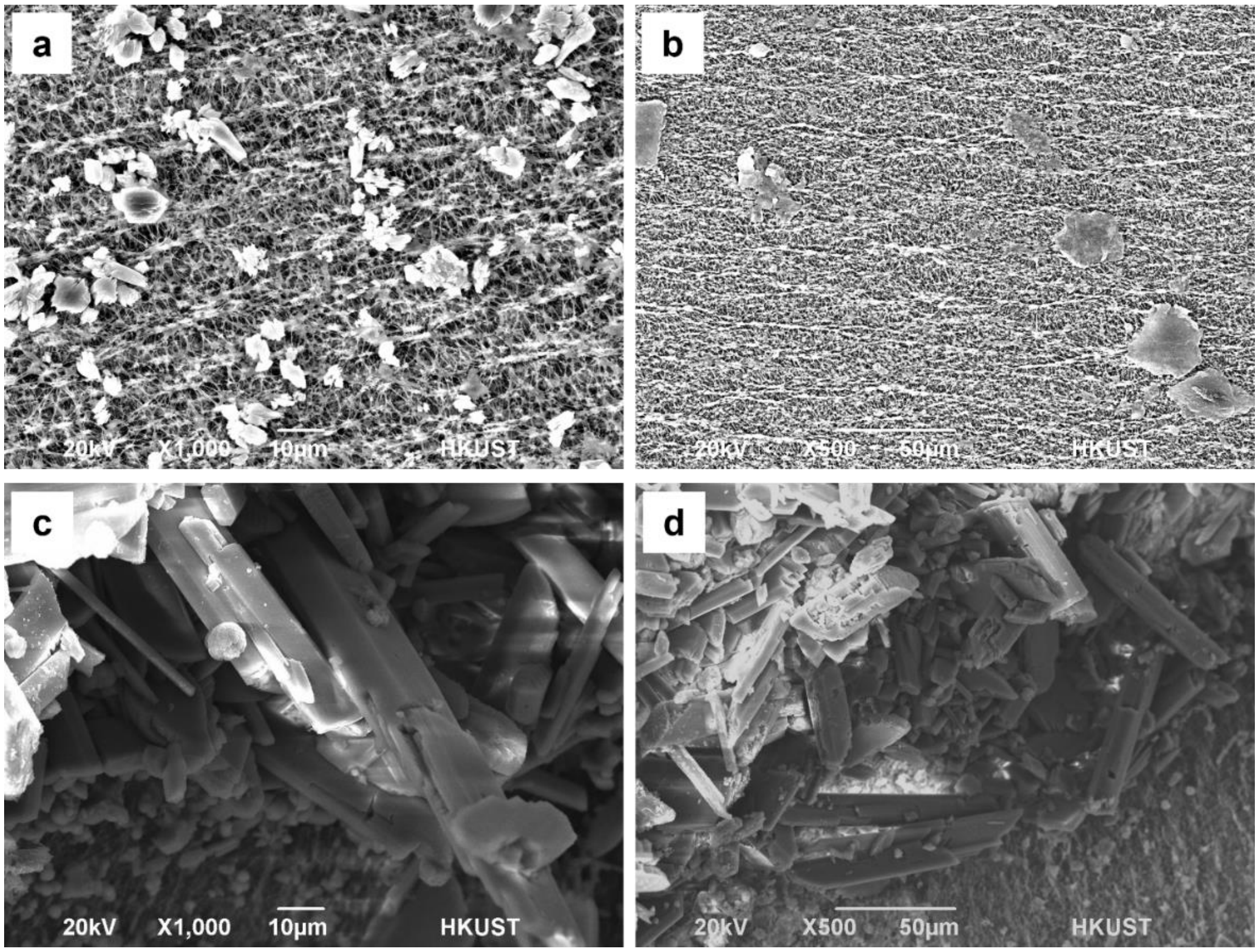

Supplementary Fig. 10 SEM images of the PTFE membrane following accelerated scaling tests in the presence and absence of organic matter. a–b Gypsum + SRNOM; **c–d** gypsum-only. Conditions: The gypsum solution contains 30 mM Na_2SO_4 and 30 mM $CaCl_2$, while the concentration of SRNOM is 10 mg L^{-1}. T_{feed} = 40°C, T_{KOH} = 20°C.

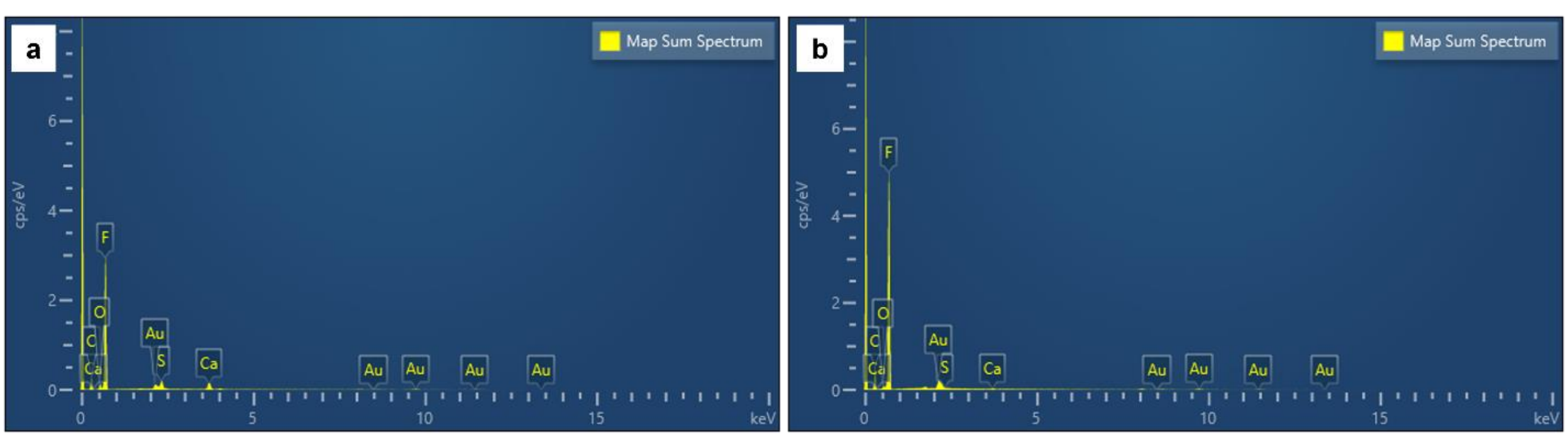

Supplementary Fig. 11 EDX spectrum of the PTFE membrane following accelerated scaling tests in the presence and absence of organic matter. a–b Gypsum + SRNOM; **c–d** gypsum-only. Conditions: The gypsum solution contains 30 mM Na_2SO_4 and 30 mM $CaCl_2$, while the concentration of SRNOM is 10 mg L^{-1}. T_{feed} = 40°C, T_{KOH} = 20°C.

Supplementary Table 14 Surface elemental composition analysis of the PTFE membrane during accelerated scaling tests. EDX showing surface elemental composition of a PTFE membrane without cleaning using gypsum + SRNOM as the feed and gypsum only as the feed.

PTFE membrane	Element	Wt%	Wt% Sigma	Atom (%)
Pristine	C	26.55	0.72	36.37
	F	73.45	0.72	63.63
	Others*	0	0	0
	F/C ratio	2.77	–	1.75
Gypsum only	C	18.43	1.15	27.16
	F	41.43	0.84	38.6
	Others*	40.14	–	34.23
	F/C ratio	2.25	–	1.42
Gypsum+SRNOM	C	30.81	0.77	41.29
	F	67.91	0.78	57.54
	Others*	1.28	–	1.17
	F/C ratio	2.20	–	1.39

*"Others" include Ca, S, and O.

These are added to the **newly revised manuscript**

Lines 217–222: "While these accelerated tests utilised inorganic salts, real seawater contains natural organic matter (NOM) that mitigates inorganic scaling by transforming mineral deposits from invasive needle-like structures to spherical aggregates through adsorption at crystal growth sites (Supplementary Figs. 9–11 and Table 14). This natural inhibition prevents hard scale formation, resulting in a foulant layer that is easily reversible during routine maintenance." with detailed discussion in Supplementary Figs. 9–11 and Table 14."

References:

1. Tong, T., Liu, X., Li, T., Park, S. & Anger, B. A tale of two foulants: the coupling of organic fouling and mineral scaling in membrane desalination. *Environ. Sci. Technol.* **57,** 7129–7149 (2023).

Q11: The PTFE membrane is subjected to a consistent ~20°C temperature gradient across its structure during operation. While PTFE is known for its thermal stability, the long-term effect of this steady-state thermal gradient on the membrane's mechanical integrity requires further discussion.

(1) Could author please discuss the potential for thermomechanical stress buildup within the membrane structure? Specifically, could the thermal expansion coefficient mismatch between the

PTFE polymer and any potential reinforcing materials (or between the membrane and its module housing) lead to issues such as creep or delamination over extended operation?

(2) Furthermore, is there any post-mortem evidence from the 500-hour test—such as visual inspection, SEM imaging of the membrane cross-section, or records of seal failure—that indicates physical deformation (e.g., warping) of the membrane or a loss of integrity at the sealing interfaces? Empirical evidence is crucial to rule out these potential failure modes.

Reply:

We appreciate this important consideration regarding the thermomechanical stability.

(1) The PTFE membrane's intrinsic mechanical properties serve as the primary mitigation against thermomechanical stress accumulation. Unlike rigid ceramic membranes that are susceptible to cracking due to thermal expansion mismatches, PTFE is a semi-crystalline polymer with low Young's modulus (~0.5 GPa) which facilitates elastic deformation and enables the material to absorb the thermal strain induced by the 20°C gradient without generating destructive internal stresses. In our lab setup, we utilised a homogeneous, unsupported PTFE flat-sheet membrane sandwiched between the feed and permeate chambers and secured by fluoroelastomer sealing rings. This configuration provides uniform mechanical support across the active area, effectively preventing warping or bowing. The elastomeric nature of the fluoroelastomer seals further accommodates minor thermal expansion at the fixation points without inducing stress concentrations. The absence of a backing layer or reinforcement inherently eliminates the risk of internal thermal expansion coefficient mismatch, eliminating the possibility of interlayer delamination. Furthermore, the system operates near atmospheric pressure, distinguishing it from high-pressure processes like RO where membrane compaction is a significant risk. Under this negligible mechanical load and moderate temperature (<40°C), the PTFE material operates well below its creep threshold, making plastic deformation or thickness reduction highly unlikely even over extended operation.

Regarding practical upscaling to commercial spiral-wound modules, where Polypropylene (PP) supports are typically employed for reinforcement, the risk of delamination remains negligible due to material compatibility. Both PTFE and PP are semi-crystalline polymers with comparable thermal expansion characteristics. Unlike rigid polymer-ceramic composites, a PTFE-PP interface expands and contracts synchronously under moderate operating temperatures (<80°C), preserving interfacial bonding. Additionally, from a system design perspective, the polymeric (PP/PE) mesh spacers within the module function as flexible mechanical buffers. These spacers effectively absorb the CTE mismatch between the membrane and the rigid housing, preventing the wrinkling or physical deformation often observed in rigidly constrained systems.

(2) Following the 500-h continuous test, visual inspection upon disassembly of the membrane cell confirmed that the PTFE membrane remained flat and taut, with no macroscopic evidence of physical deformation, warping, or bowing, and the fluoroelastomer sealing interfaces remained completely intact. Post-mortem SEM and AFM imaging of the cleaned membrane confirmed that

the characteristic node-and-fibril microstructure of the PTFE remained pristine, with absolutely no evidence of fiber fracture, pore collapse, or surface densification, definitively indicating that the material did not suffer from thermomechanical fatigue. The most sensitive and immediate indicator of mechanical failure is the salt rejection performance, as any micro-cracking in the membrane or loss of integrity at the sealing interfaces would have resulted in immediate electrolyte leakage, a spike in feed conductivity, and a drop in Faradaic efficiency (Figs. 3c and g). The sustained >99% salt rejection and the stable voltage and Faradaic efficiency recorded throughout the entire 500-h test serve as definitive, continuous proof that the membrane maintained absolute mechanical and sealing integrity under the steady-state thermal gradient.

These are added to the **newly revised Manuscript**:

Lines 336–339: "Utilising its intrinsic elasticity under near-atmospheric pressure, the membrane safely absorbed the continuous 20°C thermal gradient, prevented any micro-cracking, delamination, or seal failure, as evidenced by the sustained high salt rejection (Fig. 3d) and stable KOH concentration (Supplementary Fig. 17)."

Responses to comments of Reviewer 3:

This revised manuscript reconsidered the merits and limitation for a membrane-integrated seawater electrolysis process of OMD-AWE to produce hydrogen with water electrolysis in alkaline condition, addressing the challenge in H_2 production from non-freshwater sources. Their research provides a flexible design framework, enabling the OMD and AWE at any configuration while determining optimal operational windows for thermal self-sufficiency at any scale, moreover, the results showed a record-high H_2 production rate with running over 500 h with direct seawater electrolysis.

One point needs to clarify that the influence of ocean life upon PTFE membrane fouling due to hydrophilic species, which usually changes surface hydrophobic properties and reduces desalination in OMD. In generally, comparing with the original version, the revised manuscript has obtained obvious improvement.

Reply:

We sincerely thank you for this important comment. We fully agree that the influence of ocean life, specifically marine microorganisms and their hydrophilic extracellular polymeric substances (EPS), is a pervasive challenge that warrants careful consideration for any membrane system deployed in real marine environments. The accumulation of such hydrophilic species can alter the membrane's hydrophobic surface properties. Although the hydrophobic nature of PTFE makes initial microbial adhesion thermodynamically unfavourable, this barrier can be overcome over time as dissolved organic matter in seawater can adsorb to the membrane surface, forming a nutrient-rich conditioning film that reduces the thermodynamic barrier for microbial attachment. Once colonised, microorganisms proliferate and secrete EPS, which anchors the developing biofilm and progressively renders the surface more hydrophilic. This transformation increases the risk of pore wetting and flux decline, ultimately compromising mem performance.

To directly address this concern and evaluate the membrane performance under realistic biological conditions, our 500-h continuous test was conducted using raw, biologically active seawater. The feed was pre-filtered only through a 5 μm cartridge filter. This removed large suspended solids and algae, but it is insufficient to retain the majority of marine bacteria (typically 0.2–2 μm) or viruses (0.02–0.3 μm). Therefore, the feed stream retained a natural microbial population throughout the 500-h test, providing a rigorous evaluation of the membrane's susceptibility to biofouling and EPS accumulation. We conducted a comprehensive post-mortem analysis of the membrane, including scanning electron microscopy coupled with energy-dispersive X-ray spectroscopy (SEM-EDX), X-ray diffraction (XRD), atomic force microscopy (AFM), and X-ray photoelectron spectroscopy (XPS). They confirmed that while organic fouling did occur, it was reversible and caused no permanent degradation to the membrane's intrinsic hydrophobicity or structure.

SEM (Fig. 3c) and EDX (Supplementary Fig. 14 and Table 15) of the uncleaned membrane provided clear evidence of organic fouling. The surface fluorine-to-carbon (F/C) atomic ratio decreased from 1.75 (pristine) to 1.53 after operation, indicating surface coverage by carbonaceous

deposits, which is consistent with the accumulation of EPS and microbial matter alongside inorganic scale. Critically, the reversibility of this mixed organic-inorganic biofilm was demonstrated through a standard cleaning procedure (30 min of 0.05 M EDTA followed by a deionised water rinse). Following this treatment, the atomic F/C ratio recovered to 1.69, closely approaching the pristine value. XPS results further confirmed the effective removal of carbonaceous and oxygen-rich organic deposits, restoring the original surface chemistry and hydrophobicity (Supplementary Fig. 15). Biofilm formation and localised pH changes from microbial metabolism can sometimes cause the degradation or etching of polymeric materials; however, AFM revealed an intact node-and-fibril structure, with nanoscale surface roughness remaining within 5% of the pristine baseline, ruling out EPS-induced pore collapse or widening (Fig. 3f). XRD quantitative analysis showed a <5% reduction in crystallinity, indicating the bulk polymer structure remained completely stable.

In summary, our data indicate that the PTFE membrane remained structurally and chemically robust throughout the 500-ho exposure to biologically active seawater. The membrane's inherent hydrophobicity effectively limited irreversible biofilm anchoring. While a composite layer of EPS and mineral scale did form, it proved highly reversible with a simple cleaning protocol, ensuring long-term stability.

We have updated our 500-h test to incorporate these insights in the **newly Revised Manuscript**:
Lines 311–336: "The OMD demonstrated a stable water flux of 19.05 ± 0.41 LMH for the first 300 h before gradually declining to 17.79 ± 0.42 LMH by the end of the test (Fig. 3c). This minor decline is due to the initial fouling formation as suggested by scanning electron microscopy (SEM) characterisation of the of the uncleaned membrane surface after 500-h use (Fig. 3e). Energy-dispersive X-ray spectroscopy (EDX) identified this foulant layer as a mixture of inorganic scale (Ca, Mg, S, and O at 2.32 atom%) and carbonaceous deposits, evidenced by a decrease in the surface F/C atomic ratio from 1.75 to 1.53 (Supplementary Fig. 14 and Table 15). Since the 500-h test utilised raw, biologically active seawater, these carbonaceous deposits might include marine microorganisms and their hydrophilic extracellular polymeric substances (EPS). Importantly, this mixed fouling proved highly reversible after EDTA-based cleaning. Post-cleaning EDX showed that the inorganic scaling elements dropped to 0 atom% and the F/C ratio recovered to 1.69, indicating the removal of all mineral scale and most carbonaceous deposits. X-ray photoelectron spectroscopy (XPS) confirmed this reversibility, showing significantly diminished Ca, Mg, C, and O peaks after cleaning (Supplementary Fig. 15). To remove residual organic foulants and prevent long-term biofilm accumulation, widely used high-pH alkaline cleaners could be used in addition to EDTA cleaning36. Incorporating antiscalant chemicals into the feed or implementing basic prefiltration (e.g., sand filters) can also reduce scaling risk and extend cleaning intervals. Beyond reversible fouling, the PTFE membrane exhibited exceptional thermomechanical and chemical stability. SEM and ImageJ analysis of the cleaned membrane revealed an intact node-and-fibril microstructure with no pore collapse or widening (Fig. 3e, Supplementary Fig. 16). X-ray diffraction (XRD) and atomic force microscopy (AFM) confirmed that bulk crystallinity and

nanoscale roughness remained virtually unchanged (Figs. 3f–g), ruling out any polymer etching or degradation caused by microbial metabolism. XPS further verified chemical integrity, showing perfectly matched carbon–fluorine and elemental fluorine spectra with no evidence of defluorination or chlorine incorporation.”

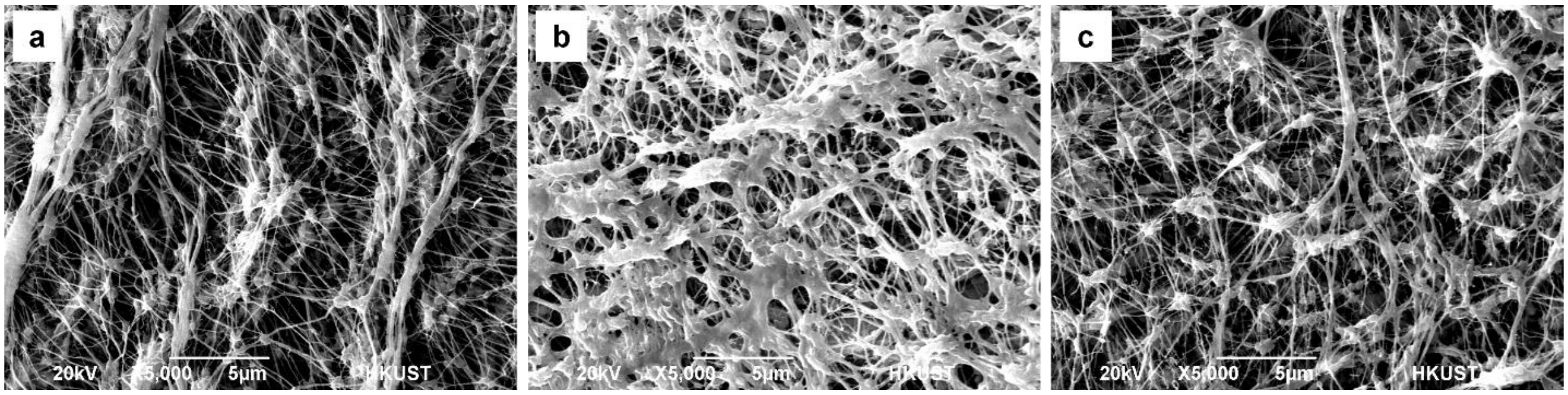

Fig. 3d Scanning electron microscopy (SEM) images of the PTFE membrane. SEM image showing the surface morphology of **a** the pristine PTFE membrane; **b** the PTFE membrane after 500-h operation without cleaning; and **c** the cleaned PTFE membrane after 500-h operation. Conditions: [feed] = seawater, [KOH] = 6.0 M, T_{feed} = 40°C, T_{KOH} = 20°C.

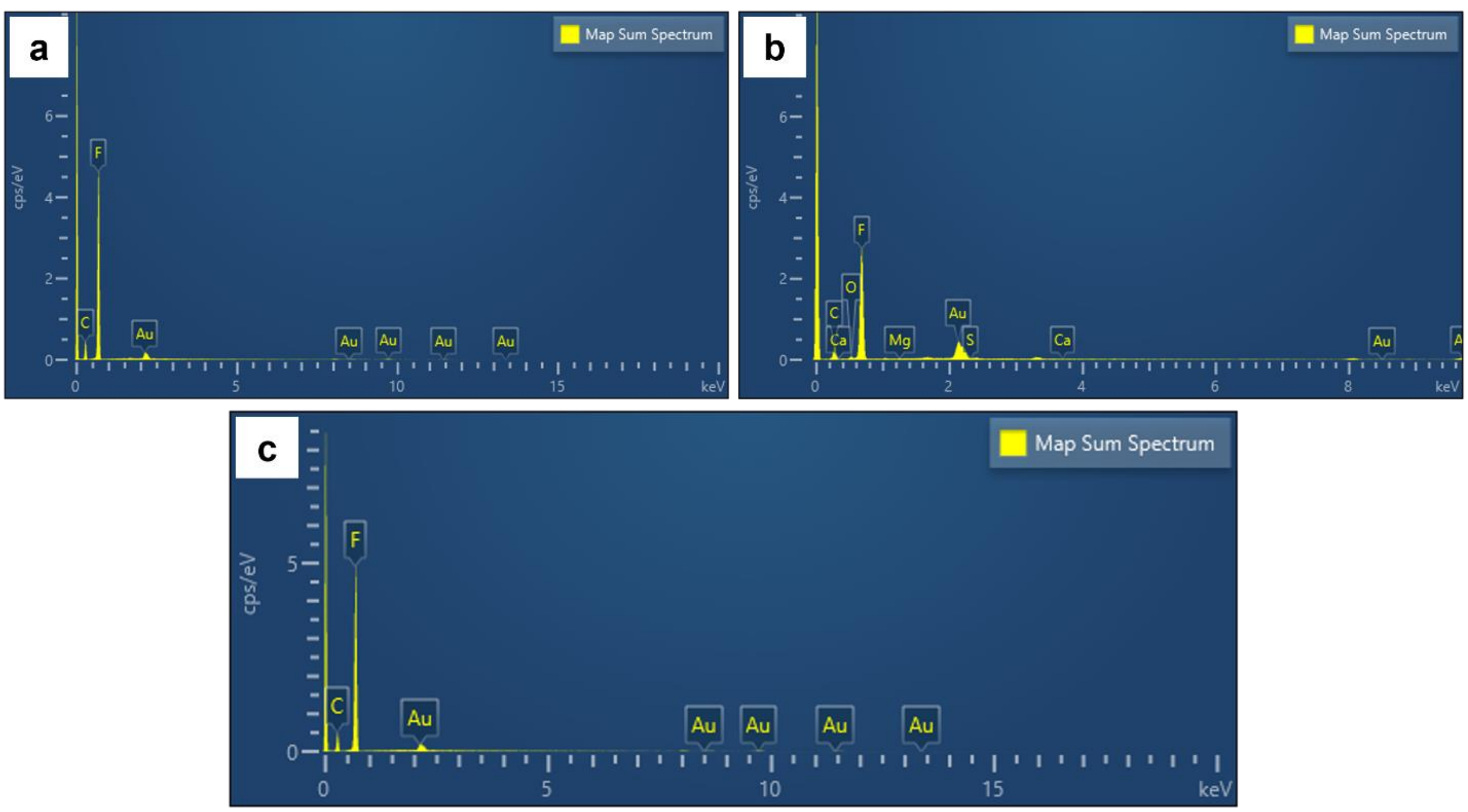

Supplementary Fig. 14 Energy-dispersive X-ray spectroscopy (EDX) spectrum of the PTFE membrane. EDX spectrum showing the surface elemental composition of **a** the pristine PTFE membrane; **b** the PTFE membrane after 500 h operation without cleaning; and **c** the cleaned PTFE membrane after 500 h operation. Conditions: [feed] = seawater, [KOH] = 6.0 M, T_{feed} = 40°C, T_{KOH} = 20°C.

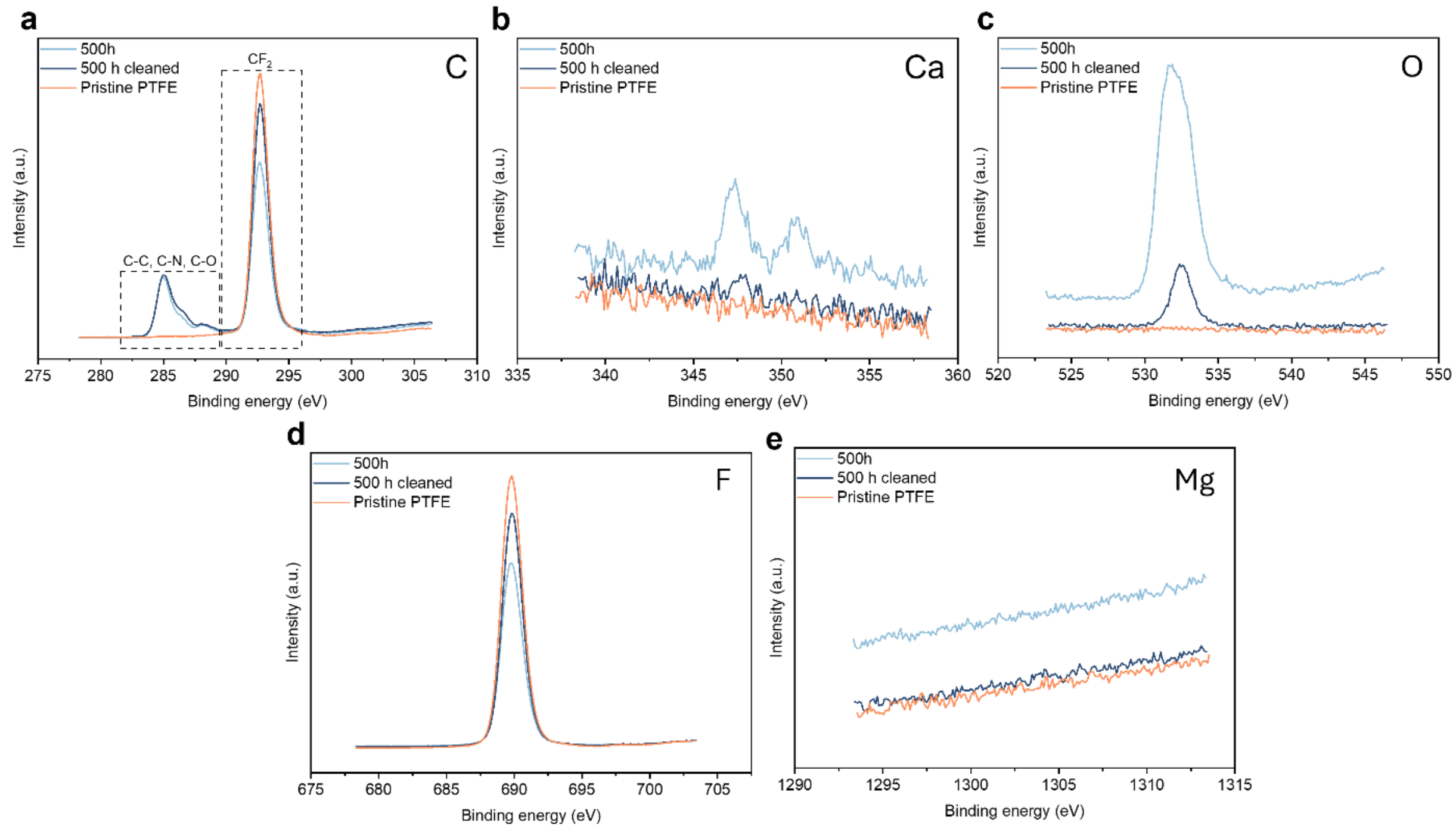

Supplementary Fig.15 High-resolution XPS spectrum of the pristine PTFE membrane, the PTFE membrane after 500-h operation without cleaning, and the cleaned PTFE membrane after the 500-h operation, showing the regions for **a** carbon (C); **b** calcium (Ca); **c** oxygen (O), **d** fluorine (F); and **e** magnesium (Mg).

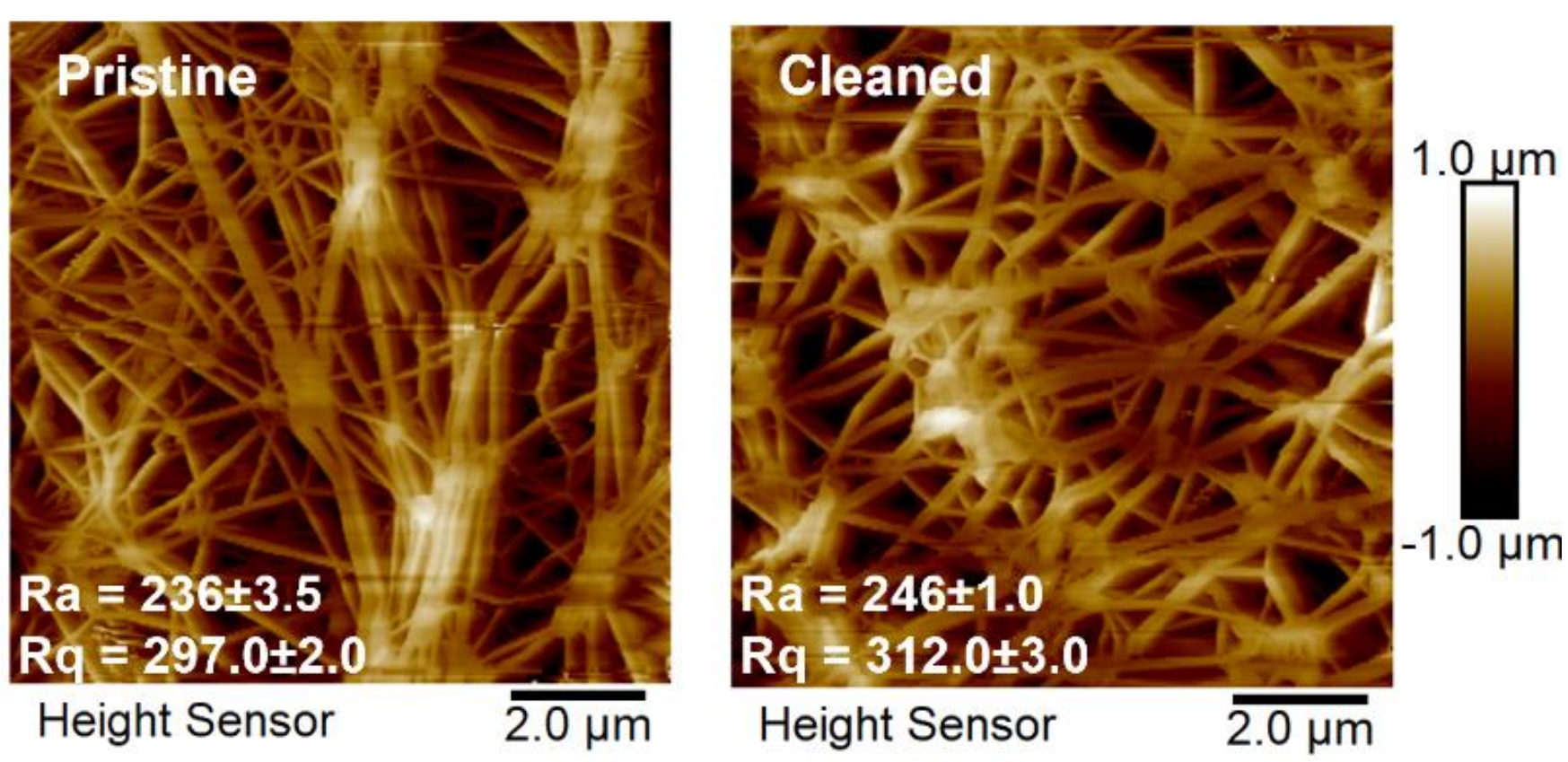

Fig. 3f Atomic force microscopy (AFM) images showing the surface morphology and roughness of a pristine PTFE membrane and the cleaned PTFE membrane after 500-h operation.

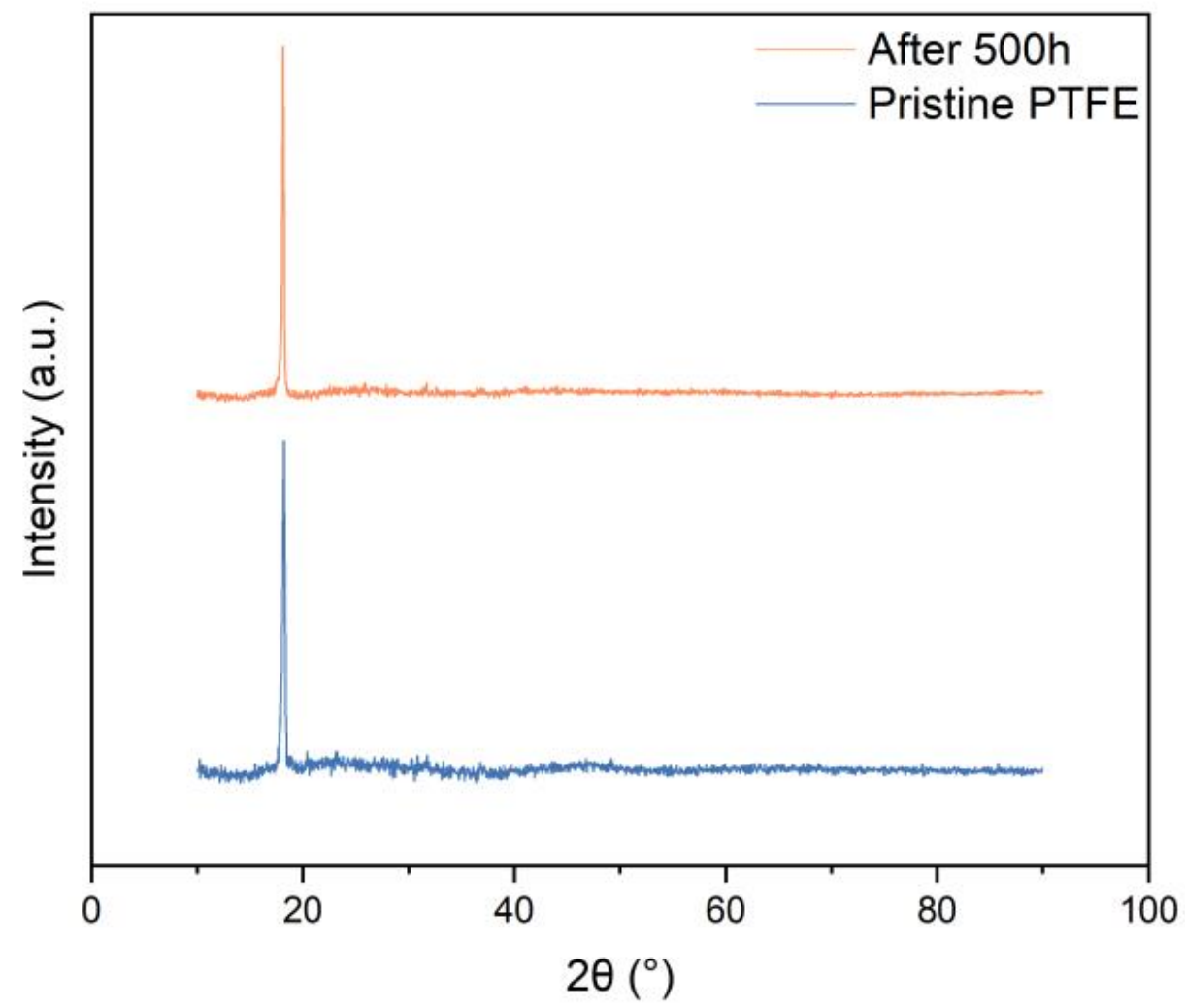

Fig. 3e X-ray diffraction (XRD) analysis of the PTFE membrane. XRD patterns of **a** the pristine PTFE membrane; and **b** the PTFE membrane after 500-h operation. Conditions: [feed] = seawater, [KOH] = 6.0 M, T_{feed} = 40°C, T_{KOH} = 20°C.

Supplementary Table 15. Surface elemental composition analysis of the PTFE membrane. The energy-dispersive X-ray spectroscopy (EDX) showing surface elemental composition of a pristine PTFE membrane and the PTFE membrane after 500 h of operation before and after cleaning.

PTFE membrane	Element	Wt%	Wt% Sigma	Atom (%)
Pristine	C	26.55	0.72	36.37
	F	73.45	0.72	63.63
	Others*	0	0	0
	F/C ratio	2.77	–	1.75
After 500 hours without cleaning	C	28.54	0.97	38.58
	F	69.13	0.97	59.09
	Others*	2.33	–	2.32
	F/C ratio	2.42	–	1.53
After 500 hours cleaned	C	27.28	0.35	37.24
	F	72.71	0.35	62.75
	Others*	0	0	0
	F/C ratio	2.67	–	1.69

*Others include Ca, Mg, S, and O.

Responses to comments of Reviewer 4:

The authors have significantly improved the manuscript in response to the reviewers' comments.

The additional experiments, long-term operation, and expanded TEA/LCA analysis strengthen the robustness and practical relevance of the work.

However, I remain unconvinced that the study delivers a sufficiently fundamental conceptual advance for Nature Communications.

In my view, the main contribution lies in a careful system-level integration and benchmarking of already known elements—alkaline water electrolysis, osmotically driven membrane distillation, thermal coupling, and low-temperature/low-recovery operation—rather than in a genuinely new physical or chemical principle.

The three "counter-intuitive" design guidelines emphasized by the authors appear to formalize trade-offs (between temperature, fouling/wetting, and recovery) that are already qualitatively recognized in the MD and electrolysis communities.

While this does not diminish the practical value of the proposed configuration, it does limit the degree of novelty, and I would characterize the advance as incremental rather than transformative.

Reply:

We thank the reviewer for acknowledging the robustness, practical relevance, and comprehensive analysis of our work. The conceptual advance of our work lies in the creation of a **thermally symbiotic architecture** that merges principles from thermal engineering, desalination, and electrochemistry. By coupling OMD with AWE through precisely designed heat exchange loops, we transform a parasitic energy penalty (electrolyser waste heat) into the driving force for seawater desalination, while simultaneously providing passive cooling for the electrolyser. This phase-locked synchronisation of mass flow and energy flow establishes a self-regulating, zero-external-heat engine without affected by intermittent energy input. We believe these cross-disciplinary insights provide significant value and align with *Nature Communications*' mandate to inspire researchers from different fields to explore new directions in their respective domains from a fresh perspective. Regarding the trade-offs between temperature, fouling/wetting, and recovery, we agree these are well-recognised in isolated MD and electrolysis. However, we clarify that formalising these trade-offs is not the primary innovation but serves as the critical operational conditions that make our thermally symbiotic architecture physically possible. The novelty of our thermally symbiotic architecture becomes evident through several key advances:

1. A new, viable direction for both desalination and direct seawater electrolysis community

Although the individual physical and chemical phenomena (MD, AWE) are indeed established, the thermodynamic principle governing our thermally symbiotic architecture is entirely new. We argue that transformative cross-disciplinary paradigm shifts do not require the discovery of "genuinely new physical or chemical principles" to achieve breakthrough innovation. In fact, the discovery of genuinely new chemical or physical principles is not only exceedingly rare, but such discoveries

often require decades of subsequent development before reaching application potential. Instead, many impactful and deployable breakthroughs in water-energy engineering were inspired by known elements from other areas to solve previously insurmountable disciplinary bottlenecks. For instance, the pioneering work by Veroneau & Nocera (*Proc. Natl. Acad. Sci. U. S. A.,* 2021, 118 [1]) demonstrated that direct electrolysis of impure water does not strictly depend on inventing new catalysts, but rather came from using commercially available membranes from the water treatment field to physically isolate impurities. Subsequently, the landmark work by Xie et al. (*Nature*, 2022, 612, 673–678 [2]) further evolved this paradigm by leveraging well-established PTFE hydrophobic membranes for gas-phase water transport to achieve the near-perfect ion rejection required for complex seawater environments. Similarly, in direct solar-driven H_2 production, the traditional focus was on developing novel photocatalysts. However, with the advancement of photovoltaic (PV) technology, the breakthroughs have come from coupling mature PV panels with electrolysis, achieving solar-to-hydrogen efficiencies of 12% (*Energy Environ. Sci.*, 2025, 18, 5264–5276 [3]) and even 30% if without considering costs (*Sci. Adv.*, 2025, 11(9) [4]), far surpassing the <1% of photocatalysis studied over decades. These demonstrate that combining mature elements to bypass fundamental bottlenecks constitutes a profound conceptual advance. Our thermally symbiotic architecture follows this trajectory of cross-disciplinary innovation. It is not merely "integration" of MD and AWE but rather a new thermodynamic architecture that simultaneously breaks through the fundamental bottlenecks of two distinct fields:

For decades, MD has been recognised for its exceptional potential in salt rejection. However, its industrial application has been limited by extensive thermal requirements. Thus, unlike widely adopted reverse osmosis (RO), MD has been trapped in a developmental bottleneck. The field has either remained entirely dependent on co-location with external industrial waste heat sources or attempted to improve thermal efficiency through complex module designs and novel photothermal materials, both of which offer limited practical success. This reliance on external heat restricts the geographical deployment and scalability of MD, especially in remote or offshore environments where external heat sources are unavailable. Our work provides a new and viable direction for MD by directly coupling it with electrolysis for H_2 production. We demonstrate that MD and AWE function as perfect thermodynamic complements, i.e., the waste heat generated by AWE matches the thermal driving force required by MD which simultaneously provides cooling for AWE to maintain its temperatures. This coupling eliminates the need for external heat, transforming MD from a heat-dependent technology into a self-sustaining solution.

Similarly, for direct seawater electrolysis (DSE), the dominant approach focuses on developing complex, chlorine-resistant, and selective catalysts. While this direct route holds scientific value, it struggles with long-term durability and lacks near-term application potential. Our work shifts this focus from catalyst development to thermodynamic engineering. While previous studies have explored membrane-based separation to physically isolate the electrolyser from impurities, these methods fail when confronted with the complex environment of seawater and the constraints of offshore applications where heat source is not available. Specifically, forward osmosis (FO) suffers from insufficient salt rejection, while isothermal OD (i.e., what SDE-DSE uses) is bottlenecked by its extremely low water yields. We introduced a new thermal management approach, i.e., the thermally symbiotic OMD-AWE, which overcomes these critical limitations. By synergistically

coupling the osmotic driving force with in situ waste heat generated by the electrolyser, we break the isothermal barrier. This creates a powerful dual-driving force that achieves massive, stable water flux with near-perfect salt rejection, without requiring external heating infrastructure. Consequently, we demonstrate that research does not need to invent entirely new catalyst principles to achieve stable seawater splitting; instead, they can leverage our thermally symbiotic architecture to unlock the use of proven, inexpensive, pure-water catalysts for direct seawater electrolysis.

2. Self-regulating thermal circulation overcomes heat scarcity and renewable intermittency

The practical deployment of current membrane-integrated electrolysis for green H_2 production is fundamentally hindered by two thermodynamic roadblocks, heat scarcity and intermittent energy. In offshore wind applications, the absence of external heat sources is the major barrier that makes state-of-the-art SDE-DSE impractical to scale. By relying only on weak osmotic driving force, the system generates extremely low water fluxes; scaling it to match the massive water consumption of a commercial electrolyser requires a large membrane footprint, rendering them physically unsuitable for space-constrained offshore environments. In another common green H_2 scenario, i.e., PV-electrolysis, solar heat can be harvested via photovoltaic-thermal (PVT) systems to drive MD, thus making it possible to produce water much more than SDE-DSE, but their thermal and electrical outputs are asynchronous, creating a mismatch between water production and water consumption. On cool, sunny mornings, high PV electrical efficiency drives high MD water consumption, but low thermal input reduces electrolytic water production. At midday, overheated PV panels reduce electrical efficiency while thermal water production peaks. Crucially, this mismatch is exacerbated by the inherent intermittency of renewable energy in both scenarios, creating cumulative imbalances that destroy the system's water balance with time. This leads to either system dry-out which causes corrosion, localised overheating, and KOH crystallisation that blocks electrolyser flow channels, or electrolyte dilution which increases ohmic resistance, collapses the osmotic driving force for subsequent high-power operation, and requires additional energy-intensive separation processes. Managing this mismatch via frequent shutdown and restart is highly destructive to hydrophobic membranes. Shutdown creates stagnation, high-salinity boundary layers at the membrane surface where salts quickly become supersaturated and crystallise on and within pores, causing irreversible scaling. Frequent hydraulic shocks from pump restarts cause mechanical fatigue in the polymer matrix, lowering the liquid entry pressure (LEP) and inducing pore wetting.

Our thermally symbiotic OMD-AWE system introduces a fundamentally new physical architecture that resolves both bottlenecks simultaneously. By capturing the in situ electrolyser waste heat to drive the OMD process, our system eliminates the need for external heat sources, enabling massive, scalable water fluxes even in heat-scarce offshore wind environments. This architecture establishes an intrinsic self-regulating mechanism that overcomes renewable intermittency. Since electrolyser waste heat generation is directly proportional to applied electrical current, the thermal driving force for water production is synchronised with electrical demand for water consumption without complex and destructive on/off control during intermittent power input. These highlight the

transformative nature of our thermally symbiotic OMD-AWE, which provides the first truly viable pathway for in situ green H_2 production from seawater.

3. Broader implications for the water-energy nexus

Beyond green H_2 production, this thermal symbiosis establishes a broader design philosophy that inspires a wide range of next-generation water-energy technologies. For example, this architecture can be directly extended to other exothermic electrochemical processes, such as electrochemical CO_2 reduction and ammonia synthesis. In CO_2 reduction, large kinetic overpotentials inevitably generate substantial parasitic waste heat, while the downstream separation of liquid products (e.g., ethanol, formate) from the aqueous electrolyte remains a massive energy bottleneck. By applying our thermally symbiotic architecture, this waste heat could be recycled to drive membrane-based pervaporation or distillation, achieving in situ product recovery without external thermal input. Similarly, in electrochemical mineral recovery (e.g., lithium extraction), the substantial Joule heat generated during ion capture can be repurposed to drive membrane distillation processes that simultaneously provide cooling and extract pure water from brine. This in situ generated pure water serves as the receiving solution to release and concentrate extracted minerals, while continuous water removal drives residual brine towards zero-liquid discharge. Across all these applications, this architecture shifts the traditional chemical engineering paradigm by unifying reaction inefficiencies (waste heat) and mass-transfer demands (separation) into a self-sustaining thermodynamic loop, rather than treating them as isolated energy penalties.

To support point 1, we have revised the following texts to **the newly revised manuscript:**

To avoid confusion in novelty, **the former texts in Lines 718–721**: "The system is designed under a new philosophy that reverses conventional membrane principles: i) a milder temperature is optimal for stability, ii) high energy demand is an asset rather than a liability, enabling full absorption and utilisation of electrolyser waste heat, and iii) low water recovery creates a powerful heat transfer engine for high, stable water flux." has been revised as:

Lines 656–659: "By coupling OMD and AWE as perfect thermodynamic complements, this architecture shifts the paradigm of seawater splitting from complex catalyst engineering to thermodynamic system design, simultaneously solving the heat-dependency bottleneck of membrane distillation and overcoming the low H_2 yield and catalyst durability limitations of direct seawater electrolysis."

We also add texts in **Lines 76–78:** "Crucially, the absence of external heat sources in remote or offshore environments is the fundamental barrier preventing SDE-DSE from overcoming its water supply limitations, severely restricting its geographical deployment and scalability."

For point 2, we added the following texts in **Abstract**:

Lines 27–30: "This thermal symbiosis establishes a self-regulating mechanism that phase-locks thermal driving force to fluctuating electrical inputs, synchronising water supply with demand to overcome renewable intermittency."

We also added a new section titled "Dynamic self-regulation under intermittent renewable energy input", with methods provided in Supplementary Note 5.

Lines 530–596: "In membrane-electrolysis, the most critical risk is the cumulative mismatch between water production and consumption from intermittent renewable energy input. If production exceeds consumption (e.g., during power lulls), the continuous water influx causes electrolyte dilution, system overflow, and collapse of osmotic driving force, requiring additional energy-intensive separation processes. Conversely, excess consumption during peak power causes progressive dry-out. The continuous removal of water causes KOH concentration to spike, leading to localised corrosion, overheating and KOH crystallisation that blocks electrolyser flow channels. Managing this mismatch through frequent shutdown and restart cycles is highly destructive to hydrophobic membranes. Shutdown creates stagnation and high-salinity boundary layers at the membrane where salts quickly become supersaturated and crystallise, causing irreversible scaling. Frequent hydraulic shocks from pump restarts cause mechanical fatigue in the polymer matrix, lowering LEP and inducing pore wetting. Our OMD-AWE overcomes this mismatch through dynamic self-regulation, because the thermal driving force (waste heat) is physically phase-locked to the input power, the system naturally adjusts water supply in exact proportion to electrolyser demand, ensuring continuous synchronisation without destructive on/off control. Although sudden power changes create brief delays before the system temperature fully adjusts, the circulating KOH acts as a liquid reservoir that accommodates temporary fluctuations in both volume and concentration. Since renewable energy fluctuations are cyclical, these transient deviations naturally cancel out over time, preventing cumulative mismatches. To validate these, we simulated the system's dynamic response using instantaneous power as the input variable (methods in Supplementary Note 5). As power input drops from the rated condition, the electrolyser current reduces proportionally, which lowers waste heat generation and naturally cools $T_{\mathrm{AWE,out}}$ and $T_{\mathrm{OMD,feed,in}}$ (Fig. 5a). Such temperature drop reduces ΔT_{OMD}, scaling down water supply to match the reduced electrolysis demand. In additions, the system naturally transitions to a higher-efficiency mode during partial loads, as the reduction in ohmic voltage losses at lower current densities dominates over slight increases in kinetic overpotentials at lower AWE temperatures, resulting in a 25% decrease in *SEC* (Fig. 5b).

We then examined the system stability under realistic solar and wind profiles characterised by significant intermittency (Supplementary Fig. 5). The stability was indicated by a water balance ratio of water production rate to water consumption rate, both derived from fitting the power profile to the system with methods detailed in Supplementary Note 5. Under intermittent solar power (Fig. 5c), our OMD-AWE maintains a stable ratio of around 1.0 during 09:00–17:00, even during highly intermittent morning hours with cloud cover. This contrasts with traditional photovoltaic-thermal (PVT)-MD systems which exhibit asynchronous responses throughout the day. In mornings, low ambient temperatures enhance PV electrical efficiency but insufficient thermal input limits water production. At midday, high temperatures maximise thermal input but reduce PV efficiency due to overheating. This mismatch creates a parabolic water balance curve with ratios varying from 0 to

1.8. Similarly, under erratic 24-h offshore wind profiles, the OMD-AWE also maintains near-perfect balance, contrasting with isothermal SDE-DSE whose water balance fluctuates wildly from 1 to over 10. (Fig. 5d). Over 24 h, SDE-DSE generates near 100% water surplus, causing catastrophic electrolyte dilution requiring energy-intensive separation to fix. The only exception to this dynamic self-regulation occurs during extreme power lulls (below 35% rated power). Under such power conditions, the electrolyser's water consumption is minimised, but the osmotic driving force of 6 M KOH continues to extract water, causing accumulation if left unchecked. Suspending feed supply during these off-periods prevents excess accumulation and enables maintenance activities like membrane cleaning to prepare for resumed high-power. These findings confirm that the OMD-AWE provides a universally self-regulating architecture, maintaining operational stability under intermittent power inputs without requiring external thermal infrastructure or complex control systems. Beyond thermodynamic water balance, the decoupling of the membrane and electrolyser ensures that power fluctuations do not compromise the PTFE membrane's intrinsic permeability or salt rejection. While intermittent operation poses known durability challenges to standard AWE catalysts, these are general electrochemical issues rather than limitations of our water-energy coupling mechanism. The chemical inertness of the PTFE barrier ensures our architecture is fully compatible with standard battery buffering or emerging intermittency-resistant electrodes, providing a comprehensive pathway for long-term electrochemical stability.

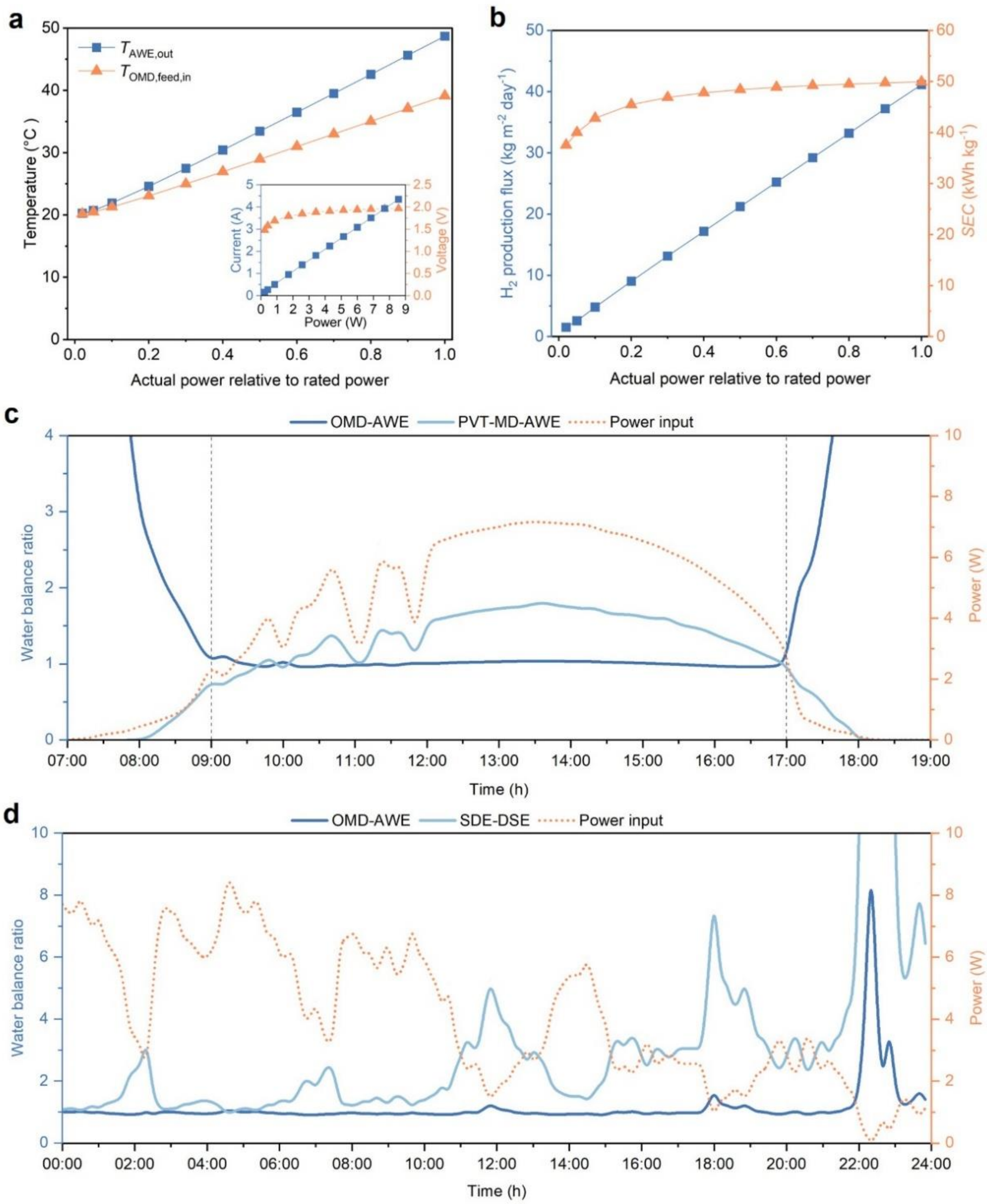

Fig. 5 The dynamic response of the thermally symbiotic OMD-AWE under intermittent power input. a the changes in $T_{\mathrm{AWE,out}}$ and $T_{\mathrm{OMD,feed,in}}$ as a function of the AWE power; **b** the changes in H_2 production flux and *SEC* as a function of the AWE power; **c** the comparison in water balance between OMD-AWE and PVT-MD-AWE under intermittent solar power input; **d** the comparison in water balance between OMD-AWE and SDE-DSE under intermittent wind power input. (The simulation considers a single-cell electrolyser operated at a current density of 0.5 A cm^{-2} with Ni nanowire-based cathode and NiFe-based anode separated by a ZIRFON diaphragm at an area of 7.5 cm^2 integrated with a 1-cm^2 PTFE membrane, with detailed system configurations and operating parameters shown in Supplementary Fig. 4)

For point 3, these insights were added to **Conclusion:**

Lines 673–679: "Beyond H_2 production, this thermal symbiosis architecture establishes a universal framework for other water-energy nexus challenges, e.g., CO_2 reduction and electrochemical mineral recovery. By repurposing Joule heating to drive MD-based separations, the system can simultaneously provide essential cooling, recover liquid chemical products, and extract pure water to drive residual brines toward zero-liquid discharge. This architecture transforms isolated engineering liabilities, such as waste heat and freshwater scarcity, into self-sustaining resource mining and chemical manufacturing engines."

Reference:

1. Veroneau, S. S. & Nocera, D. G. Continuous electrochemical water splitting from natural water sources via forward osmosis. *Proceedings of the National Academy of Sciences* **118**, (2021). https://doi.org/10.1073/pnas.2024855118
2. Xie, H., Zhao, Z., Liu, T., Wu, Y., Lan, C., Jiang, W., Zhu, L., Wang, Y., Yang, D. & Shao, Z. A membrane-based seawater electrolyser for hydrogen generation. *Nature* **612**, 673–678 (2022). https://doi.org/10.1038/s41586-022-05379-5
3. Wang, X., Gao, J., Wang, Y., Liu, Y., Liu, X., & Zhang, L. (2025). Over 12% efficiency solar-powered green hydrogen production from seawater. *Energy & Environmental Science*, **18(11)**, 5264–5276. https://doi.org/10.1039/d4ee06203e
4. Zhang, Q., Shan, Y., Pan, J., Kumar, P., Keevers, M. J., Lasich, J., Kour, G., Daiyan, R., Perez-Wurf, I., Thomsen, L., Cheong, S., Jiang, J., Wu, K., Chiang, C., Grayson, K., Green, M. A., Amal, R., & Lu, X. (2025). A photovoltaic-electrolysis system with high solar-to-hydrogen efficiency under practical current densities. *Science Advances*, **11(9)**, eads0836. https://doi.org/10.1126/sciadv.ads0836

Responses to comments of Reviewer 5:

I co-reviewed this manuscript with one of the reviewers who provided the listed reports. This is part of the Nature Communications initiative to facilitate training in peer review and to provide appropriate recognition for Early Career Researchers who co-review manuscripts.

Reply: We thank you for participating in the co-review process and appreciate your comments to help us improve our work.

Former title: Thermally symbiotic osmotic membrane distillation-electrolysis unlocks efficient and stable hydrogen production directly from seawater

New title: Thermally symbiotic integration of osmotic membrane distillation and electrolysis for direct seawater hydrogen production

Authors: Gabriela Scheibel Cassol, Chii Shang, Paul Westerhoff, Yinghao Song

Manuscript ID: NCOMMS-25-36631C

Responses to comments of Reviewer 1:

I would like to acknowledge that the authors have provided a very comprehensive response and have made detailed revisions to several technical aspects of the manuscript. The effort put into the updated manuscript and the clarity of certain experimental sections is evident.

However, regarding the core concept of the "thermally symbiotic" system, I find the current presentation somewhat misleading. The "Thermal Symbiosis" appears to be a theoretical projection rather than an experimentally validated achievement in this study. In the Abstract and Introduction, the authors describe a "thermally symbiotic system" that operates stably for 500 hours. Yet, in the Response Letter, it is admitted that heat loss was significant and that waste heat utilization was not actually realized in the experimental setup, relying instead on modelling simulations. This creates a discrepancy with the revised manuscript, which states that "Both pipes and heat exchangers are wrapped with aluminium-clad polyurethane foam insulation, resulting in negligible heat losses."

Please revise the title or the main text to clarify that the proposed "thermally symbiotic system" is a result of numerical modelling, rather than an experimental result of the 500-hour operation. Furthermore, if the primary novelty of this work lies in the "thermally symbiotic" system derived from thermodynamic calculations, I believe the current elaboration is not sufficiently profound or comprehensive. The authors might consider expanding this section to provide a more in-depth discussion.

Reply:

We sincerely thank you for such careful reading and for pointing out this critical misleading. We agree that the boundary between our experimental achievement and theoretical projections should be clear, and we appreciate the opportunity to refine our manuscript. To address this, we have made explicit revisions throughout the manuscript to clarify that the 500-h operation under expected thermal conditions (via external temperature controllers) was to prove the material and operational stability of the system, while the self-sustaining design was established through thermodynamic modelling. We have addressed your points as follows:

We have revised the **Title** to emphasize the broader architectural framework rather than specific experimental outcomes. The new title "Thermally symbiotic integration of osmotic membrane distillation and electrolysis for direct seawater hydrogen production" removes claims of "efficient and stable" performance and focuses on the integration concept itself.

We have adjusted **Abstract** and **Introduction** to indicate that the high H_2 yield and system stability was obtained experimentally before presenting thermally symbiotic architecture analysed by our thermal-water-hydrogen model as follow:

Lines 25–36: "Here we overcome these limitations by integrating osmotic membrane distillation (OMD) with alkaline water electrolysis (AWE). Driven by dual thermal and osmotic gradients to enhance salt-free water vapour transport, the OMD-AWE delivers a record-high H_2 production rate of 60 kg m^{-2} day^{-1} with excellent stability over 500 h of continuous operation. To eliminate the

external heating energy penalty of OMD, we propose a thermally symbiotic architecture that converts the AWE's waste heat to OMD's thermal driving force while OMD simultaneously providing cooling to maintain AWE optimal temperatures, as validated by our thermal-water-hydrogen model. This thermal symbiosis not only makes OMD-AWE energy self-sufficiency with best-in-class energy efficiency of 51 kWh $kg(H_2)^{-1}$ but also establishes a self-regulating mechanism that phase-locks thermal driving force to fluctuating electrical inputs, synchronising water supply with demand to overcome renewable intermittency."

Lines 102–116: "Here we present a thermally symbiotic architecture integrating OMD and AWE (Fig. 1). To establish the physical prerequisites essential for this symbiosis, we first experimentally determined the operational boundaries of the system. We identified 40°C seawater feed paired with ambient 6 M KOH osmotic agent provides optimal OMD thermal conditions, delivering sufficient water flux over 20 L m^{-2} h^{-1} (LMH) for H_2 production while minimising membrane scaling and wetting risks to maintain excellent long-term salt rejection. Its dual-driving-force ensures stable water production that resists latent heat losses during water vapour transport. By using external heating to maintain this optimal gradient, our OMD-AWE achieved a H_2 output of 60.2 kg m^{-2} day^{-1}, 4 times higher than $FOWS_{AWE}$, while maintaining stability over 500 h of continuous operation. To eliminate the external energy penalties at scale, we designed self-sustaining heat exchange loops through thermodynamic modelling. This thermal symbiosis captures waste heat from AWE to heat the OMD's feed to 40°C, while simultaneously cooling the AWE to maintain its temperature at ~50°C, ensuring high-efficiency, salt-free electrolysis. This integrated approach achieves best-in-class energy consumption of 51.05 kWh $kg(H_2)^{-1}$ and a market-leading cost of \$5.73 $kg(H_2)^{-1}$, competitive with pure-water electrolysis."

Additionally, we included explicit clarifications at key points in the **Results** section:

Lines 133–136: "The water flux tests were conducted using a 0.5 M sodium chloride (NaCl) feed solution at various temperatures up to 60°C (controlled by a water bath) against a permeate side maintained at room temperature (RT) of ~20°C."

Lines 288–290: "Our OMD-AWE with 0.5 M NaCl feed at 40°C (maintained by an external water bath) and a 6 M KOH osmotic agent, which shows a water flux of 22.44 LMH (Fig. 2a), driven by 4.6 kPa ΔP_v, requires J_m of 1.34 A cm^{-2} (Fig. 3a)."

Lines 308–309: "We experimentally tested this externally heated OMD-AWE system for 500 h with real seawater to assess long-term material and operational stability (Fig. 3c)."

Lines 403−404: "To assess if the waste heat can sustain thermal self-circulation, we developed a thermal-water-hydrogen model to quantify the system's thermal balance"

Lines 567–568: "To validate these, we applied the thermal-water-hydrogen model to simulate the system's dynamic response using instantaneous power as the input variable."

Regarding the thermally symbiosis concept, we would like to respectfully clarify that the novelty and validity of the thermally symbiotic architecture do not lie solely in thermodynamic calculations, but rather in a convergence of empirical validation and thermodynamic system engineering, both

of which are equally important. The establishment of this symbiotic architecture was built upon a complete narrative structure:

1. Rigorous empirical foundations

We have conducted extensive experiments, including evaluating water flux, scaling and wetting behaviours, and membrane regeneration as a function of operating temperatures, to determine the optimal thermal boundaries for OMD-AWE (as detailed in Lines 169–289 “Thermal trade-offs between water flux and stability of PTFE membranes”), which serve as the physical prerequisites that make this thermal symbiosis possible. We then seamlessly integrated the two systems by coupling water balance with temperature and vapor pressure theory (i.e., the Clausius-Clapeyron equation). This allowed us to experimentally integrate the two systems when introducing the thermal gradients and validate that the introduced thermal gradient overcomes the limitations of state-of-the-art seawater electrolysis in H_2 yield without compromising long-term material and operational stability (as shown in Lines 291–428 “Ultrafast and stable H_2 production via OMD-AWE integration”). These massive empirical datasets form the bedrock of the thermal symbiosis framework, ensuring its practical engineering feasibility in the real world.

2. Thermodynamic system modelling

Although the physical execution of the thermally symbiotic OMD-AWE system was limited by the lab-scale setup, we employed classical thermodynamic analysis to prove its feasibility and potential in enhancing energy efficiency at scale. As currently presented, this model is already comprehensive. It encompasses detailed heat transfer modelling from the system level to each individual unit, including detailed system process design, system heat flow, membrane latent and conductive heat transfer, electrolyser efficiency and waste heat generation with different materials and polarization curves, heat exchanger design, and scale-dependent heat loss analysis (as detailed in “Thermal self-circulation improves OMD-AWE energy efficiency” Lines 431–487). From this, we derived key design parameters and operating windows (e.g., temperature control point, flowrate) and system performance under the thermal symbiosis scenario and discussed its counter-intuitive design philosophies (e.g., low temperature operation, low water recovery, and high thermal demand) that break from traditional membrane distillation norms. The model also revealed the system compatibility with advanced membrane materials and various electrolysis technologies (e.g., PEM, AEM). These can be found in Lines 488–570. Furthermore, in the last revision, we expanded this section by introducing dynamic thermodynamic modelling under fluctuating renewable energy inputs (as shown in lines 585–655 “Dynamic self-regulation under intermittent renewable energy input”). This revealed the system’s unique dynamic self-regulation capability, demonstrating its superiority over traditional PVT-MD and state-of-the-art SDE-DSE (*Nature* 612, 673–678 (2022)) in managing environmental intermittency. These analyses translate the thermal symbiosis from a theoretical concept into a robust, scalable, and universally adaptable engineering paradigm.

3. Techno-economic and environmental assessment (TEA and LCA)

Through TEA and LCA, we completed the narrative arc by proving that this thermally symbiotic system simultaneously achieves lower energy consumption, lower costs, and lower environmental impacts compared to peer technologies (Lines 653–707). This establishes a pathway towards commercially viable, environmentally sustainable seawater hydrogen production at industrial scale.

Therefore, these three components, from empirical boundary determination to thermodynamic modelling and techno-economic evaluation, are all indispensable and collectively establish the transformative value of our OMD-AWE system. We believe these are sufficiently profound to prove the system's feasibility and conceptual advance. While we acknowledge that introducing granular thermodynamic calculations would provide additional engineering specifics, we carefully weighed this against the risk of overloading the text. Doing so might upset the manuscript's interdisciplinary balance and sacrifice accessibility for readers from other disciplines, such as water treatment, materials science, and electrochemistry. Given the diverse readership of *Nature Communications*, maintaining this cross-disciplinary readability is crucial for maximizing the impact and inspiring wider adoption of our proposed architecture. Furthermore, our primary expertise is rooted in water treatment, rather than thermal engineering. While the classical thermodynamic framework we have presented is sufficient to prove the system's conceptual viability, pushing into highly specialized theoretical thermal modelling exceeds our primary domain. We are concerned that using unfamiliar or overly complex thermal engineering methods without empirical validation could inadvertently introduce speculative assumptions or misleading conclusions, which we strictly wish to avoid. For these engineering specifics, our team strongly prefers an empirical approach. We are currently seeking corporate partnerships to construct a larger system to physically validate the thermally symbiotic architecture. We believe that acquiring real-world data would eventually yield a much more comprehensive, accurate, and dedicated engineering thermodynamic study. To ensure clarity for readers and avoid any conflation between our empirical tests and thermodynamic projections, we have acknowledged the limitations of laboratory-scale heat loss in the revised Manuscript:

Lines 532–536: "It should be noted that while the thermal self-circulation feasibility is validated through our modeling, laboratory-scale execution faces heat loss constraints due to unfavourable surface-to-volume ratios (Supplementary Note 4). Larger-scale pilot validation, where thermal losses are naturally minimised, will be essential to fully realise this symbiotic architecture."

Responses to comments of Reviewer 3:

This study investigated a thermally symbiotic osmotic membrane distillation-electrolysis (OMD-AWE) system for direct hydrogen production from seawater, and made 500 hours measurement actually. For a real application, it brings novel technology definitely; however, on the view of academic advance, this is only incremental rather than transformative progress. A similar program has been published as seen in Nature 612, 673–678 (2022), titled a membrane-based seawater electrolyser for hydrogen generation, in which a demonstration system was stably operated at a current density of 250 milliamperes per square centimetre for over 3,200 hours under practical application conditions, without any thermal energy input and no biofouling and extracellular polymeric substances (EPS) accumulation. At least, more durable pilot test over 3,200h may support its real availability.

Reply:

We thank you for acknowledging the novelty of our work and practical relevance of our OMD-AWE system. The conceptual advance of our work lies in the creation of a thermally symbiotic architecture that resolves the fundamental thermodynamic bottlenecks in current state-of-the-art systems. We address your concerns through the following key points:

1. The bottleneck of water flux without thermal energy input

You correctly note that the system in Xie et al. (2022) [1] operates without any thermal energy input. However, this is exactly the most critical limitation. Without available heat sources offshore, the system must rely on only isothermal osmotic gradients, resulting in extremely weak driving force for water vapor transport (i.e., vapor pressure gradient is only 0.24 kPa). Therefore, the water flux is extremely low (96% lower than our system). To match the massive water consumption of a commercial-scale electrolyser, this isothermal system requires an unfeasibly large, economically prohibitive membrane footprint. This makes its offshore deployment even more impractical due to the harsh offshore environment and limited space constraints. Their pilot test alone without economic assessment cannot support the claim of real availability for large-scale applications. In addition to water flux, their system faces reverse water flux and over-pressure risks that limited its scaling-up. We already discussed its limitations due to heat scarcity in Introduction **Lines 68–83**: "Integrating PTFE membranes to AWE (PTFE-AWE), also known as self-dampening electrolyte-assisted direct seawater electrolysis (SDE-DSE), eliminates salt passages by allowing only water vapour transport through their hydrophobic membrane pores, maintaining stability over 3,200 h[13]. However, this approach produced H_2 at a rate of 0.09% of $FOWS_{AWE}$ due to insufficient water supply, limited by its isothermal osmotic distillation (OD) creating a small 0.24 kPa vapour pressure gradient (ΔP_v). More critically, the direct coupling of OD with AWE creates inherent constraints for system optimisation and scaling, as both optimal water production and AWE conditions are not allowed. Heating the feed to activate thermally driven membrane distillation (MD) improves ΔP_v by >20 times (Supplementary Fig. 2), but its energy consumption is 17–70 times higher than RO, making it economically unfeasible. Crucially, the absence of external heat

sources in remote or offshore environments is the fundamental barrier preventing SDE-DSE from overcoming its water supply limitations, severely restricting its geographical deployment and scalability. Furthermore, operating AWE at high temperatures raises KOH's vapour pressure (P_v) above that of the feed, causing reverse vapour transport. Pressurised H_2 production is also impossible, as operating above PTFE's liquid entry pressure (LEP, 200–500 kPa[14]) leads to liquid penetration and membrane wetting."

Our advance is not adding heat but rather inventing a thermally symbiotic architecture that solves this exact bottleneck. **We must emphasize that our thermally symbiotic system also operates without external thermal energy input.** The key distinction is how we manage the system's internal thermodynamics. Our architecture repurposes inevitable waste heat from the electrolyser, transforming what was a liability requiring energy-intensive cooling into a valuable resource to drive the OMD process which simultaneously provides cooling to the electrolyser. Therefore, our system could match the zero external heat benchmark of Xie et al.'s work but delivers 25-fold higher productivity with drastically reduced footprint but 14% lower energy consumption due to higher temperature operation of AWE and elimination of active cooling. It also provides key solutions for offshore environment lacking heat sources. This conceptual advance represents not incremental heat addition, but a transformative thermodynamic paradigm shift for transitioning membrane-based seawater electrolysis from demonstration to commercial viability.

2. The 3,200-h vs. our 500-h durability test

Regarding the 3,200-h test by Xie et al., we respectfully point out that membrane fouling, scaling, and degradation are fundamentally governed by the cumulative permeate volume per unit area and the severity of concentration polarization, rather than strictly operational time [2,3]. Because our system operated at a water flux 25 times higher than their isothermal system, our membrane is subjected to a vastly accelerated throughput, which induces more severe concentration polarization at the membrane-seawater interface, exponentially increasing the thermodynamic driving force for mineral scaling and biofouling. Mathematically, the cumulative permeate volume processed during our 500-h test is equivalent to 12,500 h of operation in Xie et al.'s system, 4 times than their entire 3,200-h demonstration. Thus, our 500-h test under high-flux, thermally driven conditions actually represents a more rigorous and accelerated stress test of membrane durability than a 3,200-h test at near-zero flux.

Additionally, regarding biofouling and EPS accumulation, operating at this high flux inherently accelerates the transport and accumulation of natural organic matter (NOM) and microorganisms at the membrane surface. However, we believe our 500-h operational data provides a scientifically definitive validation of system stability based on the established "induction period" of fouling. The validity of a stability study is defined by its ability to capture the critical window during which fouling initiates and stabilizes. Research on seawater distillation at 40°C demonstrates that while a conditioning film may form within 4 h, a noticeable biofilm takes ~28 h to develop, reaching a stabilized state with only a slight decline in flux after 180 h [4–8]. Since this critical induction occurs within the first 30 to 180 h, our 500-h test represents nearly 3 times the duration of the

established observation window and is thus sufficient to demonstrate a long-term operational state. The stability observed is further reinforced by our 40°C operating temperature, which avoids the thermal stress thresholds that trigger catastrophic biofouling. Studies indicate that traditional MD systems operating at higher ranges, such as 60–65°C, trigger significant thermal stress in marine microbes, leading to the hyper-secretion of EPS [4,6]. Higher temperatures of 70°C can thermally convert humic substances into low-molecular-weight organics, increasing the concentration of assimilable organic carbon and accelerating biofilm growth [7]. Studies confirms that increasing the temperature from 45°C to 65°C can trigger a 2.5-fold increase in biofilm thickness, acting as both a physical barrier and a thermal insulator that cause flux declines of up to 79% [8]. By maintaining a 40°C environment, our system ensures a thinner and more stable biological interface that remains highly reversible and compatible with mild, non-invasive cleaning protocols [9].

To rigorously evaluate our system under realistic conditions, our 500-h test used raw, biologically active seawater. While we did observe the accumulation of a mixed organic-inorganic foulant layer, our comprehensive post-mortem analyses (SEM, EDX, XRD, XPS, AFM) proved that this fouling is highly reversible. We discovered that the natural organic matter in real seawater mitigates hard inorganic scaling by altering crystal morphology, ensuring the membrane can be fully regenerated without any permanent degradation to the PTFE polymer's hydrophobicity or structure. While Xie et al. demonstrated stability at ambient conditions with low flux, our work proves that we can achieve a 25-fold increase in water production while staying safely below the thermal thresholds that trigger irreversible biofouling. Consequently, because the membrane can be restored to its pristine state using standard cleaning protocols, this 500-h milestone is merely a starting point; it definitively proves that the system can sustain stable operation well beyond this timeframe.

These are added to **Manuscript Lines 326–328: "**Operating at a moderate 40°C avoids the microbial thermal stress typical of high-temperature MD (60–70°C), which triggers EPS hyper-secretion and catastrophic biofouling. This ensures a thin, stable biological interface." and **Lines 355–361:** "Since membrane fouling depends on cumulative permeate volume rather than strictly operational time, our 500-h test at >25-fold higher water flux than SDE-DSE extracted permeate equivalent to >12,500 h of isothermal operation, nearly 4 times that of previous 3,200-h demonstrations. Combined with the proven reversibility of such accelerated fouling, this represents a significantly more rigorous validation of membrane durability, confirming that the system can sustain robust operation well beyond 500 h through routine maintenance."

3. Other advances beyond isothermal systems

To further clarify why our architecture represents a fundamental conceptual advance rather than an incremental integration, we highlight several transformative capabilities absent in SDE-DSE:

We enhanced our analysis by including system stability under fluctuating energy inputs. Beyond the absence of external heat sources being a barrier for SDE-DSE systems, energy fluctuations pose another critical challenge. Their system's water production relies solely on constant osmotic gradients, while electrolysis water consumption varies with fluctuating energy input, causing water

balance collapse where production fails to match consumption over time. This imbalance leads to either system dry-out, which causes corrosion, localized overheating, and KOH crystallization that blocks electrolyzer flow channels, or electrolyte dilution, which increases ohmic resistance, collapses the osmotic driving force for subsequent high-power operation, and requires additional energy-intensive separation processes. Our thermally symbiotic system establishes an intrinsic self-regulating mechanism that overcomes renewable intermittency. Since electrolyzer waste heat generation is directly proportional to applied electrical current, the thermal driving force for water production synchronizes with electrical demand for water consumption during intermittent power input. This creates an inherent feedback loop that maintains water balance automatically. Details of this self-regulation are provided our **Manuscript Lines 547–614** **Dynamic self-regulation under intermittent renewable energy input**. This further highlights the transformative nature of our thermally symbiotic architecture, which provides the first truly viable pathway for seawater H_2 production.

Compared to SDE-DSE, our thermally symbiotic OMD-AWE system demonstrates energetic and economic advantages as revealed by techno-economic analysis (TEA), and life-cycle assessment (LCA). OMD-AWE achieves specific energy consumption of 51.05 kWh $kg(H_2)^{-1}$, which is 14% lower than that of SDE-DSE due to the operational temperature constraints, and three times more balance-of-plant (BoP) electricity for active cooling. Consequently, OMD-AWE proves to be substantially more cost-effective, delivering a Levelised Cost of Hydrogen (LCOH) of \$5.73 $kg(H_2)^{-1}$ (at an electricity price of \$0.1 kWh^{-1}), which represents a 18% cost reduction compared to SDE-DSE. Furthermore, LCA shows that OMD-AWE generates 10% to 37% lower overall environmental impacts than SDE-DSE, driven by its superior energy efficiency and the elimination of external cooling infrastructure. These are detailed in **Manuscript Lines 616–670** **Comparative techno-economic and environmental assessment.** These results reveal that relying solely on isothermal osmotic driving forces imposes energetic and economic penalties. By unifying reaction inefficiencies (waste heat) and mass-transfer demands (water separation) into a self-sustaining thermodynamic loop, our OMD-AWE architecture provides a more scalable, cost-effective, and environmentally sustainable pathway for commercial seawater hydrogen production.

Beyond seawater H_2 production, our thermally symbiotic architecture establishes a transformative design philosophy with profound implications for the broader scientific community. Although MD was widely recognised for its exceptional salt rejection, its industrial application has been limited by extensive thermal requirements. Unlike widely adopted reverse osmosis, MD has been trapped in a developmental bottleneck for decades. The field has either remained entirely dependent on co-location with external industrial waste heat sources or attempted to improve thermal efficiency through complex module designs and novel photothermal materials, both of which offer limited practical success. This reliance on external heat restricts the geographical deployment and scalability of MD, especially in remote or offshore environments where external heat sources are unavailable. Our work provides a new and viable direction for MD by directly coupling it with electrolysis. We demonstrate that MD and AWE function as perfect thermodynamic complements,

i.e., the waste heat generated by AWE matches the thermal driving force required by MD which simultaneously provides cooling for AWE to maintain its temperatures. This coupling eliminates the need for external heat, transforming MD from a heat-dependent technology into a self-sustaining solution. Furthermore, our thermal symbiosis extends far beyond dedicated H_2 systems. This establishes a universal design philosophy that can inspire next-generation water-energy technologies. For example, this architecture can be extended to other exothermic electrochemical processes, like electrochemical CO_2 reduction and ammonia synthesis. In CO_2 reduction, large kinetic overpotentials inevitably generate substantial waste heat, while the downstream separation of liquid products (e.g., ethanol, formate) from the aqueous electrolyte remains a massive energy bottleneck. By applying our thermally symbiotic architecture, this waste heat could be recycled to drive membrane-based pervaporation or distillation, achieving in situ product recovery without external thermal input. Similarly, in electrochemical mineral recovery (e.g., lithium extraction), the substantial Joule heat generated during ion capture can be repurposed to drive membrane distillation processes that simultaneously provide cooling and extract pure water from brine. This in situ generated pure water serves as the receiving solution to release and concentrate extracted minerals, while continuous water removal drives residual brine towards zero-liquid discharge. Across all these applications, this architecture shifts the traditional chemical engineering paradigm by unifying reaction inefficiencies (waste heat) and mass-transfer demands (separation) into a self-sustaining thermodynamic loop, rather than treating them as isolated energy penalties. There are presented in our **Conclusion** **Lines 674–677**: "By coupling OMD and AWE as perfect thermodynamic complements, this architecture shifts the paradigm of seawater splitting from complex catalyst engineering to thermodynamic system design, simultaneously solving the heat-dependency bottleneck of membrane distillation and overcoming the low H_2 yield and catalyst durability limitations of direct seawater electrolysis." and **Lines 691–699**: "Beyond H_2 production, this thermal symbiosis architecture establishes a universal thermodynamic framework for other water-energy nexus challenges. By coupling exothermic electrochemical processes, e.g., CO_2 reduction, ammonia synthesis, and lithium extraction, with thermally-driven separations, this architecture repurposes parasitic Joule heating to drive MD-based distillation. In doing so, the system simultaneously provides essential passive cooling, recovers liquid chemical products, and extracts pure water to push residual brines toward zero-liquid discharge. This architecture shifts the traditional chemical engineering paradigm, transforming isolated engineering liabilities into self-sustaining resource mining and chemical manufacturing engines."

References:

1. Xie, H., Zhao, Z., Liu, T., Wu, Y., Lan, C., Jiang, W., Zhu, L., Wang, Y., Yang, D. & Shao, Z. A membrane-based seawater electrolyser for hydrogen generation. *Nature* **612**, 673–678 (2022). https://doi.org/10.1038/s41586-022-05379-5

2. L Martínez-Díez, M.I Vázquez-González, Temperature and concentration polarization in membrane distillation of aqueous salt solutions, *Journal of Membrane Science,* 156, 265-273, 0376-7388 (1999). https://doi.org/10.1016/S0376-7388(98)00349-4.

3. Omkar R. Lokare and Radisav D. Vidic, Impact of Operating Conditions on Measured and Predicted Concentration Polarization in Membrane Distillation, *Environmental Science & Technology, 53* (20), 11869-11876 (2019). https://doi.org/10.1021/acs.est.9b04182.

4. Ng, Y. S. & Chan, D. J. C. Thermal Effect on Algae, Biofilm and Their Composition Towards Membrane Distillation Unit: A Mini-review. *Mol. Biotechnol*. (2023). https://doi.org/10.1007/s12033-023-00853-5

5. Bogler, A. & Bar-Zeev, E. Membrane Distillation Biofouling: Impact of Feedwater Temperature on Biofilm Characteristics and Membrane Performance. Environ. Sci. Technol. 52, 10019–10029 (2018). https://doi.org/10.1021/acs.est.8b02744

6. Zodrow, K. R., Bar-Zeev, E., Giannetto, M. J. & Elimelech, M. Biofouling and Microbial Communities in Membrane Distillation and Reverse Osmosis. Environ. Sci. Technol. 48, 13155–13164 (2014). https://doi.org/10.1021/es503051t

7. Naidu, G., Jeong, S. & Vigneswaran, S. Interaction of humic substances on fouling in membrane distillation for seawater desalination. Chemical Engineering Journal 262, 946–957 (2015). https://doi.org/10.1016/j.cej.2014.10.060

8. Elcik, H., Alpatova, A., Gonzalez-Gil, G., Blankert, B., Farhat, N., Amin, N. A., Vrouwenvelder, J. S. & Ghaffour, N. Elucidating biofouling over thermal and spatial gradients in seawater membrane distillation in hot climatic conditions. Water Res. 223, 118983 (2022). https://doi.org/10.1016/j.watres.2022.118983

9. Mpala, T. J., Etale, A., Richards, H. & Nthunya, L. N. Biofouling phenomena in membrane distillation: mechanisms and mitigation strategies. Environmental Science: Advances 2, 39–54 (2023). https://doi.org/10.1039/D2VA00161F

Title: Thermally symbiotic integration of osmotic membrane distillation and electrolysis for direct seawater hydrogen production

Authors: Gabriela Scheibel Cassol, Chii Shang, Paul Westerhoff, Yinghao Song

Manuscript ID: NCOMMS-25-36631C

Responses to comments of Reviewer 1:

The revisions made to the manuscript are clear and well-justified, and the authors have responded in a thorough and professional manner. I have no further comments at this stage and am pleased to recommend the manuscript for acceptance in its current revised form. I would like to thank the authors for their time and effort in preparing the detailed response and for the quality of the revised work.

Reply:

We deeply appreciate the significant time, dedication, and expertise you volunteered to evaluate our study and guide us toward a much stronger final paper.

Responses to comments of Reviewer 3:

I agree the point of view that an osmotic membrane distillation need continuous heat energy input, and it is exactly the most critical limitation for MD without thermal energy supply. A system simulation is also useful for promoting its engineering proceeding of OMD-AWE to hydrogen production with direct seawater electrolysis in alkaline condition. Although that, I have reviewed this manuscript many times, and find almost no obvious change of manuscript's novelty. I believe that it is difficulty to improve its novelty only based on the idea of thermally symbiotic integration of osmotic membrane distillation process. For this reason, this manuscript maybe suitable for professional journals due to the lack of conceptual framework innovation to meet the requirement of Nature Communications publications.

Reply:

We sincerely thank you for sustained dedication and thorough evaluation of our manuscript through multiple rounds of revision. We highly value the recognition that our system is useful for promoting the engineering advancement of seawater hydrogen production, which motivates us to continue our efforts in this important area. We appreciate all the constructive feedback throughout the review process, which has helped us strengthen our work significantly.
